# Detection of False-Positive Deletions from the Database of Genomic Variants

**DOI:** 10.1155/2019/8420547

**Published:** 2019-04-04

**Authors:** Junbo Duan, Han Liu, Lanling Zhao, Xiguo Yuan, Yu-Ping Wang, Mingxi Wan

**Affiliations:** ^1^Department of Biomedical Engineering, Xi'an Jiaotong University, Xi'an, China; ^2^School of Computer Science and Technology, Xidian University, Xi'an, China; ^3^Department of Biomedical Engineering, Tulane University, New Orleans, USA

## Abstract

Next generation sequencing is an emerging technology that has been widely used in the detection of genomic variants. However, since its depth of coverage, a main signature used for variant calling, is affected greatly by biases such as GC content and mappability, some callings are false positives. In this study, we utilized paired-end read mapping, another signature that is not affected by the aforementioned biases, to detect false-positive deletions in the database of genomic variants. We first identified 1923 suspicious variants that may be false positives and then conducted validation studies on each suspicious variant, which detected 583 false-positive deletions. Finally we analysed the distribution of these false positives by chromosome, sample, and size. Hopefully, incorrect documentation and annotations in downstream studies can be avoided by correcting these false positives in public repositories.

## 1. Introduction

A genomic variant is an alteration of the DNA sequence of an organism. Since an organism's DNA sequence encodes the genetic instructions used in its development, any alteration of this sequence may cause genetic abnormalities or even fatality. According to their sizes, genomic variants are classified into small-scale variants, such as single nucleotide polymorphisms (SNP) and indels, and large-scale variants, namely, structural variations (SV), including copy number variations (CNV), insertions, deletions, inversions, segmental duplications, and translocations [1]. Various complex diseases have been reported to be associated with genomic variants in human genomes [2].

Prior to next generation sequencing (NGS), cytogenetic techniques, such as fluorescence* in situ* hybridization (FISH) and array comparative genomic hybridization (aCGH), were employed to detect SV. However, due to their relatively low genomic resolution (e.g., microscopic scale (Mbp) for FISH, and submicroscopic scale (kbp) for aCGH) [3], most medical and biological research teams have migrated their platforms to NGS, which can provide base-pair level resolution.

Several approaches have been proposed to detect SV from NGS data. Generally, these approaches can be classified into two categories: paired-end read mapping (PEM)-based or depth of coverage (DOC)-based approaches [4]. For PEM-based approaches, if the span of a pair of mapped reads is longer/shorter than a specified cutoff related to the insert size of the sequencing library, a deletion/insertion can be identified [5], whereas for DOC-based approaches, if the local depth of reads is significantly larger/smaller than the global DOC, a duplication/deletion can be identified [3, 6, 7]. PEM-based approaches have advantage of detecting balanced SVs (inversion) and unbalanced SVs (deletion and insertion) of relatively small sizes, whereas DOC-based approaches are good at detecting unbalanced SVs (CNV) of relatively large sizes. Besides PEM and DOC, several other supplementary signatures, such as split read mapping, have been combined into PEM and DOC to improve detection performance, leading to integrative models [8–10].

Despite PEM's advantage in balanced SV detection, the majority of detection approaches use DOC as the primary signature to identify CNVs [11]. However, the DOC signature is biased due to two main factors: GC content [12] and mappability [13].

- Since G and C form a triple hydrogen bond (whereas A and T form a double bound), theoretically the melting temperature of GC-rich segments is approximately 2°C higher than that of AT-rich segments [14]. As a result, when the sequencing protocol involves polymerase chain reaction (PCR), GC-rich segments and AT-rich ones are unevenly amplified [15], yielding the correlation between DOC and GC content [16, 17].
- Due to the complexity of the human genome, there are regions in which sequenced short reads cannot be uniquely mapped, e.g., repeated regions, such as retrotransposons (LINE and SINE) [13]. Mappability was introduced to measure the uniqueness of such regions using a score that ranges from 0 to 1, corresponding to highly repeated and unique regions, respectively. From the definition, it is clear that the DOC is correlated with mappability.

In human genomes, both GC content and mappability are distributed unevenly along chromosomes, and, therefore, they introduce biases into DOC. Several methods have been developed to correct these two biases [13, 18, 19].

However, due to the overlook of biases introduced by GC content and mappability, some DOC-based SV studies contain false detection. From the mechanism of PEM and DOC signatures, it is clear that PEM is less affected by GC content or mappability than DOC, and, therefore, PEM can be used to detect false positives. In this paper, we used this idea to verify the entries in the database of genomic variants (DGV). We hope that incorrect documentation and annotations can be avoided in downstream studies by correcting the false positives in this public repository and other related ones such as EMBL-EBI's Genomic Variants archive (DGVa) and NCBI's dbVar.

## 2. Materials and Methods

### 2.1. Samples and Data

The database of genomic variants (DGV, http://dgv.tcag.ca/dgv/app/home) [20] provides a comprehensive summary of structural variation (SV) in the human genome. In DGV, SVs are defined as genomic alterations that involve segments of DNA with length larger than 50 bp. In DGV, the 6.4 millions of variants represent collections from 55 thousand healthy control samples in 72 studies. DGV provides a curated catalogue of genomic variations in the human genome, which was integrated into EMBL-EBI's Genomic Variants archive (DGVa) and NCBI's dbVar. Therefore, this database is of tremendous importance to investigators whose study interest is about genomic variance, which is also the focus of the current study.

The 1000 Genomes Project (http://www.1000genomes.org/ [21]) is a well-known international collaborative NGS project, which aims to sequence the genomes of approximately 2500 people from 25 populations around the world. In our study, the BGZF compressed sequence alignment/map (SAM) data files (BAM) of most samples were downloaded from the website of this project as the primary dataset.

Sequence read archive (SRA, https://www.ncbi.nlm.nih.gov/sra) is the NCBI database that stores raw sequencing data obtained from NGS technology. The aim of SRA is to make NGS data available to researchers to both improve reproducibility and enable new discoveries. This database includes data from most common sequencing platforms and most NGS studies. The BAM file of some specific samples in our study was not available, so the SRA files were downloaded as the primary dataset.

### 2.2. Methods

The steps conducted in the study are shown as follows, and the pseudocode is listed in Pseudocode 1 to illustrate the logical structure of these steps.

1. The latest spreadsheet of supporting variants was downloaded from the DGV website. The GC content and mappability profile of each chromosome of hg18 were downloaded from the readDepth website (https://github.com/chrisamiller/readdepth) [18], which is an NGS-based CNV detection software package.
2. GC content profiles were smoothed with LOESS [22], and segments with size larger than 500 bp and an average GC content lower than *th*_1_ = 0.26 or greater than *th*_2_ = 0.59 were obtained as suspicious regions.
3. Mappability profiles were smoothed with LOESS, and segments with size larger than 500 bp and an average mappability lower than *th*_3_ = 0.92 were added to suspicious regions.
4. All supporting variants in DGV were resolved one-by-one to collect the fields needed in the current study, including variant accession ID, chromosome, genomic location (starting and ending loci), variant subtype, reference, method, and samples used.
5. Variants associated with the* sequencing* method and* loss* or* deletion* subtype, size smaller than 10 kbp, and nonempty sample fields were filtered for further analysis.
6. Duplicated variants were merged.
7. GDV variants that overlapped (F-score greater than 0.9) with suspicious regions were identified as suspicious variants.
8. For each suspicious variant, the corresponding BAM file was downloaded. If no BAM file was available, the corresponding SRA file was downloaded, aligned with BWA, compressed, and sorted with SAMtools to obtain a BAM file.
9. For each suspicious variant, both the PEM and DOC signatures were extracted from the corresponding BAM file.
10. The PEM signature was used to verify whether this suspicious variant was a false-positive or true variant (see Section 2.3).
11. Finally, the GC content, mappability, DOC, and PEM profiles of each false positive were displayed in an individual figure for visual inspection, and information of all suspicious variants was outputted to a spreadsheet.

Here are some notes that should be addressed:

- A* supporting variant* represents a variant called in a single sample/individual, which can also be described as sample level variant [20].
- One sample is the minimal requirement to verify a specific supporting variant, so the sample field should be nonempty.
- The terms* deletion* and* loss* are equivalent in the database [20].
- Duplicated variants are defined in the sense of the same chromosome ID and genomic location.
- The* corresponding* BAM or SRA file of a suspicious variant was retrieved according to the* sample* and* reference* field.

### 2.3. Validation

The validation of variants is based on the PEM signature. First, for each suspicious variant, we extracted all the read pairs in which both ends were mapped within the region of interest (ROI) from the corresponding BAM file. To provide an adaptive zoom, the ROI is defined as the genomic region that extends both upstream and downstream with 1 kbp plus half of the variant length.

**Figure 1:**
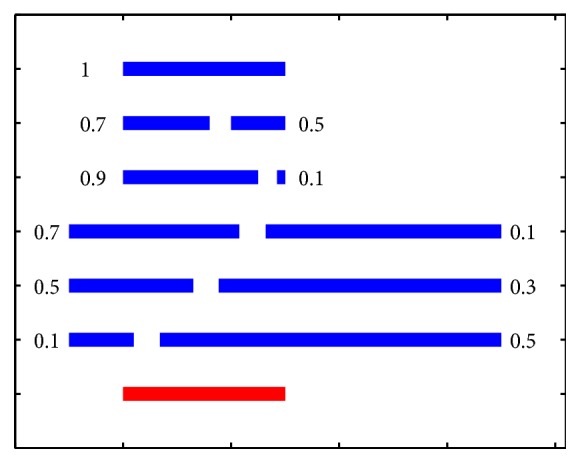
An illustration between F-score and overlapping quality. The bottom red span is the reference, and the 11 blue spans are tests, whose F-scores are shown with respect to the reference, ranging from 0.1 (very bad overlapping) to 1 (perfect one).

Next, the F-score [23] is employed to measure the overlapping quality between the span of a suspicious variant and that of a mapped read pair. The F-scores quantify the overlap quality between two spans, with values ranging from 0 to 1 (see Figure 1, which demonstrates several typical scores). A small value close to 0 means a bad overlap, whereas a high value close to 1 means a good overlap. The F-score is calculated as follows: for a test span, if it has no overlap with the reference span, the F-score is set to 0; otherwise, *F* = 2(*PR*/(*P* + *R*)), where *P* is the precision (percentage of the test span that overlaps with the reference span) and *R* is the recall (percentage of the reference span that overlaps with the test span).

**Figure 2:**
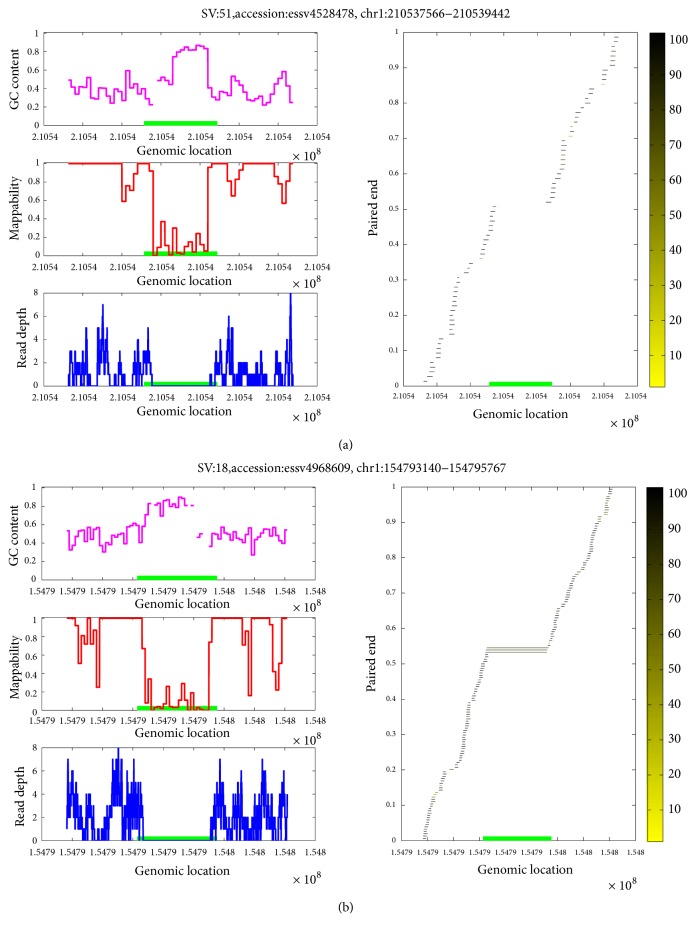
Two examples of suspicious variants. (a) A false positive and (b) a true positive of sample NA18507. The left upper, middle, and lower panels of each subfigure display the profiles of GC content, mappability, and DOC, respectively; the right panel displays the PEM profile, and each horizontal line represents a read pair, where the face colour encodes the mapping quality (yellow and black represent low and high mapping quality, respectively). The green bar in each panel is the studied DGV variant.

In our study, mapped read pairs with F-scores larger than 0.7 were selected, and the average and sum of the mapping quality of all selected pairs were calculated. A suspicious variant was identified as a true positive if the average and sum were above 30 and 90, respectively; otherwise, it was classified as a false positive. Therefore, a pair with a mapping quality of 90, two pairs with mapping quality of 45, or three pairs with a mapping quality of 30 constitute the minimal requirement to confirm a true positive.  Figure 2 demonstrates two typical examples. It is shown that both regions have high GC content and low mappability; (a) shows no PEM signature while (b) does. Therefore, (a) is a false positive, and (b) is a true positive.

## 3. Results

We identified a total of 1923 suspicious variants, which cluster in 7 samples from 8 studies (see Figure 4(b) and Table 1). Among these 7 samples, the BAM files of four samples (NA18507, NA18505, NA12156, and NA10851) were downloaded from the FTP site of the 1000 Genomes Project (ftp://ftp-trace.ncbi.nih.gov/1000genomes/). For other three samples, i.e., YH, HuRef, and NA15510, sequencing data were downloaded: the paired reads of sample YH were downloaded from the FTP site of the YanHuang Project (ftp://public.genomics.org.cn/BGI/yanhuang/), and the SRAs of samples HuRef and NA15510 were downloaded from the NCBI FTP site using the* sra-toolkit* package.

BWA [30] was used to align short sequencing data to the reference genome hg18. Here, human reference genome hg18 was used in order to have a consistent genomic coordinate with the downloaded BAM files from the 1000 Genomes Project. The maximum insert size (*-a* option of BWA) was set to 1e4.

From these 1923 suspicious variants, 583 were detected as false positives. Complete information on suspicious variants and false positives is listed in Supplementary Table S1, and the validation figures of each false positive are shown in the supplementary FIG directory. Two typical examples (a false positive and a true positive) and statistical analysis are shown in the following examples.

Figure 2 shows two typical suspicious variants from NA18507.  Figure 2(a) shows the variant with accession essv4528478, whose genomic location is chr1:210537566-210539442 (green bar in all panels). It is shown that both GC content (the magenta curve in the upper left panel) and mappability (the red curve in the middle left panel) profiles at the ROI are abnormal, and hence they yield a valley in the DOC profile (the blue curve in the lower left panel). As a result, a deletion variant was detected in the DOC profile. However, the PEM signature (the right panel) contains no mapped read pairs (the horizontal lines) that overlap with the green bar, suggesting that this suspicious variant is a false positive. In contrast, Figure 2(b) shows the variant with accession essv4968609 (genomic location chr1:154793140-154795767), whose GC content, mappability, and DOC profiles show similar behaviours to those in (a). However, the PEM signature contains well-mapped read pairs (three black lines in the centre) that overlap with the green bar, suggesting a true positive.

**Figure 3:**
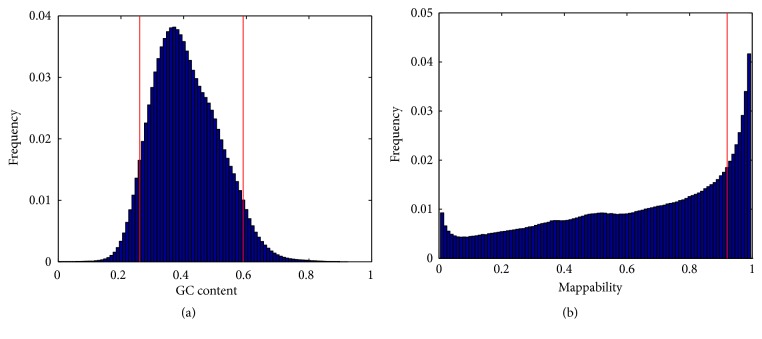
The GC content (a) and mappability (b) distribution of human genome (hg18). The three vertical red lines represent thresholds *th*_1_ = 0.26, *th*_2_ = 0.59, and *th*_3_ = 0.92.

Figure 3(a) shows the distribution of the GC content of the human genome (hg18) with a bin size of 100 bp. Based on this distribution, the threshold values *th*_1_ = 0.26 and *th*_2_ = 0.59 are used to determine the estimated extreme GC content regions, such that both the left and right tail areas cover 5% of the whole distribution.

Figure 3(b) shows the distribution of the mappability of the human genome (hg18) with a bin size of 100 bp. Since exact 1 and 0 mappability values occupy a large portion of the distribution (68% and 7%, respectively), these two values were excluded from the distribution. Based on the remaining values, the threshold value *th*_3_ = 0.92 was chosen such that the right area covers 20% of the distribution.

**Figure 4:**
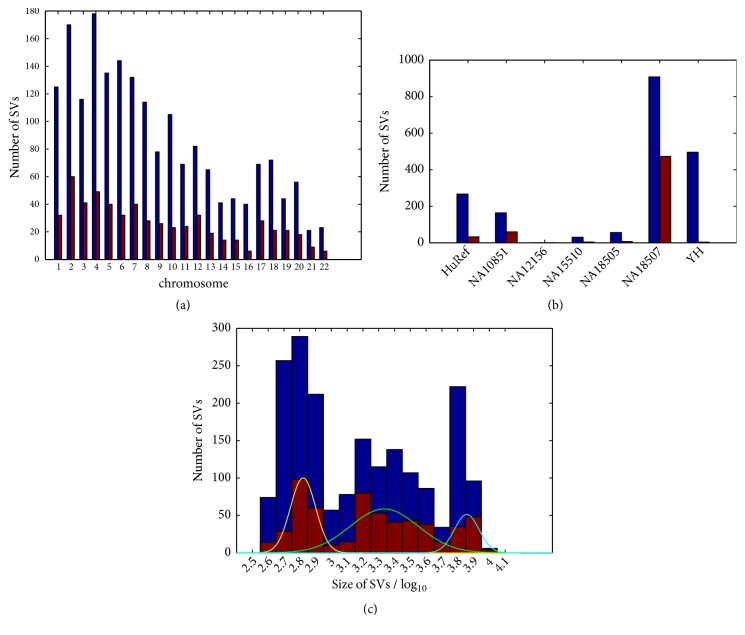
The distributions of variants with respect to the chromosome (a), sample (b), and size (c). Blue and red bars represent the suspicious and false-positive variants, respectively.

Figure 4(a) shows the distribution of suspicious variants and false positives across chromosomes. It is shown that the number of false positives decreases with respect to the chromosome index number. The correlation analysis between the chromosome lengths and the number of false positives yielded the correlation coefficient *r* = 0.83 and *p*-value of 1.8e-6, indicating that the false positives are distributed evenly among chromosomes.

Figure 4(b) shows the distribution of suspicious variants and false positives across samples. It is shown that NA18507 contains more suspicious variants (908) and false positives (473) than the other samples. We further analysed the distribution of suspicious variants and false positives across studies and found that the studies by ‘Bentley et al. 2008' [16] and ‘McKernan et al. 2009' [27] contained more false positives (289 and 184) than the other studies, and all of these false positives came from the sample NA18507.

Figure 4(c) shows the distribution of suspicious variants and false positives with respect to the size. Three modalities are shown: the left one with size smaller than 1 kbp (3), the middle one with size between 1 kbp and 5 kbp (3.7), and the right one with size larger than 5 kbp, which have proportions of 36%, 46%, and 18%, respectively. These results indicate that most false positives are small- or medium- sized variants. By fitting each modality with a Gaussian curve, the means of the three modalities are 660 bp (2.8), 2.2 kbp (3.3), and 7.2 kbp (3.9) for the left, middle, and right modalities, respectively.

## 4. Conclusion and Discussion

We proposed an approach to detect GC content and mappability related to false positives from the database of genomic variants. The proposed approach utilized the PEM signature, whose presence is necessary and provides evidence for true positives. 583 false positives were detected by conducting a validation study on the database of genomic variant. The results can avoid incorrect documentation and annotations in downstream studies.

We excluded variants with sizes larger than 10 kbp in this study, and the reasons are as follows: for most NGS alignment/mapping tools, the maximal insert size is limited to thousands of base pairs; e.g., the default values of both the* -a* parameter in BWA and* -X* parameter in Bowtie 2 are 500 bp, and a very large insert size degrades mapping performance. However, large variants (deletions and inversions) do require a large insert size. As a result, there is a conflict between the mapping quality and the maximal size of detectable variants, and a tradeoff has to be taken with caution. Therefore, we confined the maximal variant size to 10 kbp.

In this study, since there are several software packages/algorithms (e.g., smoothing, segmentation, mapping, etc.) and parameters (F-score, thresholds *th*_1_, *th*_2_, *th*_3_, etc.) that are used in the method, the robustness is an important issue. A global optimization of all parameters is not conducted due to the larger number of parameters, but we set each software/algorithm to its recommended setting and tune each parameter separately to a reasonable value (e.g., th1, th2, and th3). We used the F-score to identify whether a segment overlaps with another segment. From Figure 1, we can see that a threshold value of 0.7 is appropriate to determine that two segments are almost overlapping with each other, so we used 0.7 in the validation step to determine an overlap status. When it is increased to 0.75, 644 false positives are detected, and when it is decreased to 0.65, 546 false positives are detected. Therefore, the results are roughly robust with respect to this parameter. In step 7, we used a large threshold value of 0.9 in order to narrow down the total number of suspicious variants to be validated. We also used three threshold values *th*_*i*_  (*i* = 1,2, 3) to identify suspicious regions. In Figure 3(a), we chose *th*_1_ = 0.26 and *th*_2_ = 0.59 so that both the left and right tail areas covered 5% (or 10% in total) of the whole distribution, whereas in (b) we chose the threshold value *th*_3_ = 0.92 so that the right area covered 20% of the distribution. Since the distribution of GC content is close to a Gaussian distribution, tail areas with 10% are appropriate. However, the distribution of mappability is far from a Gaussian distribution, and values other than 1 are unfavourable. When we adopted the strategy used for GC content to mappability, i.e., the left tail area covering 10%, the resultant threshold value was *th*_3_, which was too tight to identify suspicious variants. Therefore, we chose 20% for the right area, which yielded a much looser threshold.

There are two limitations of the current study. First, since the PEM signature is more straightforward for studying deletion than other types of variants, the current study focuses on only this type of variant. In future studies, we hope to extend the spectrum of variants being studied. Second, this study focused on only the database of genomic variants as a pilot study to validate our method. In future works, we hope to conduct large-scale validations on other well-known public repositories related to structural variants, such as dbVar (https://www.ncbi.nlm.nih.gov/dbvar), which is NCBI's database of human genomic structural variation, and the Database of Genomic Variants archive (DGVa, https://www.ebi.ac.uk/dgva), which is an EMBL-EBI database that archives publicly available genomic structural variants of all species.

## Figures and Tables

**Pseudocode 1:**
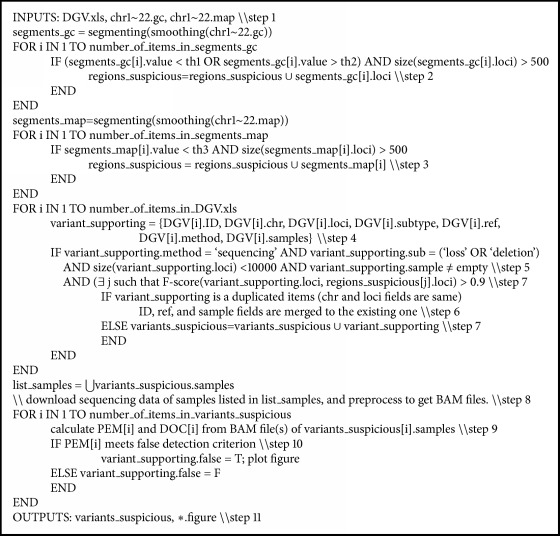
Pseudocode of processing pipeline.

**Table 1:** The samples and associated studies.

Sample	Study
HuRef	Levy et al. 2007 [24], Pang et al. 2010 [25]
NA10851	Ju et al. 2010 [26]
NA15510, NA18505	Korbel et al. 2007 [5]
NA18507	Bentley et al. 2008 [16], McKernan et al. 2009[27]
YH	Wang et al. 2008 [28]
NA12156	Kidd et al. 2008 [29]

## Data Availability

The data used to support the findings of this study are available from the corresponding author upon request.

## Supplementary Materials

Supplementary Materials Supplementary data are available with this article at http://gr.xjtu.edu.cn/c/document_library/get_file?p_l_id=2403541&folderId=2539941&name=DLFE-115097.zip. Table S1 lists the complete information of suspicious variants and false positives, and the FIG directory contains the validation figures of each false positive.
