# Supplementary figures and images for: Detection of False-Positive Deletions from the Database of Genomic Variants (part 2 of 2)
Source: Biomed Res Int. 2019 Apr 4;2019:8420547. doi: 10.1155/2019/8420547 (PMC6475568; doi:10.1155/2019/8420547)

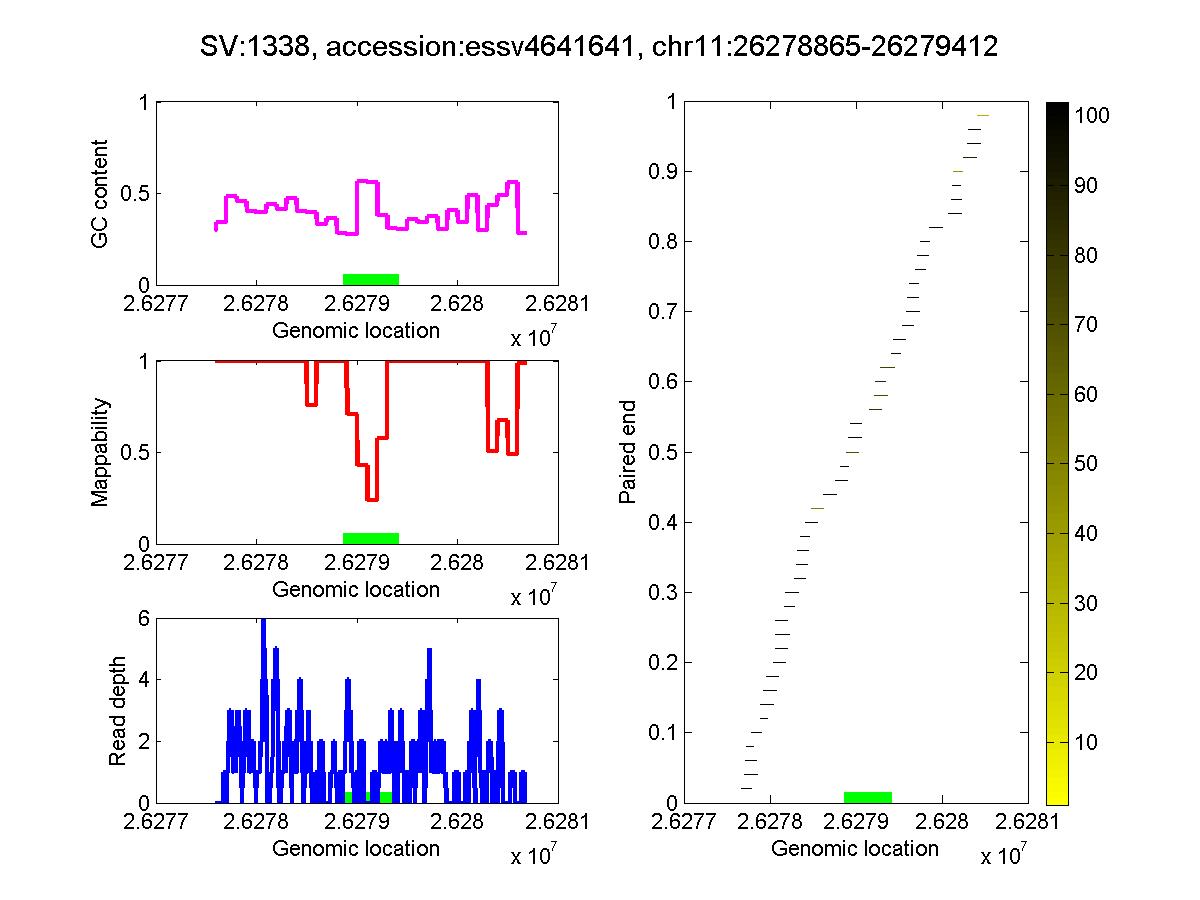

Supplement: Supplementary Materials — Supplementary data are available with this article at http://gr.xjtu.edu.cn/c/document_library/get_file?p_l_id=2403541&folderId=2539941&name=DLFE-115097.zip. Table S1 lists the complete information of suspicious variants and false positives, and the FIG directory contains the validation figures of each false positive. [file 8420547.f1.zip › 8420547.f1/FIG/SV1338.jpg]

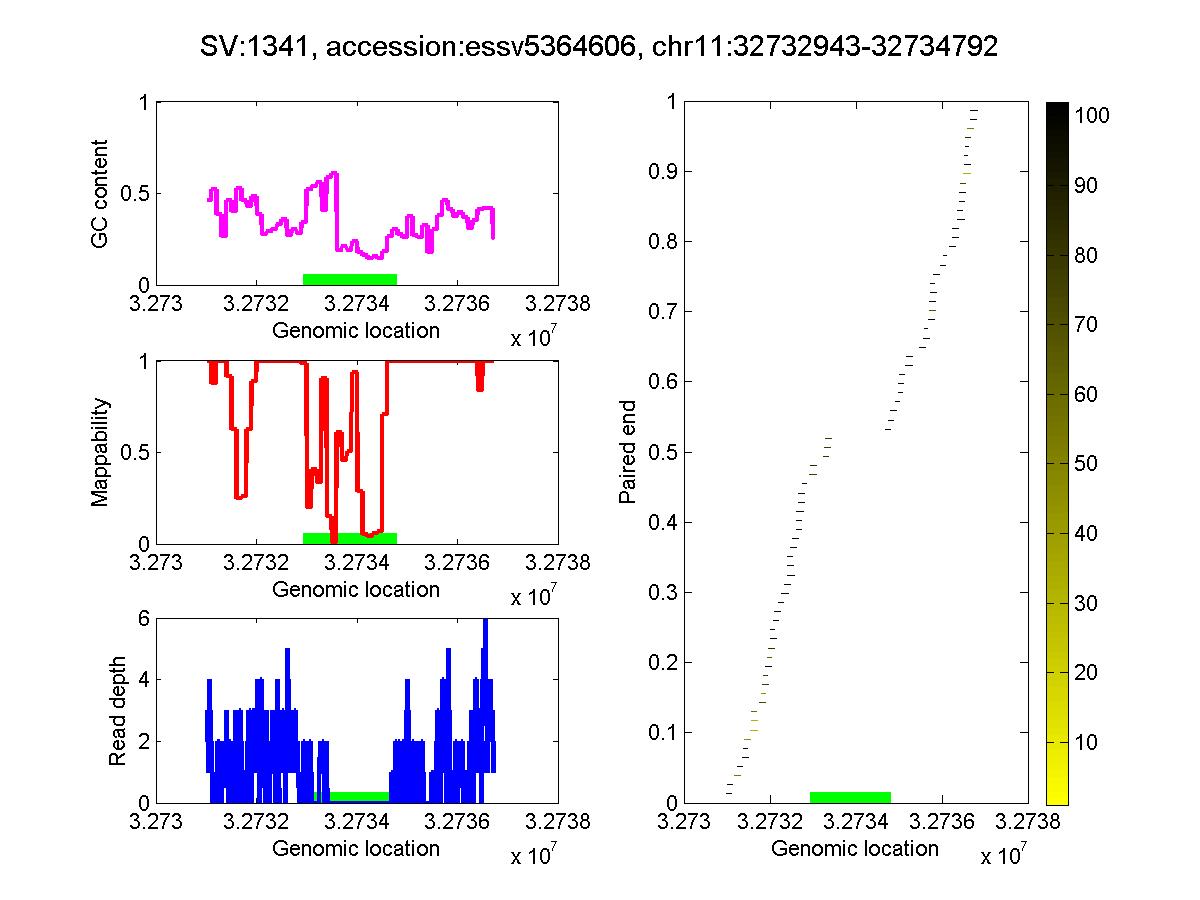

Supplement: Supplementary Materials — Supplementary data are available with this article at http://gr.xjtu.edu.cn/c/document_library/get_file?p_l_id=2403541&folderId=2539941&name=DLFE-115097.zip. Table S1 lists the complete information of suspicious variants and false positives, and the FIG directory contains the validation figures of each false positive. [file 8420547.f1.zip › 8420547.f1/FIG/SV1341.jpg]

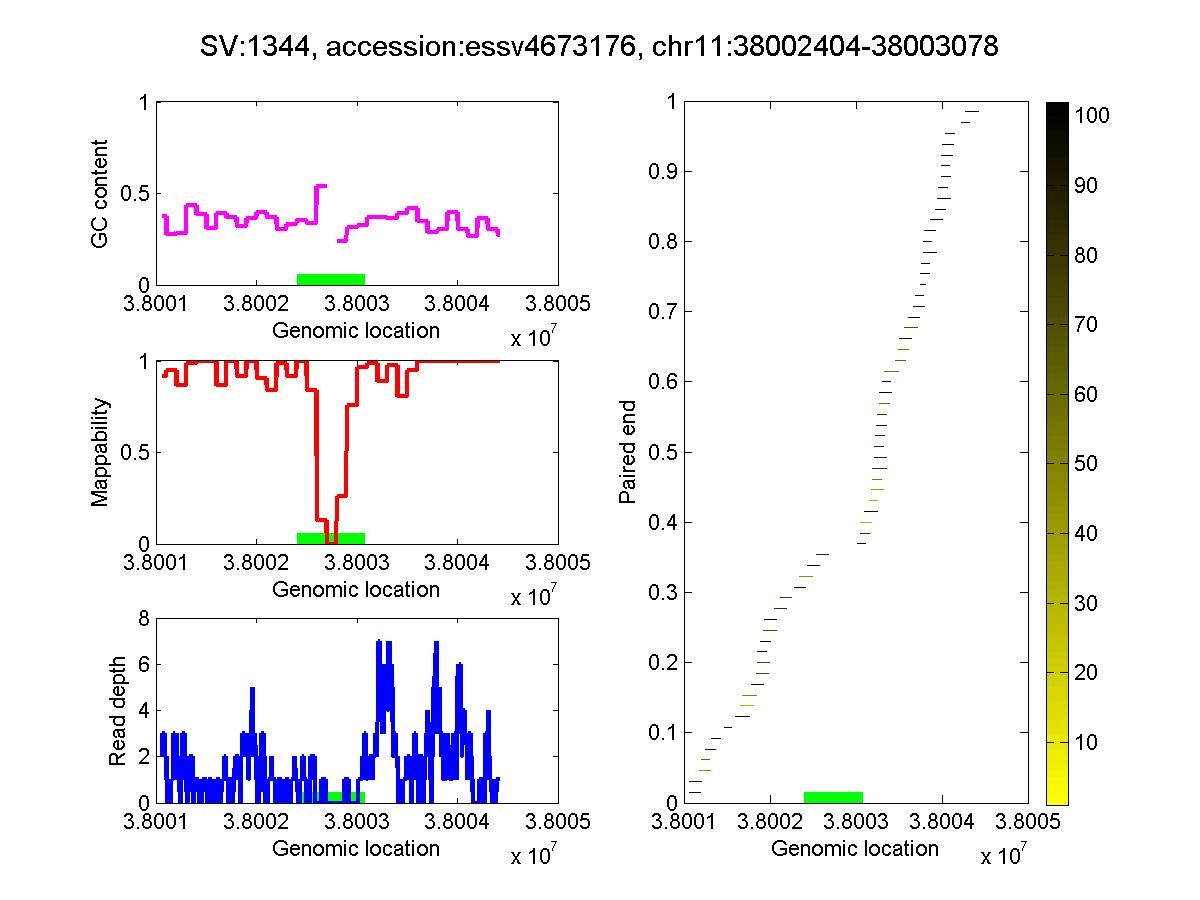

Supplement: Supplementary Materials — Supplementary data are available with this article at http://gr.xjtu.edu.cn/c/document_library/get_file?p_l_id=2403541&folderId=2539941&name=DLFE-115097.zip. Table S1 lists the complete information of suspicious variants and false positives, and the FIG directory contains the validation figures of each false positive. [file 8420547.f1.zip › 8420547.f1/FIG/SV1344.jpg]

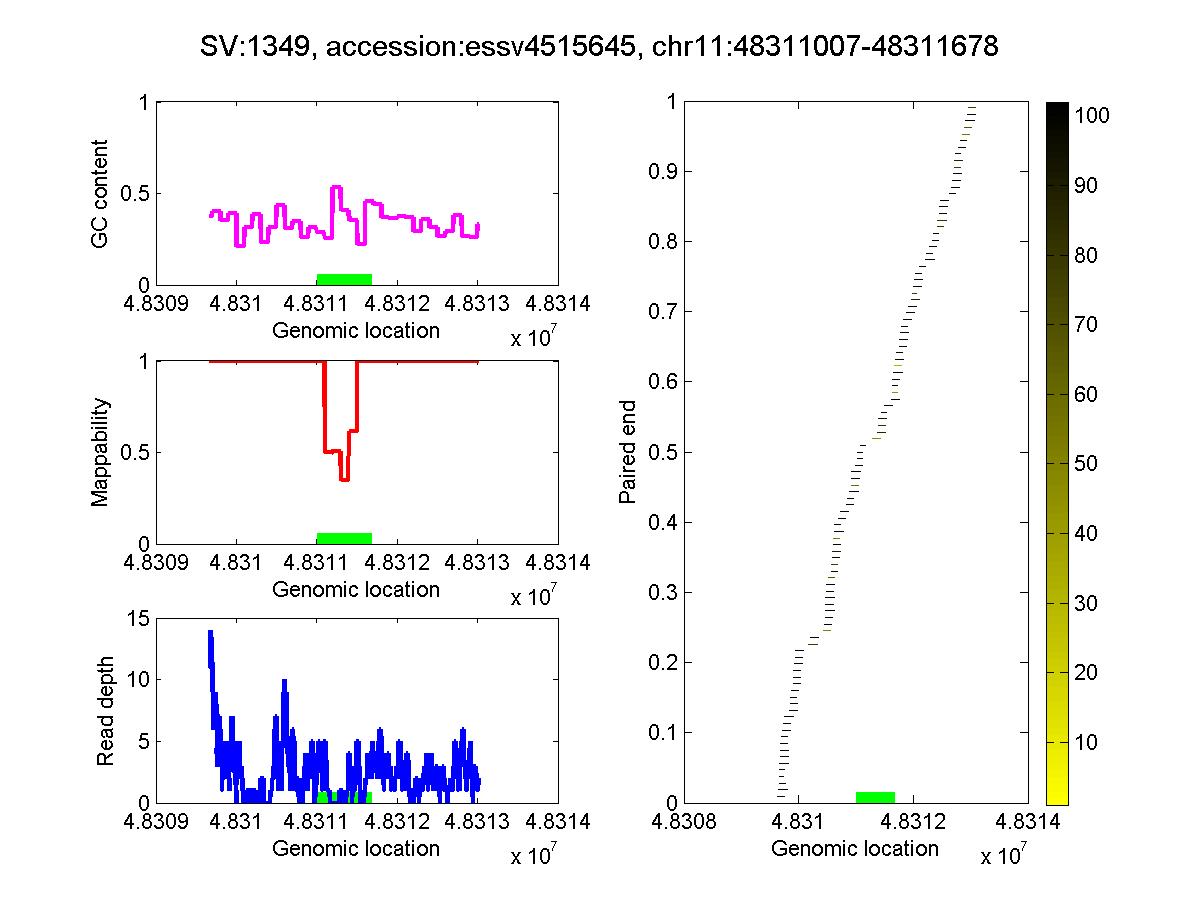

Supplement: Supplementary Materials — Supplementary data are available with this article at http://gr.xjtu.edu.cn/c/document_library/get_file?p_l_id=2403541&folderId=2539941&name=DLFE-115097.zip. Table S1 lists the complete information of suspicious variants and false positives, and the FIG directory contains the validation figures of each false positive. [file 8420547.f1.zip › 8420547.f1/FIG/SV1349.jpg]

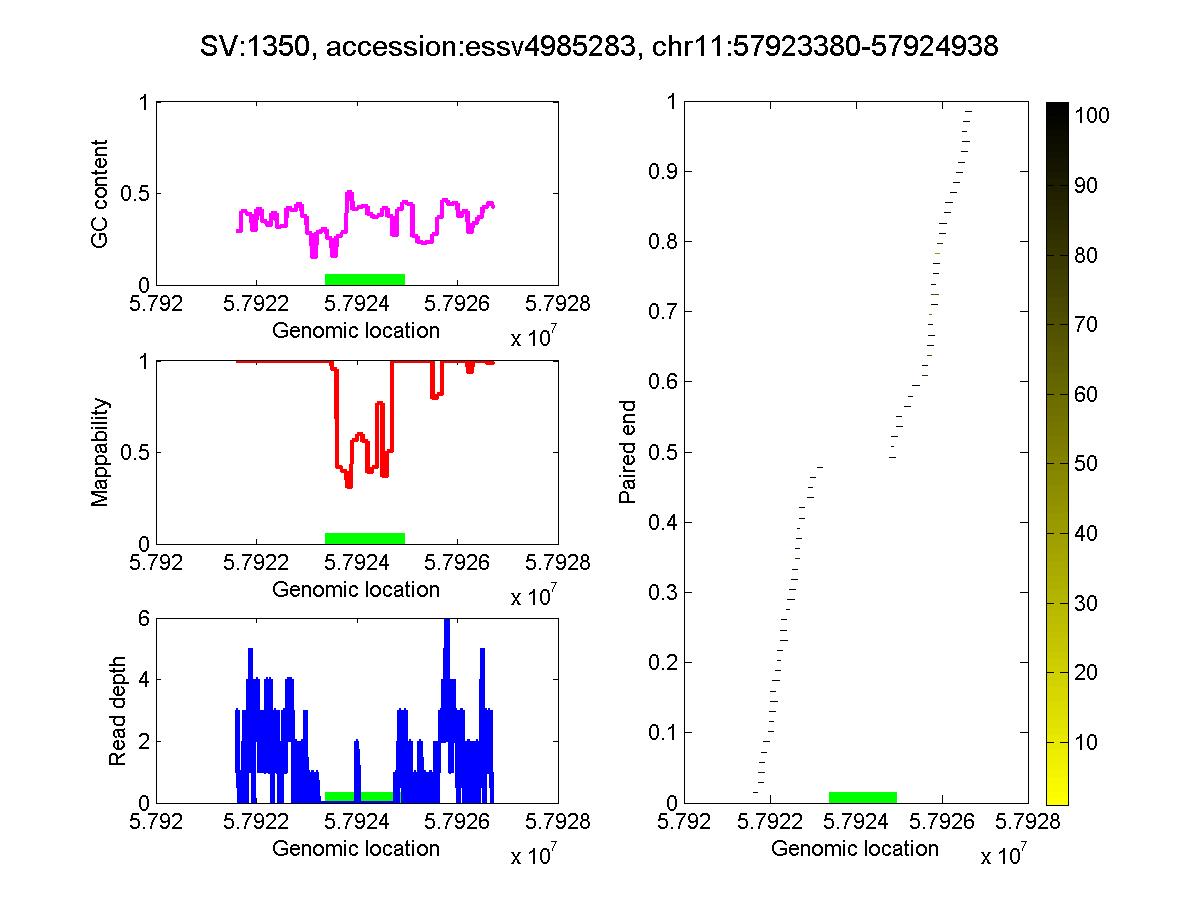

Supplement: Supplementary Materials — Supplementary data are available with this article at http://gr.xjtu.edu.cn/c/document_library/get_file?p_l_id=2403541&folderId=2539941&name=DLFE-115097.zip. Table S1 lists the complete information of suspicious variants and false positives, and the FIG directory contains the validation figures of each false positive. [file 8420547.f1.zip › 8420547.f1/FIG/SV1350.jpg]

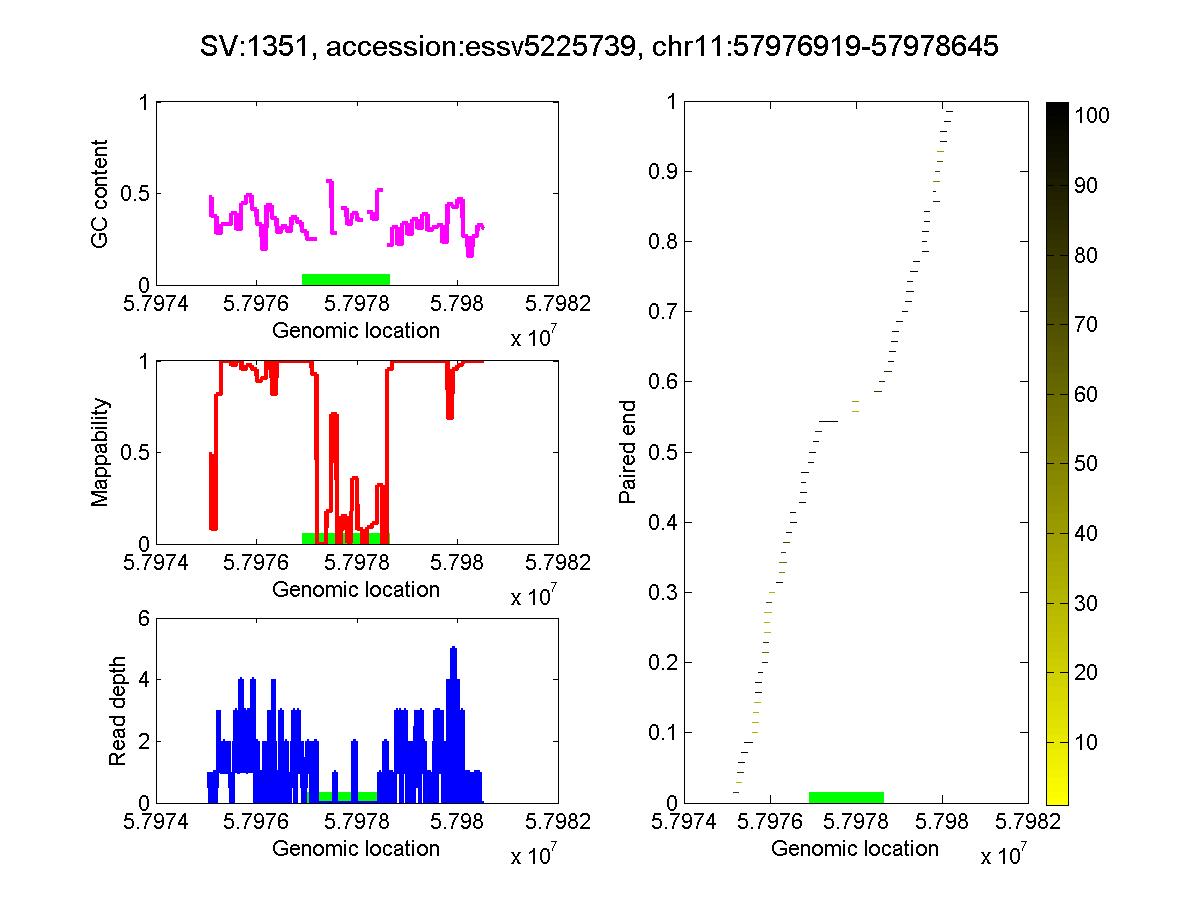

Supplement: Supplementary Materials — Supplementary data are available with this article at http://gr.xjtu.edu.cn/c/document_library/get_file?p_l_id=2403541&folderId=2539941&name=DLFE-115097.zip. Table S1 lists the complete information of suspicious variants and false positives, and the FIG directory contains the validation figures of each false positive. [file 8420547.f1.zip › 8420547.f1/FIG/SV1351.jpg]

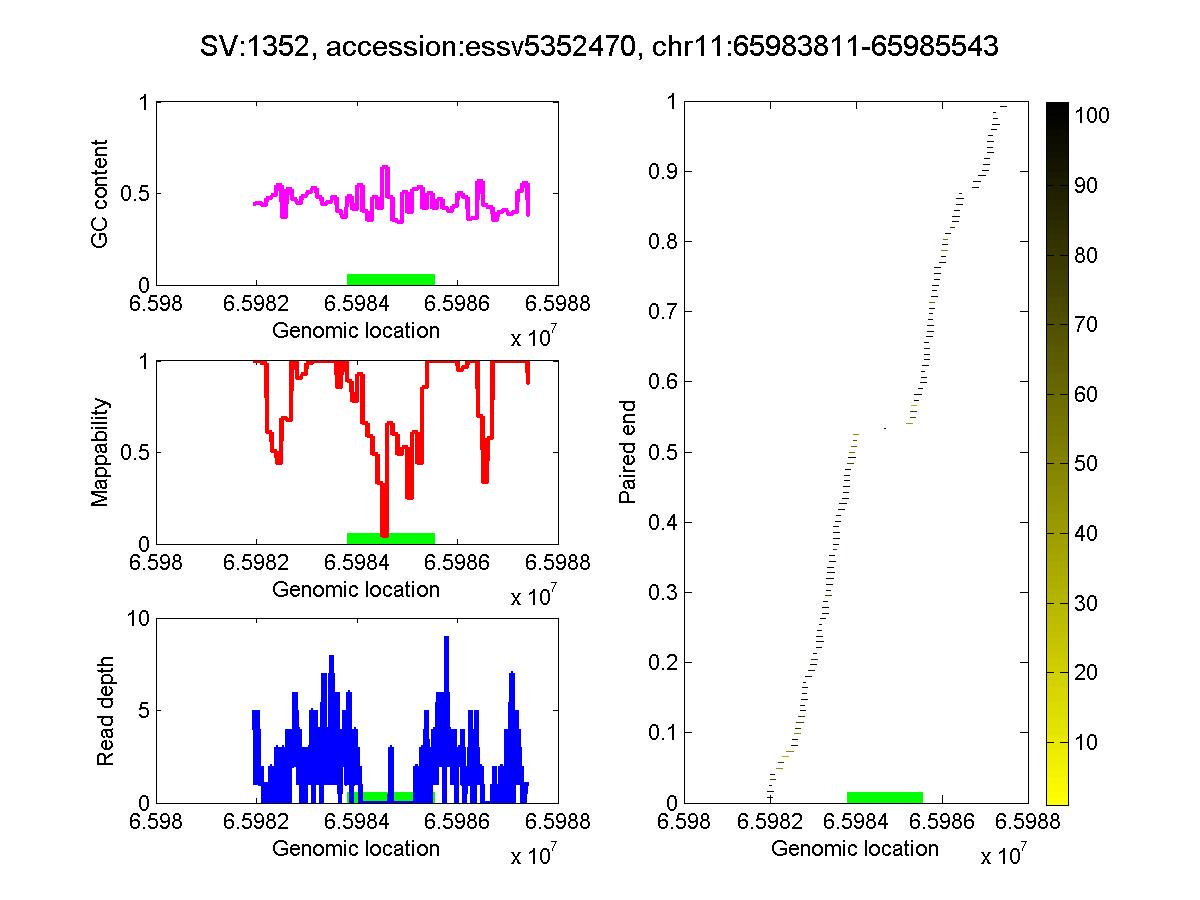

Supplement: Supplementary Materials — Supplementary data are available with this article at http://gr.xjtu.edu.cn/c/document_library/get_file?p_l_id=2403541&folderId=2539941&name=DLFE-115097.zip. Table S1 lists the complete information of suspicious variants and false positives, and the FIG directory contains the validation figures of each false positive. [file 8420547.f1.zip › 8420547.f1/FIG/SV1352.jpg]

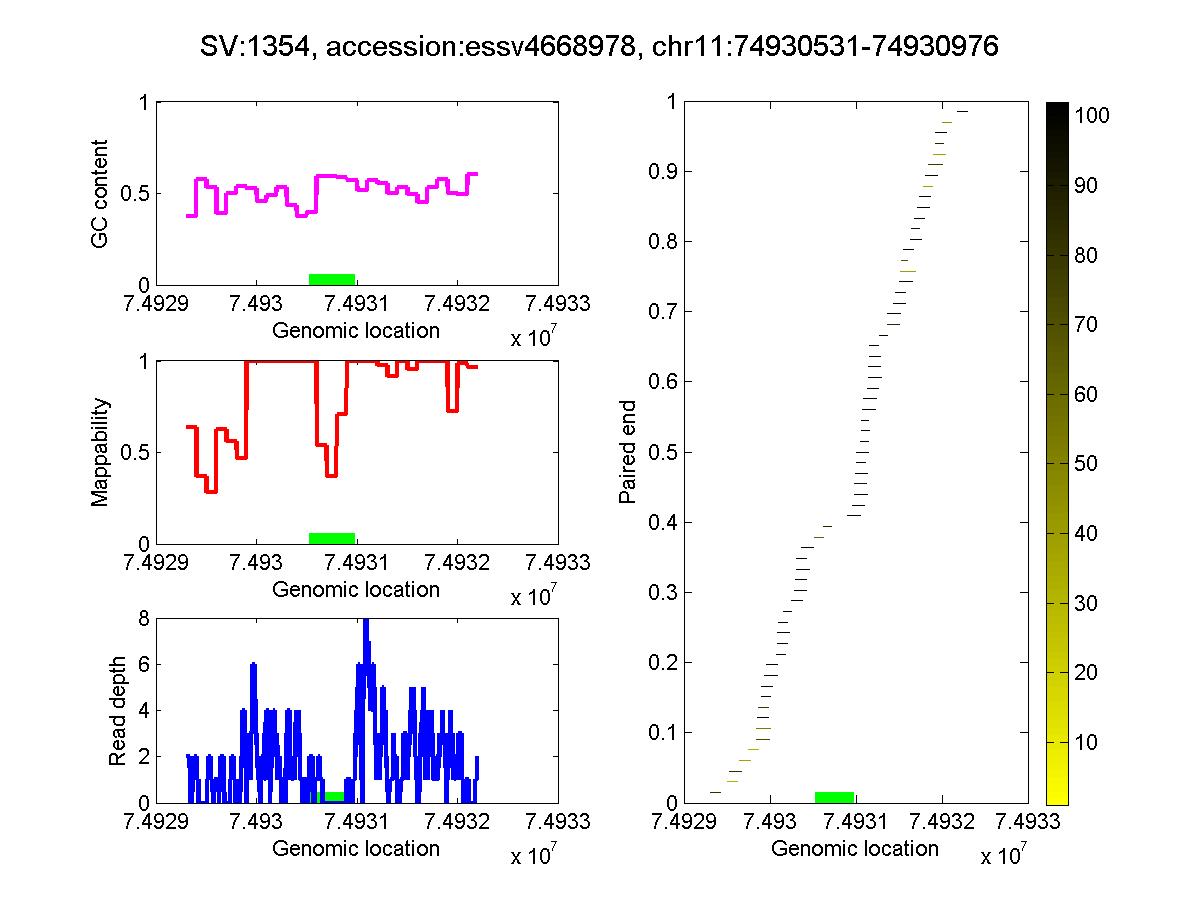

Supplement: Supplementary Materials — Supplementary data are available with this article at http://gr.xjtu.edu.cn/c/document_library/get_file?p_l_id=2403541&folderId=2539941&name=DLFE-115097.zip. Table S1 lists the complete information of suspicious variants and false positives, and the FIG directory contains the validation figures of each false positive. [file 8420547.f1.zip › 8420547.f1/FIG/SV1354.jpg]

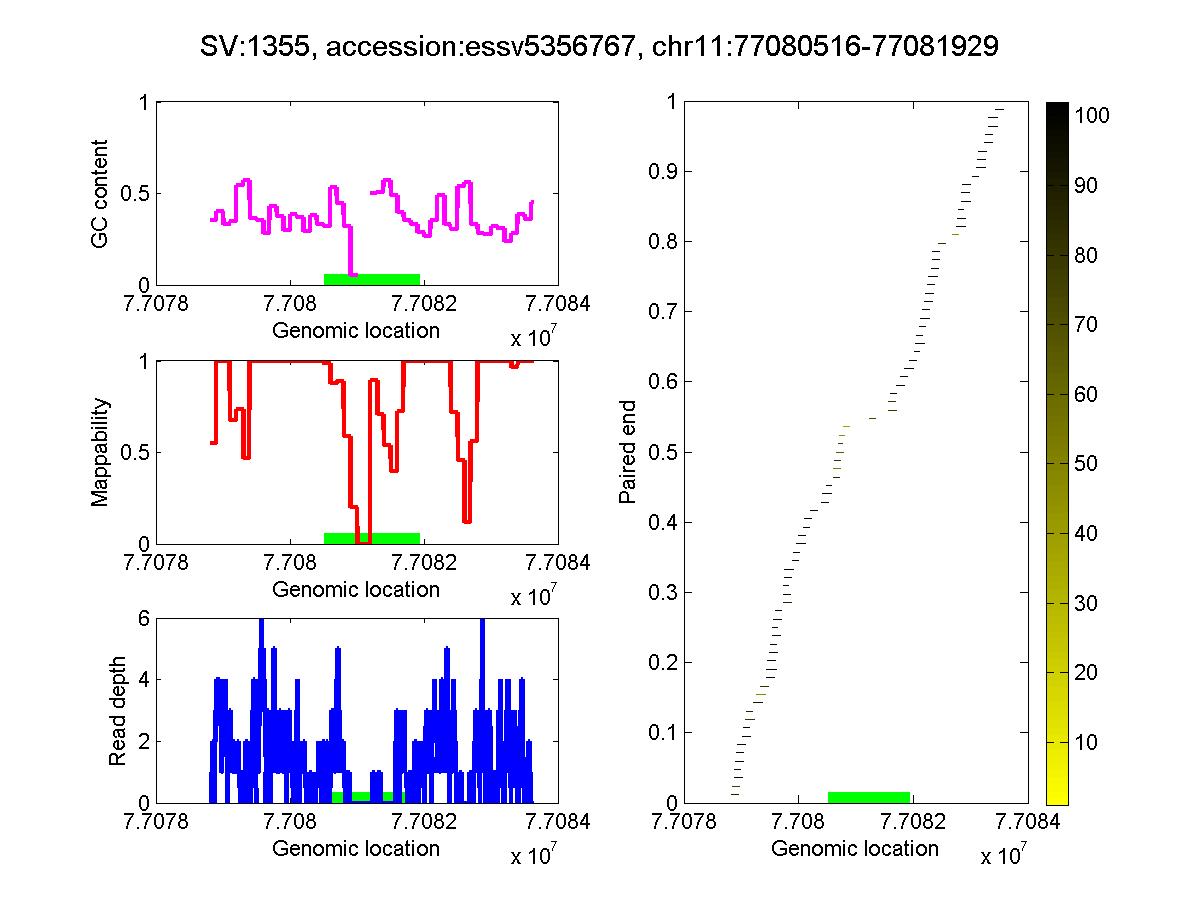

Supplement: Supplementary Materials — Supplementary data are available with this article at http://gr.xjtu.edu.cn/c/document_library/get_file?p_l_id=2403541&folderId=2539941&name=DLFE-115097.zip. Table S1 lists the complete information of suspicious variants and false positives, and the FIG directory contains the validation figures of each false positive. [file 8420547.f1.zip › 8420547.f1/FIG/SV1355.jpg]

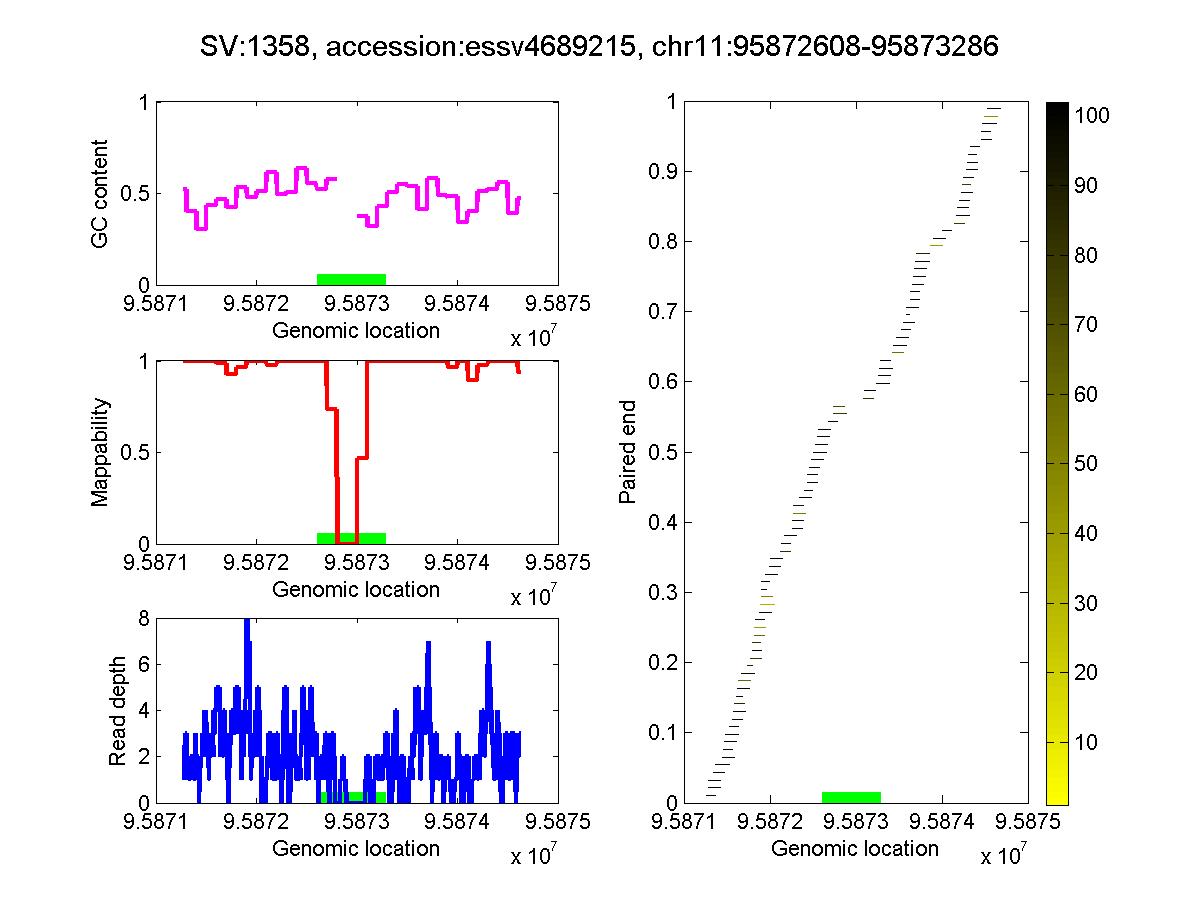

Supplement: Supplementary Materials — Supplementary data are available with this article at http://gr.xjtu.edu.cn/c/document_library/get_file?p_l_id=2403541&folderId=2539941&name=DLFE-115097.zip. Table S1 lists the complete information of suspicious variants and false positives, and the FIG directory contains the validation figures of each false positive. [file 8420547.f1.zip › 8420547.f1/FIG/SV1358.jpg]

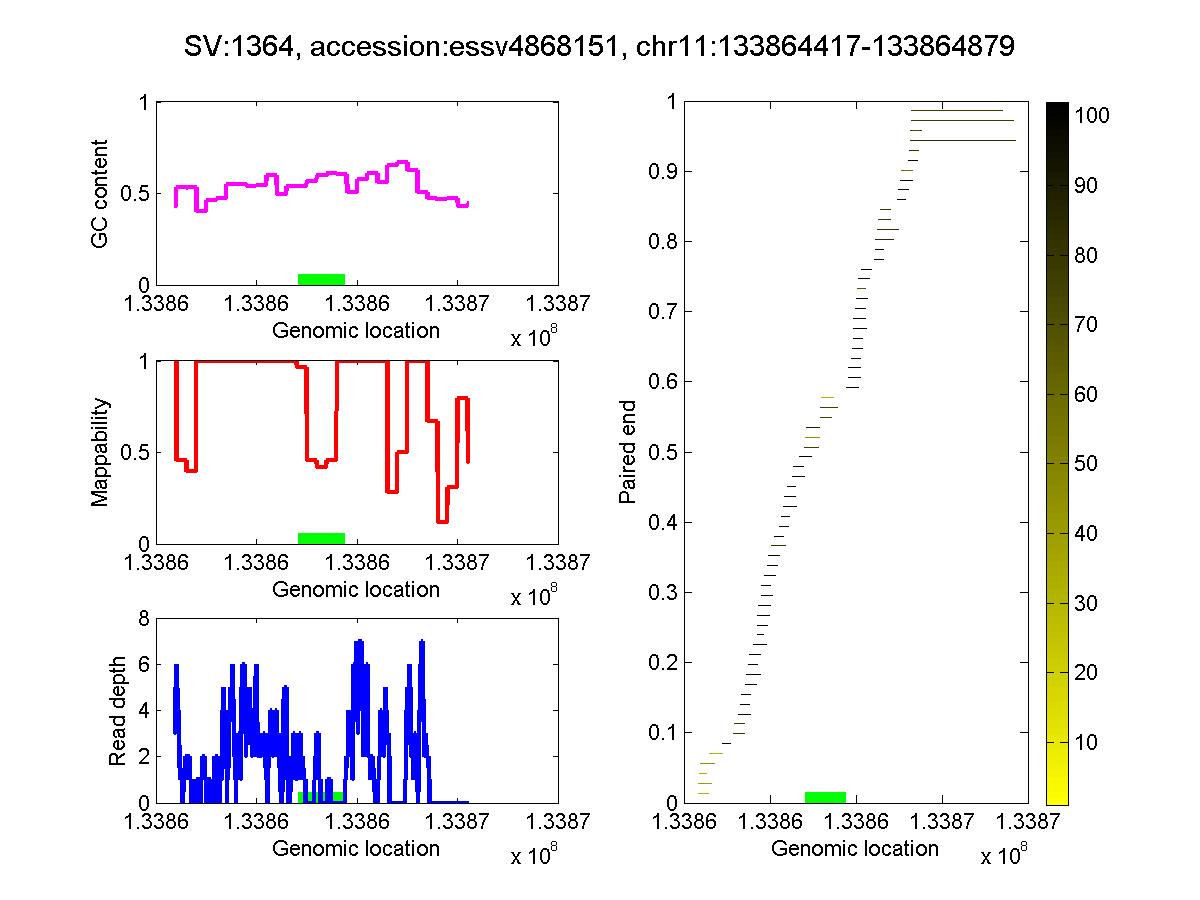

Supplement: Supplementary Materials — Supplementary data are available with this article at http://gr.xjtu.edu.cn/c/document_library/get_file?p_l_id=2403541&folderId=2539941&name=DLFE-115097.zip. Table S1 lists the complete information of suspicious variants and false positives, and the FIG directory contains the validation figures of each false positive. [file 8420547.f1.zip › 8420547.f1/FIG/SV1364.jpg]

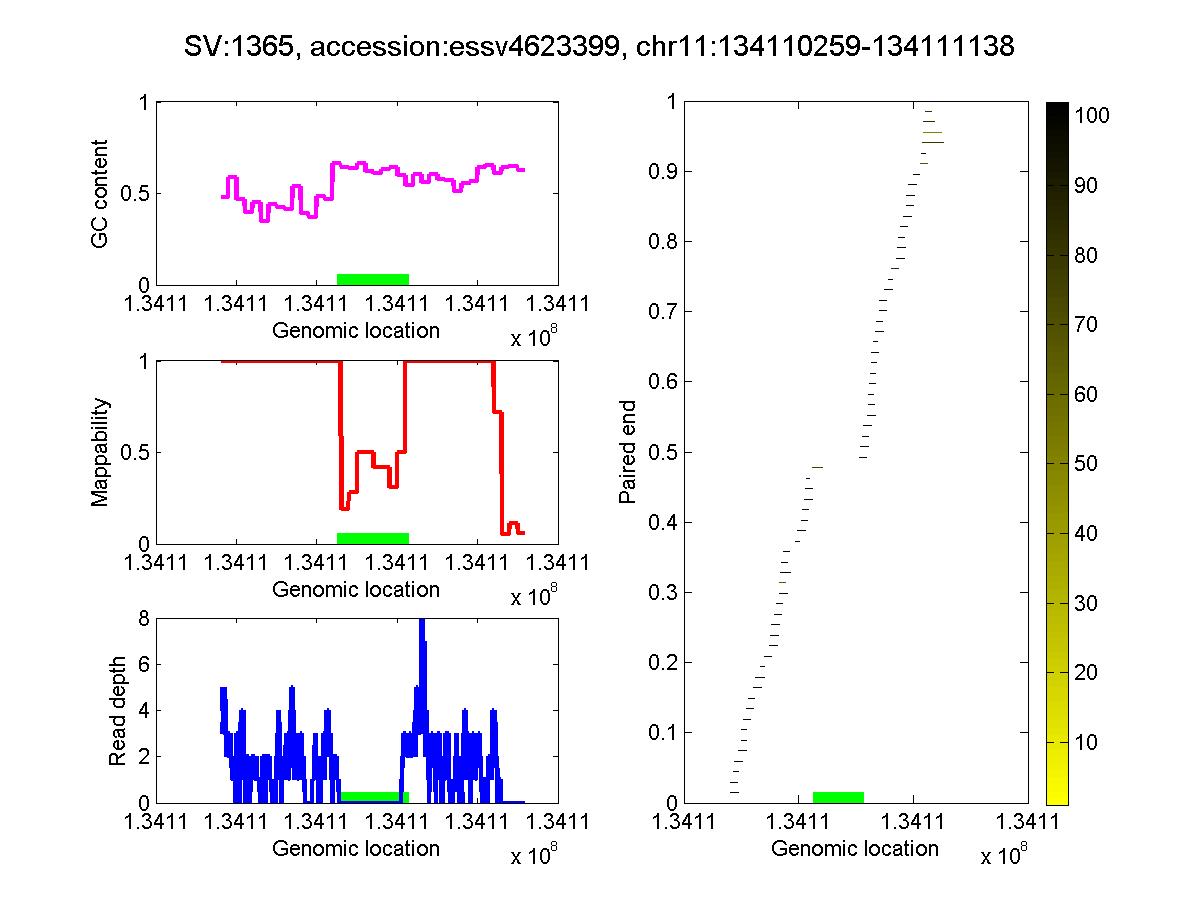

Supplement: Supplementary Materials — Supplementary data are available with this article at http://gr.xjtu.edu.cn/c/document_library/get_file?p_l_id=2403541&folderId=2539941&name=DLFE-115097.zip. Table S1 lists the complete information of suspicious variants and false positives, and the FIG directory contains the validation figures of each false positive. [file 8420547.f1.zip › 8420547.f1/FIG/SV1365.jpg]

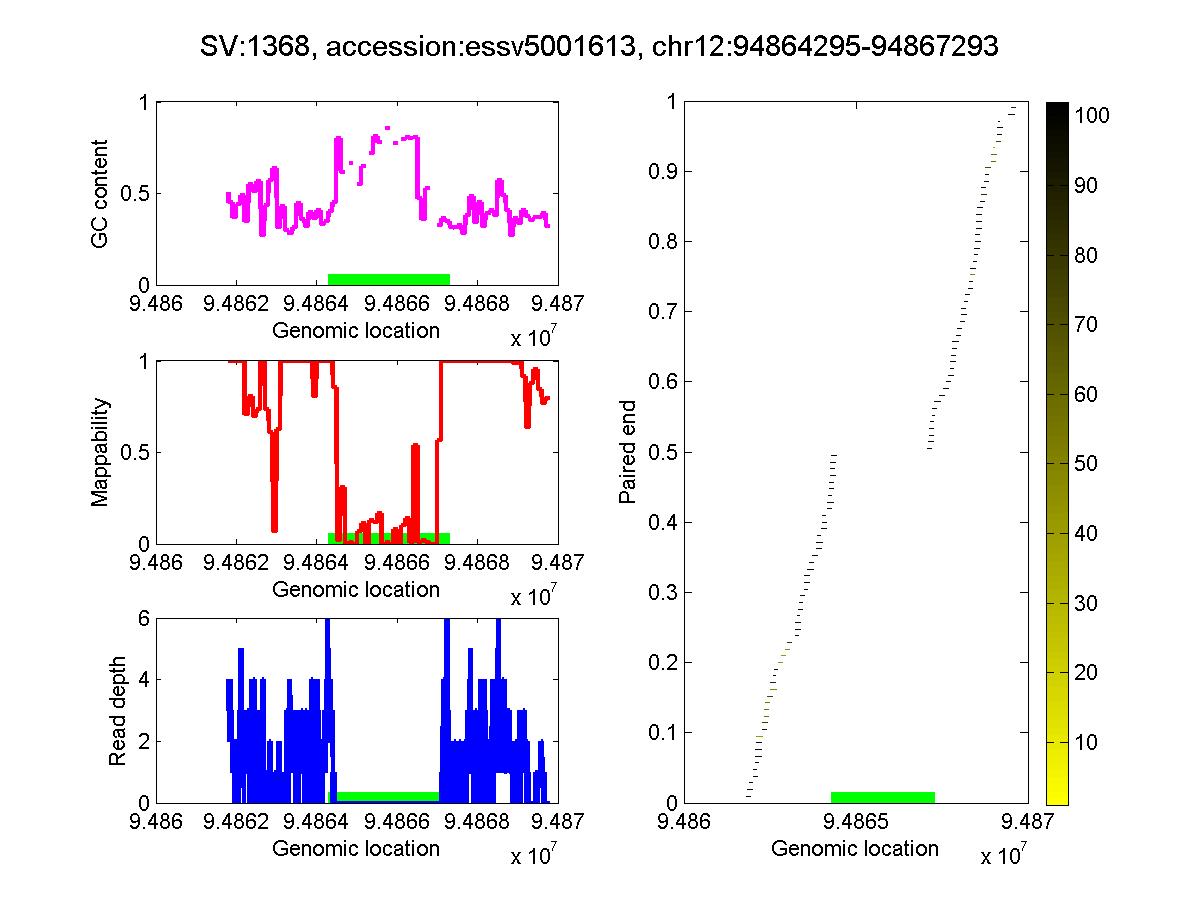

Supplement: Supplementary Materials — Supplementary data are available with this article at http://gr.xjtu.edu.cn/c/document_library/get_file?p_l_id=2403541&folderId=2539941&name=DLFE-115097.zip. Table S1 lists the complete information of suspicious variants and false positives, and the FIG directory contains the validation figures of each false positive. [file 8420547.f1.zip › 8420547.f1/FIG/SV1368.jpg]

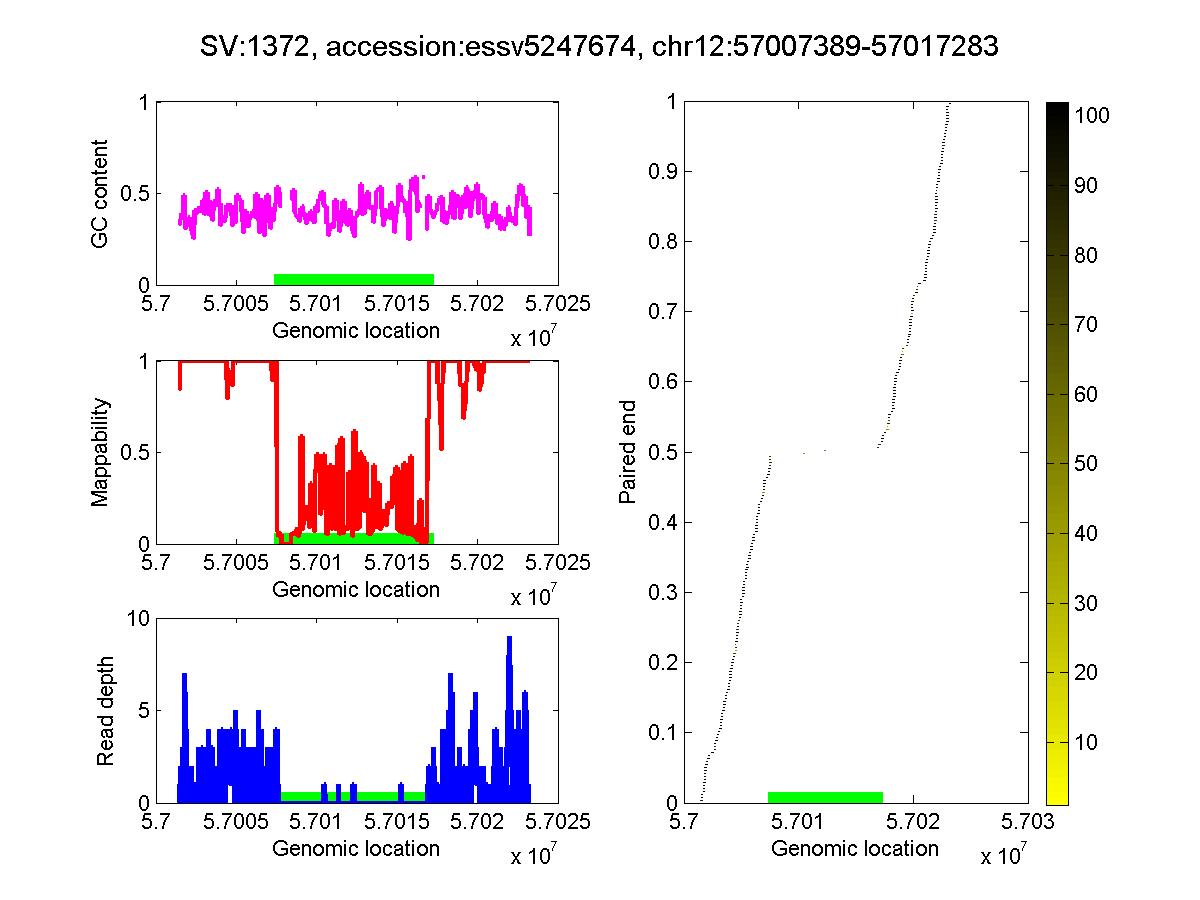

Supplement: Supplementary Materials — Supplementary data are available with this article at http://gr.xjtu.edu.cn/c/document_library/get_file?p_l_id=2403541&folderId=2539941&name=DLFE-115097.zip. Table S1 lists the complete information of suspicious variants and false positives, and the FIG directory contains the validation figures of each false positive. [file 8420547.f1.zip › 8420547.f1/FIG/SV1372.jpg]

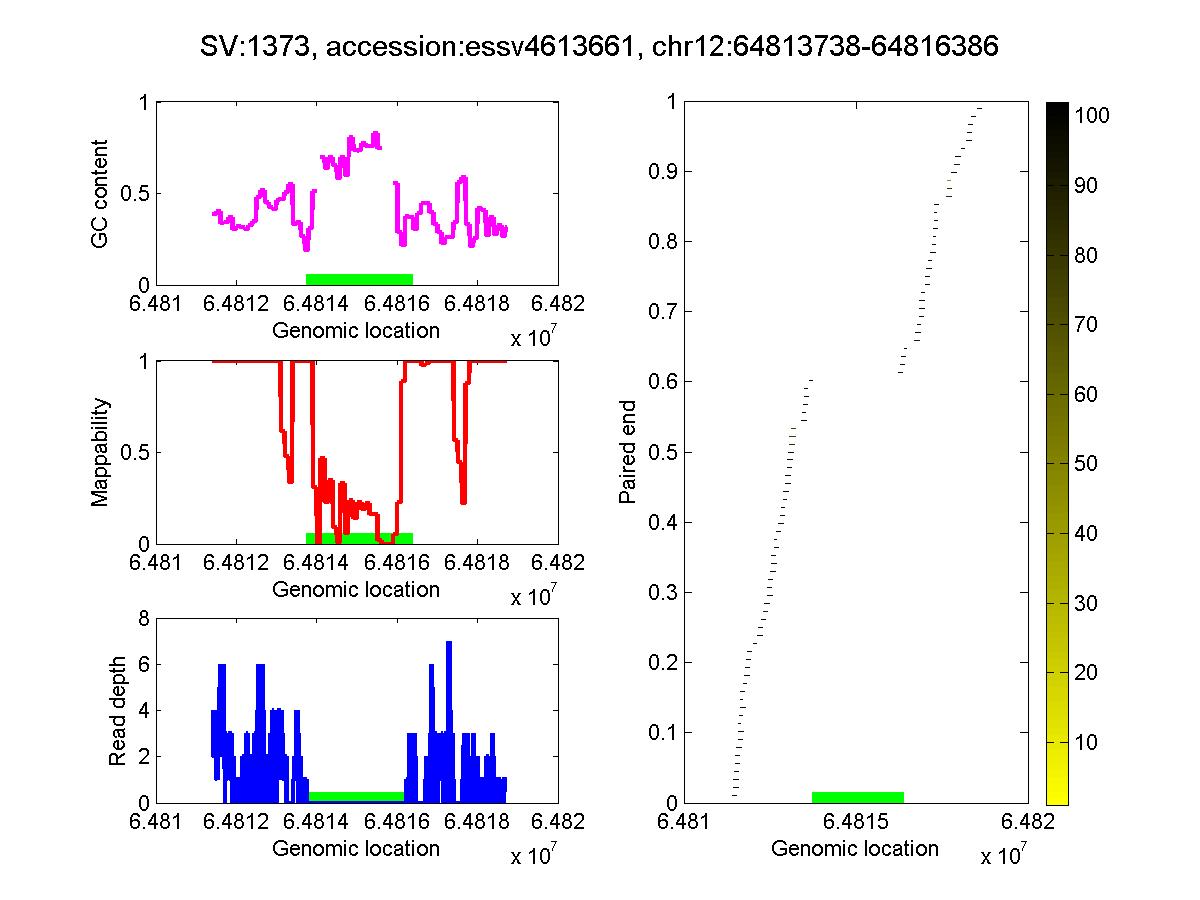

Supplement: Supplementary Materials — Supplementary data are available with this article at http://gr.xjtu.edu.cn/c/document_library/get_file?p_l_id=2403541&folderId=2539941&name=DLFE-115097.zip. Table S1 lists the complete information of suspicious variants and false positives, and the FIG directory contains the validation figures of each false positive. [file 8420547.f1.zip › 8420547.f1/FIG/SV1373.jpg]

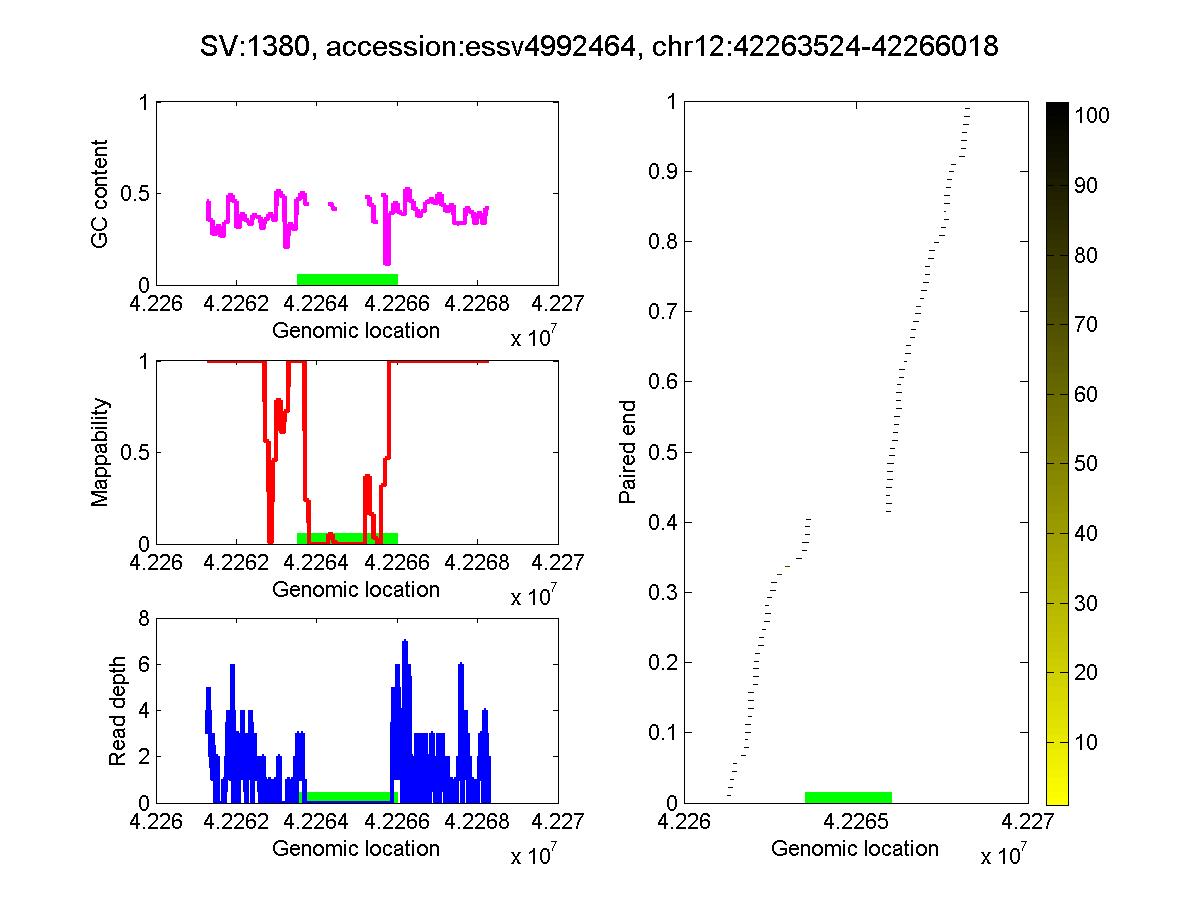

Supplement: Supplementary Materials — Supplementary data are available with this article at http://gr.xjtu.edu.cn/c/document_library/get_file?p_l_id=2403541&folderId=2539941&name=DLFE-115097.zip. Table S1 lists the complete information of suspicious variants and false positives, and the FIG directory contains the validation figures of each false positive. [file 8420547.f1.zip › 8420547.f1/FIG/SV1380.jpg]

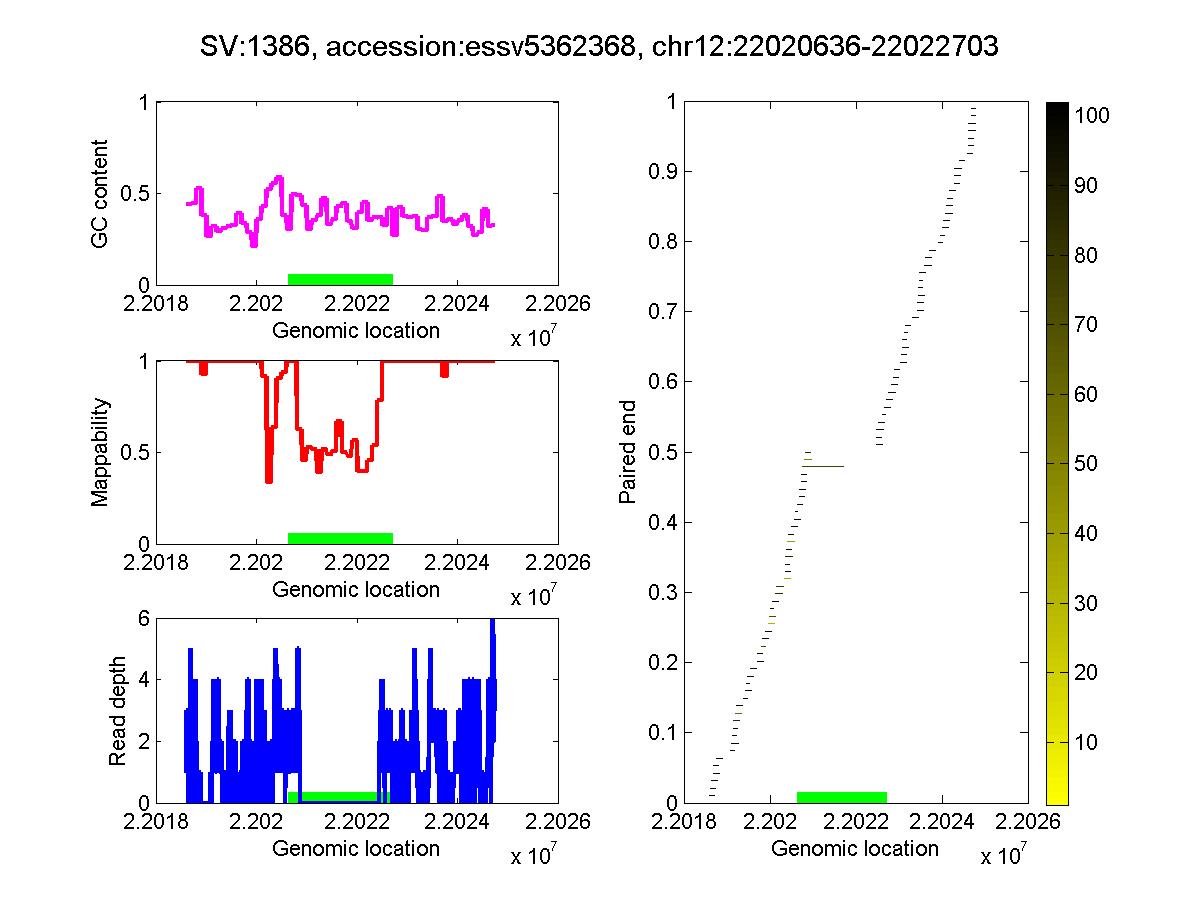

Supplement: Supplementary Materials — Supplementary data are available with this article at http://gr.xjtu.edu.cn/c/document_library/get_file?p_l_id=2403541&folderId=2539941&name=DLFE-115097.zip. Table S1 lists the complete information of suspicious variants and false positives, and the FIG directory contains the validation figures of each false positive. [file 8420547.f1.zip › 8420547.f1/FIG/SV1386.jpg]

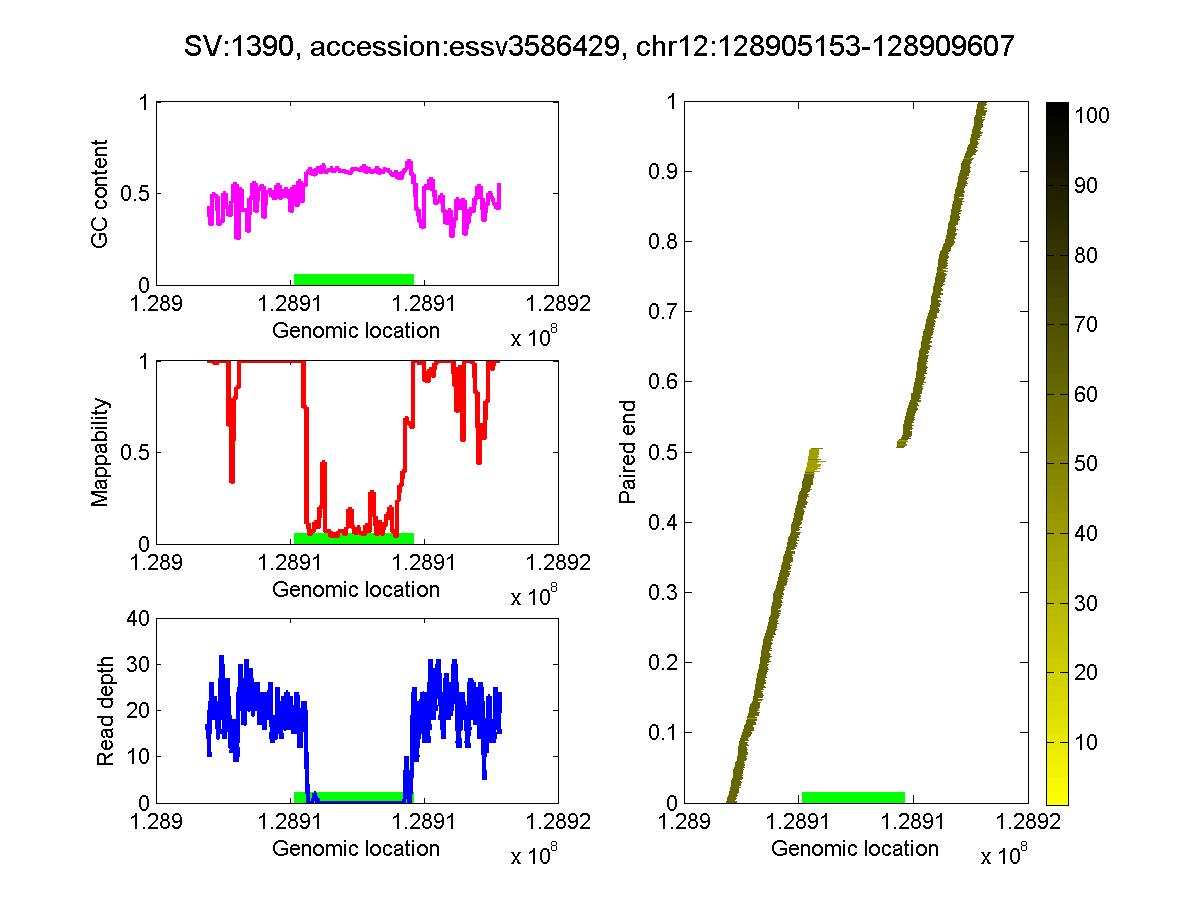

Supplement: Supplementary Materials — Supplementary data are available with this article at http://gr.xjtu.edu.cn/c/document_library/get_file?p_l_id=2403541&folderId=2539941&name=DLFE-115097.zip. Table S1 lists the complete information of suspicious variants and false positives, and the FIG directory contains the validation figures of each false positive. [file 8420547.f1.zip › 8420547.f1/FIG/SV1390.jpg]

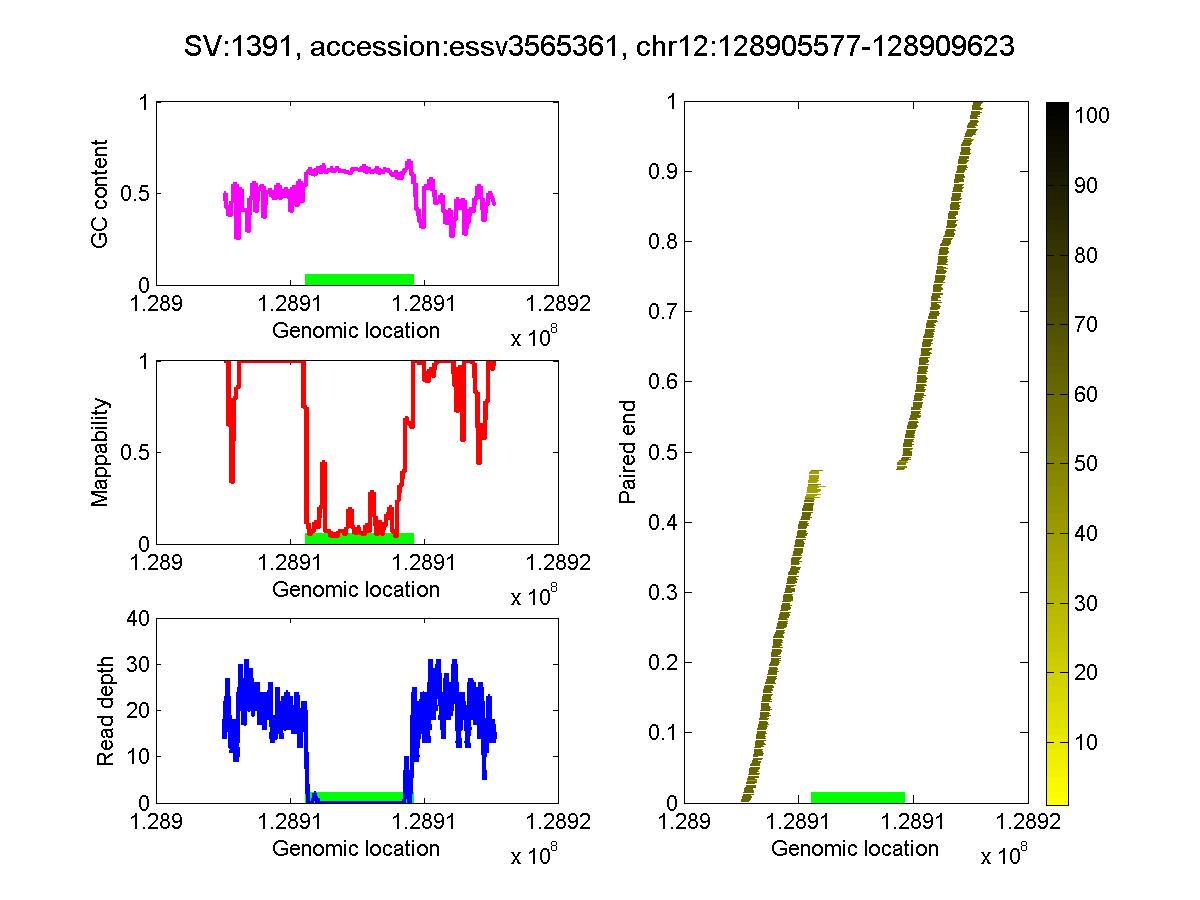

Supplement: Supplementary Materials — Supplementary data are available with this article at http://gr.xjtu.edu.cn/c/document_library/get_file?p_l_id=2403541&folderId=2539941&name=DLFE-115097.zip. Table S1 lists the complete information of suspicious variants and false positives, and the FIG directory contains the validation figures of each false positive. [file 8420547.f1.zip › 8420547.f1/FIG/SV1391.jpg]

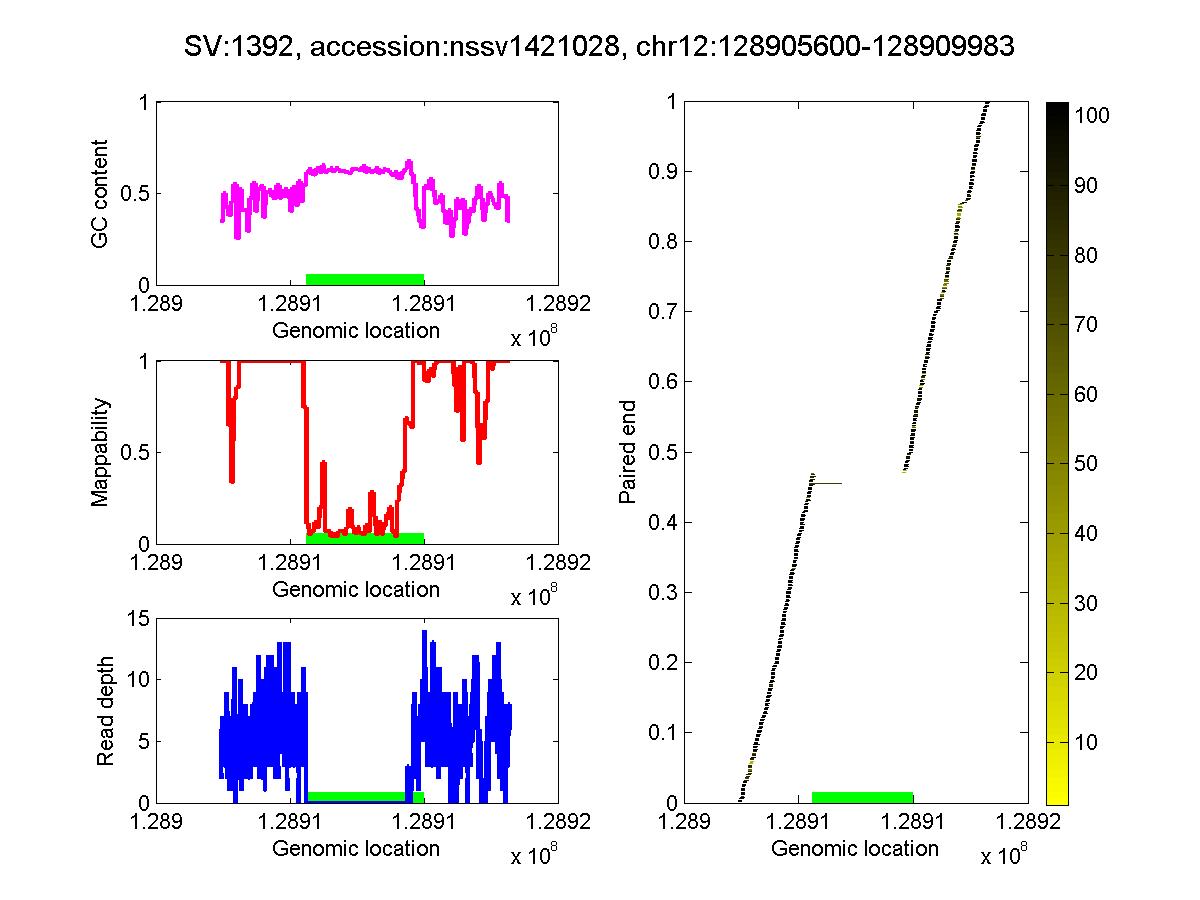

Supplement: Supplementary Materials — Supplementary data are available with this article at http://gr.xjtu.edu.cn/c/document_library/get_file?p_l_id=2403541&folderId=2539941&name=DLFE-115097.zip. Table S1 lists the complete information of suspicious variants and false positives, and the FIG directory contains the validation figures of each false positive. [file 8420547.f1.zip › 8420547.f1/FIG/SV1392.jpg]

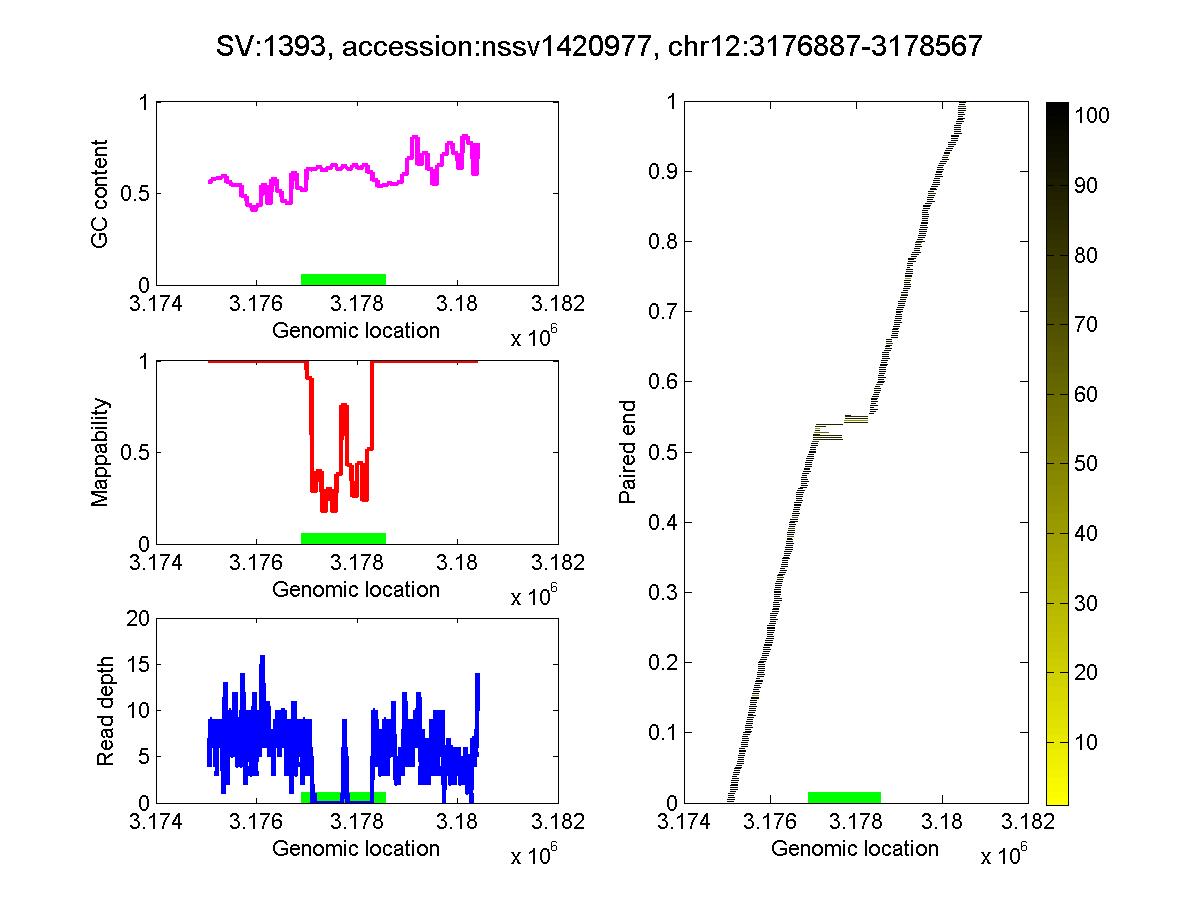

Supplement: Supplementary Materials — Supplementary data are available with this article at http://gr.xjtu.edu.cn/c/document_library/get_file?p_l_id=2403541&folderId=2539941&name=DLFE-115097.zip. Table S1 lists the complete information of suspicious variants and false positives, and the FIG directory contains the validation figures of each false positive. [file 8420547.f1.zip › 8420547.f1/FIG/SV1393.jpg]

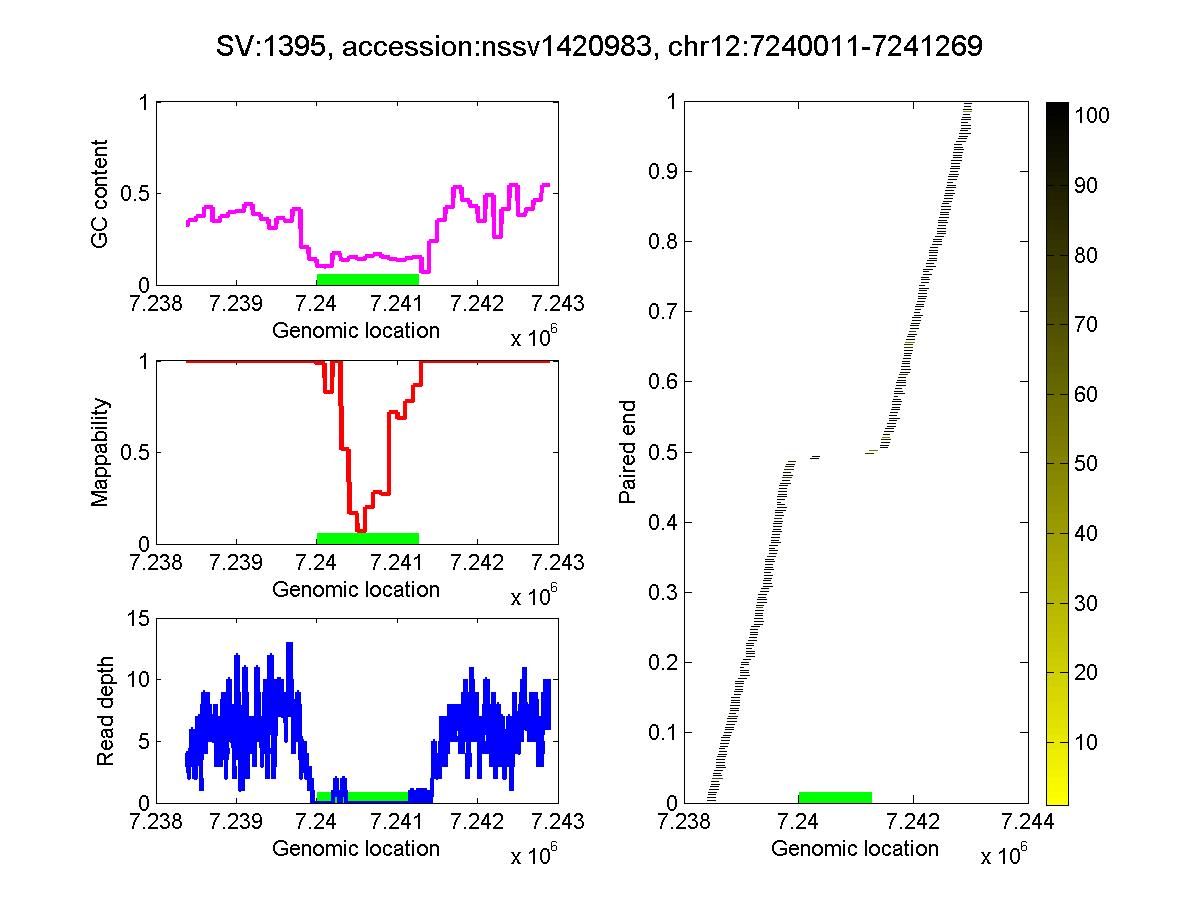

Supplement: Supplementary Materials — Supplementary data are available with this article at http://gr.xjtu.edu.cn/c/document_library/get_file?p_l_id=2403541&folderId=2539941&name=DLFE-115097.zip. Table S1 lists the complete information of suspicious variants and false positives, and the FIG directory contains the validation figures of each false positive. [file 8420547.f1.zip › 8420547.f1/FIG/SV1395.jpg]

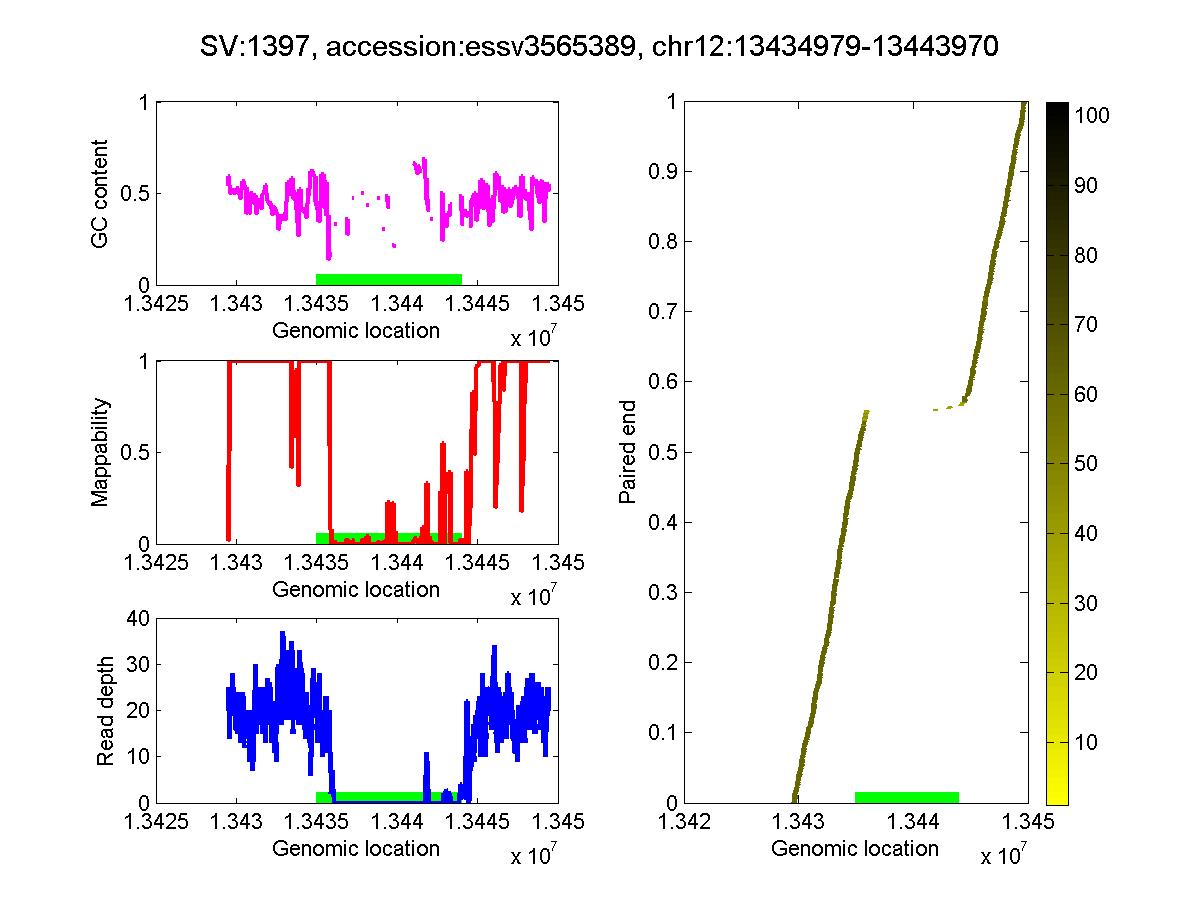

Supplement: Supplementary Materials — Supplementary data are available with this article at http://gr.xjtu.edu.cn/c/document_library/get_file?p_l_id=2403541&folderId=2539941&name=DLFE-115097.zip. Table S1 lists the complete information of suspicious variants and false positives, and the FIG directory contains the validation figures of each false positive. [file 8420547.f1.zip › 8420547.f1/FIG/SV1397.jpg]

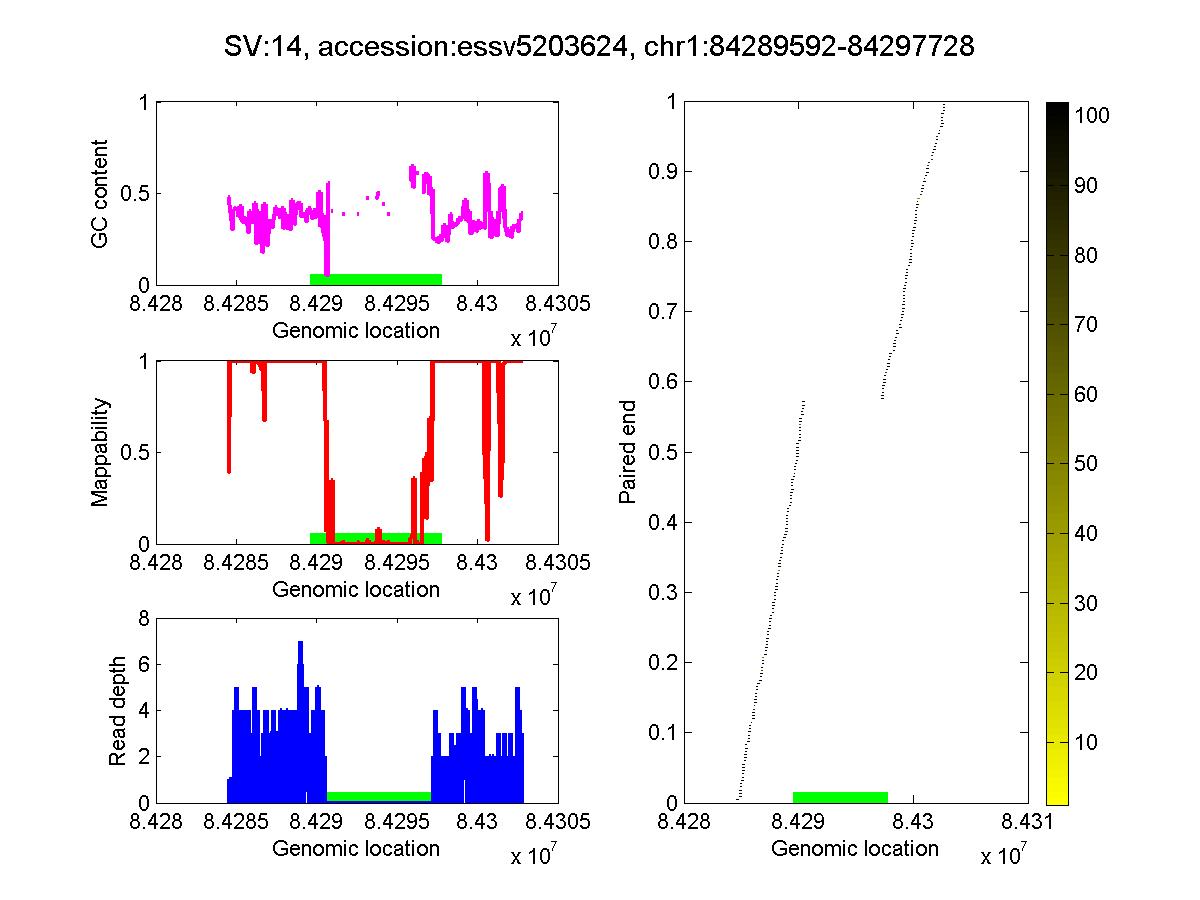

Supplement: Supplementary Materials — Supplementary data are available with this article at http://gr.xjtu.edu.cn/c/document_library/get_file?p_l_id=2403541&folderId=2539941&name=DLFE-115097.zip. Table S1 lists the complete information of suspicious variants and false positives, and the FIG directory contains the validation figures of each false positive. [file 8420547.f1.zip › 8420547.f1/FIG/SV14.jpg]

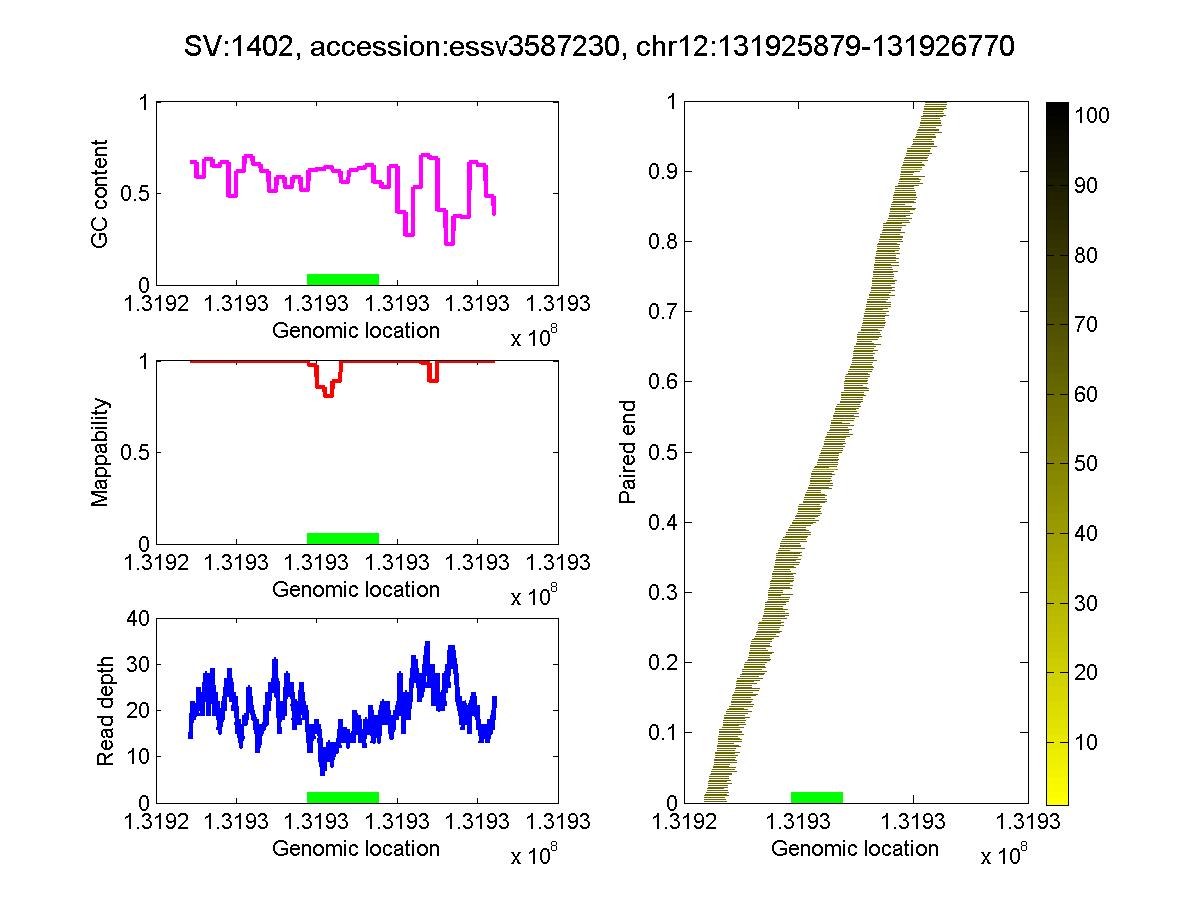

Supplement: Supplementary Materials — Supplementary data are available with this article at http://gr.xjtu.edu.cn/c/document_library/get_file?p_l_id=2403541&folderId=2539941&name=DLFE-115097.zip. Table S1 lists the complete information of suspicious variants and false positives, and the FIG directory contains the validation figures of each false positive. [file 8420547.f1.zip › 8420547.f1/FIG/SV1402.jpg]

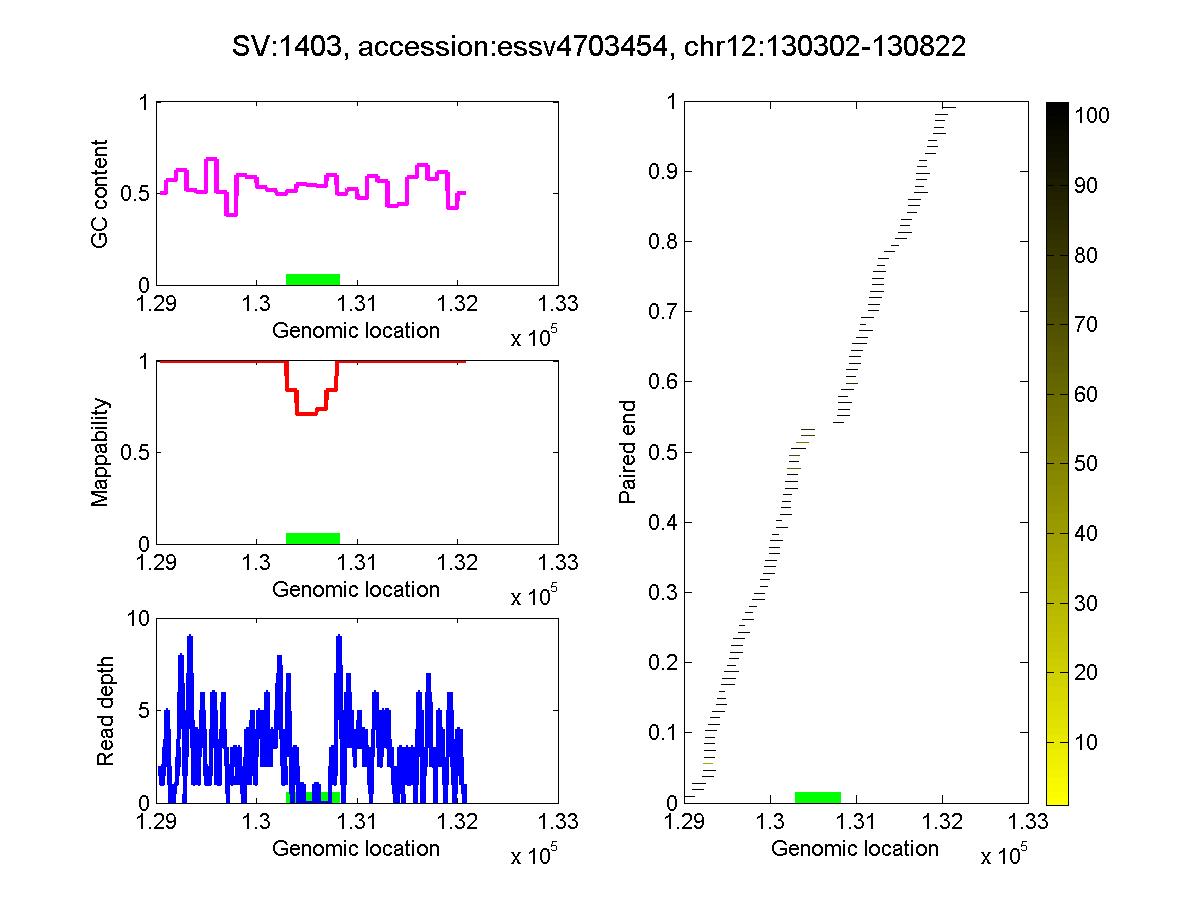

Supplement: Supplementary Materials — Supplementary data are available with this article at http://gr.xjtu.edu.cn/c/document_library/get_file?p_l_id=2403541&folderId=2539941&name=DLFE-115097.zip. Table S1 lists the complete information of suspicious variants and false positives, and the FIG directory contains the validation figures of each false positive. [file 8420547.f1.zip › 8420547.f1/FIG/SV1403.jpg]

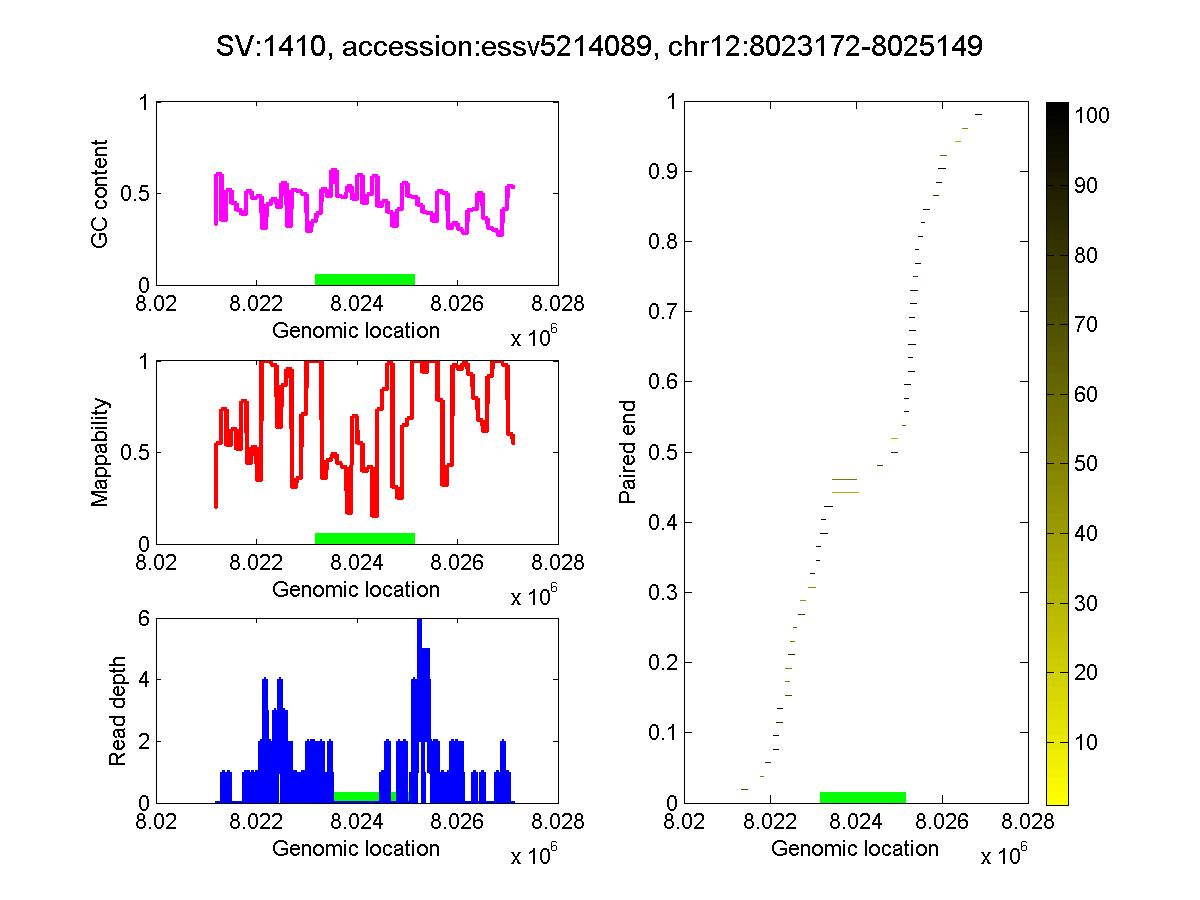

Supplement: Supplementary Materials — Supplementary data are available with this article at http://gr.xjtu.edu.cn/c/document_library/get_file?p_l_id=2403541&folderId=2539941&name=DLFE-115097.zip. Table S1 lists the complete information of suspicious variants and false positives, and the FIG directory contains the validation figures of each false positive. [file 8420547.f1.zip › 8420547.f1/FIG/SV1410.jpg]

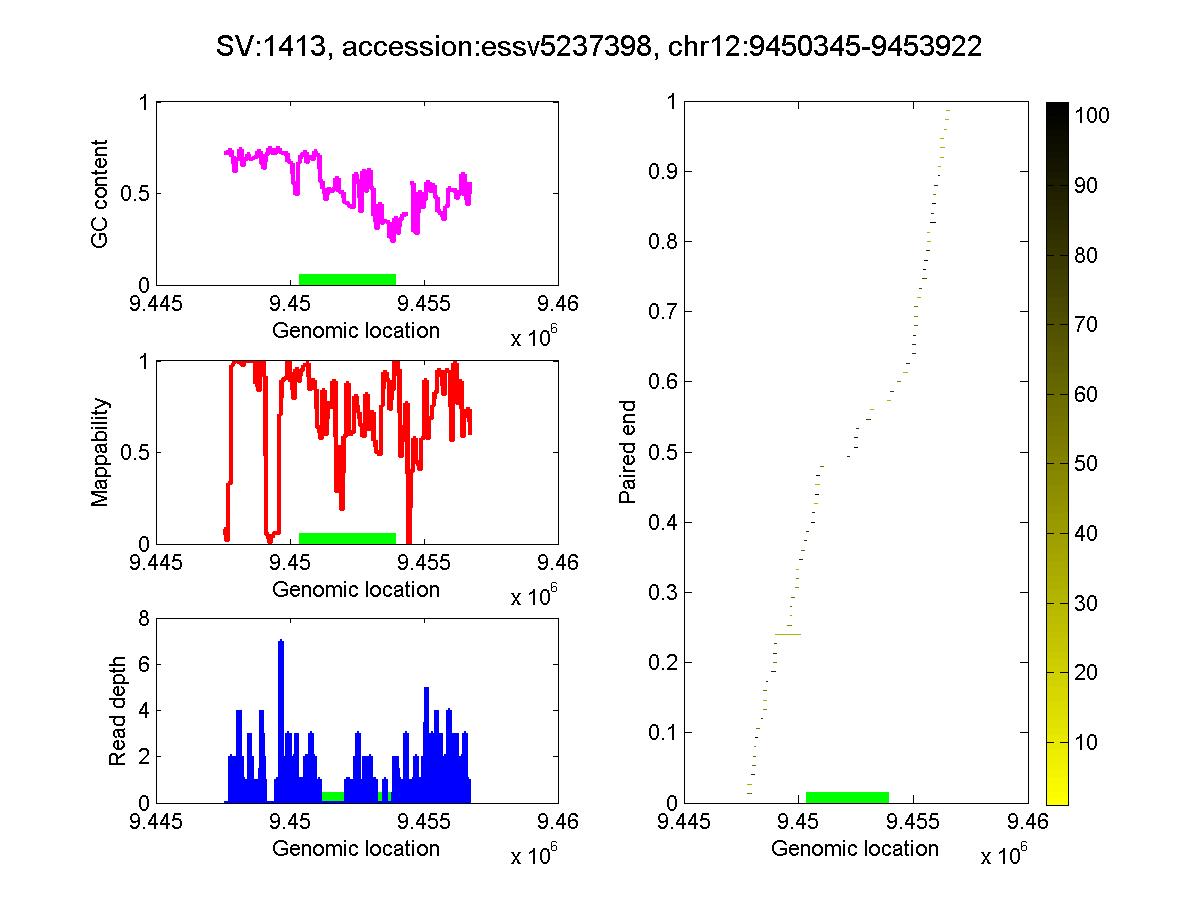

Supplement: Supplementary Materials — Supplementary data are available with this article at http://gr.xjtu.edu.cn/c/document_library/get_file?p_l_id=2403541&folderId=2539941&name=DLFE-115097.zip. Table S1 lists the complete information of suspicious variants and false positives, and the FIG directory contains the validation figures of each false positive. [file 8420547.f1.zip › 8420547.f1/FIG/SV1413.jpg]

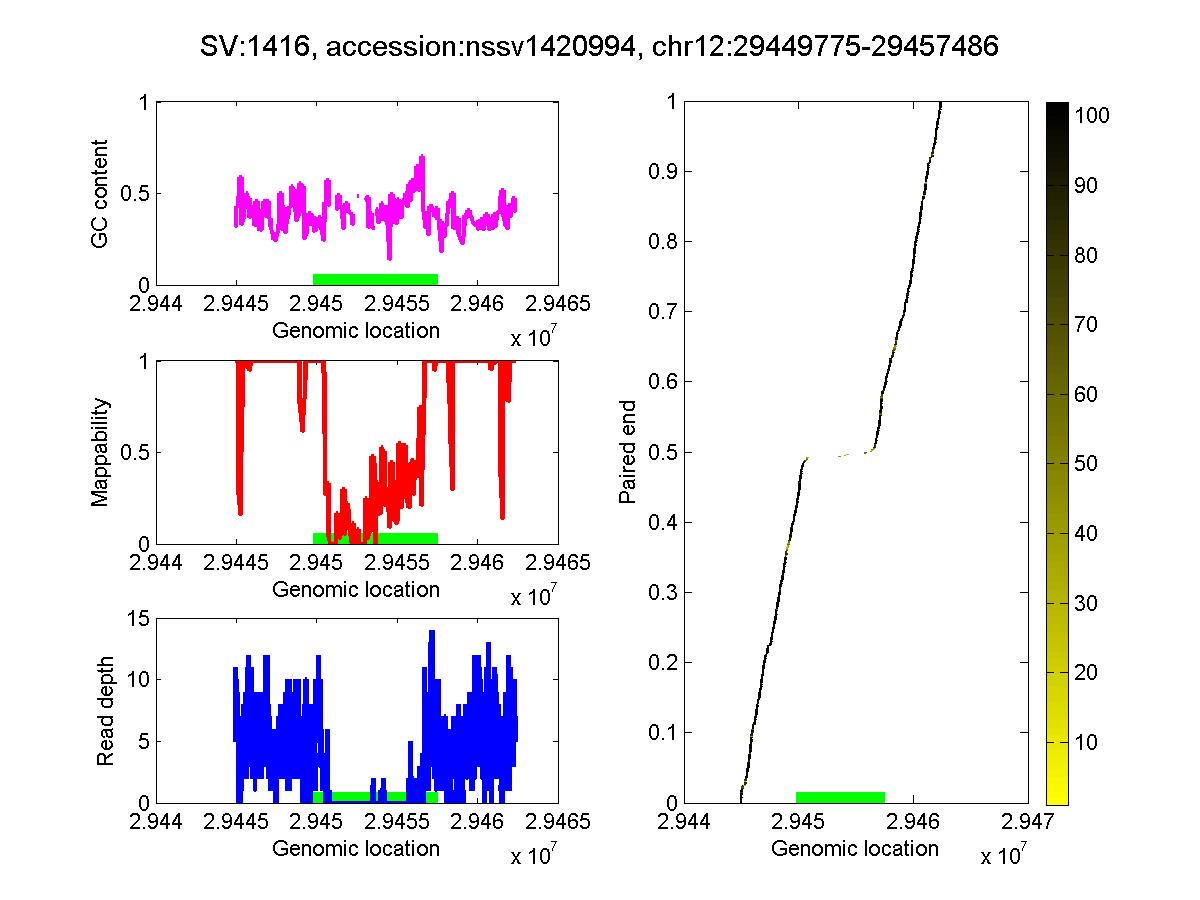

Supplement: Supplementary Materials — Supplementary data are available with this article at http://gr.xjtu.edu.cn/c/document_library/get_file?p_l_id=2403541&folderId=2539941&name=DLFE-115097.zip. Table S1 lists the complete information of suspicious variants and false positives, and the FIG directory contains the validation figures of each false positive. [file 8420547.f1.zip › 8420547.f1/FIG/SV1416.jpg]

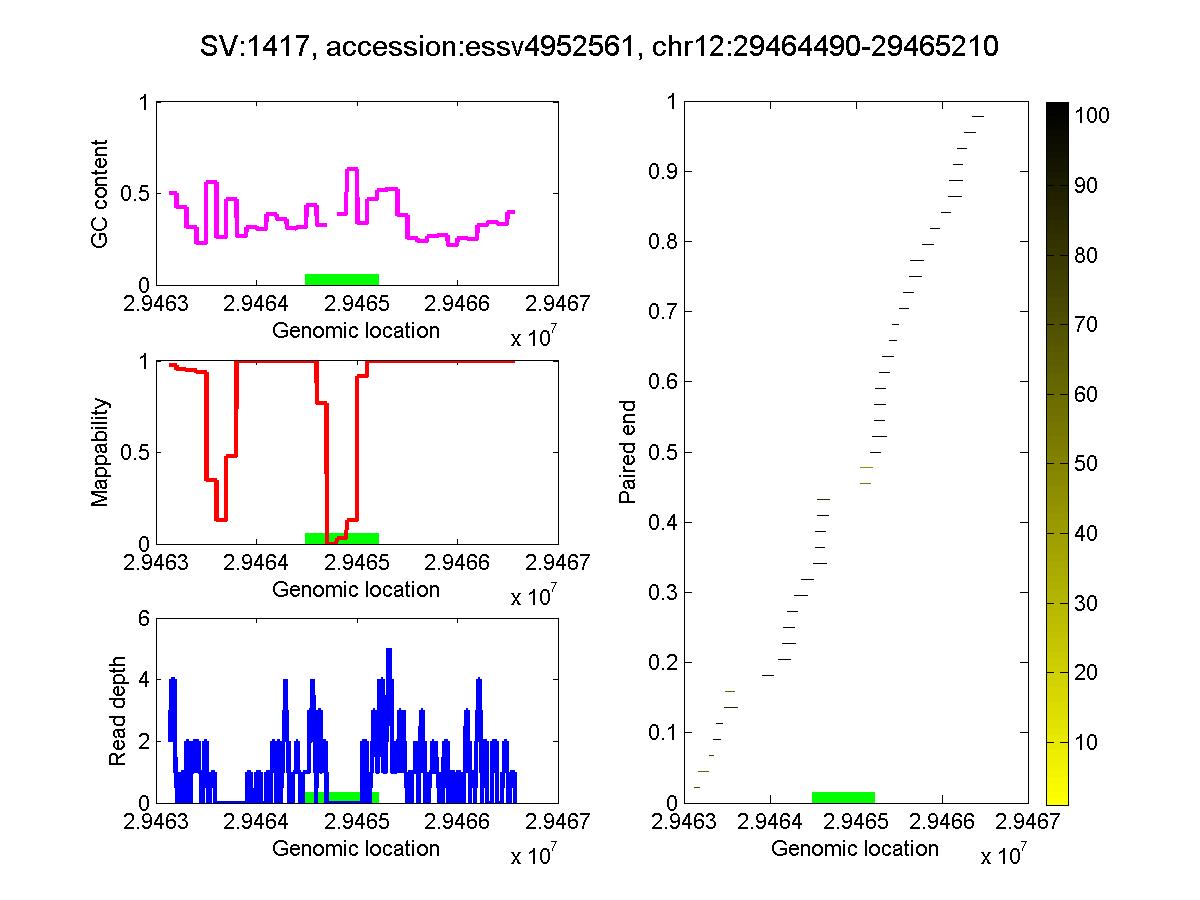

Supplement: Supplementary Materials — Supplementary data are available with this article at http://gr.xjtu.edu.cn/c/document_library/get_file?p_l_id=2403541&folderId=2539941&name=DLFE-115097.zip. Table S1 lists the complete information of suspicious variants and false positives, and the FIG directory contains the validation figures of each false positive. [file 8420547.f1.zip › 8420547.f1/FIG/SV1417.jpg]

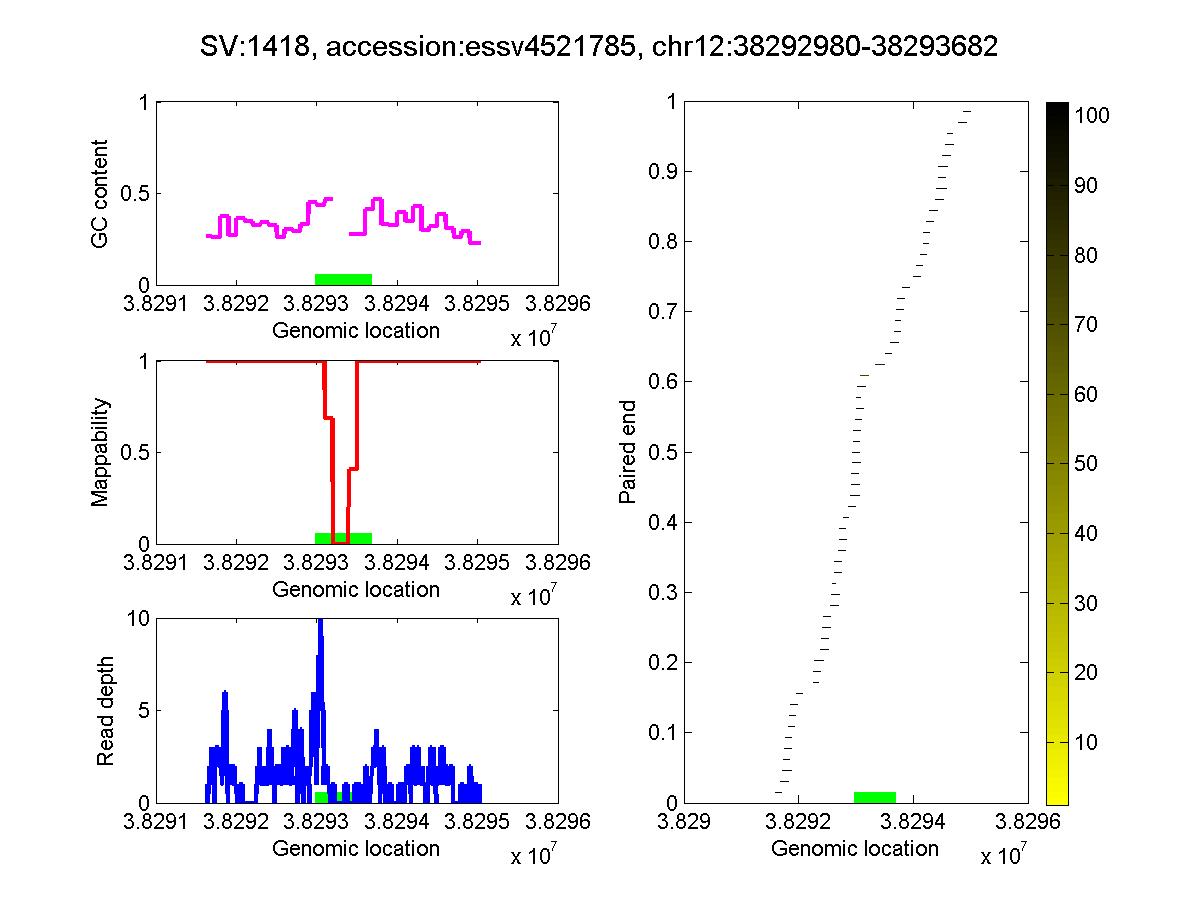

Supplement: Supplementary Materials — Supplementary data are available with this article at http://gr.xjtu.edu.cn/c/document_library/get_file?p_l_id=2403541&folderId=2539941&name=DLFE-115097.zip. Table S1 lists the complete information of suspicious variants and false positives, and the FIG directory contains the validation figures of each false positive. [file 8420547.f1.zip › 8420547.f1/FIG/SV1418.jpg]

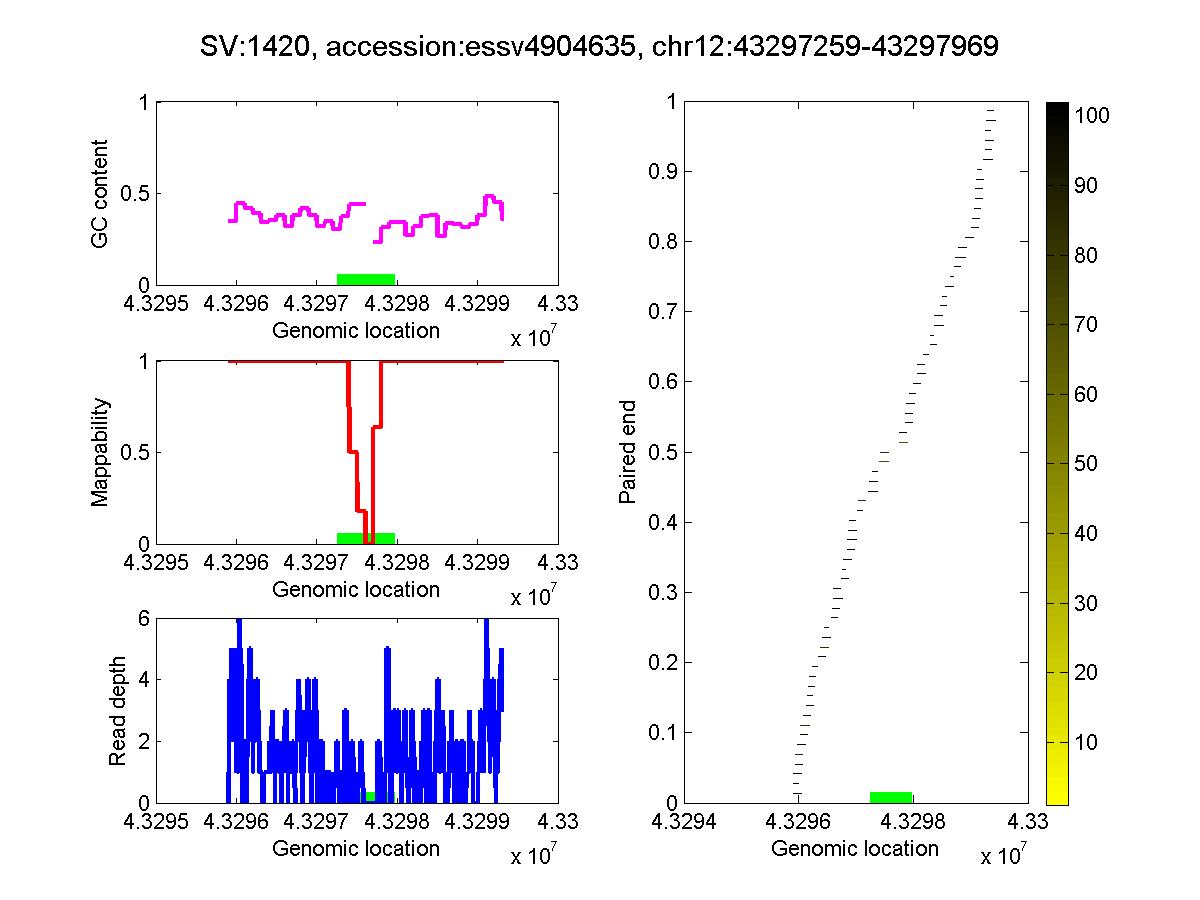

Supplement: Supplementary Materials — Supplementary data are available with this article at http://gr.xjtu.edu.cn/c/document_library/get_file?p_l_id=2403541&folderId=2539941&name=DLFE-115097.zip. Table S1 lists the complete information of suspicious variants and false positives, and the FIG directory contains the validation figures of each false positive. [file 8420547.f1.zip › 8420547.f1/FIG/SV1420.jpg]

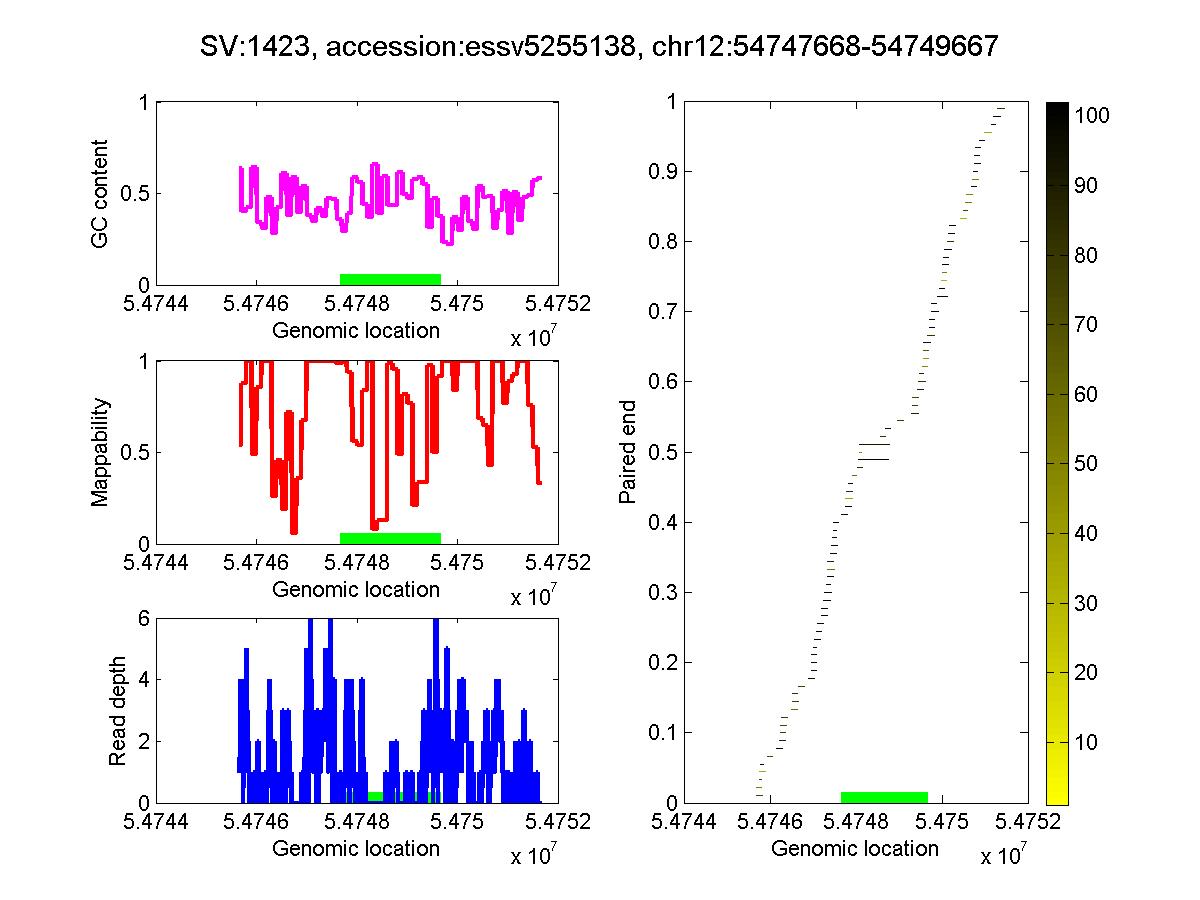

Supplement: Supplementary Materials — Supplementary data are available with this article at http://gr.xjtu.edu.cn/c/document_library/get_file?p_l_id=2403541&folderId=2539941&name=DLFE-115097.zip. Table S1 lists the complete information of suspicious variants and false positives, and the FIG directory contains the validation figures of each false positive. [file 8420547.f1.zip › 8420547.f1/FIG/SV1423.jpg]

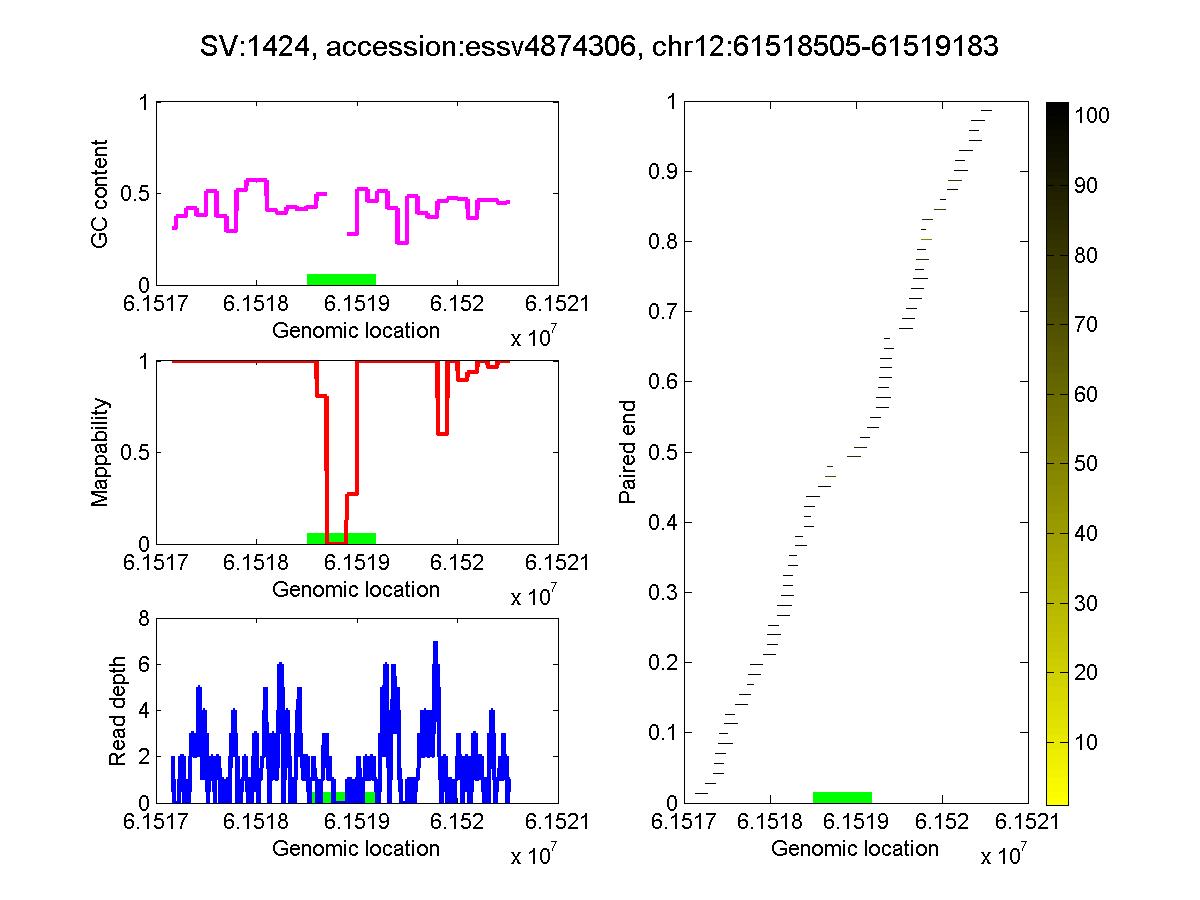

Supplement: Supplementary Materials — Supplementary data are available with this article at http://gr.xjtu.edu.cn/c/document_library/get_file?p_l_id=2403541&folderId=2539941&name=DLFE-115097.zip. Table S1 lists the complete information of suspicious variants and false positives, and the FIG directory contains the validation figures of each false positive. [file 8420547.f1.zip › 8420547.f1/FIG/SV1424.jpg]

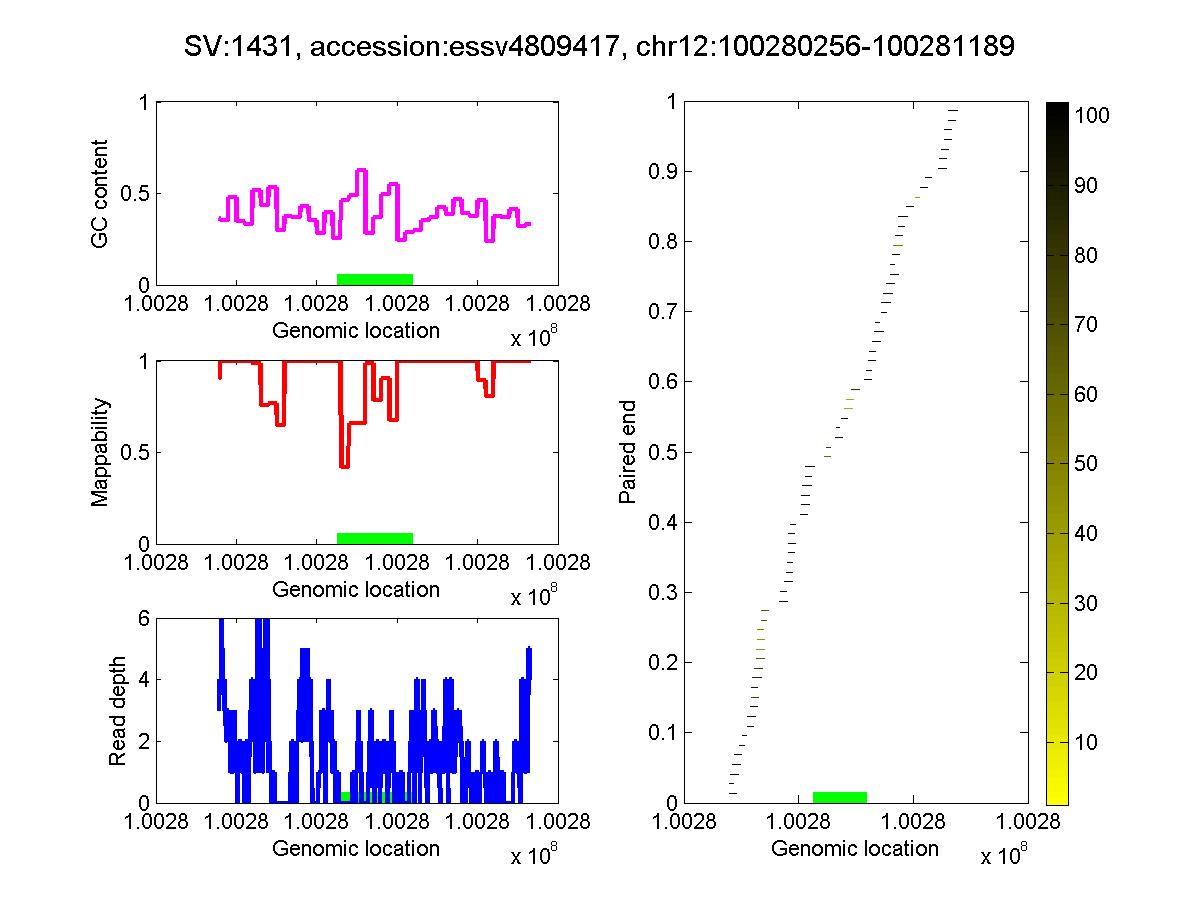

Supplement: Supplementary Materials — Supplementary data are available with this article at http://gr.xjtu.edu.cn/c/document_library/get_file?p_l_id=2403541&folderId=2539941&name=DLFE-115097.zip. Table S1 lists the complete information of suspicious variants and false positives, and the FIG directory contains the validation figures of each false positive. [file 8420547.f1.zip › 8420547.f1/FIG/SV1431.jpg]

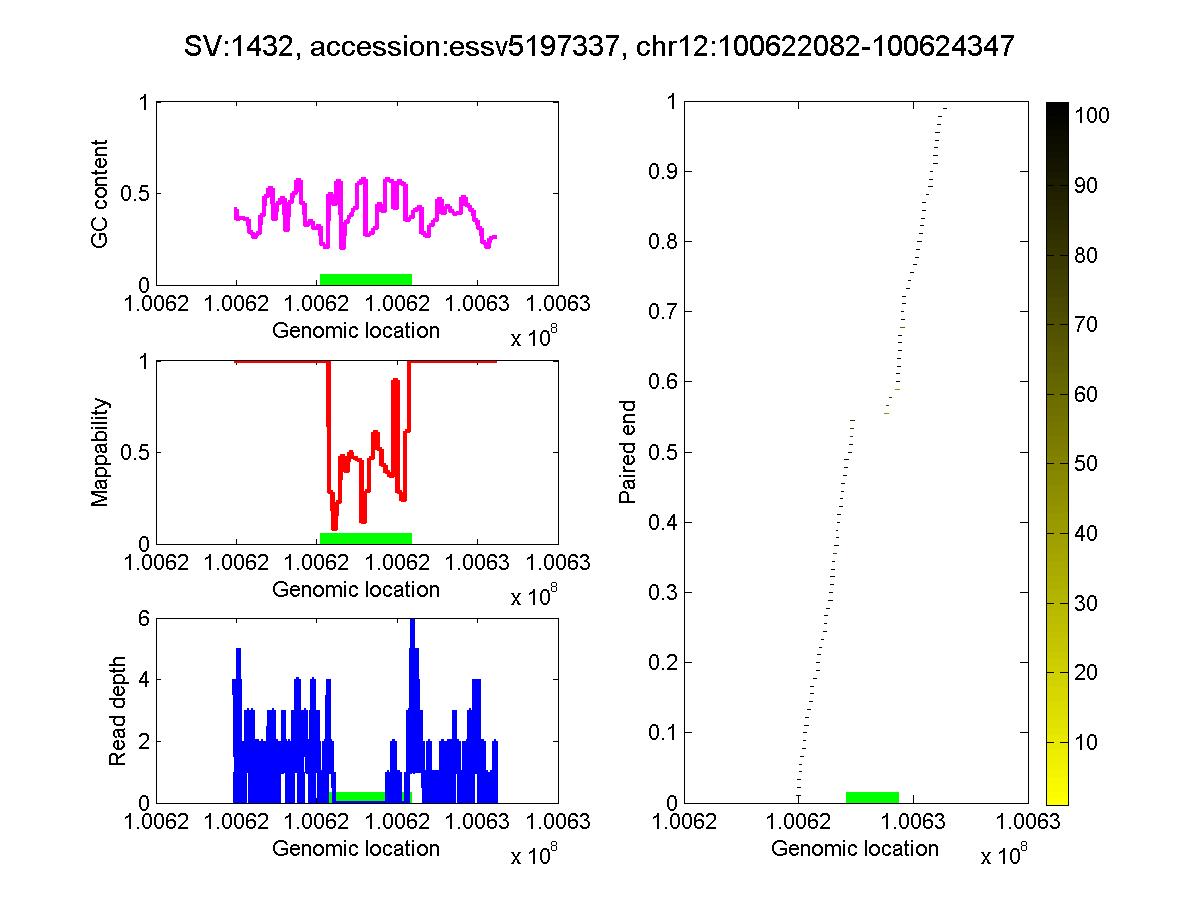

Supplement: Supplementary Materials — Supplementary data are available with this article at http://gr.xjtu.edu.cn/c/document_library/get_file?p_l_id=2403541&folderId=2539941&name=DLFE-115097.zip. Table S1 lists the complete information of suspicious variants and false positives, and the FIG directory contains the validation figures of each false positive. [file 8420547.f1.zip › 8420547.f1/FIG/SV1432.jpg]

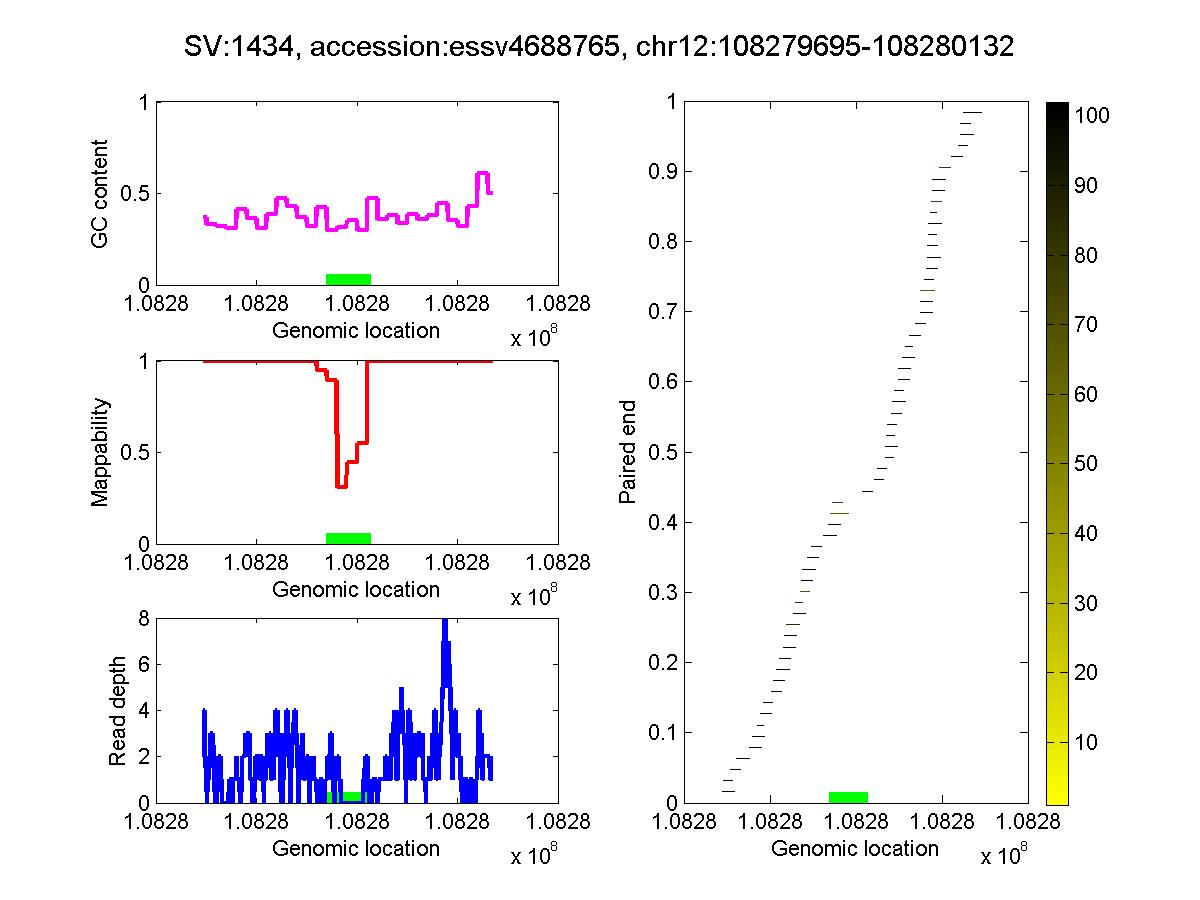

Supplement: Supplementary Materials — Supplementary data are available with this article at http://gr.xjtu.edu.cn/c/document_library/get_file?p_l_id=2403541&folderId=2539941&name=DLFE-115097.zip. Table S1 lists the complete information of suspicious variants and false positives, and the FIG directory contains the validation figures of each false positive. [file 8420547.f1.zip › 8420547.f1/FIG/SV1434.jpg]

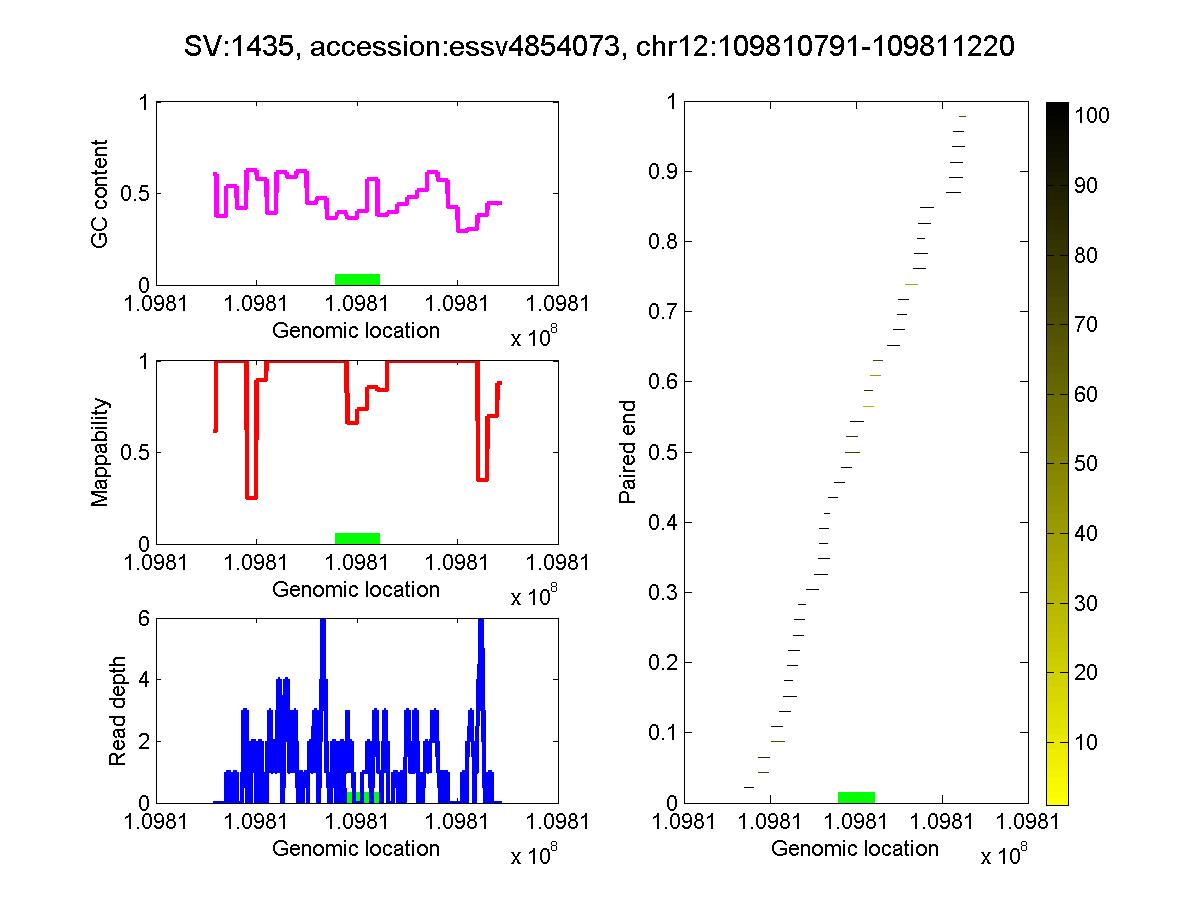

Supplement: Supplementary Materials — Supplementary data are available with this article at http://gr.xjtu.edu.cn/c/document_library/get_file?p_l_id=2403541&folderId=2539941&name=DLFE-115097.zip. Table S1 lists the complete information of suspicious variants and false positives, and the FIG directory contains the validation figures of each false positive. [file 8420547.f1.zip › 8420547.f1/FIG/SV1435.jpg]

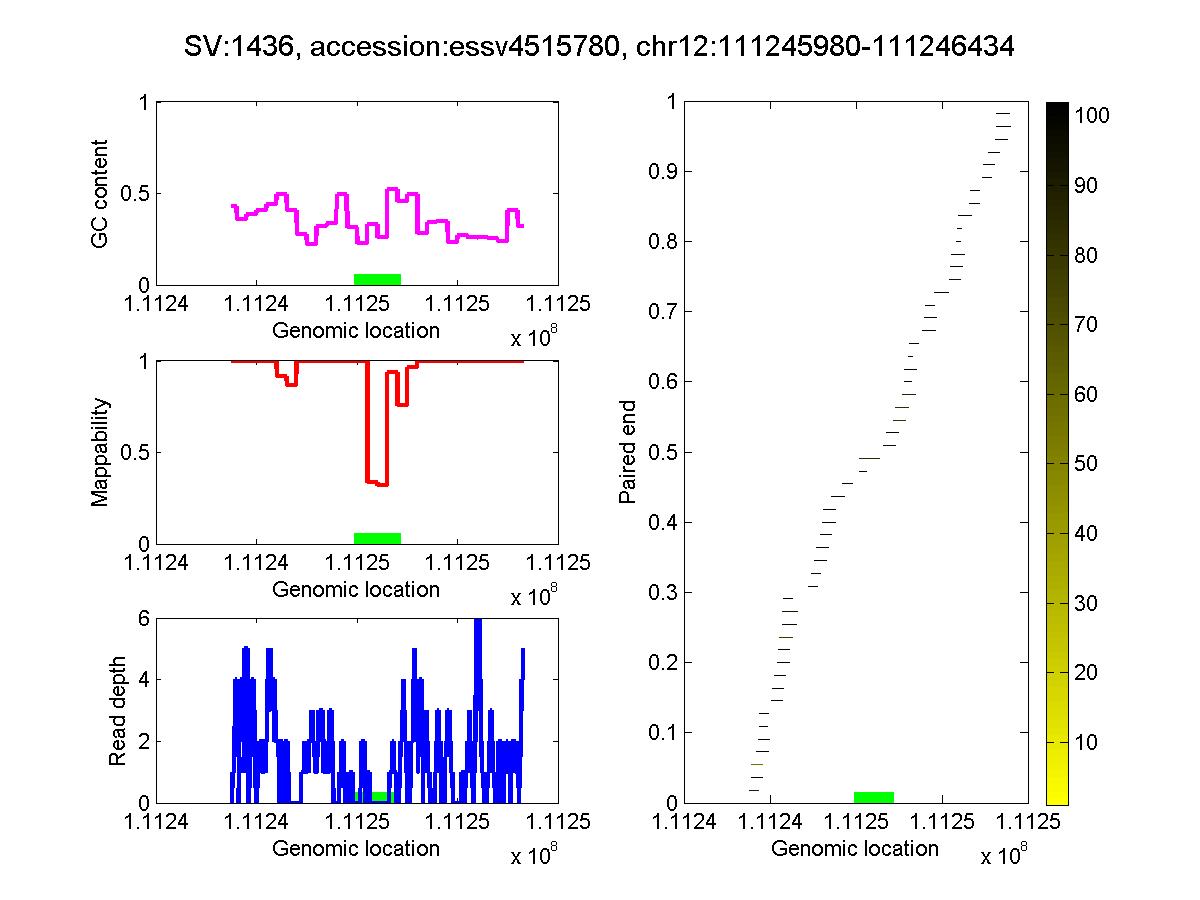

Supplement: Supplementary Materials — Supplementary data are available with this article at http://gr.xjtu.edu.cn/c/document_library/get_file?p_l_id=2403541&folderId=2539941&name=DLFE-115097.zip. Table S1 lists the complete information of suspicious variants and false positives, and the FIG directory contains the validation figures of each false positive. [file 8420547.f1.zip › 8420547.f1/FIG/SV1436.jpg]

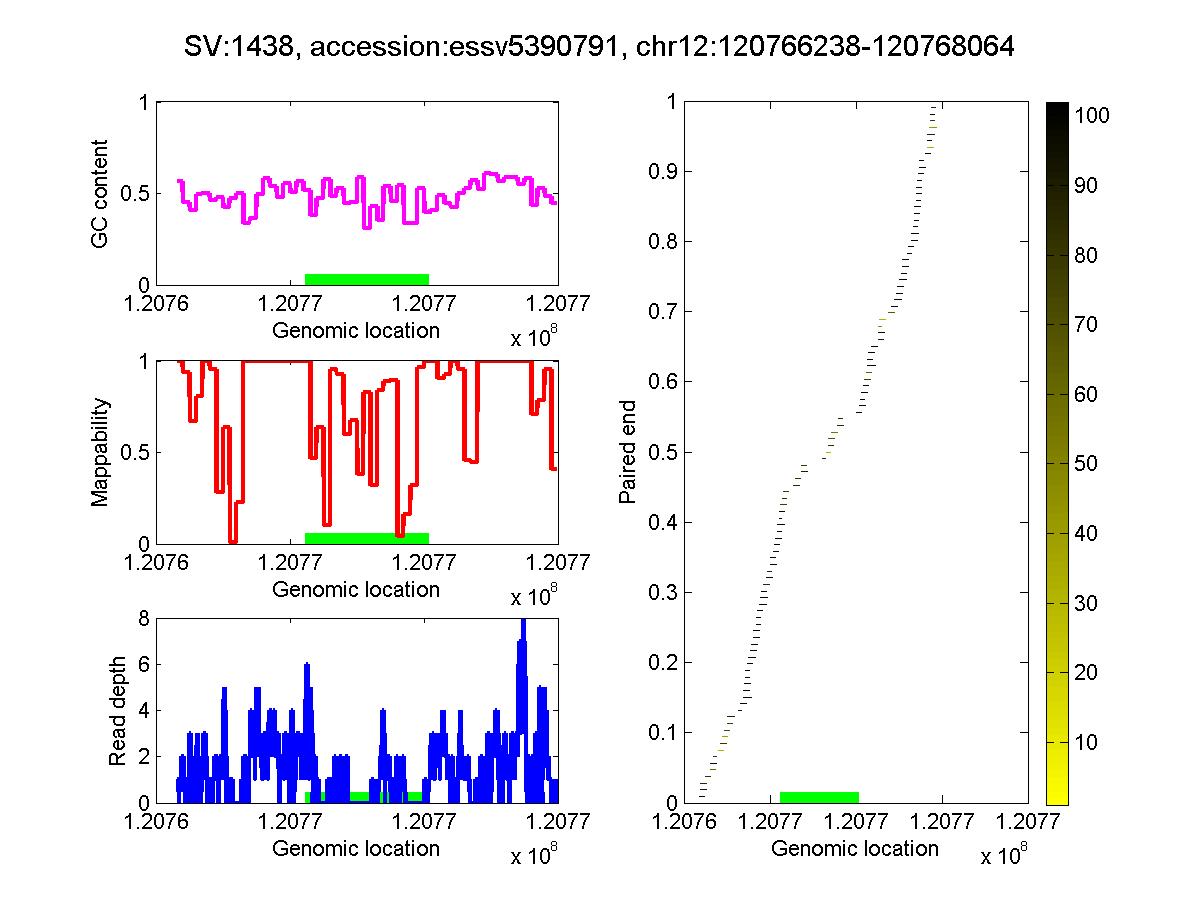

Supplement: Supplementary Materials — Supplementary data are available with this article at http://gr.xjtu.edu.cn/c/document_library/get_file?p_l_id=2403541&folderId=2539941&name=DLFE-115097.zip. Table S1 lists the complete information of suspicious variants and false positives, and the FIG directory contains the validation figures of each false positive. [file 8420547.f1.zip › 8420547.f1/FIG/SV1438.jpg]

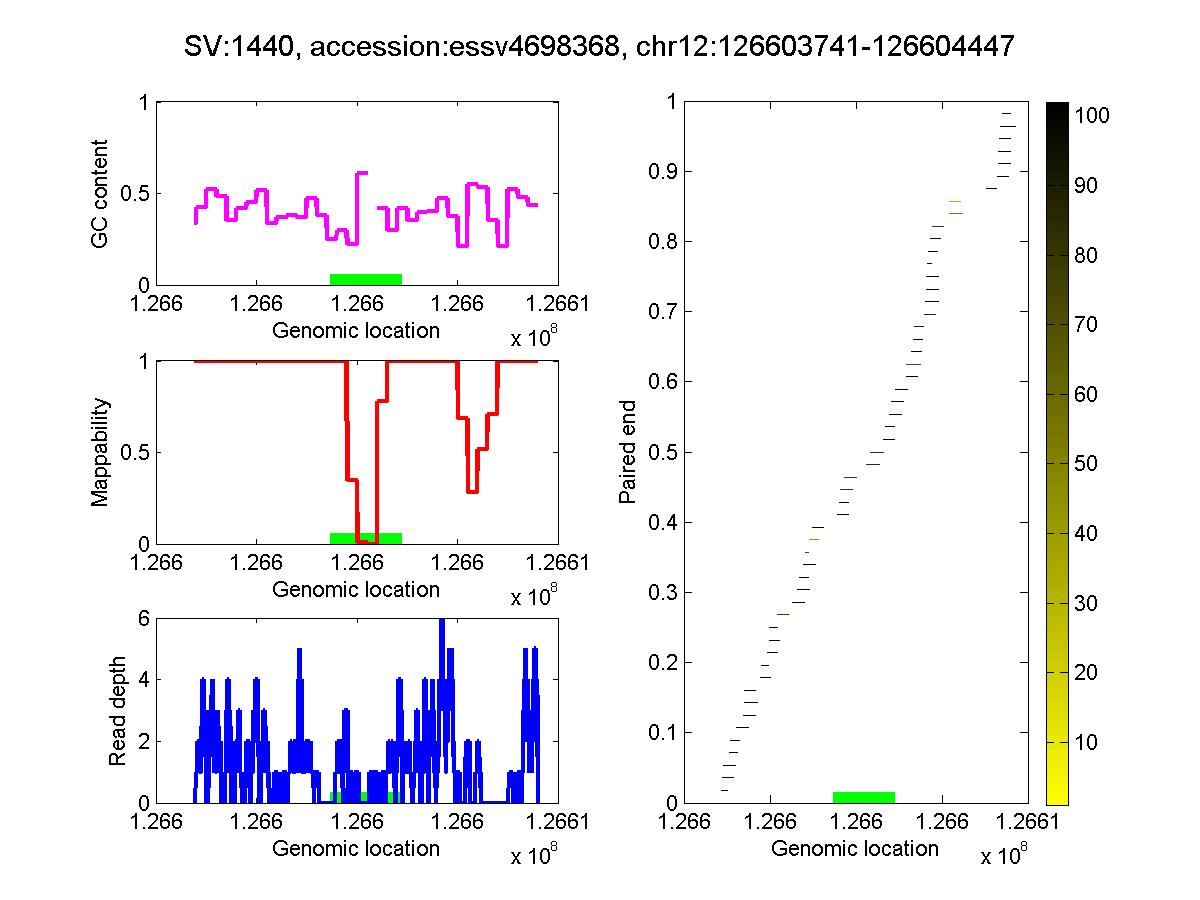

Supplement: Supplementary Materials — Supplementary data are available with this article at http://gr.xjtu.edu.cn/c/document_library/get_file?p_l_id=2403541&folderId=2539941&name=DLFE-115097.zip. Table S1 lists the complete information of suspicious variants and false positives, and the FIG directory contains the validation figures of each false positive. [file 8420547.f1.zip › 8420547.f1/FIG/SV1440.jpg]

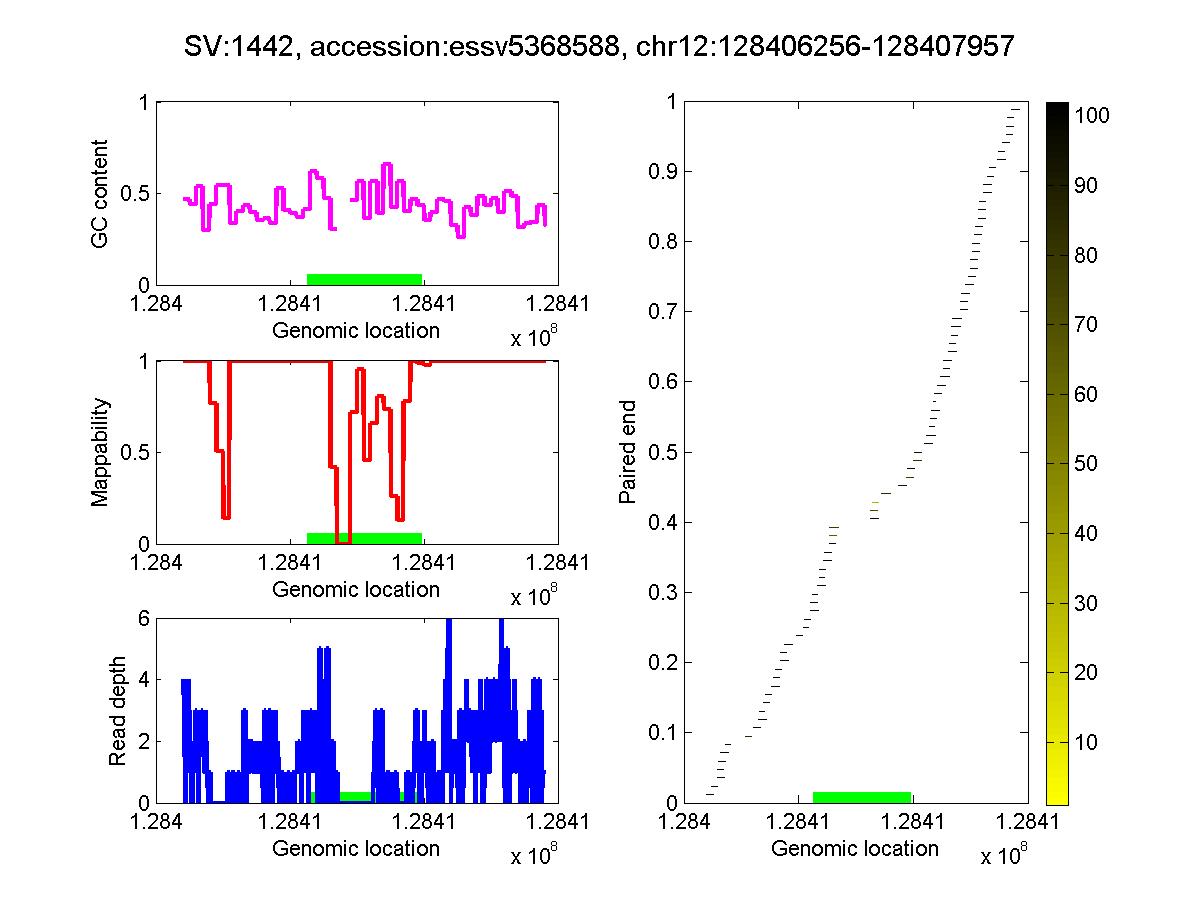

Supplement: Supplementary Materials — Supplementary data are available with this article at http://gr.xjtu.edu.cn/c/document_library/get_file?p_l_id=2403541&folderId=2539941&name=DLFE-115097.zip. Table S1 lists the complete information of suspicious variants and false positives, and the FIG directory contains the validation figures of each false positive. [file 8420547.f1.zip › 8420547.f1/FIG/SV1442.jpg]

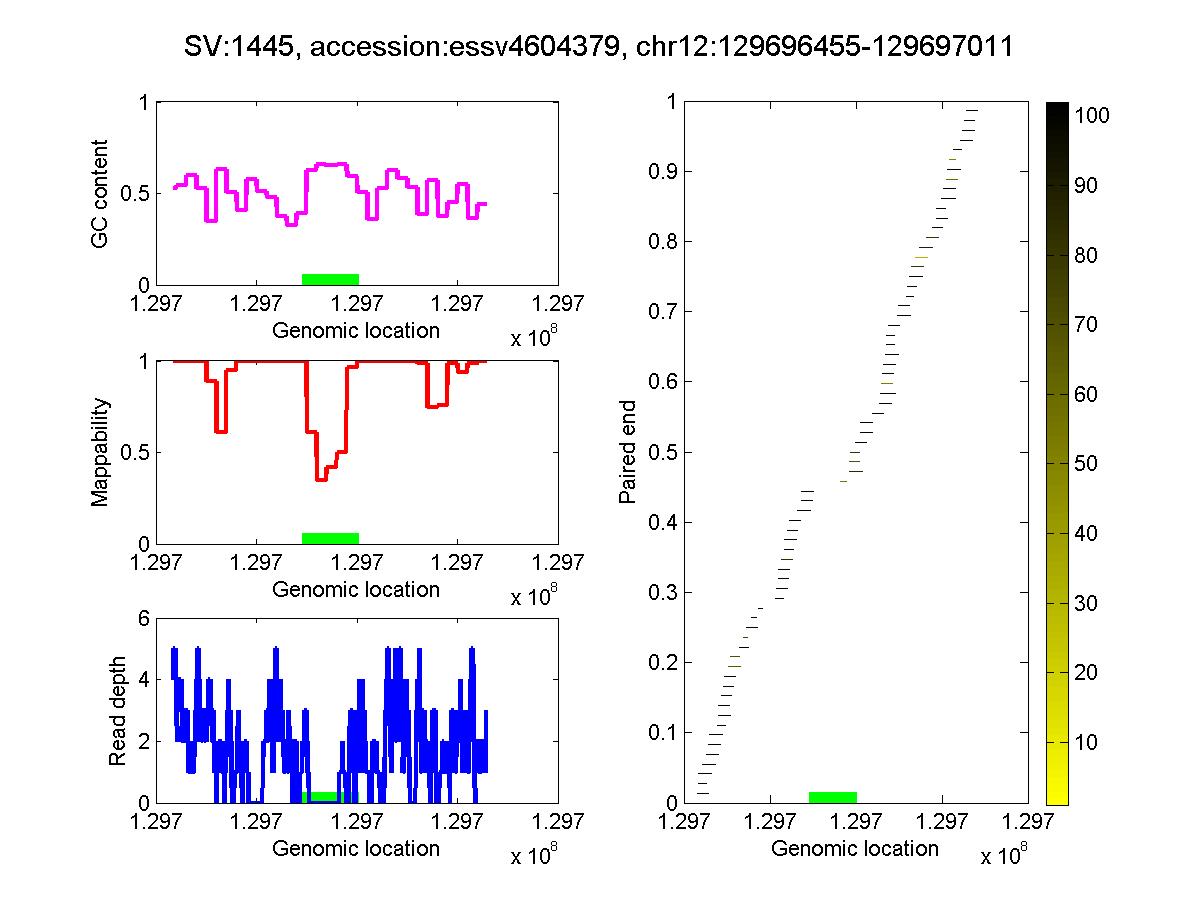

Supplement: Supplementary Materials — Supplementary data are available with this article at http://gr.xjtu.edu.cn/c/document_library/get_file?p_l_id=2403541&folderId=2539941&name=DLFE-115097.zip. Table S1 lists the complete information of suspicious variants and false positives, and the FIG directory contains the validation figures of each false positive. [file 8420547.f1.zip › 8420547.f1/FIG/SV1445.jpg]

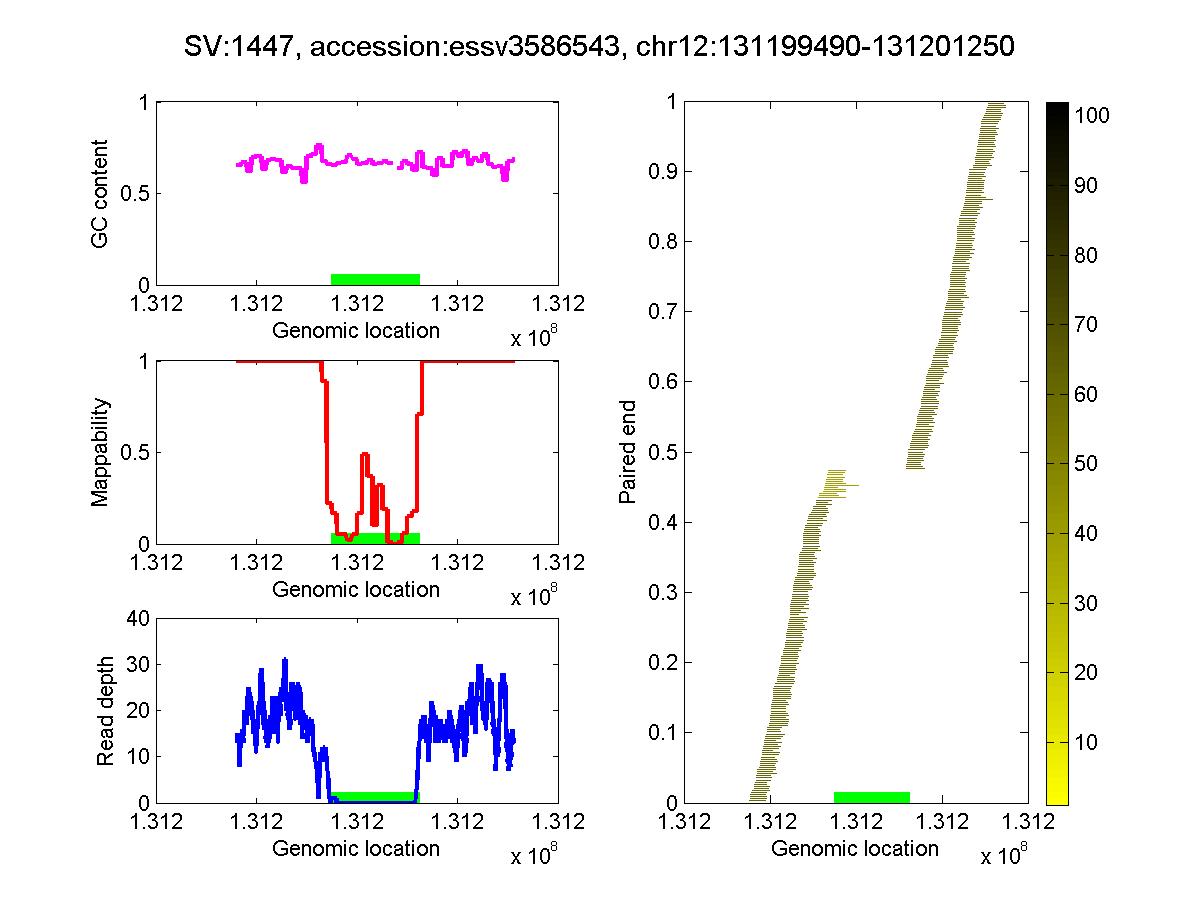

Supplement: Supplementary Materials — Supplementary data are available with this article at http://gr.xjtu.edu.cn/c/document_library/get_file?p_l_id=2403541&folderId=2539941&name=DLFE-115097.zip. Table S1 lists the complete information of suspicious variants and false positives, and the FIG directory contains the validation figures of each false positive. [file 8420547.f1.zip › 8420547.f1/FIG/SV1447.jpg]

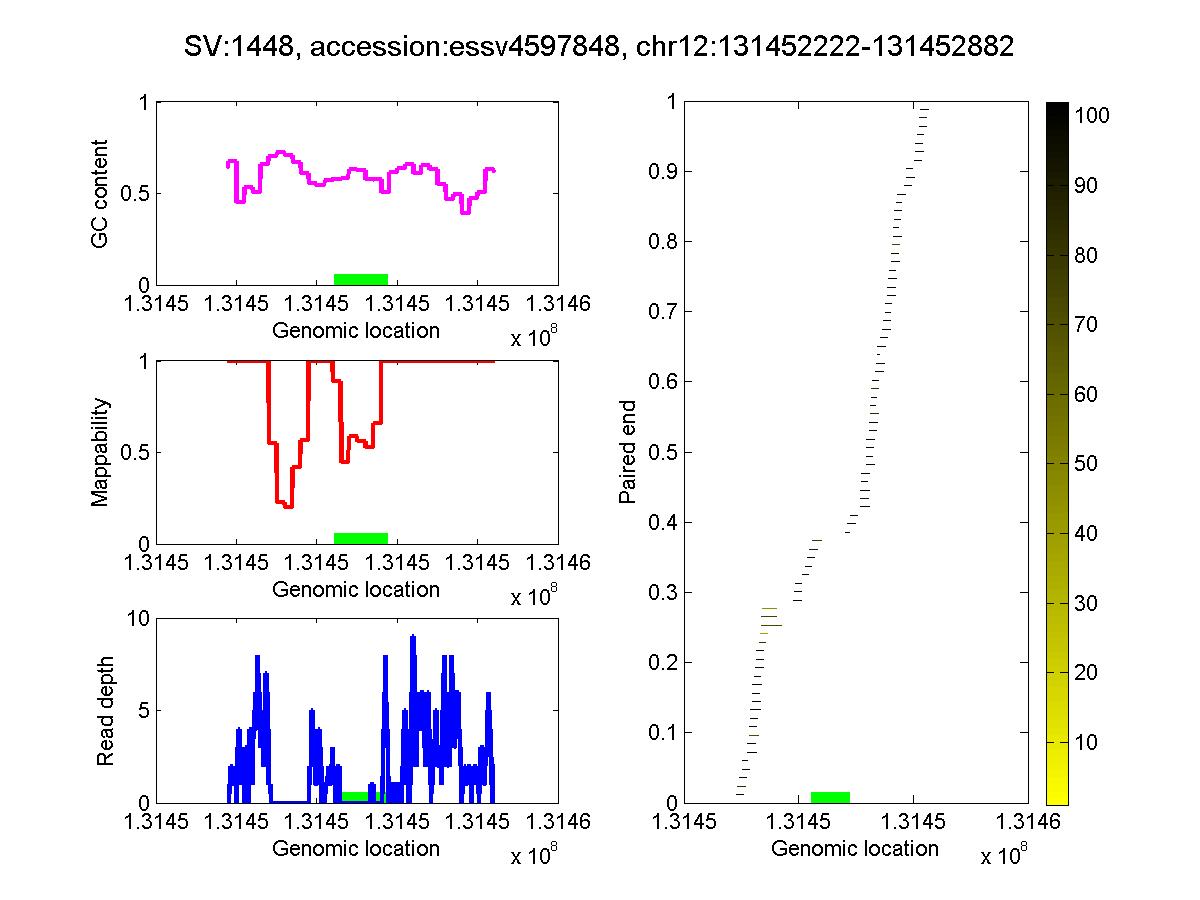

Supplement: Supplementary Materials — Supplementary data are available with this article at http://gr.xjtu.edu.cn/c/document_library/get_file?p_l_id=2403541&folderId=2539941&name=DLFE-115097.zip. Table S1 lists the complete information of suspicious variants and false positives, and the FIG directory contains the validation figures of each false positive. [file 8420547.f1.zip › 8420547.f1/FIG/SV1448.jpg]

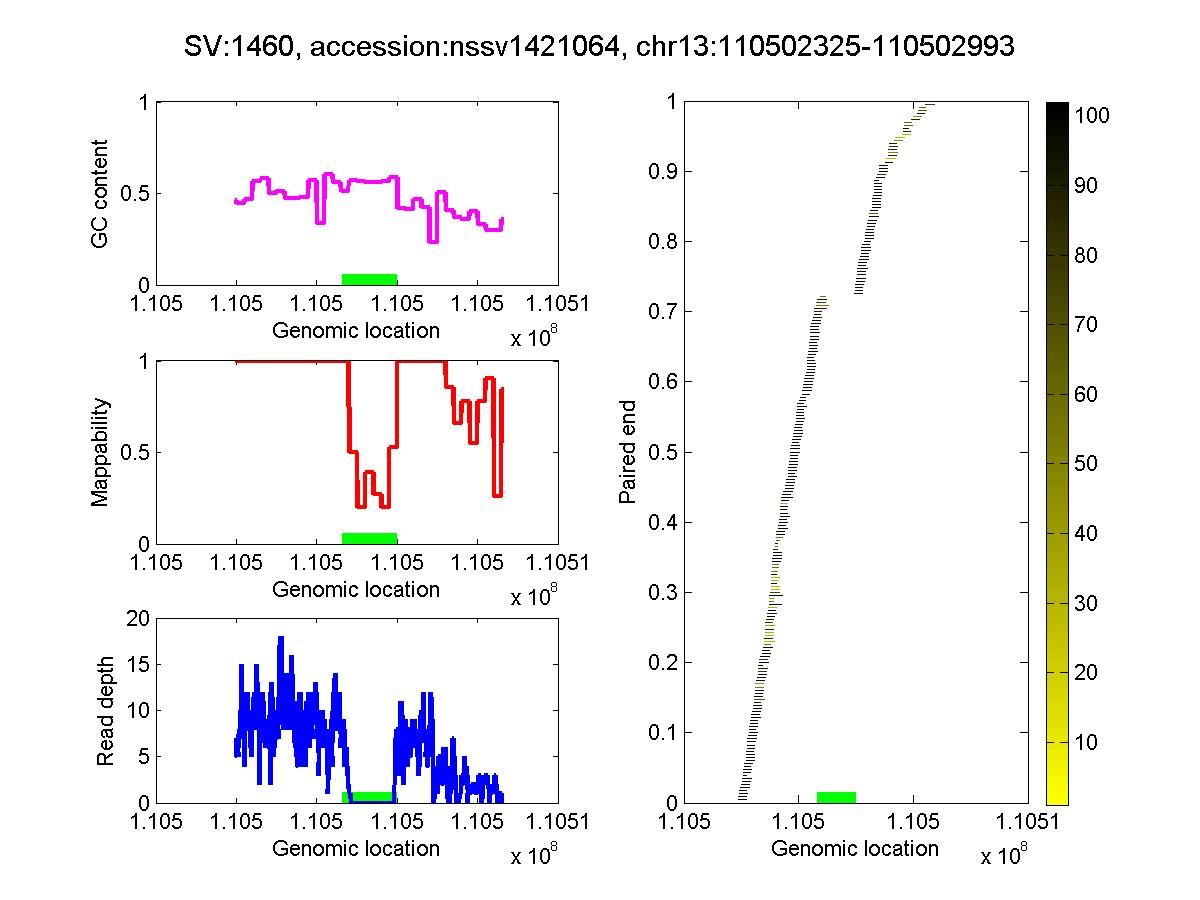

Supplement: Supplementary Materials — Supplementary data are available with this article at http://gr.xjtu.edu.cn/c/document_library/get_file?p_l_id=2403541&folderId=2539941&name=DLFE-115097.zip. Table S1 lists the complete information of suspicious variants and false positives, and the FIG directory contains the validation figures of each false positive. [file 8420547.f1.zip › 8420547.f1/FIG/SV1460.jpg]

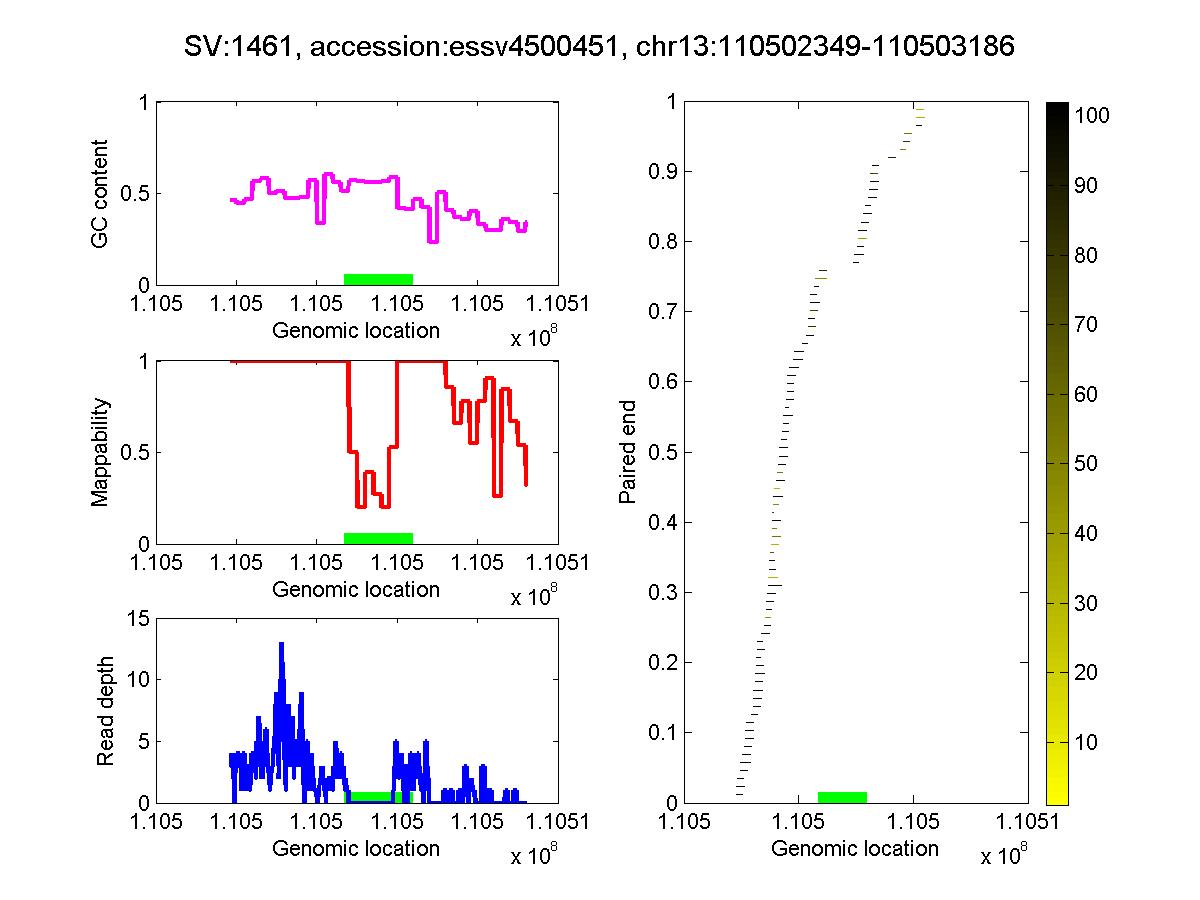

Supplement: Supplementary Materials — Supplementary data are available with this article at http://gr.xjtu.edu.cn/c/document_library/get_file?p_l_id=2403541&folderId=2539941&name=DLFE-115097.zip. Table S1 lists the complete information of suspicious variants and false positives, and the FIG directory contains the validation figures of each false positive. [file 8420547.f1.zip › 8420547.f1/FIG/SV1461.jpg]

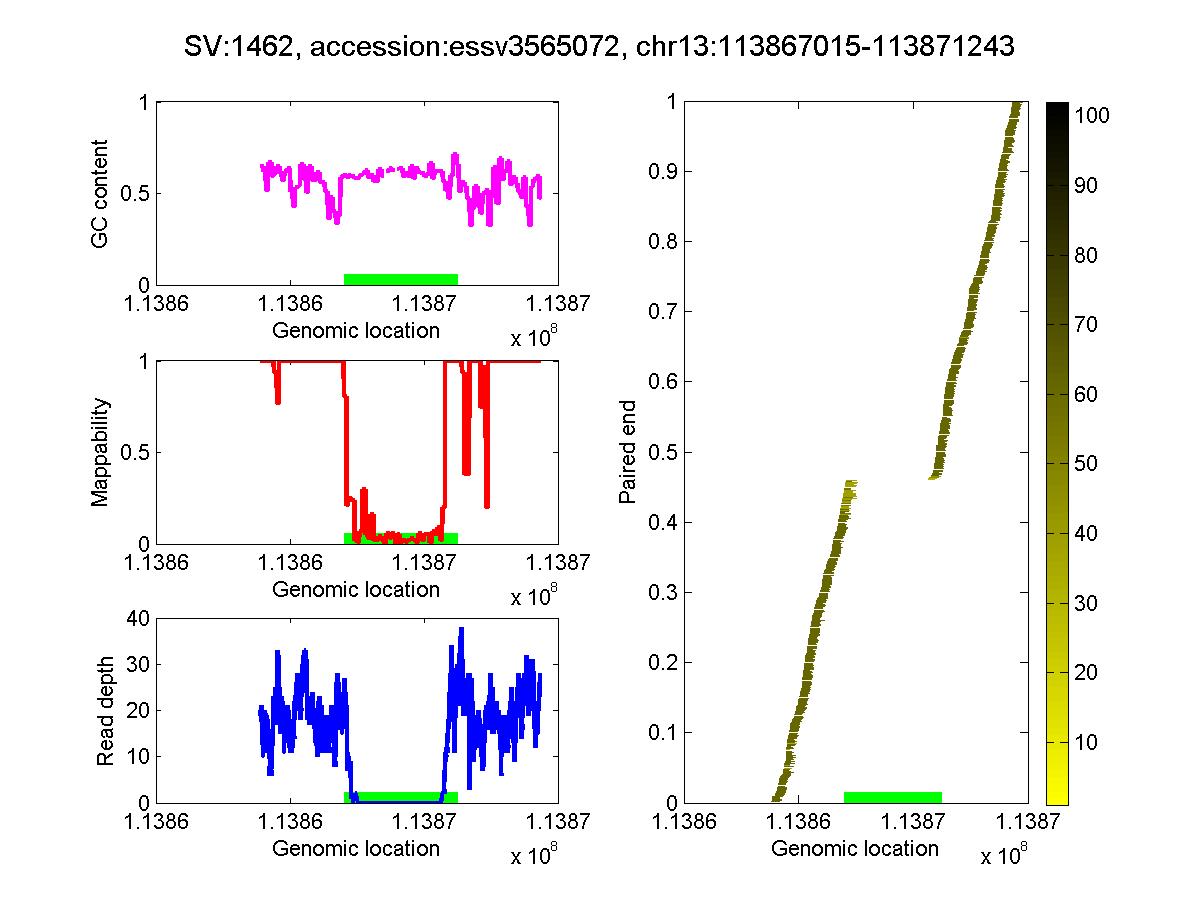

Supplement: Supplementary Materials — Supplementary data are available with this article at http://gr.xjtu.edu.cn/c/document_library/get_file?p_l_id=2403541&folderId=2539941&name=DLFE-115097.zip. Table S1 lists the complete information of suspicious variants and false positives, and the FIG directory contains the validation figures of each false positive. [file 8420547.f1.zip › 8420547.f1/FIG/SV1462.jpg]

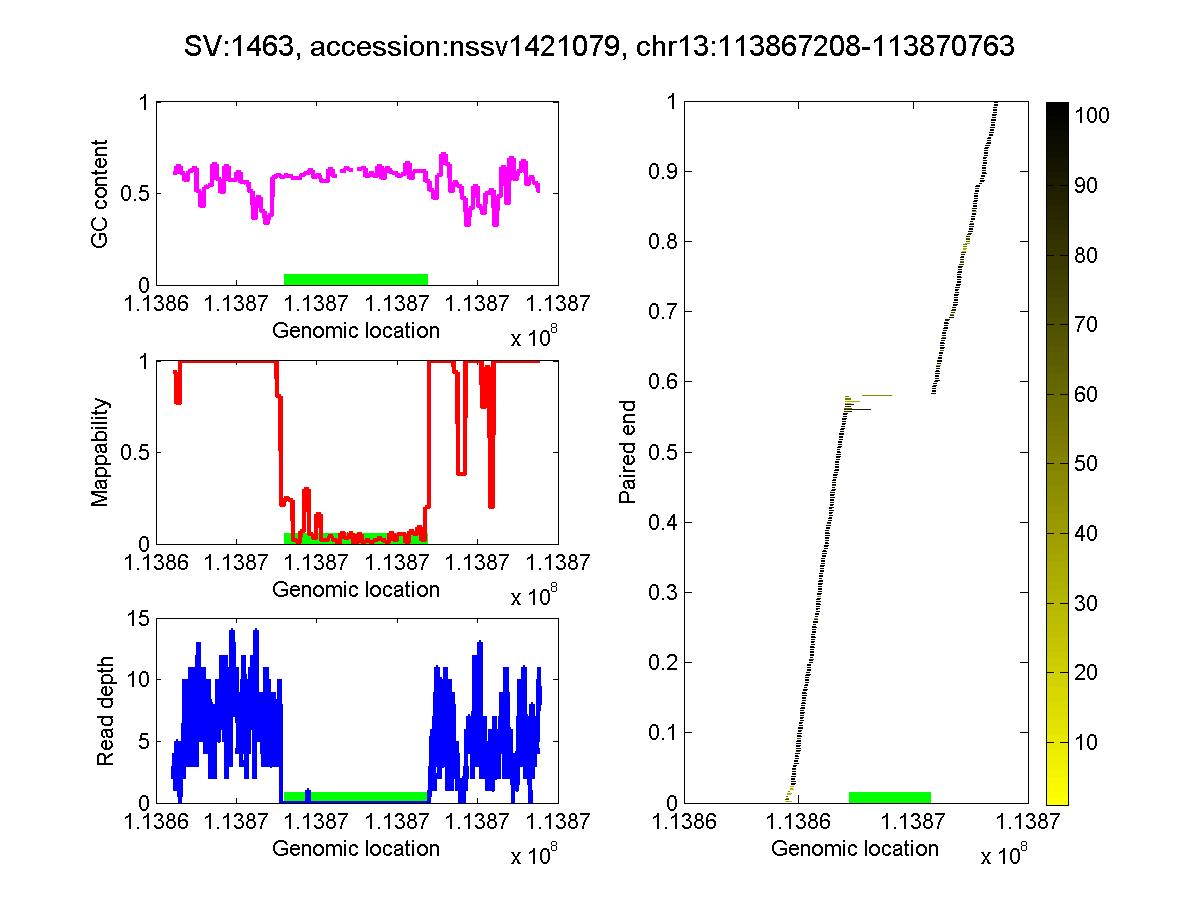

Supplement: Supplementary Materials — Supplementary data are available with this article at http://gr.xjtu.edu.cn/c/document_library/get_file?p_l_id=2403541&folderId=2539941&name=DLFE-115097.zip. Table S1 lists the complete information of suspicious variants and false positives, and the FIG directory contains the validation figures of each false positive. [file 8420547.f1.zip › 8420547.f1/FIG/SV1463.jpg]

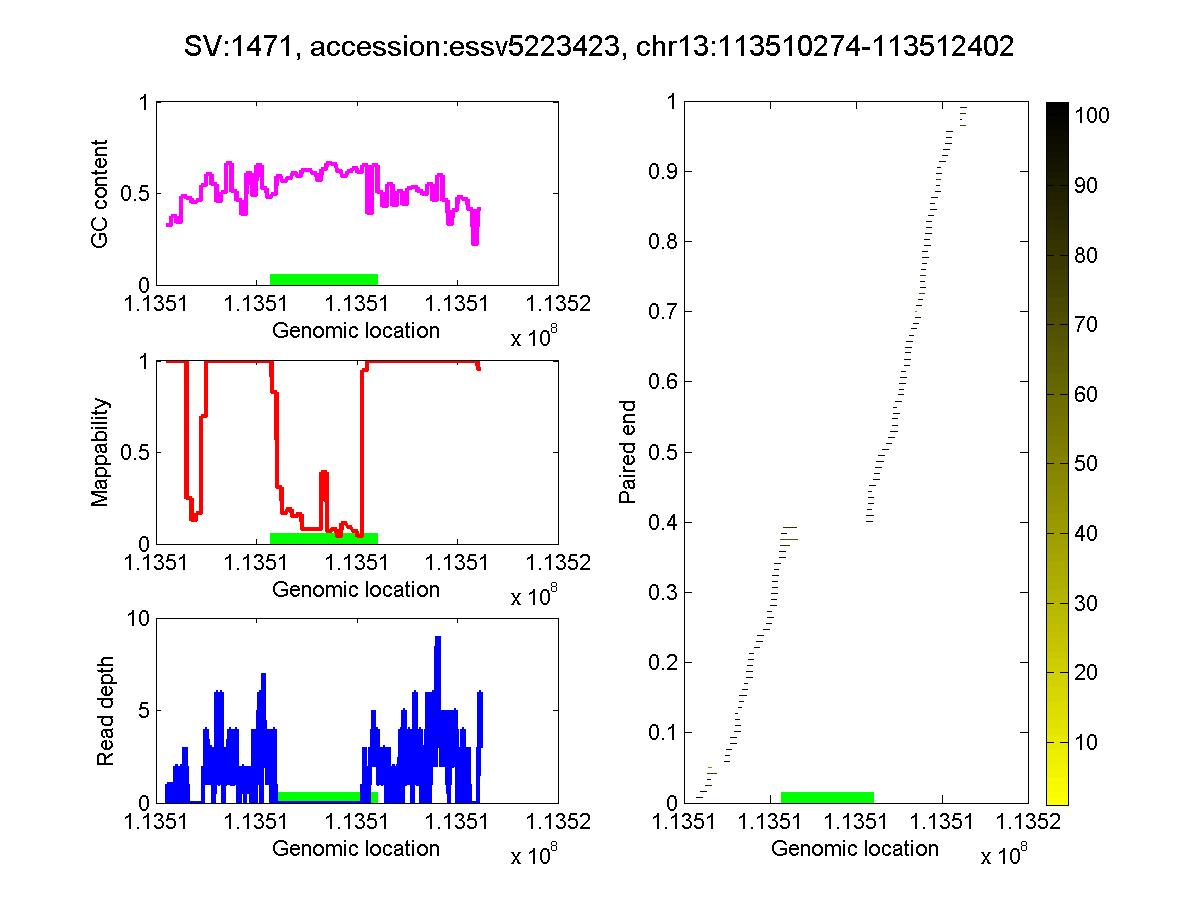

Supplement: Supplementary Materials — Supplementary data are available with this article at http://gr.xjtu.edu.cn/c/document_library/get_file?p_l_id=2403541&folderId=2539941&name=DLFE-115097.zip. Table S1 lists the complete information of suspicious variants and false positives, and the FIG directory contains the validation figures of each false positive. [file 8420547.f1.zip › 8420547.f1/FIG/SV1471.jpg]

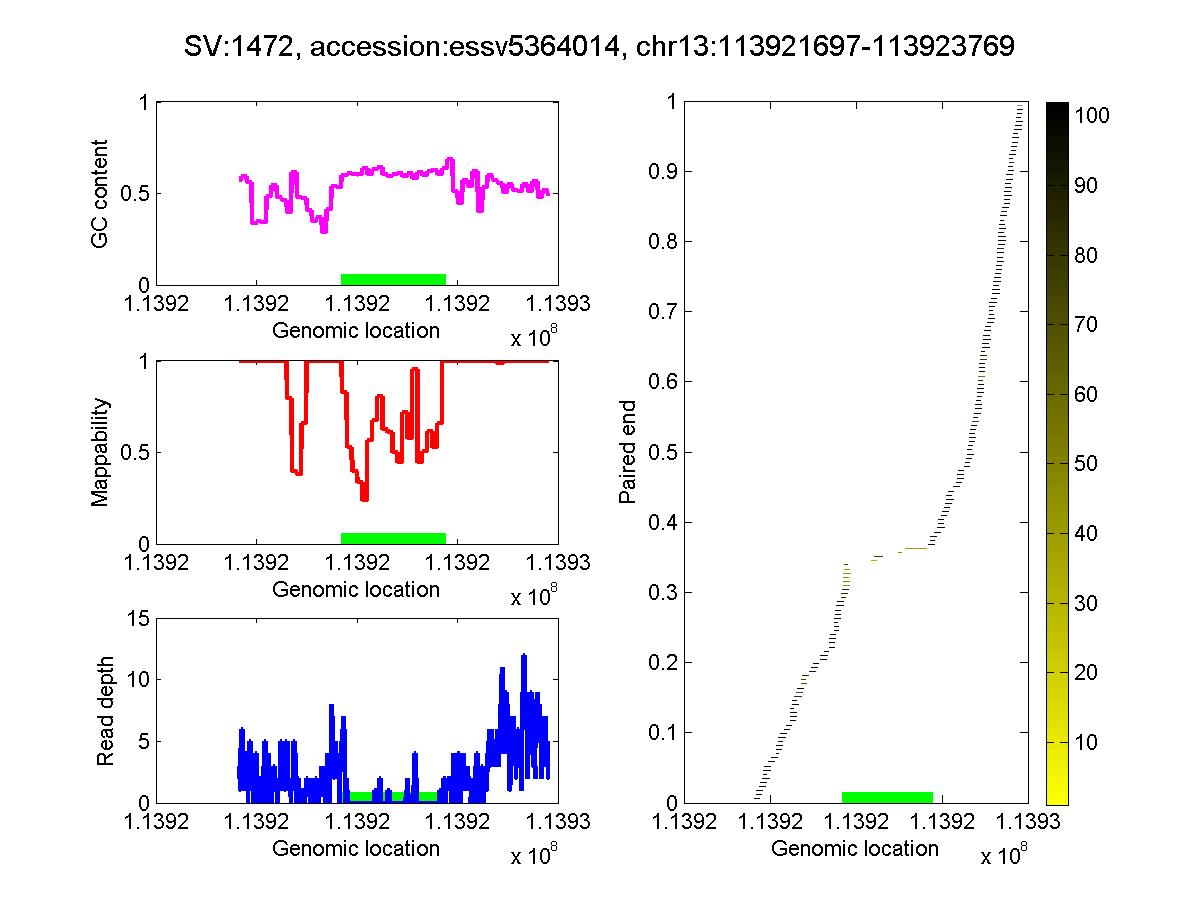

Supplement: Supplementary Materials — Supplementary data are available with this article at http://gr.xjtu.edu.cn/c/document_library/get_file?p_l_id=2403541&folderId=2539941&name=DLFE-115097.zip. Table S1 lists the complete information of suspicious variants and false positives, and the FIG directory contains the validation figures of each false positive. [file 8420547.f1.zip › 8420547.f1/FIG/SV1472.jpg]

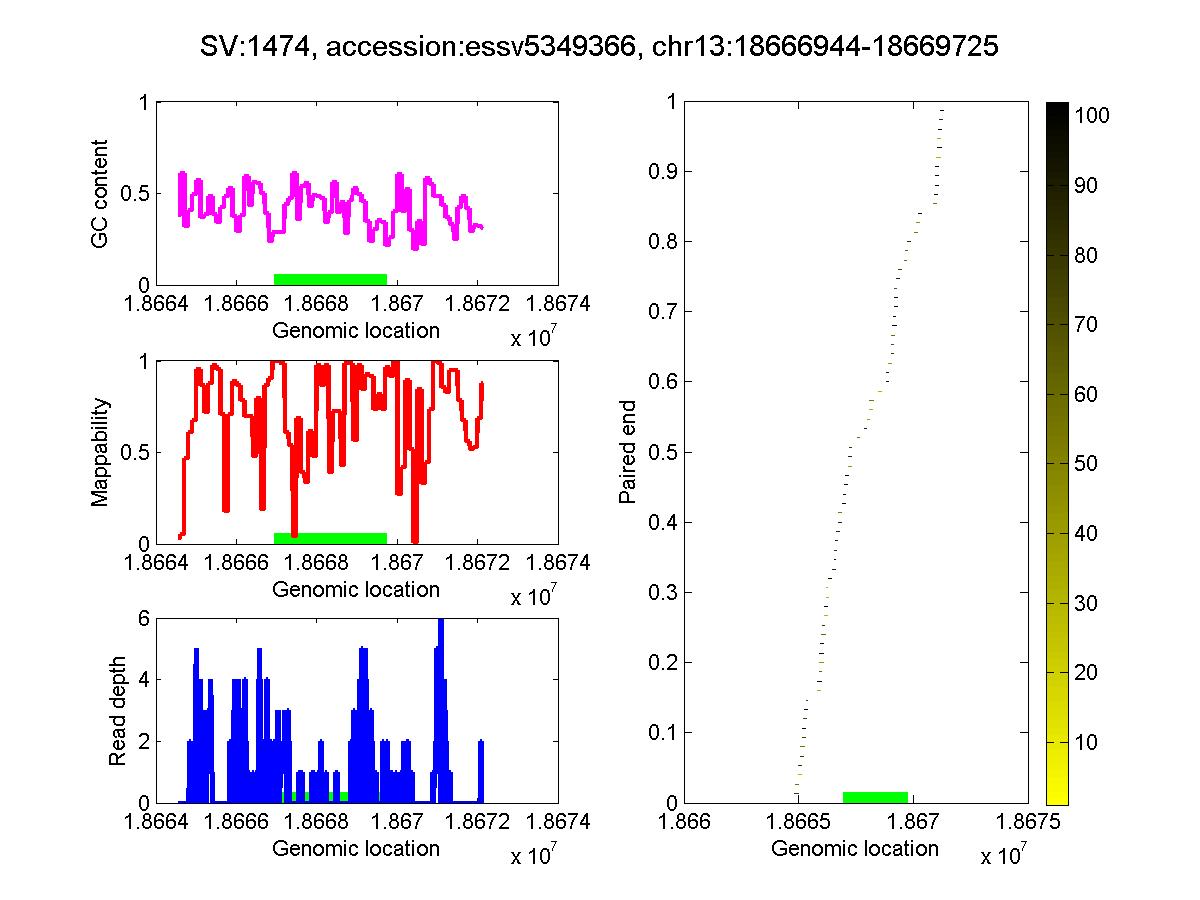

Supplement: Supplementary Materials — Supplementary data are available with this article at http://gr.xjtu.edu.cn/c/document_library/get_file?p_l_id=2403541&folderId=2539941&name=DLFE-115097.zip. Table S1 lists the complete information of suspicious variants and false positives, and the FIG directory contains the validation figures of each false positive. [file 8420547.f1.zip › 8420547.f1/FIG/SV1474.jpg]

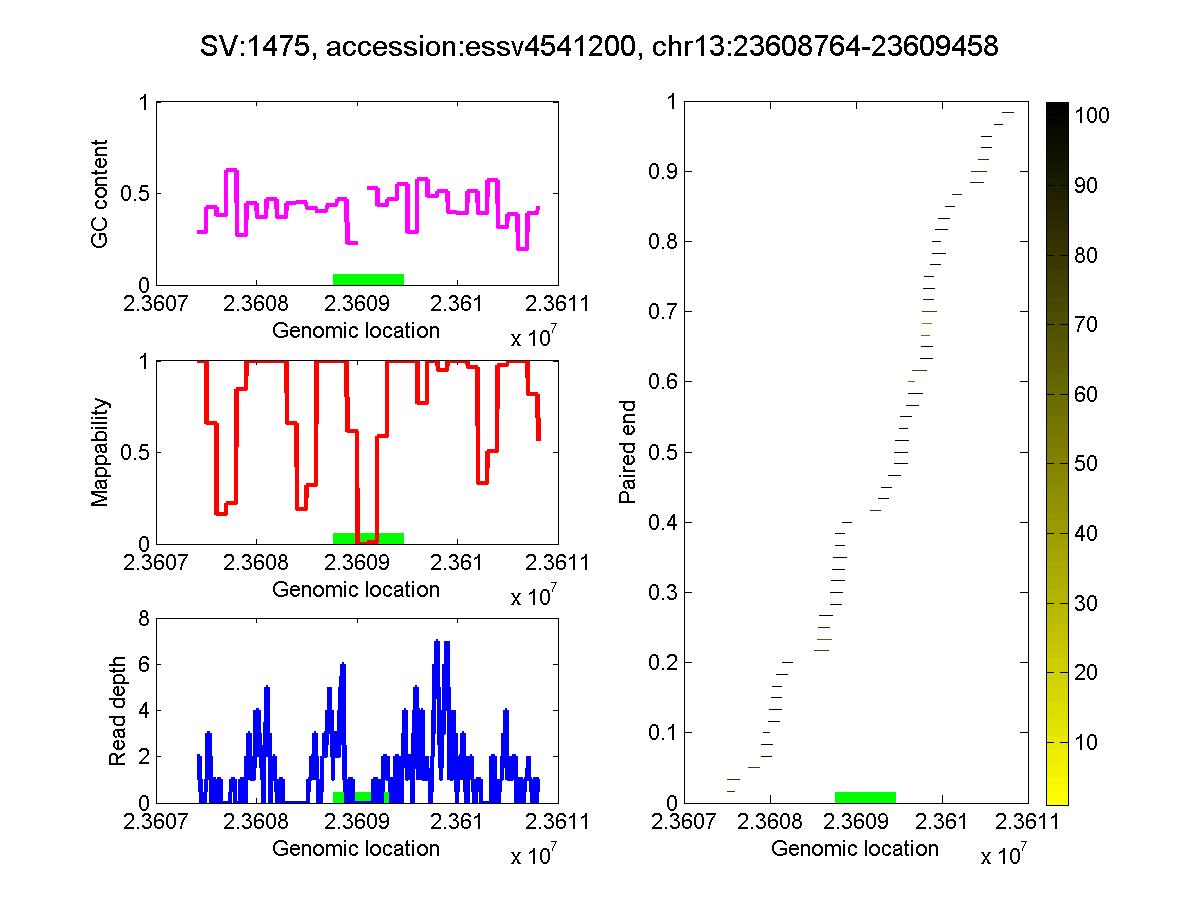

Supplement: Supplementary Materials — Supplementary data are available with this article at http://gr.xjtu.edu.cn/c/document_library/get_file?p_l_id=2403541&folderId=2539941&name=DLFE-115097.zip. Table S1 lists the complete information of suspicious variants and false positives, and the FIG directory contains the validation figures of each false positive. [file 8420547.f1.zip › 8420547.f1/FIG/SV1475.jpg]

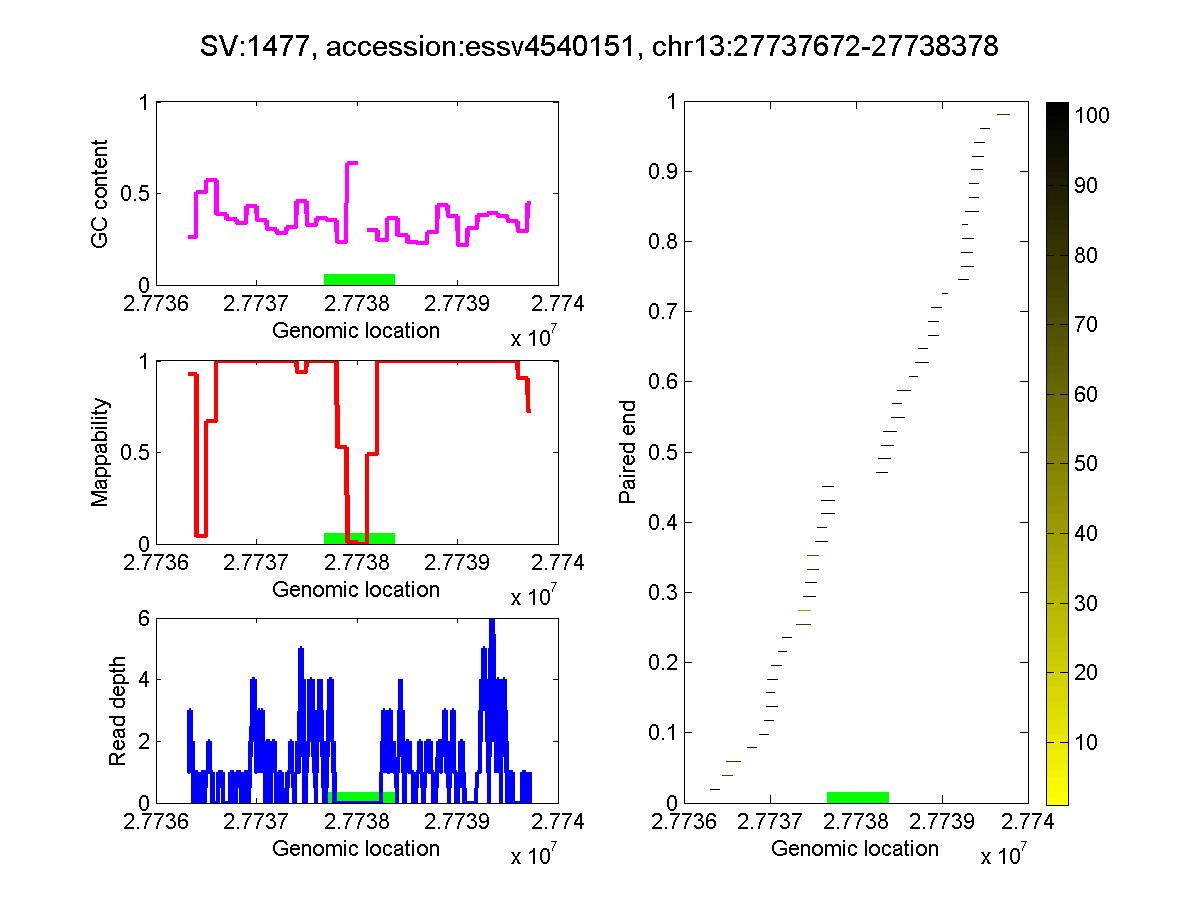

Supplement: Supplementary Materials — Supplementary data are available with this article at http://gr.xjtu.edu.cn/c/document_library/get_file?p_l_id=2403541&folderId=2539941&name=DLFE-115097.zip. Table S1 lists the complete information of suspicious variants and false positives, and the FIG directory contains the validation figures of each false positive. [file 8420547.f1.zip › 8420547.f1/FIG/SV1477.jpg]

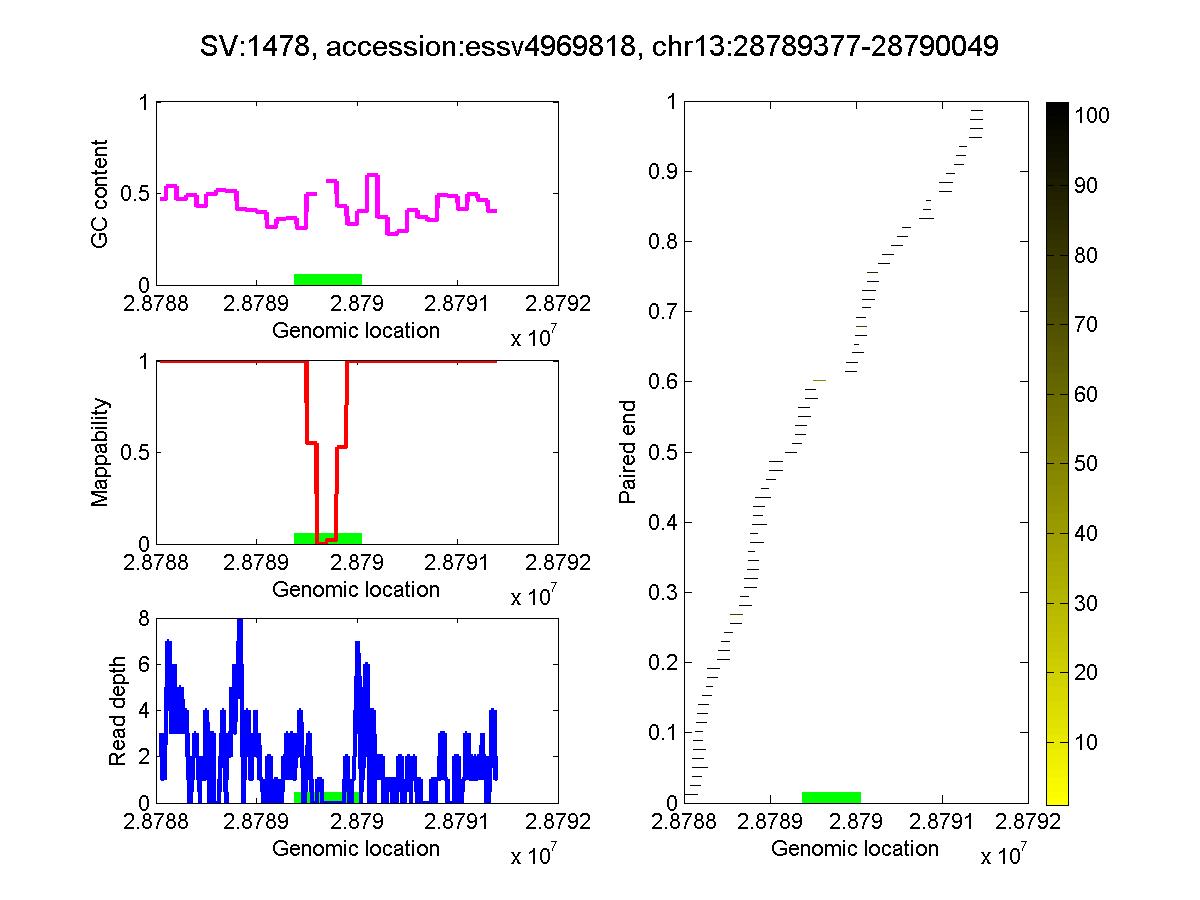

Supplement: Supplementary Materials — Supplementary data are available with this article at http://gr.xjtu.edu.cn/c/document_library/get_file?p_l_id=2403541&folderId=2539941&name=DLFE-115097.zip. Table S1 lists the complete information of suspicious variants and false positives, and the FIG directory contains the validation figures of each false positive. [file 8420547.f1.zip › 8420547.f1/FIG/SV1478.jpg]

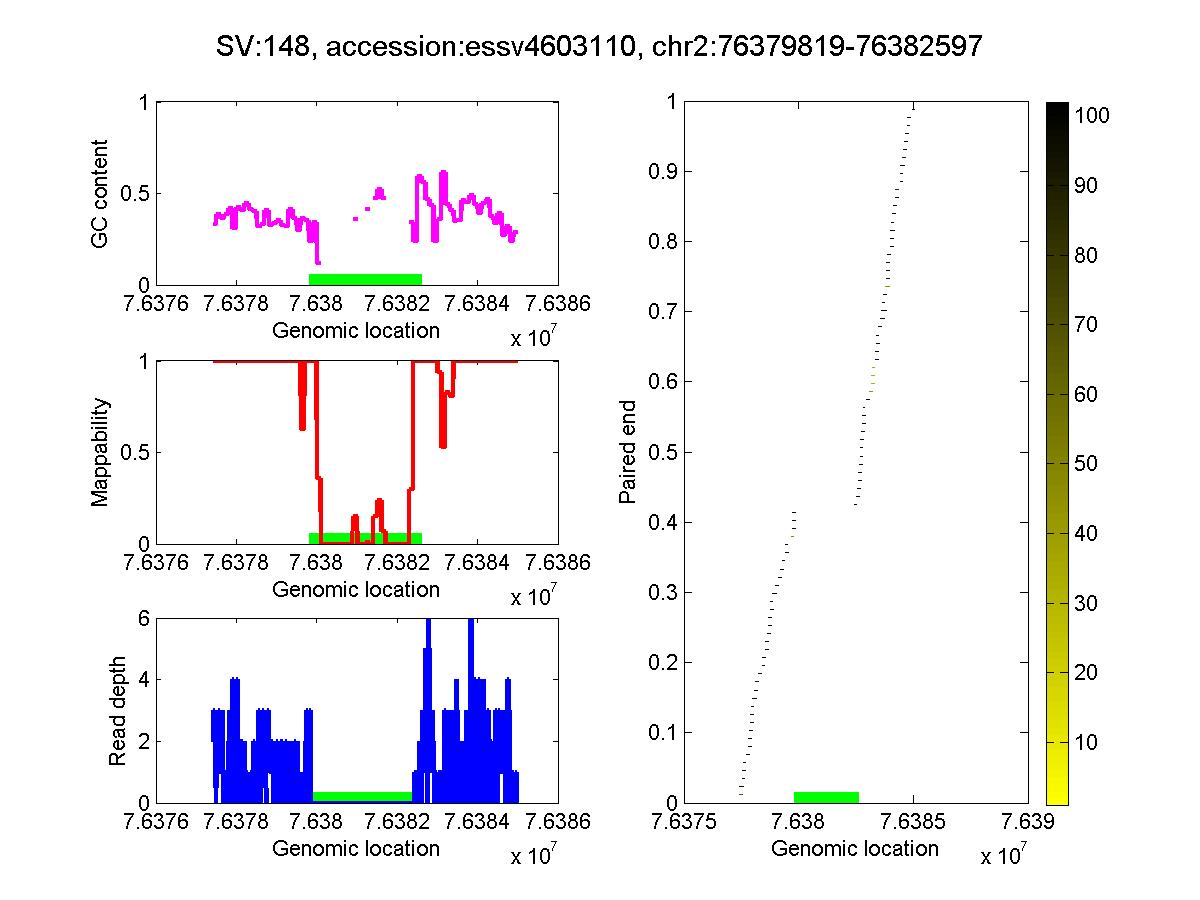

Supplement: Supplementary Materials — Supplementary data are available with this article at http://gr.xjtu.edu.cn/c/document_library/get_file?p_l_id=2403541&folderId=2539941&name=DLFE-115097.zip. Table S1 lists the complete information of suspicious variants and false positives, and the FIG directory contains the validation figures of each false positive. [file 8420547.f1.zip › 8420547.f1/FIG/SV148.jpg]

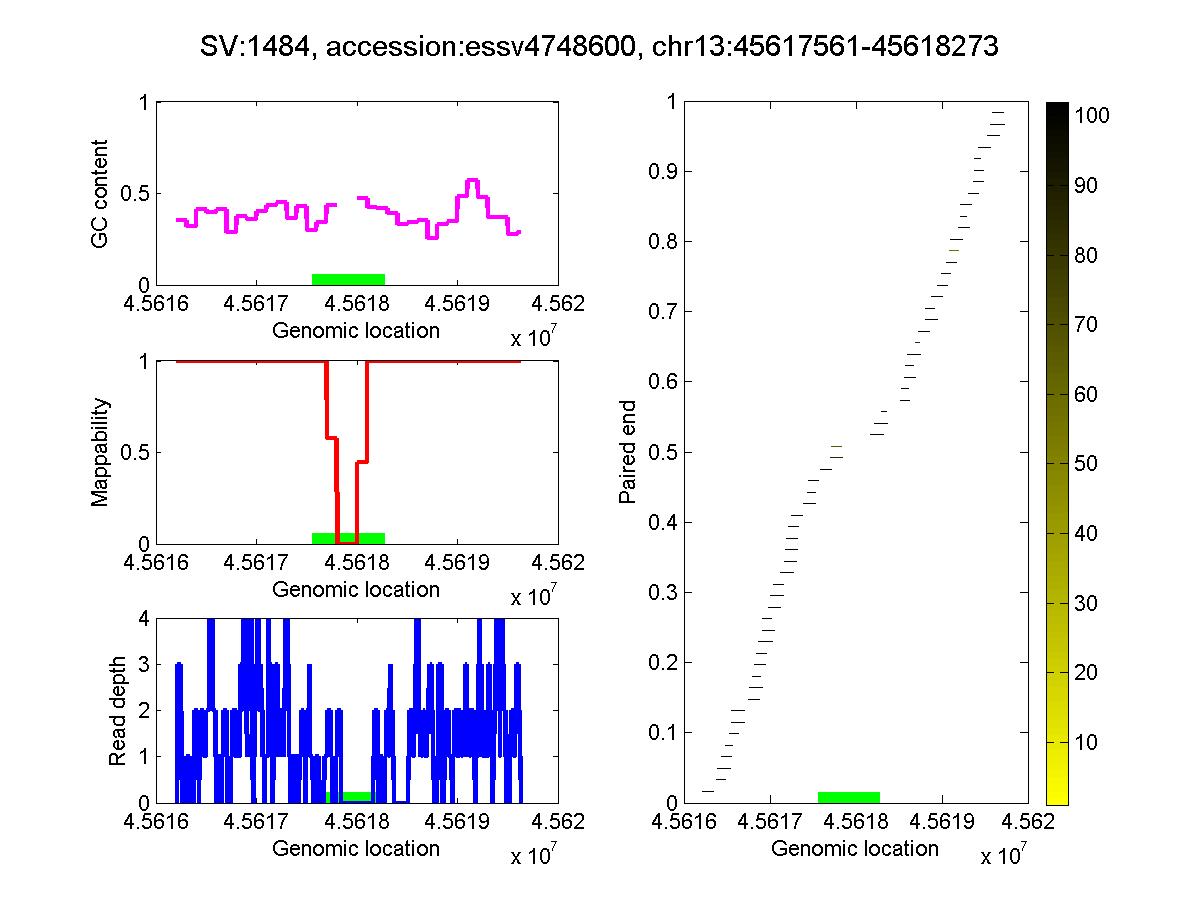

Supplement: Supplementary Materials — Supplementary data are available with this article at http://gr.xjtu.edu.cn/c/document_library/get_file?p_l_id=2403541&folderId=2539941&name=DLFE-115097.zip. Table S1 lists the complete information of suspicious variants and false positives, and the FIG directory contains the validation figures of each false positive. [file 8420547.f1.zip › 8420547.f1/FIG/SV1484.jpg]

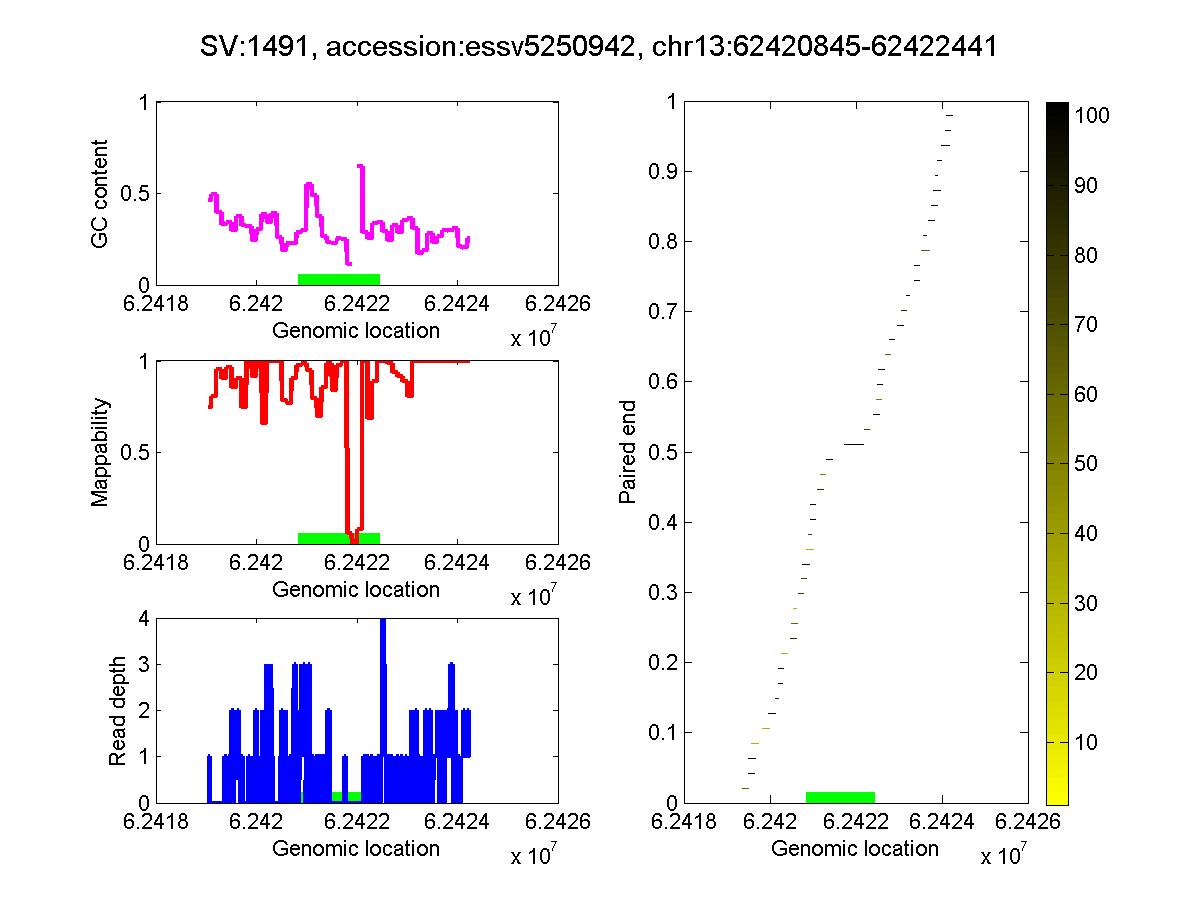

Supplement: Supplementary Materials — Supplementary data are available with this article at http://gr.xjtu.edu.cn/c/document_library/get_file?p_l_id=2403541&folderId=2539941&name=DLFE-115097.zip. Table S1 lists the complete information of suspicious variants and false positives, and the FIG directory contains the validation figures of each false positive. [file 8420547.f1.zip › 8420547.f1/FIG/SV1491.jpg]

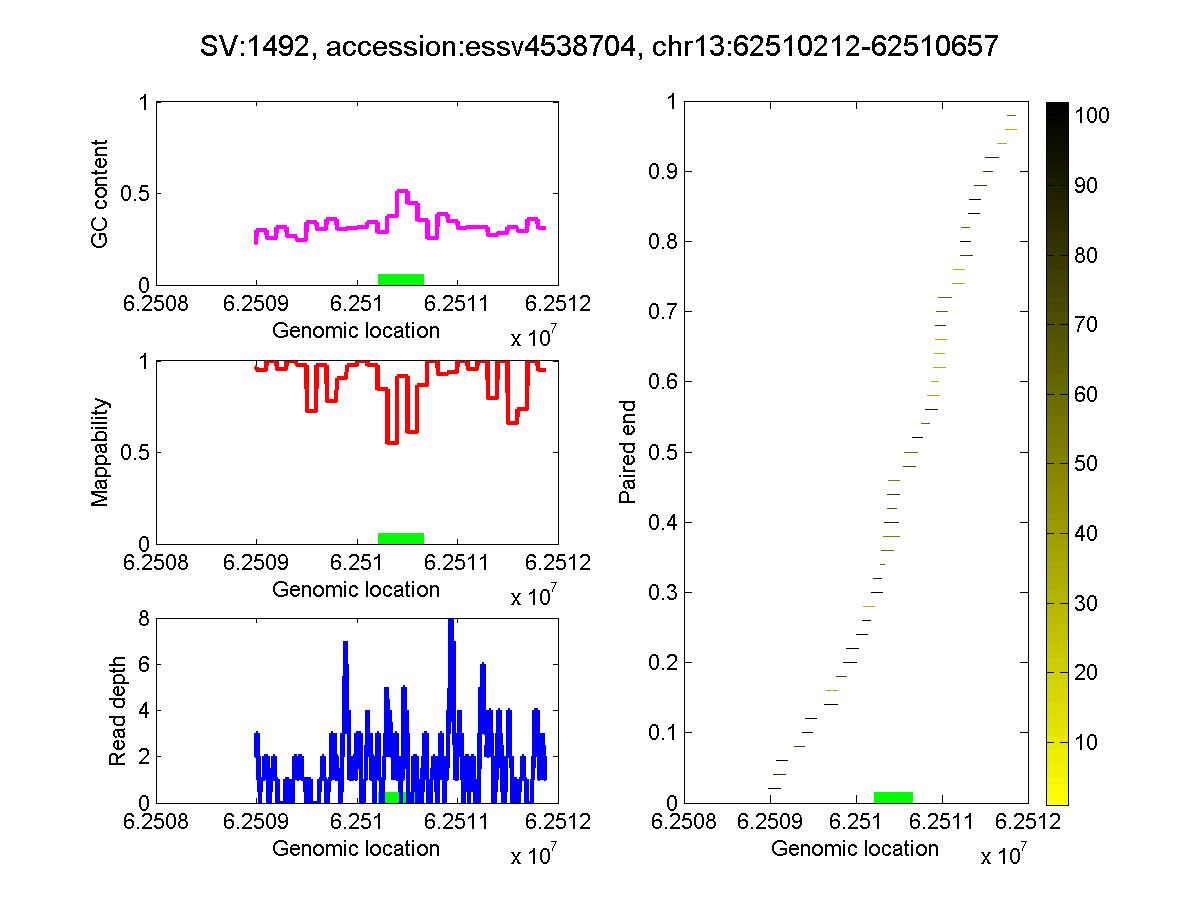

Supplement: Supplementary Materials — Supplementary data are available with this article at http://gr.xjtu.edu.cn/c/document_library/get_file?p_l_id=2403541&folderId=2539941&name=DLFE-115097.zip. Table S1 lists the complete information of suspicious variants and false positives, and the FIG directory contains the validation figures of each false positive. [file 8420547.f1.zip › 8420547.f1/FIG/SV1492.jpg]

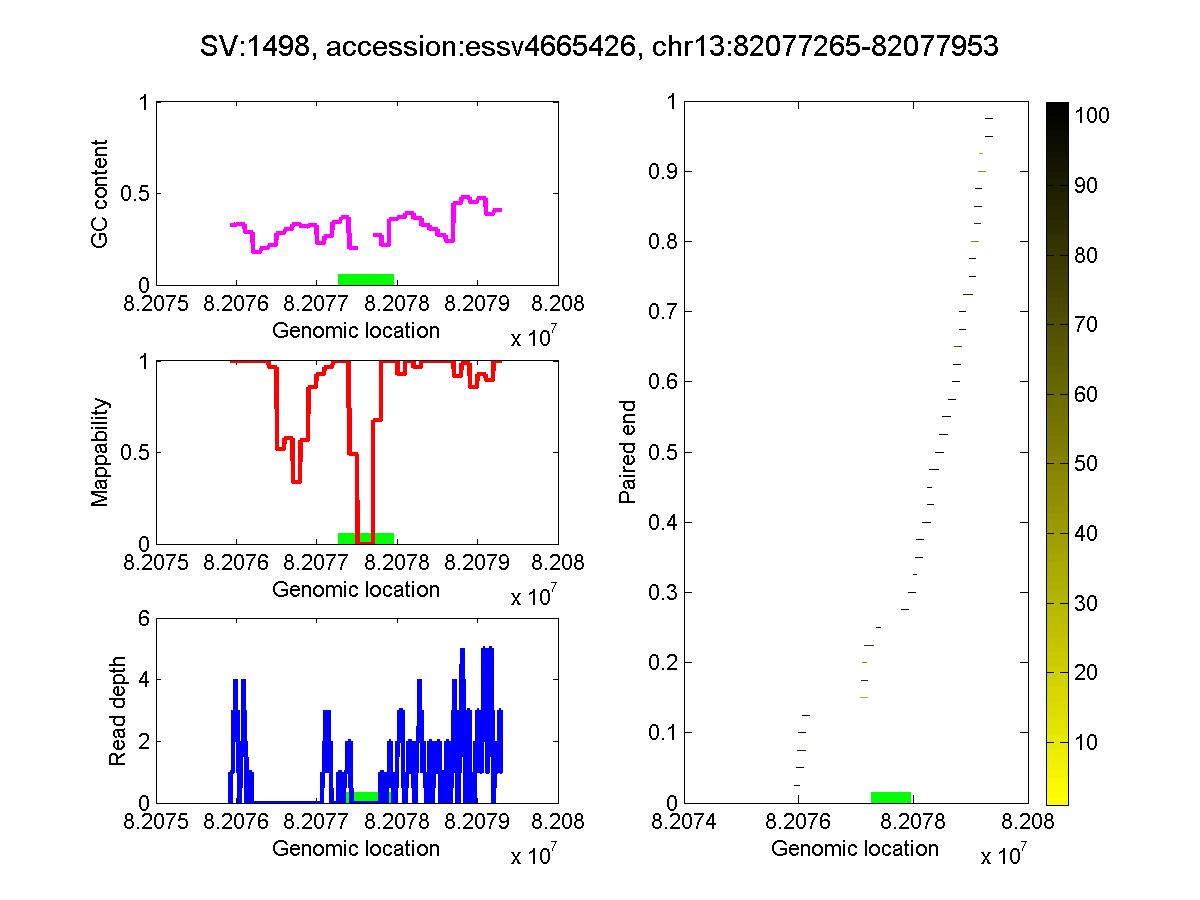

Supplement: Supplementary Materials — Supplementary data are available with this article at http://gr.xjtu.edu.cn/c/document_library/get_file?p_l_id=2403541&folderId=2539941&name=DLFE-115097.zip. Table S1 lists the complete information of suspicious variants and false positives, and the FIG directory contains the validation figures of each false positive. [file 8420547.f1.zip › 8420547.f1/FIG/SV1498.jpg]

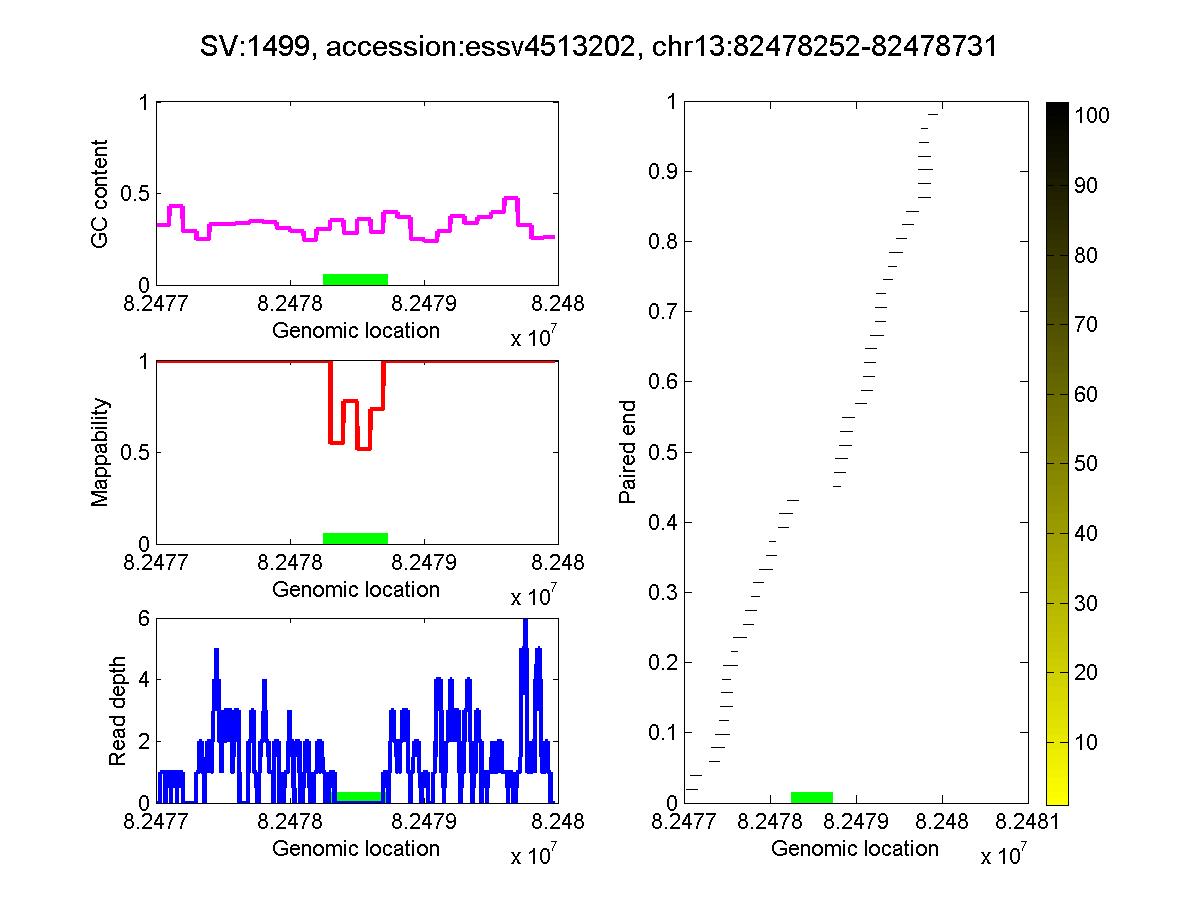

Supplement: Supplementary Materials — Supplementary data are available with this article at http://gr.xjtu.edu.cn/c/document_library/get_file?p_l_id=2403541&folderId=2539941&name=DLFE-115097.zip. Table S1 lists the complete information of suspicious variants and false positives, and the FIG directory contains the validation figures of each false positive. [file 8420547.f1.zip › 8420547.f1/FIG/SV1499.jpg]

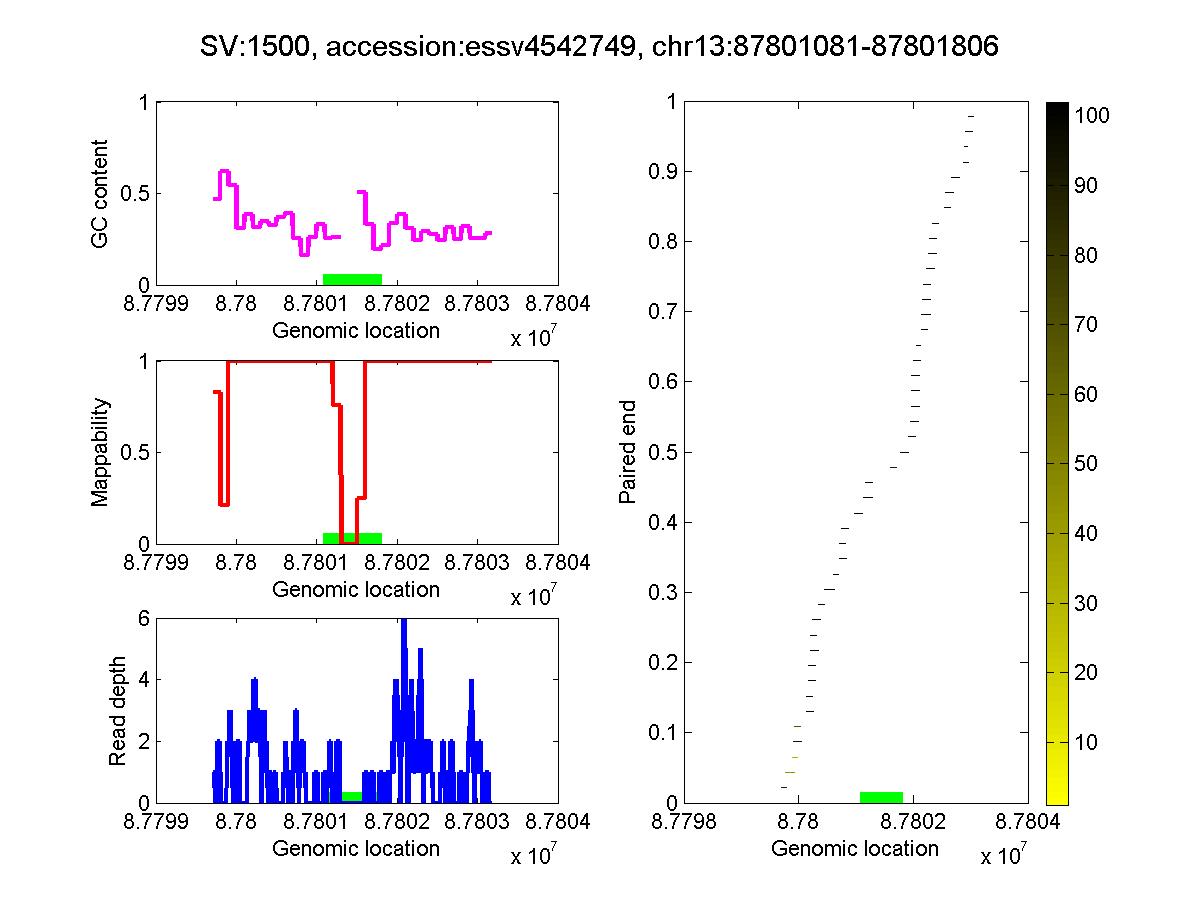

Supplement: Supplementary Materials — Supplementary data are available with this article at http://gr.xjtu.edu.cn/c/document_library/get_file?p_l_id=2403541&folderId=2539941&name=DLFE-115097.zip. Table S1 lists the complete information of suspicious variants and false positives, and the FIG directory contains the validation figures of each false positive. [file 8420547.f1.zip › 8420547.f1/FIG/SV1500.jpg]

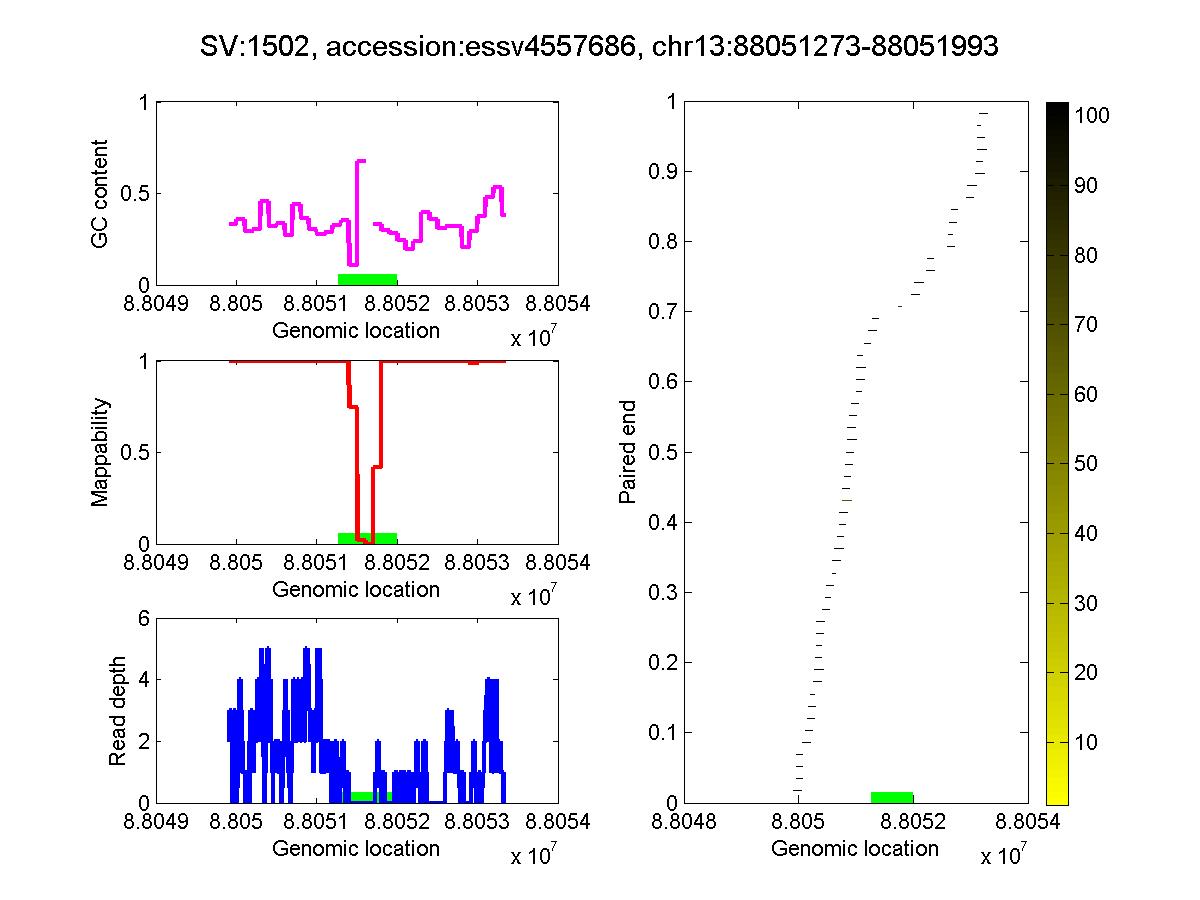

Supplement: Supplementary Materials — Supplementary data are available with this article at http://gr.xjtu.edu.cn/c/document_library/get_file?p_l_id=2403541&folderId=2539941&name=DLFE-115097.zip. Table S1 lists the complete information of suspicious variants and false positives, and the FIG directory contains the validation figures of each false positive. [file 8420547.f1.zip › 8420547.f1/FIG/SV1502.jpg]

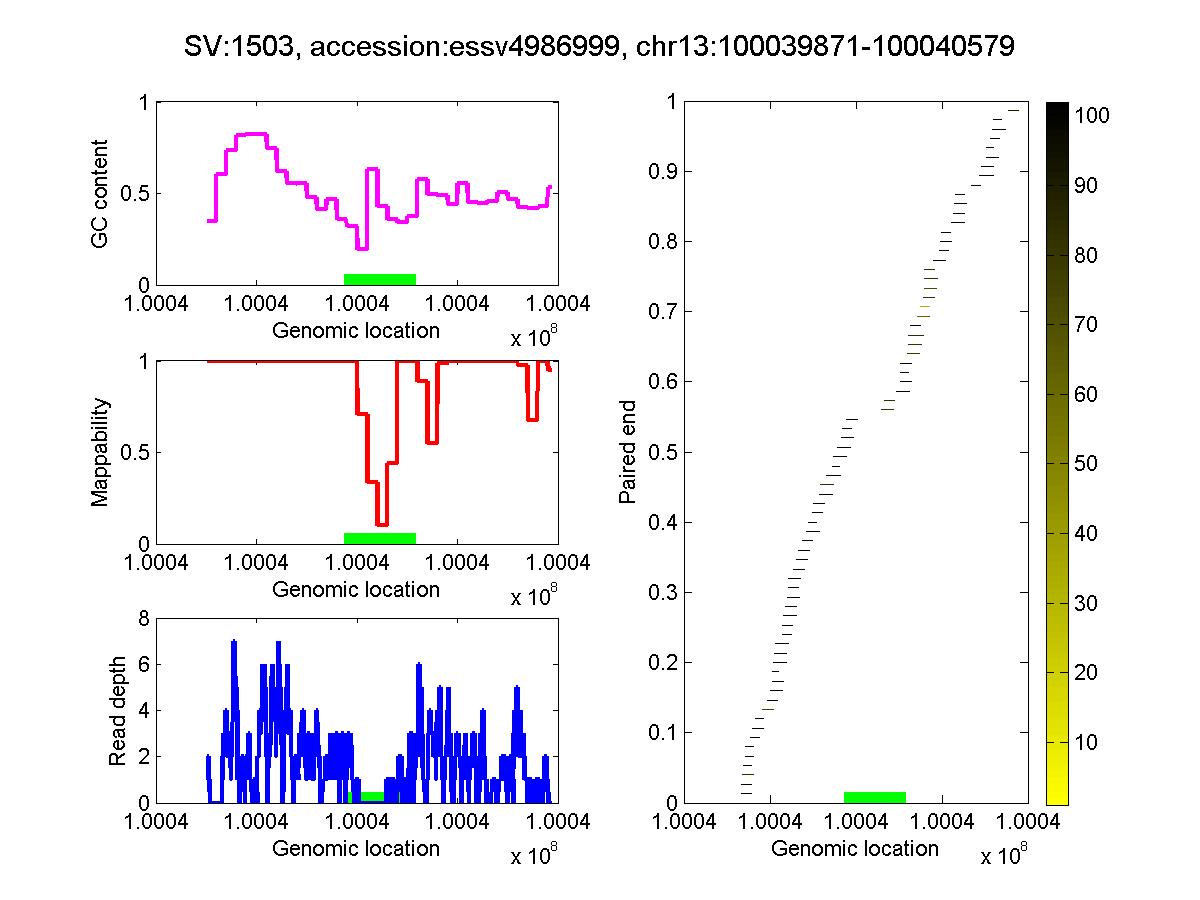

Supplement: Supplementary Materials — Supplementary data are available with this article at http://gr.xjtu.edu.cn/c/document_library/get_file?p_l_id=2403541&folderId=2539941&name=DLFE-115097.zip. Table S1 lists the complete information of suspicious variants and false positives, and the FIG directory contains the validation figures of each false positive. [file 8420547.f1.zip › 8420547.f1/FIG/SV1503.jpg]

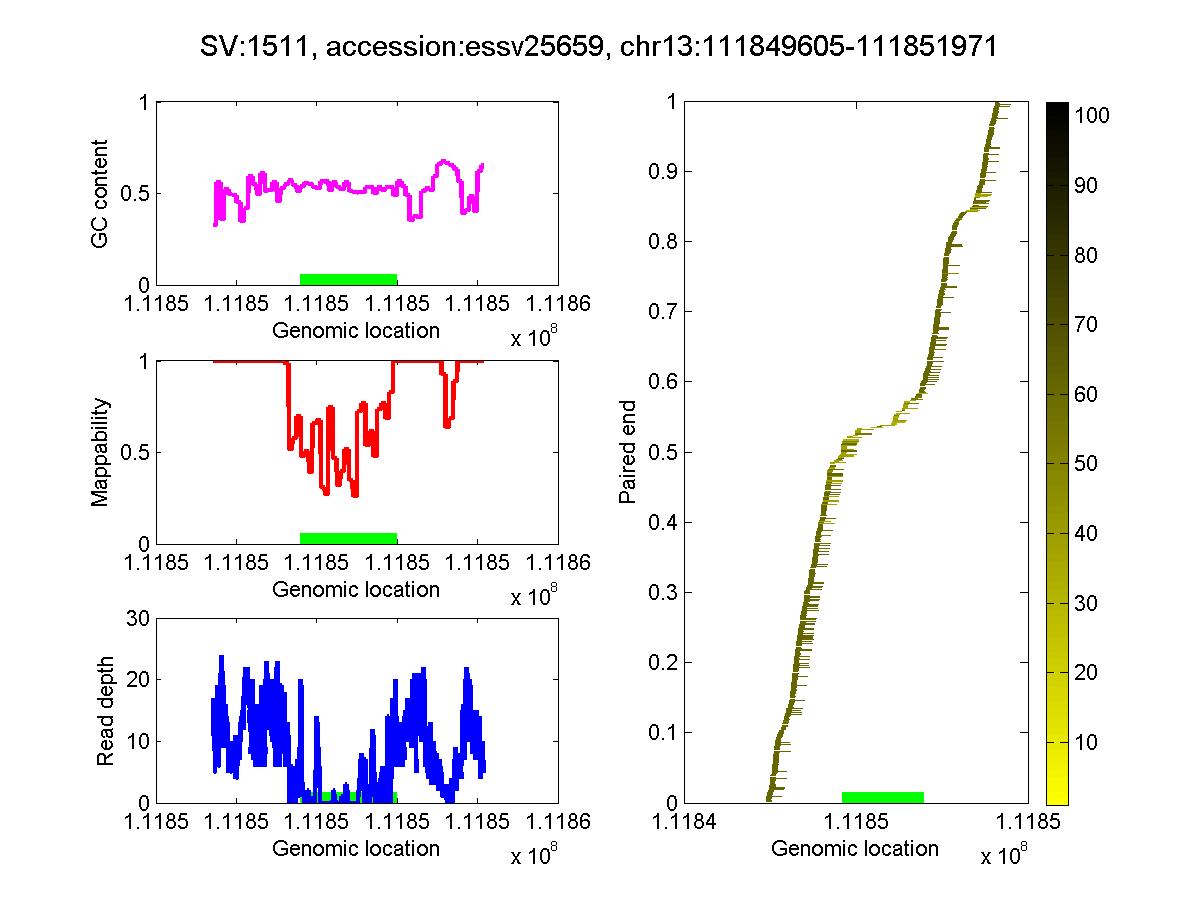

Supplement: Supplementary Materials — Supplementary data are available with this article at http://gr.xjtu.edu.cn/c/document_library/get_file?p_l_id=2403541&folderId=2539941&name=DLFE-115097.zip. Table S1 lists the complete information of suspicious variants and false positives, and the FIG directory contains the validation figures of each false positive. [file 8420547.f1.zip › 8420547.f1/FIG/SV1511.jpg]

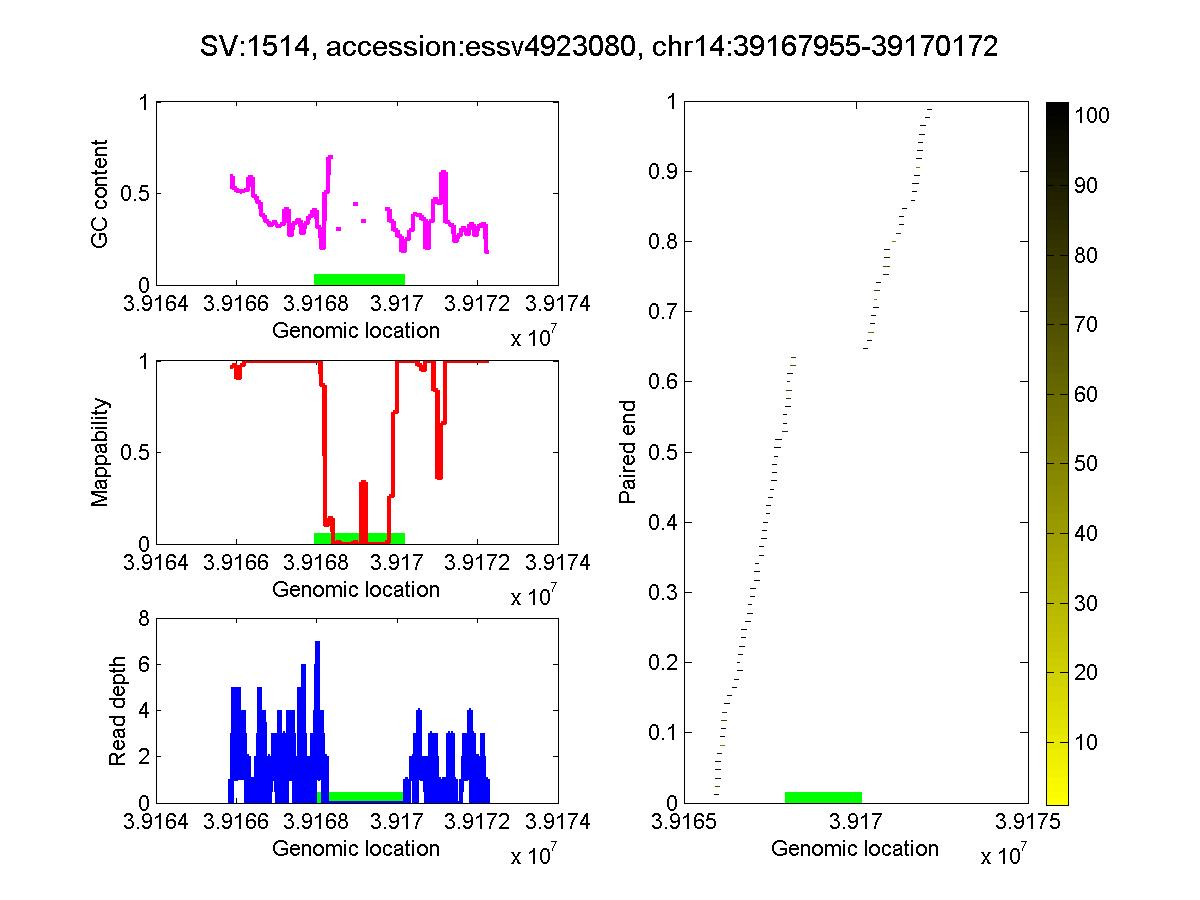

Supplement: Supplementary Materials — Supplementary data are available with this article at http://gr.xjtu.edu.cn/c/document_library/get_file?p_l_id=2403541&folderId=2539941&name=DLFE-115097.zip. Table S1 lists the complete information of suspicious variants and false positives, and the FIG directory contains the validation figures of each false positive. [file 8420547.f1.zip › 8420547.f1/FIG/SV1514.jpg]

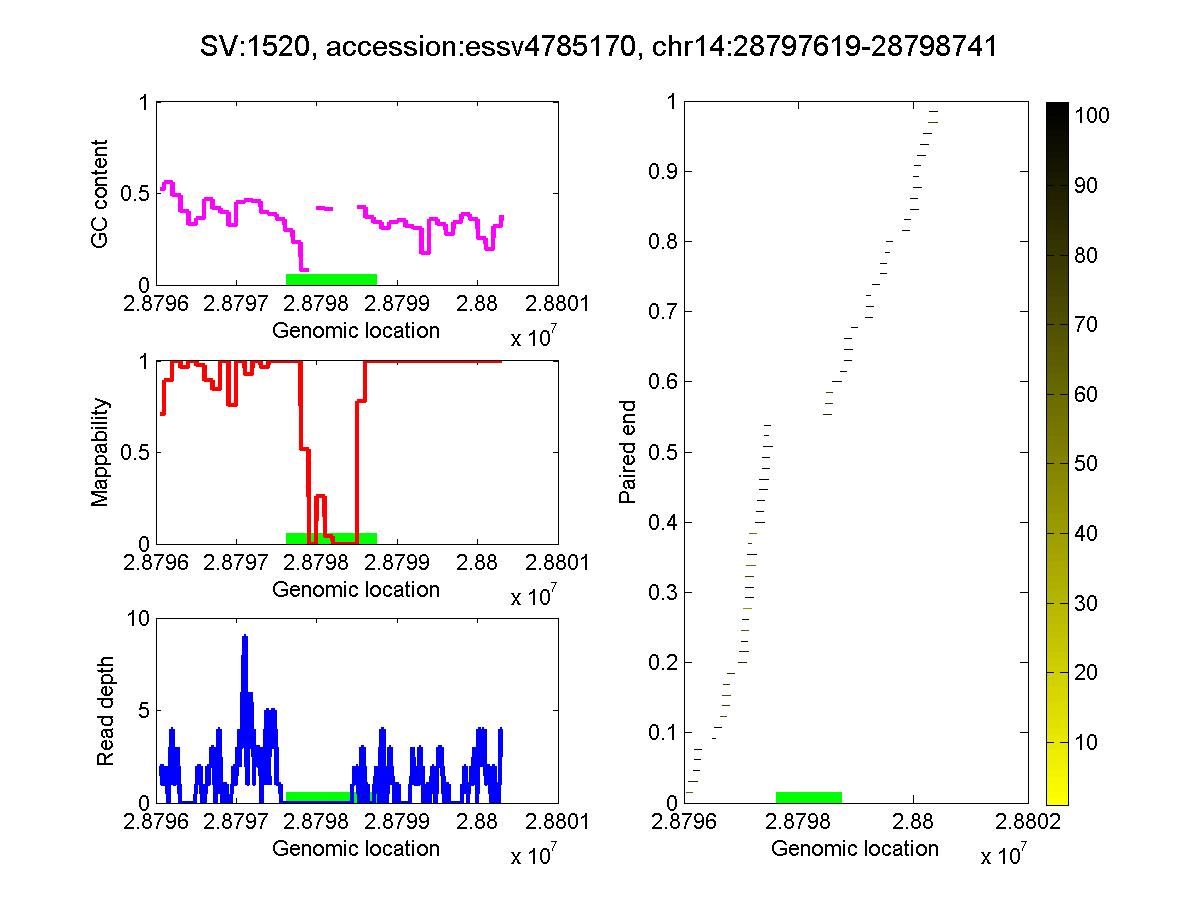

Supplement: Supplementary Materials — Supplementary data are available with this article at http://gr.xjtu.edu.cn/c/document_library/get_file?p_l_id=2403541&folderId=2539941&name=DLFE-115097.zip. Table S1 lists the complete information of suspicious variants and false positives, and the FIG directory contains the validation figures of each false positive. [file 8420547.f1.zip › 8420547.f1/FIG/SV1520.jpg]

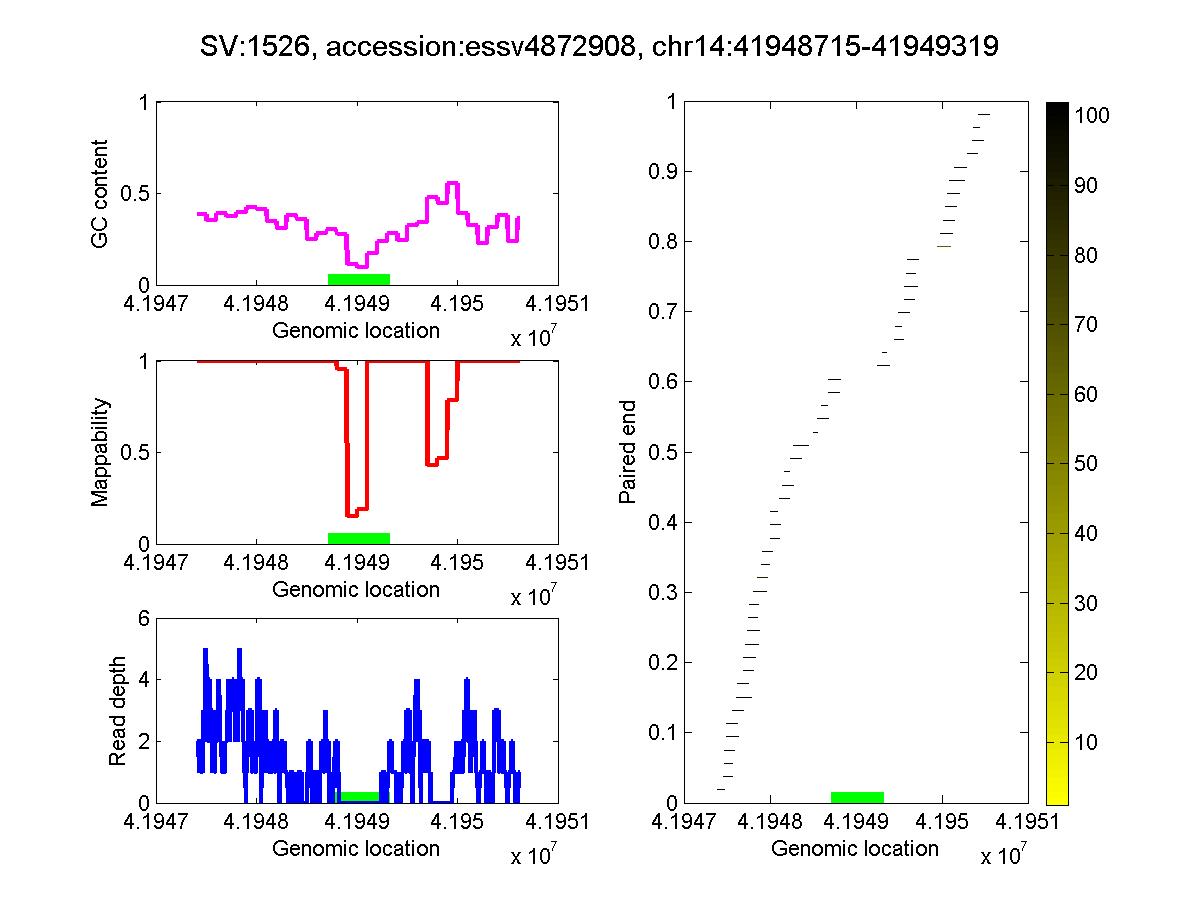

Supplement: Supplementary Materials — Supplementary data are available with this article at http://gr.xjtu.edu.cn/c/document_library/get_file?p_l_id=2403541&folderId=2539941&name=DLFE-115097.zip. Table S1 lists the complete information of suspicious variants and false positives, and the FIG directory contains the validation figures of each false positive. [file 8420547.f1.zip › 8420547.f1/FIG/SV1526.jpg]

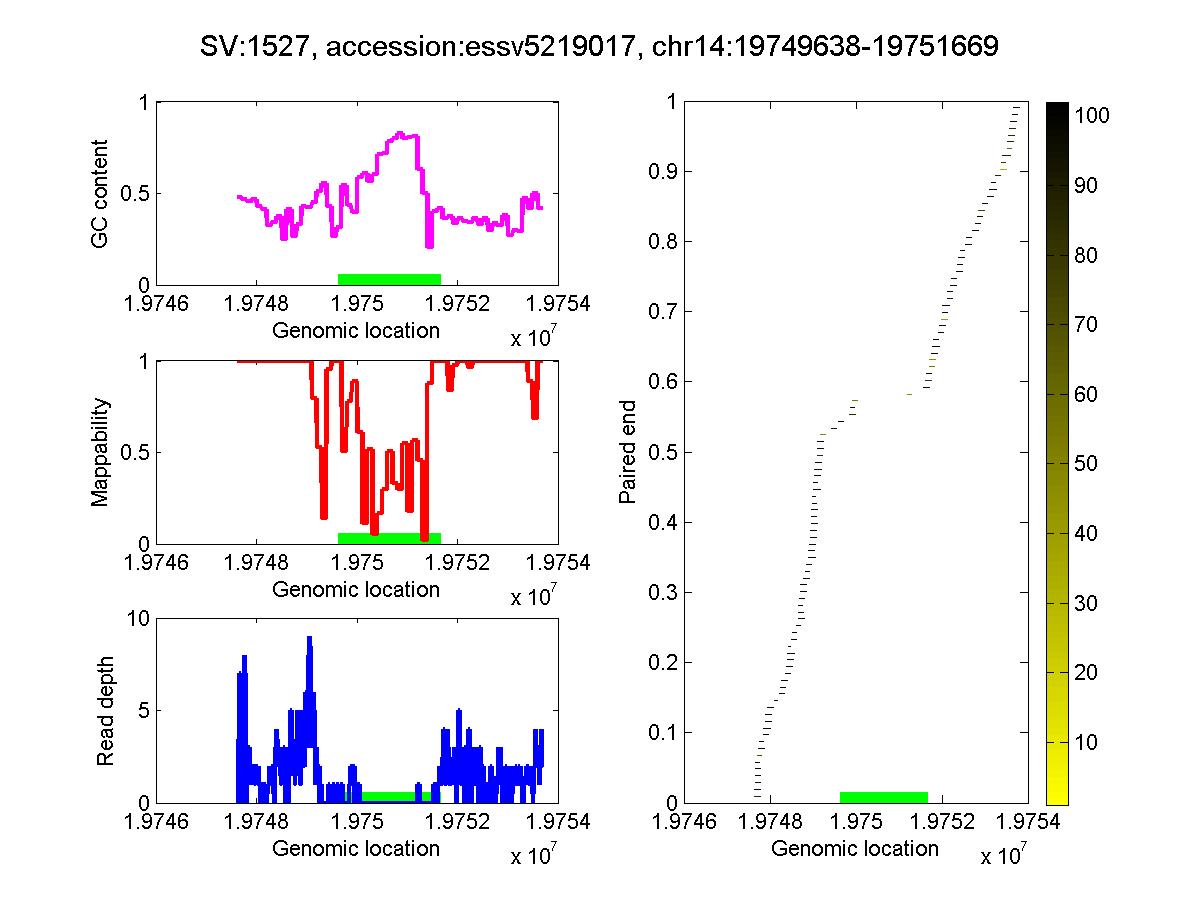

Supplement: Supplementary Materials — Supplementary data are available with this article at http://gr.xjtu.edu.cn/c/document_library/get_file?p_l_id=2403541&folderId=2539941&name=DLFE-115097.zip. Table S1 lists the complete information of suspicious variants and false positives, and the FIG directory contains the validation figures of each false positive. [file 8420547.f1.zip › 8420547.f1/FIG/SV1527.jpg]

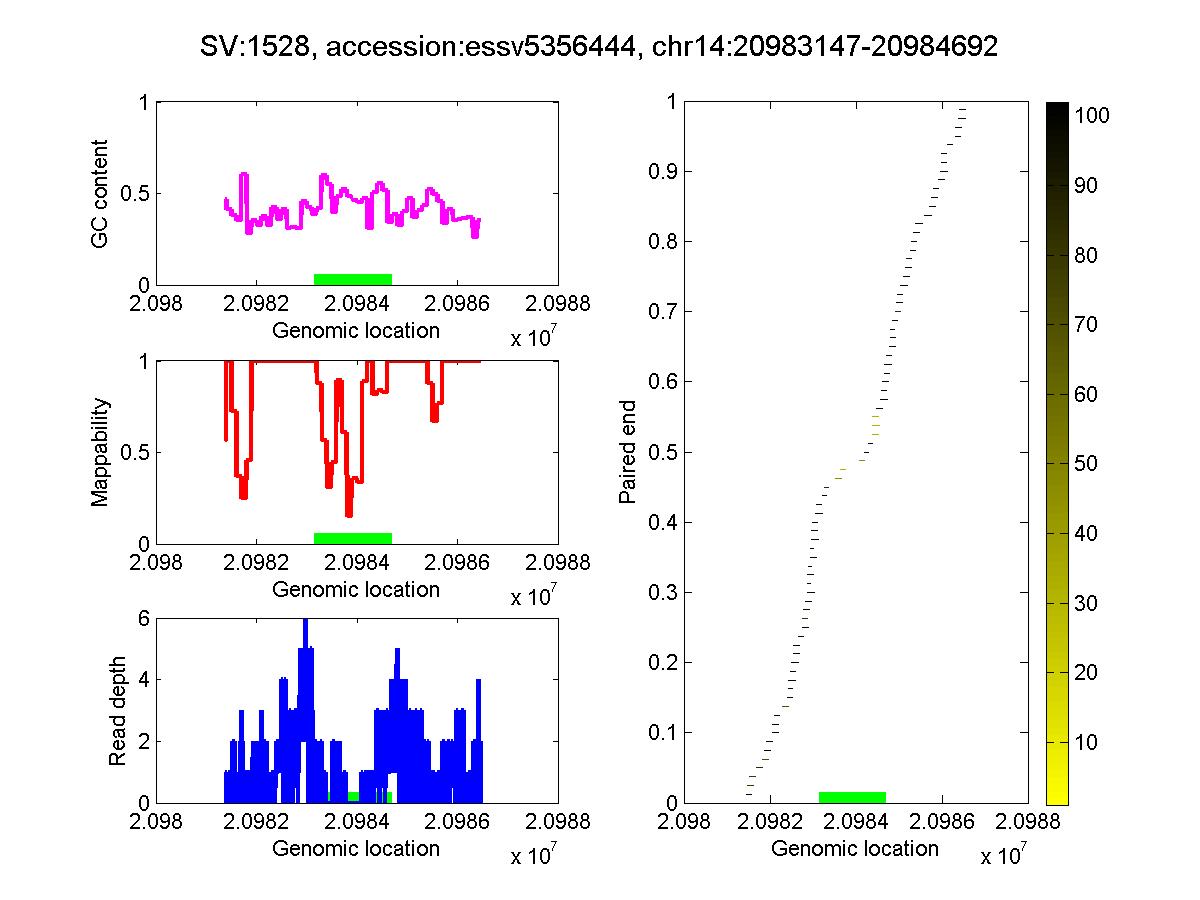

Supplement: Supplementary Materials — Supplementary data are available with this article at http://gr.xjtu.edu.cn/c/document_library/get_file?p_l_id=2403541&folderId=2539941&name=DLFE-115097.zip. Table S1 lists the complete information of suspicious variants and false positives, and the FIG directory contains the validation figures of each false positive. [file 8420547.f1.zip › 8420547.f1/FIG/SV1528.jpg]

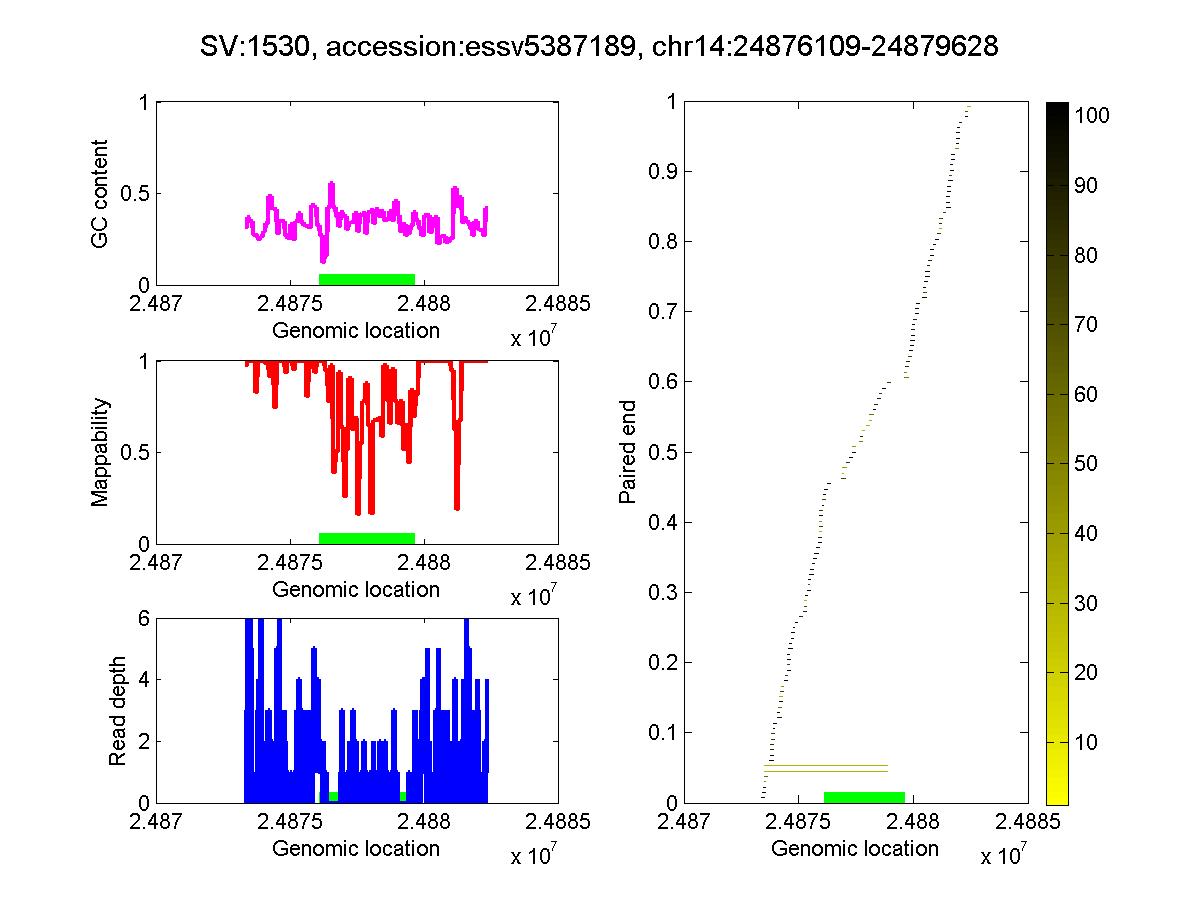

Supplement: Supplementary Materials — Supplementary data are available with this article at http://gr.xjtu.edu.cn/c/document_library/get_file?p_l_id=2403541&folderId=2539941&name=DLFE-115097.zip. Table S1 lists the complete information of suspicious variants and false positives, and the FIG directory contains the validation figures of each false positive. [file 8420547.f1.zip › 8420547.f1/FIG/SV1530.jpg]

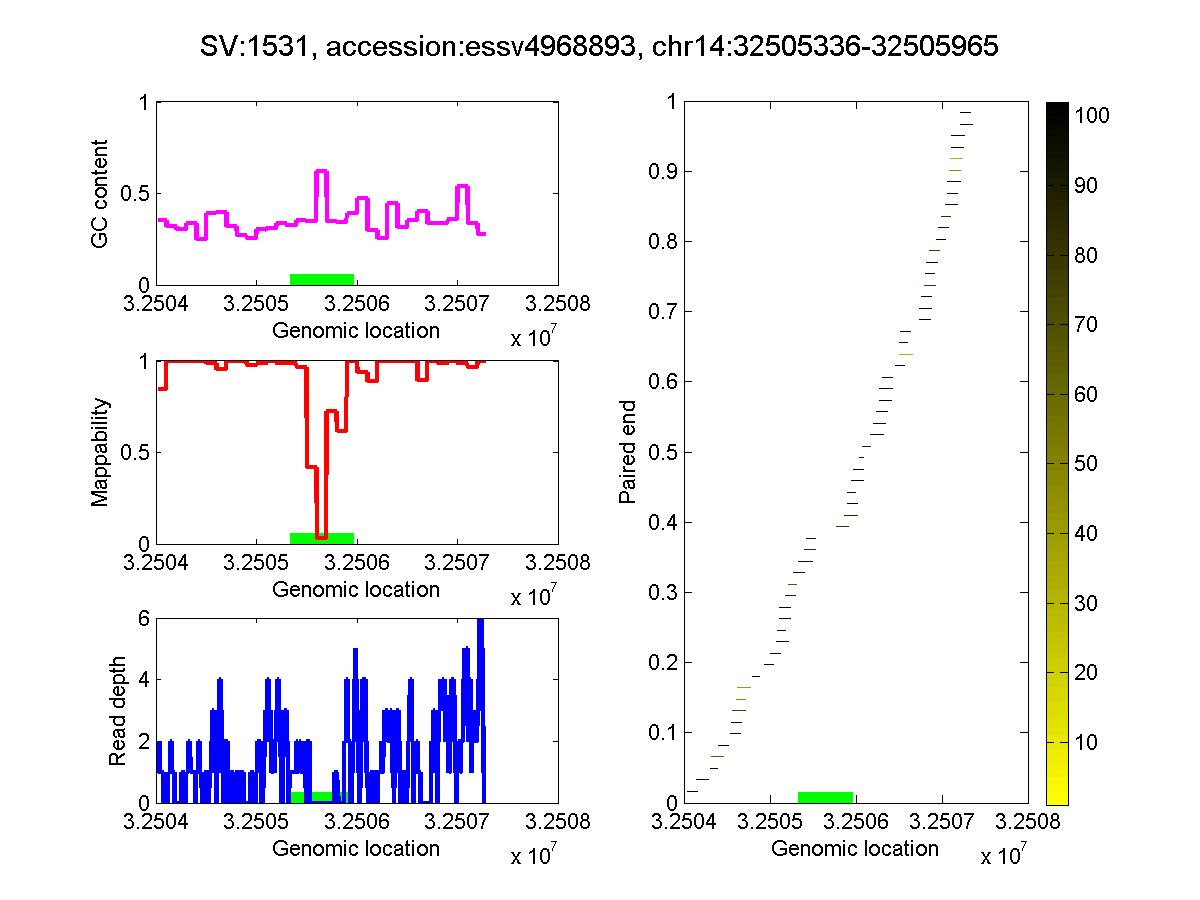

Supplement: Supplementary Materials — Supplementary data are available with this article at http://gr.xjtu.edu.cn/c/document_library/get_file?p_l_id=2403541&folderId=2539941&name=DLFE-115097.zip. Table S1 lists the complete information of suspicious variants and false positives, and the FIG directory contains the validation figures of each false positive. [file 8420547.f1.zip › 8420547.f1/FIG/SV1531.jpg]

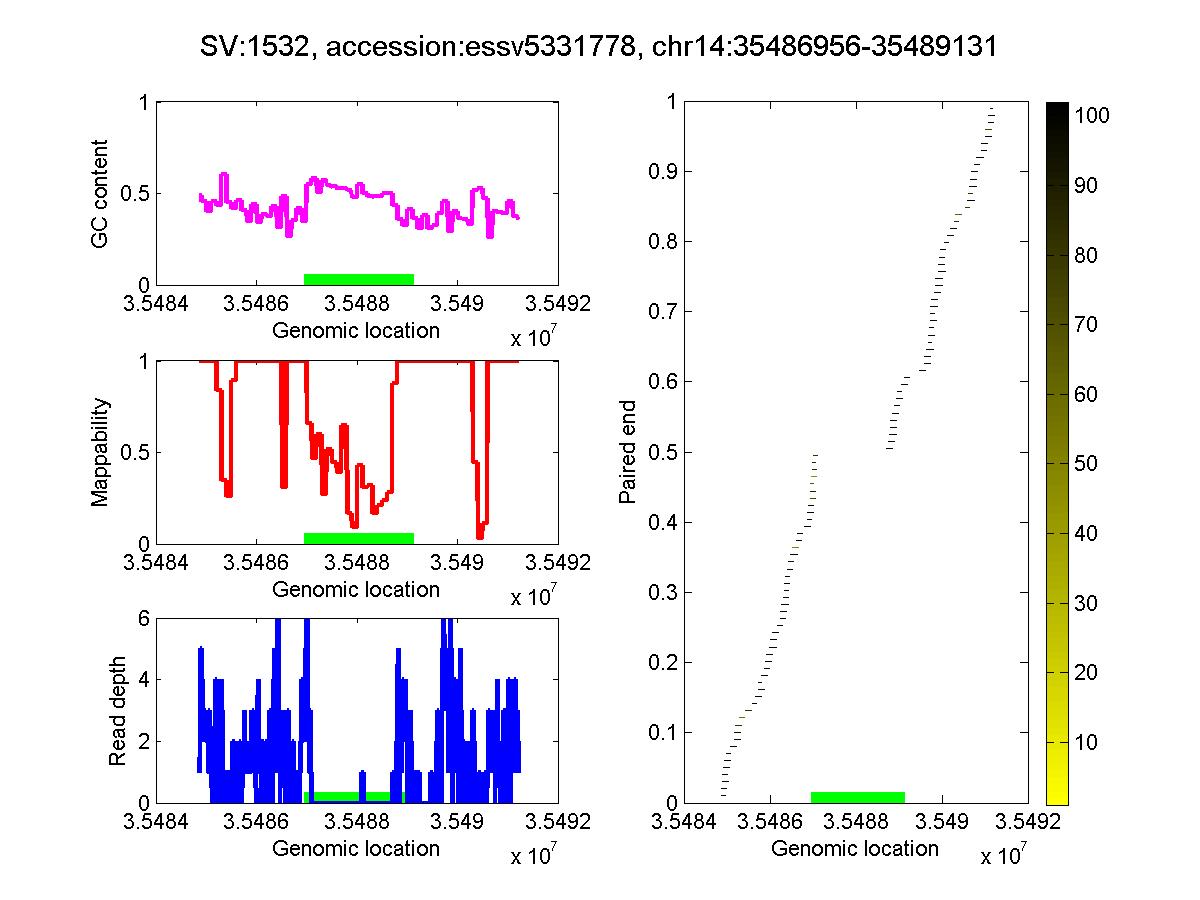

Supplement: Supplementary Materials — Supplementary data are available with this article at http://gr.xjtu.edu.cn/c/document_library/get_file?p_l_id=2403541&folderId=2539941&name=DLFE-115097.zip. Table S1 lists the complete information of suspicious variants and false positives, and the FIG directory contains the validation figures of each false positive. [file 8420547.f1.zip › 8420547.f1/FIG/SV1532.jpg]

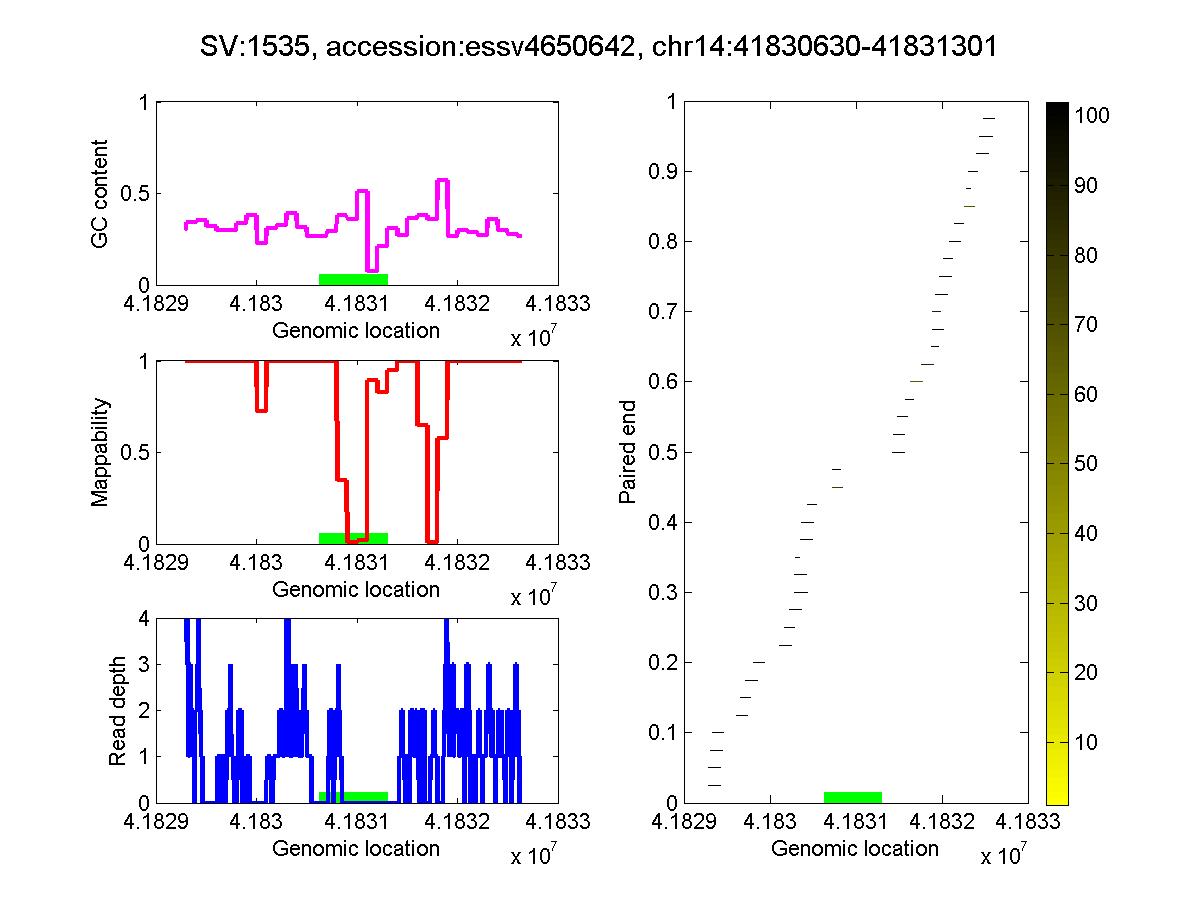

Supplement: Supplementary Materials — Supplementary data are available with this article at http://gr.xjtu.edu.cn/c/document_library/get_file?p_l_id=2403541&folderId=2539941&name=DLFE-115097.zip. Table S1 lists the complete information of suspicious variants and false positives, and the FIG directory contains the validation figures of each false positive. [file 8420547.f1.zip › 8420547.f1/FIG/SV1535.jpg]

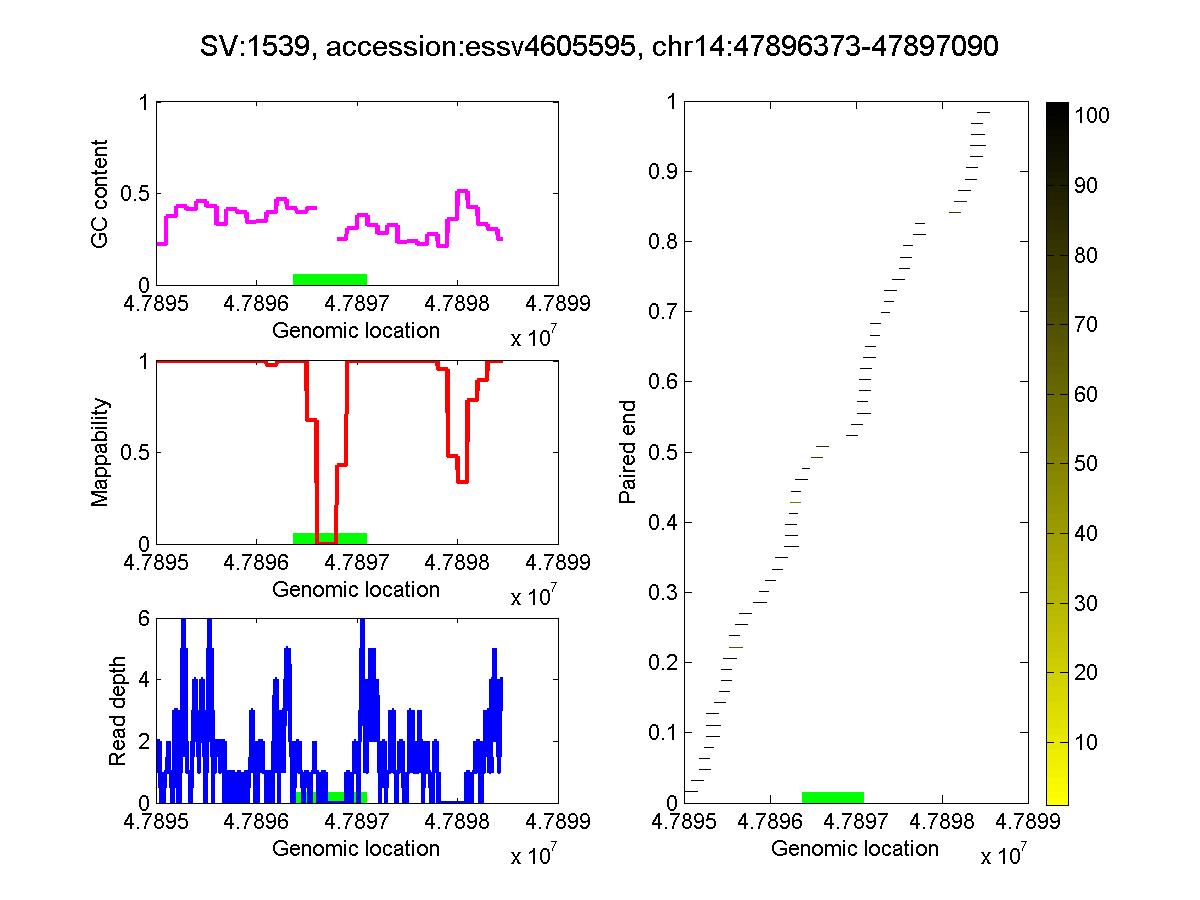

Supplement: Supplementary Materials — Supplementary data are available with this article at http://gr.xjtu.edu.cn/c/document_library/get_file?p_l_id=2403541&folderId=2539941&name=DLFE-115097.zip. Table S1 lists the complete information of suspicious variants and false positives, and the FIG directory contains the validation figures of each false positive. [file 8420547.f1.zip › 8420547.f1/FIG/SV1539.jpg]

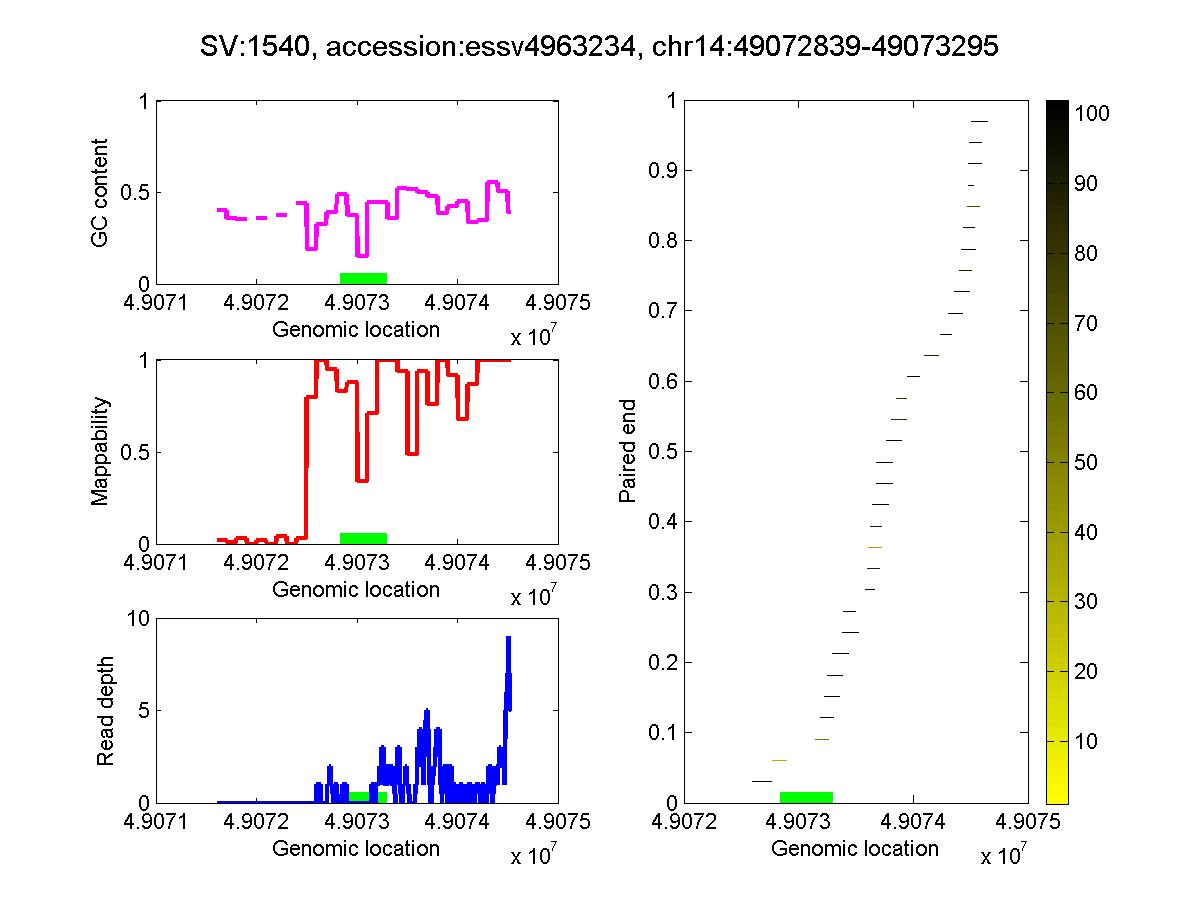

Supplement: Supplementary Materials — Supplementary data are available with this article at http://gr.xjtu.edu.cn/c/document_library/get_file?p_l_id=2403541&folderId=2539941&name=DLFE-115097.zip. Table S1 lists the complete information of suspicious variants and false positives, and the FIG directory contains the validation figures of each false positive. [file 8420547.f1.zip › 8420547.f1/FIG/SV1540.jpg]

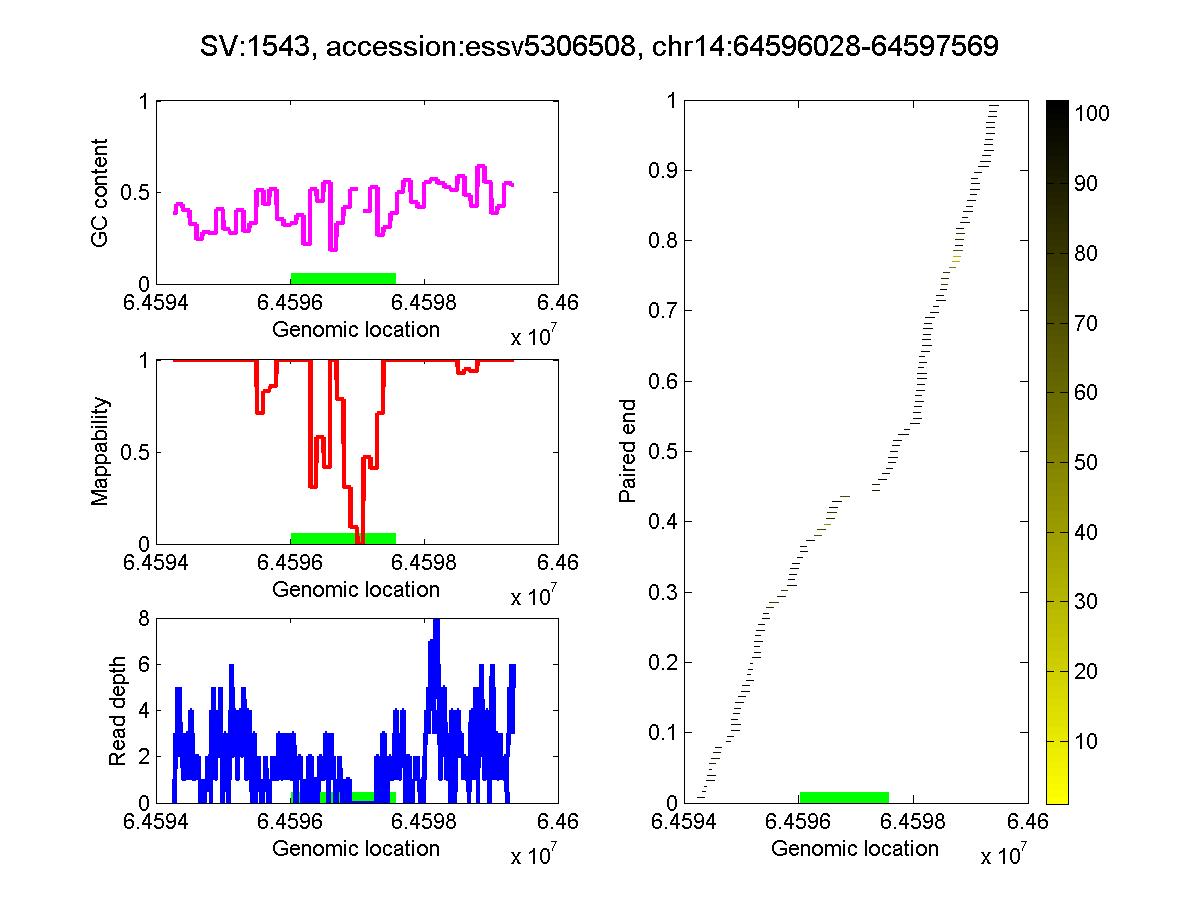

Supplement: Supplementary Materials — Supplementary data are available with this article at http://gr.xjtu.edu.cn/c/document_library/get_file?p_l_id=2403541&folderId=2539941&name=DLFE-115097.zip. Table S1 lists the complete information of suspicious variants and false positives, and the FIG directory contains the validation figures of each false positive. [file 8420547.f1.zip › 8420547.f1/FIG/SV1543.jpg]

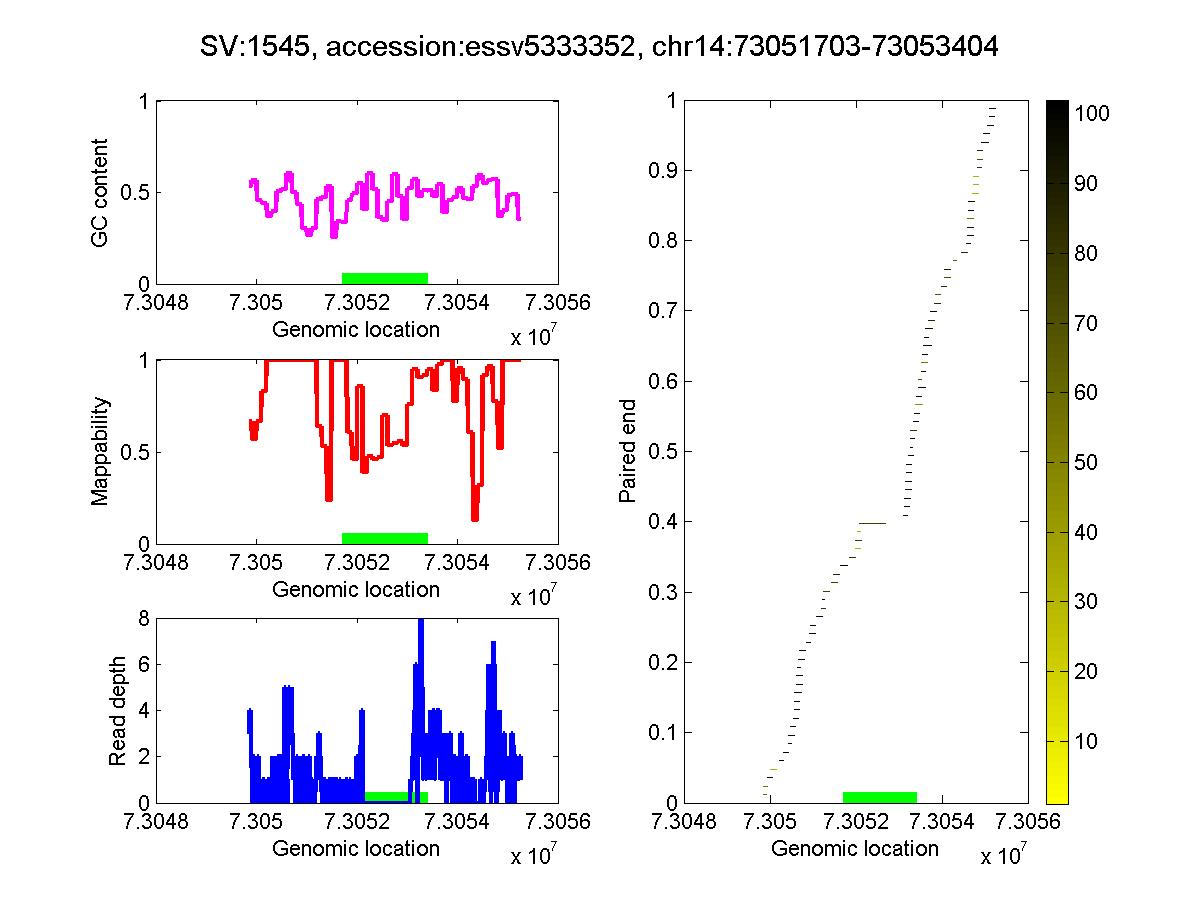

Supplement: Supplementary Materials — Supplementary data are available with this article at http://gr.xjtu.edu.cn/c/document_library/get_file?p_l_id=2403541&folderId=2539941&name=DLFE-115097.zip. Table S1 lists the complete information of suspicious variants and false positives, and the FIG directory contains the validation figures of each false positive. [file 8420547.f1.zip › 8420547.f1/FIG/SV1545.jpg]

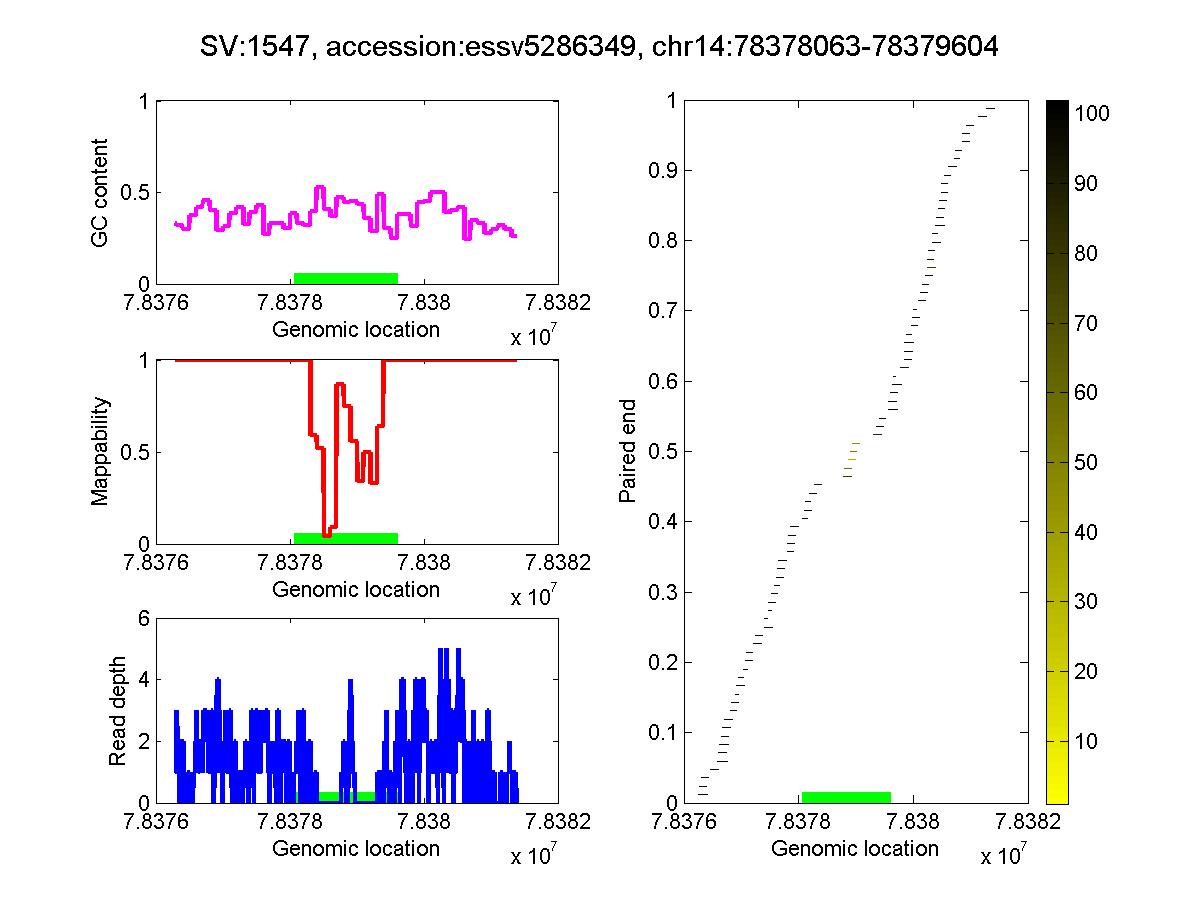

Supplement: Supplementary Materials — Supplementary data are available with this article at http://gr.xjtu.edu.cn/c/document_library/get_file?p_l_id=2403541&folderId=2539941&name=DLFE-115097.zip. Table S1 lists the complete information of suspicious variants and false positives, and the FIG directory contains the validation figures of each false positive. [file 8420547.f1.zip › 8420547.f1/FIG/SV1547.jpg]

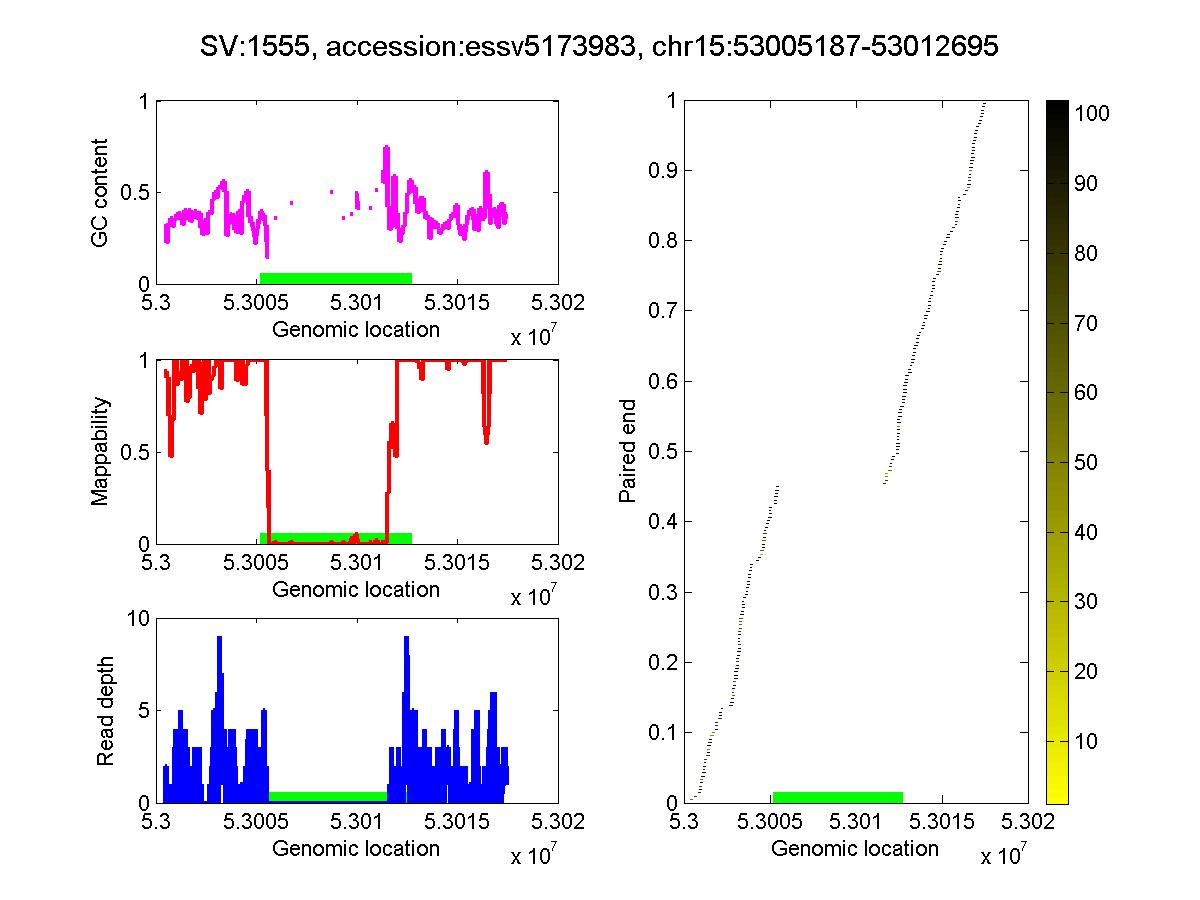

Supplement: Supplementary Materials — Supplementary data are available with this article at http://gr.xjtu.edu.cn/c/document_library/get_file?p_l_id=2403541&folderId=2539941&name=DLFE-115097.zip. Table S1 lists the complete information of suspicious variants and false positives, and the FIG directory contains the validation figures of each false positive. [file 8420547.f1.zip › 8420547.f1/FIG/SV1555.jpg]

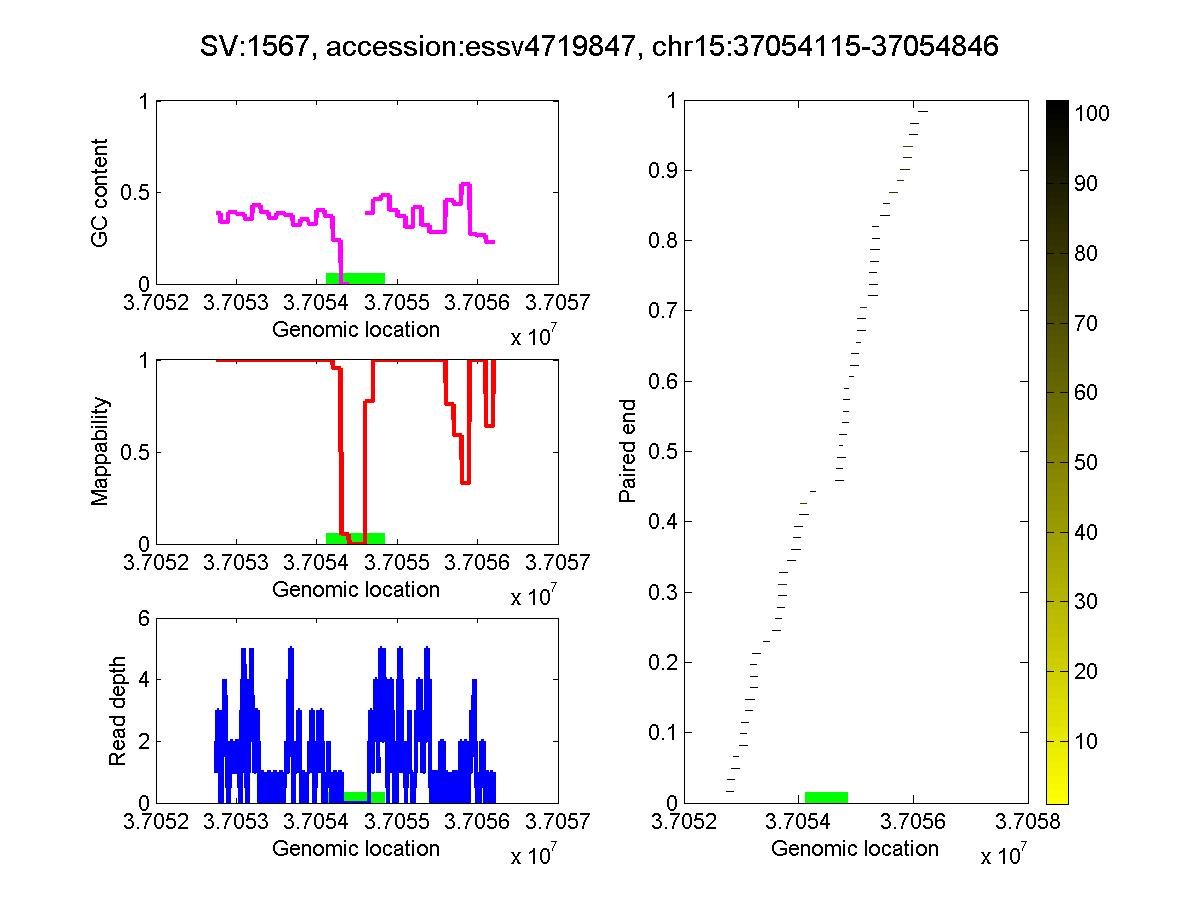

Supplement: Supplementary Materials — Supplementary data are available with this article at http://gr.xjtu.edu.cn/c/document_library/get_file?p_l_id=2403541&folderId=2539941&name=DLFE-115097.zip. Table S1 lists the complete information of suspicious variants and false positives, and the FIG directory contains the validation figures of each false positive. [file 8420547.f1.zip › 8420547.f1/FIG/SV1567.jpg]

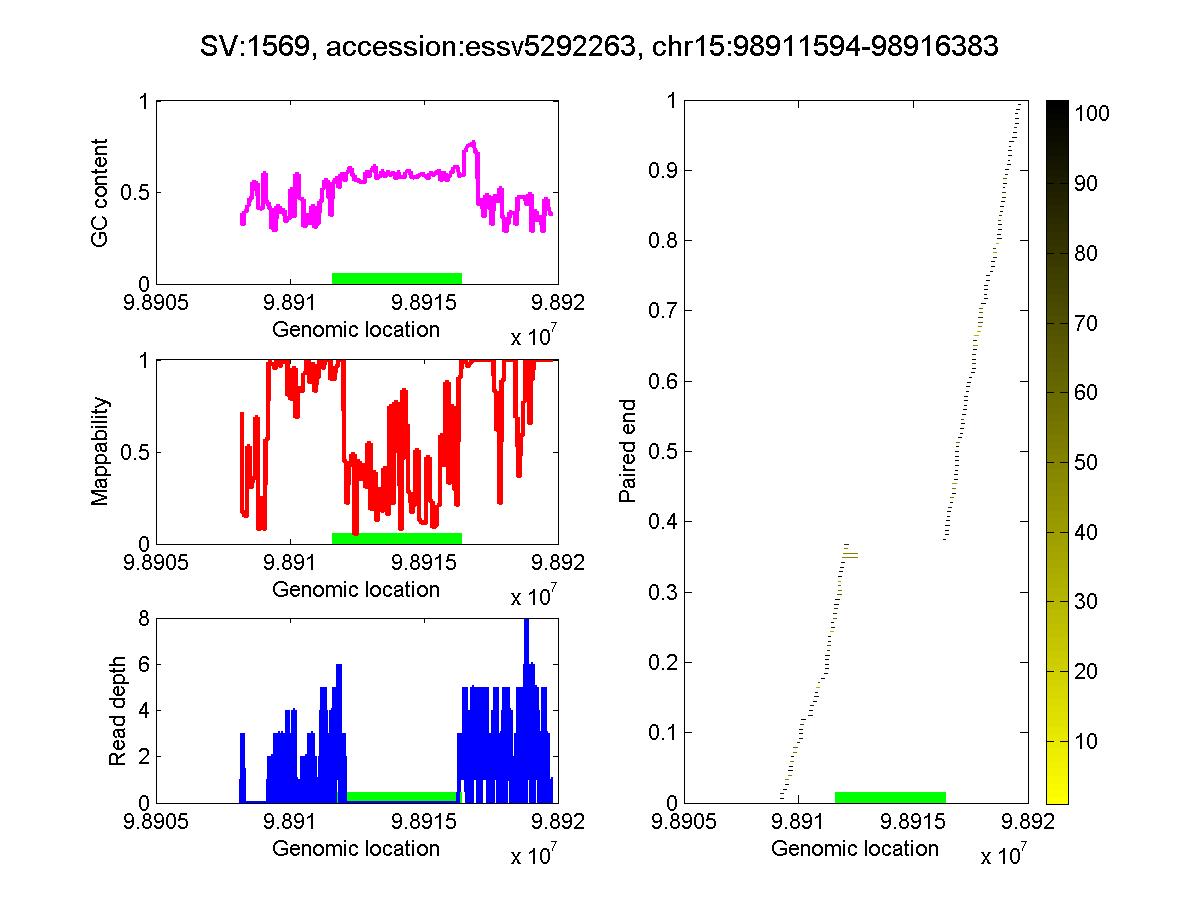

Supplement: Supplementary Materials — Supplementary data are available with this article at http://gr.xjtu.edu.cn/c/document_library/get_file?p_l_id=2403541&folderId=2539941&name=DLFE-115097.zip. Table S1 lists the complete information of suspicious variants and false positives, and the FIG directory contains the validation figures of each false positive. [file 8420547.f1.zip › 8420547.f1/FIG/SV1569.jpg]

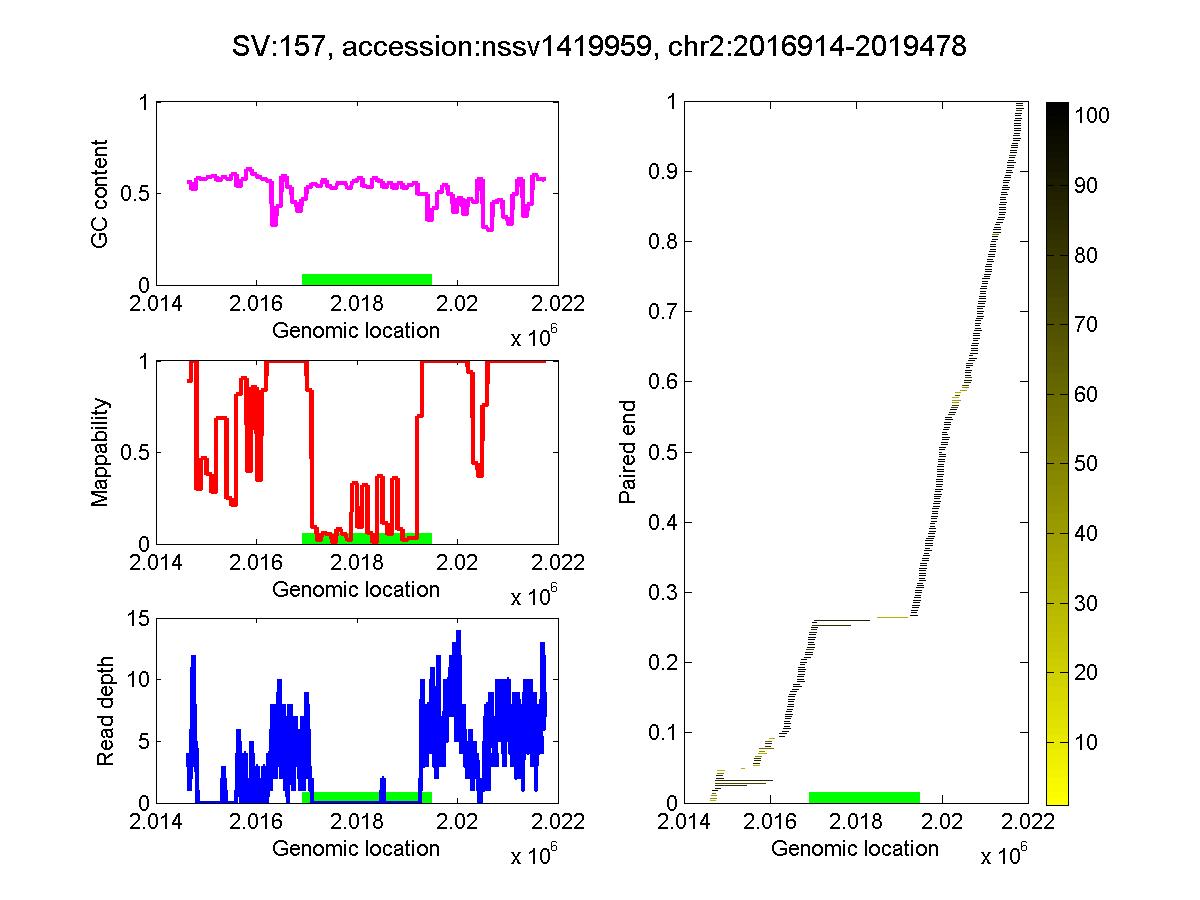

Supplement: Supplementary Materials — Supplementary data are available with this article at http://gr.xjtu.edu.cn/c/document_library/get_file?p_l_id=2403541&folderId=2539941&name=DLFE-115097.zip. Table S1 lists the complete information of suspicious variants and false positives, and the FIG directory contains the validation figures of each false positive. [file 8420547.f1.zip › 8420547.f1/FIG/SV157.jpg]

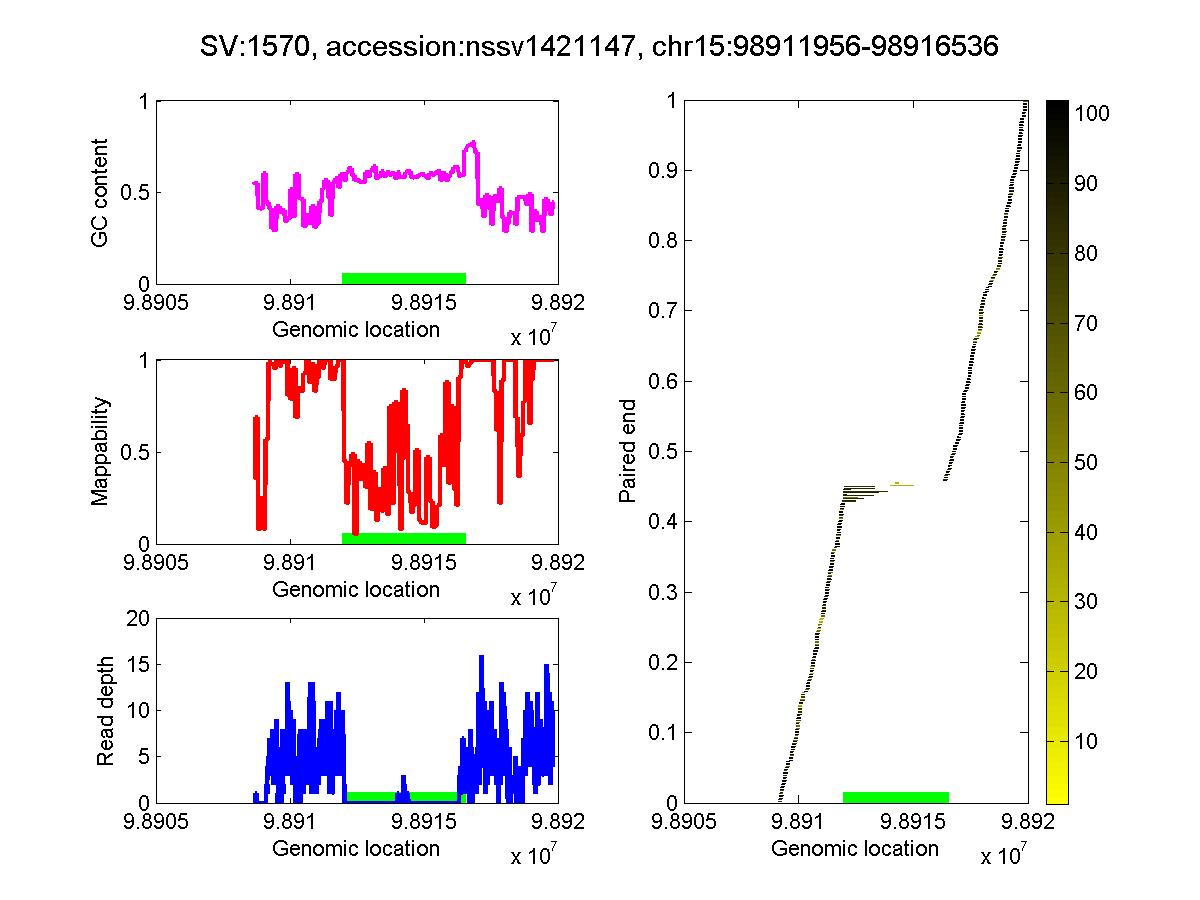

Supplement: Supplementary Materials — Supplementary data are available with this article at http://gr.xjtu.edu.cn/c/document_library/get_file?p_l_id=2403541&folderId=2539941&name=DLFE-115097.zip. Table S1 lists the complete information of suspicious variants and false positives, and the FIG directory contains the validation figures of each false positive. [file 8420547.f1.zip › 8420547.f1/FIG/SV1570.jpg]

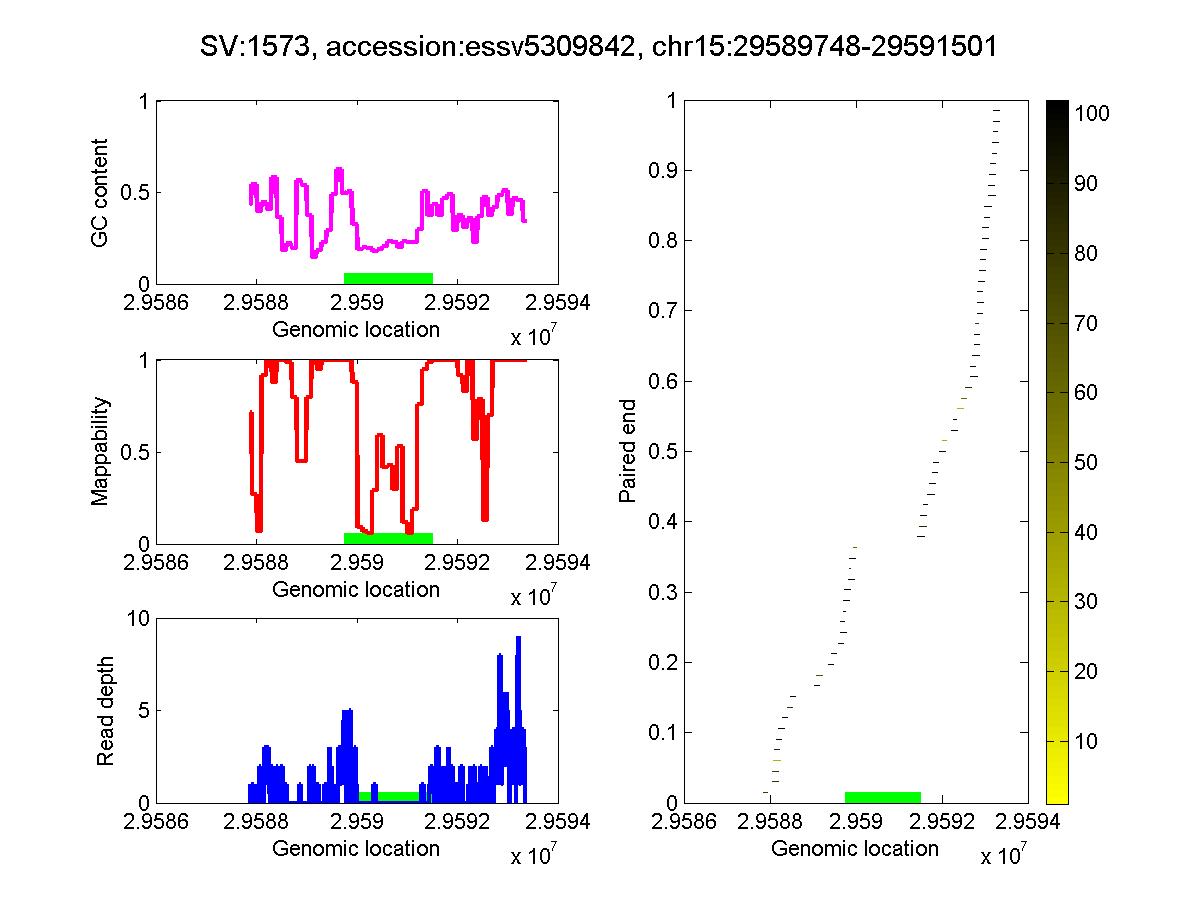

Supplement: Supplementary Materials — Supplementary data are available with this article at http://gr.xjtu.edu.cn/c/document_library/get_file?p_l_id=2403541&folderId=2539941&name=DLFE-115097.zip. Table S1 lists the complete information of suspicious variants and false positives, and the FIG directory contains the validation figures of each false positive. [file 8420547.f1.zip › 8420547.f1/FIG/SV1573.jpg]

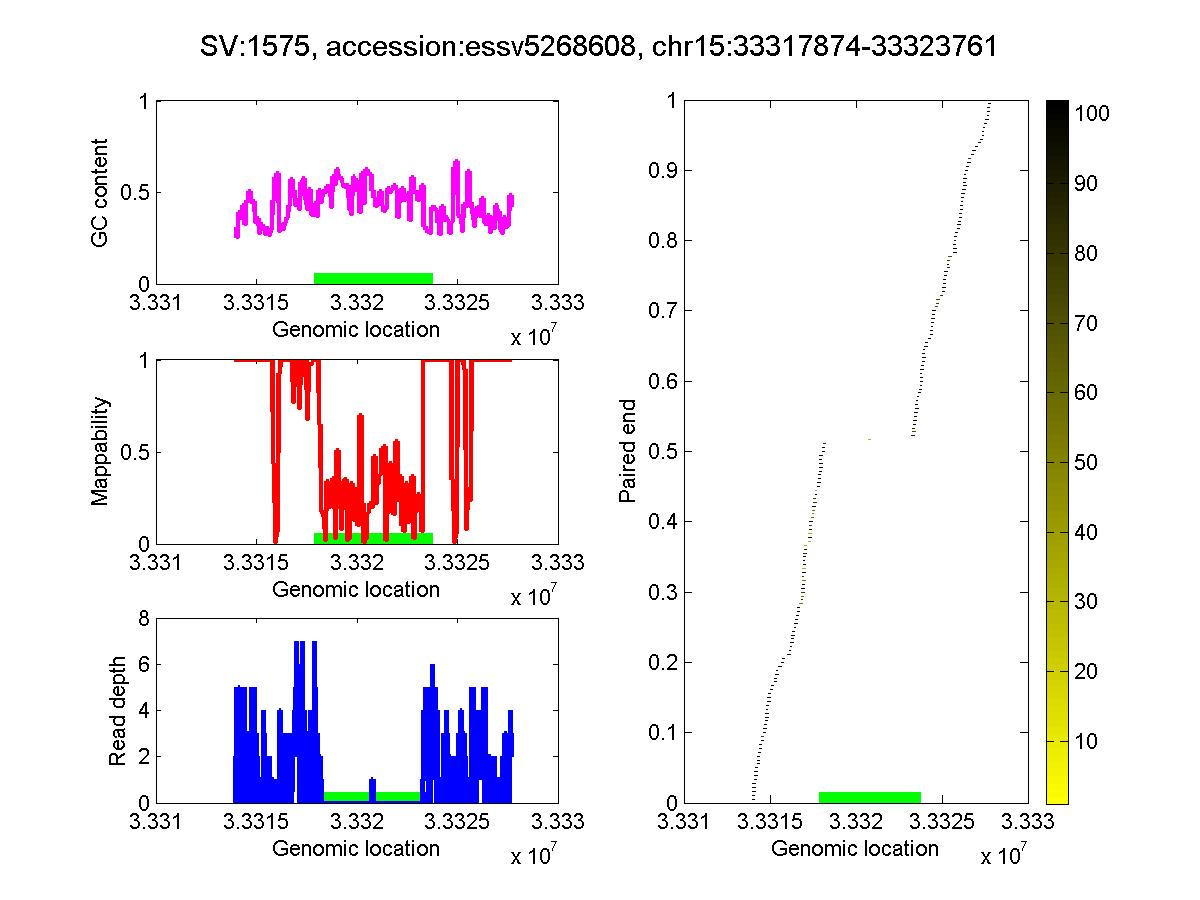

Supplement: Supplementary Materials — Supplementary data are available with this article at http://gr.xjtu.edu.cn/c/document_library/get_file?p_l_id=2403541&folderId=2539941&name=DLFE-115097.zip. Table S1 lists the complete information of suspicious variants and false positives, and the FIG directory contains the validation figures of each false positive. [file 8420547.f1.zip › 8420547.f1/FIG/SV1575.jpg]

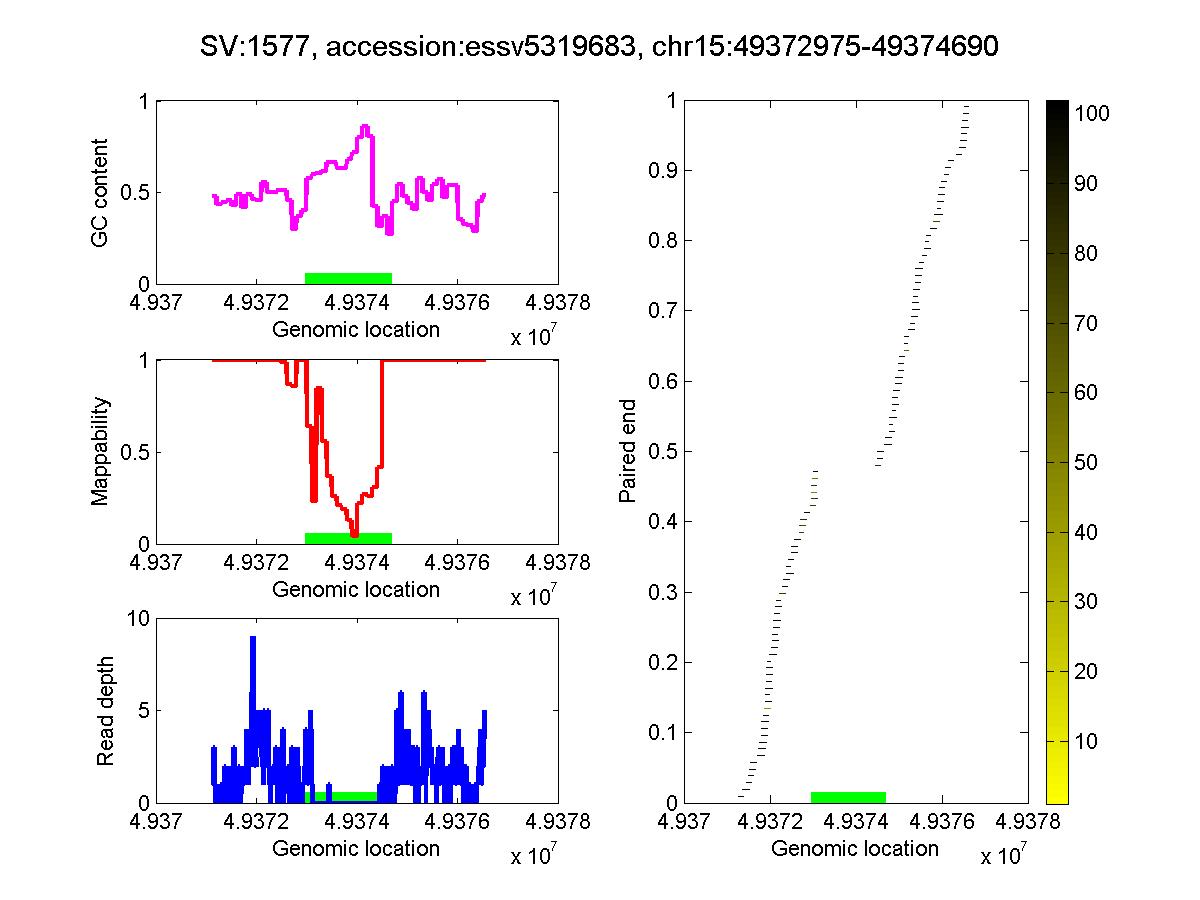

Supplement: Supplementary Materials — Supplementary data are available with this article at http://gr.xjtu.edu.cn/c/document_library/get_file?p_l_id=2403541&folderId=2539941&name=DLFE-115097.zip. Table S1 lists the complete information of suspicious variants and false positives, and the FIG directory contains the validation figures of each false positive. [file 8420547.f1.zip › 8420547.f1/FIG/SV1577.jpg]

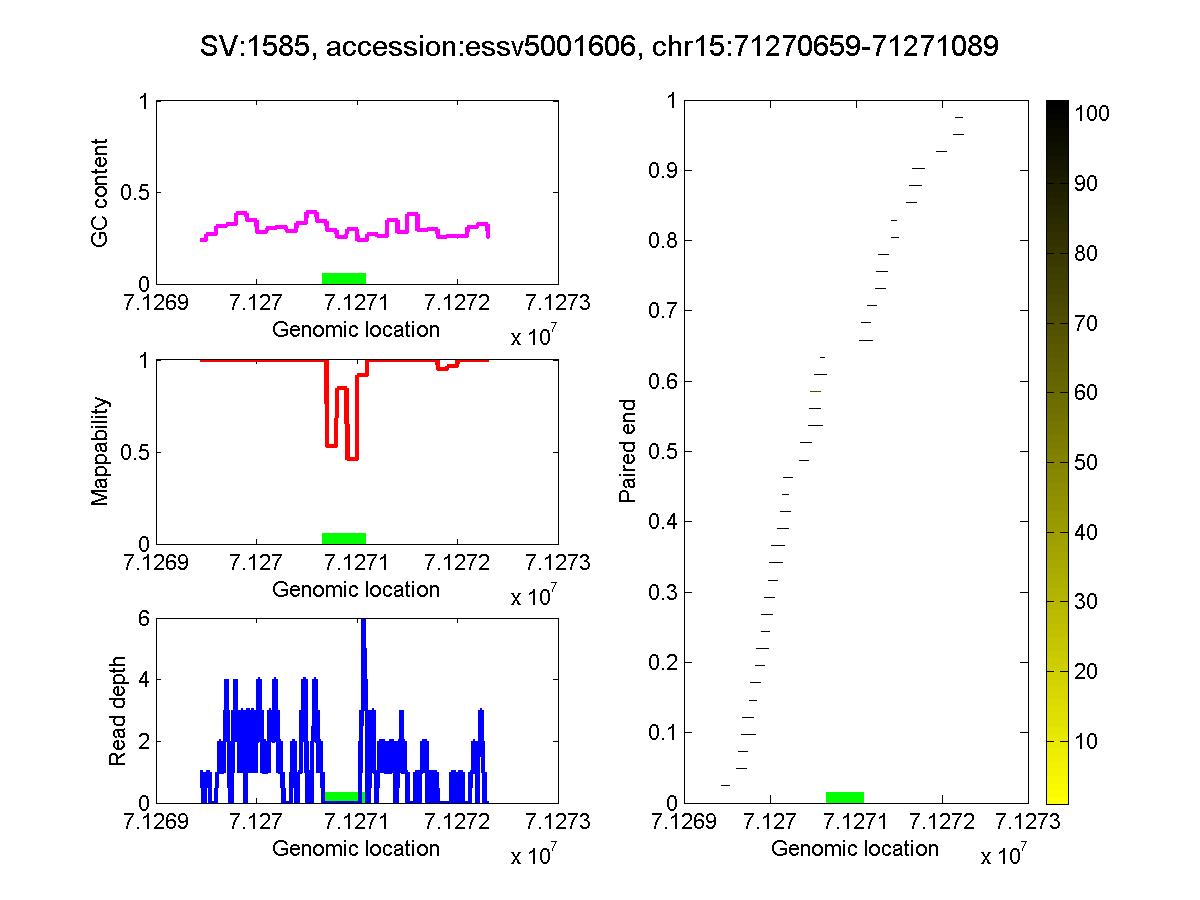

Supplement: Supplementary Materials — Supplementary data are available with this article at http://gr.xjtu.edu.cn/c/document_library/get_file?p_l_id=2403541&folderId=2539941&name=DLFE-115097.zip. Table S1 lists the complete information of suspicious variants and false positives, and the FIG directory contains the validation figures of each false positive. [file 8420547.f1.zip › 8420547.f1/FIG/SV1585.jpg]

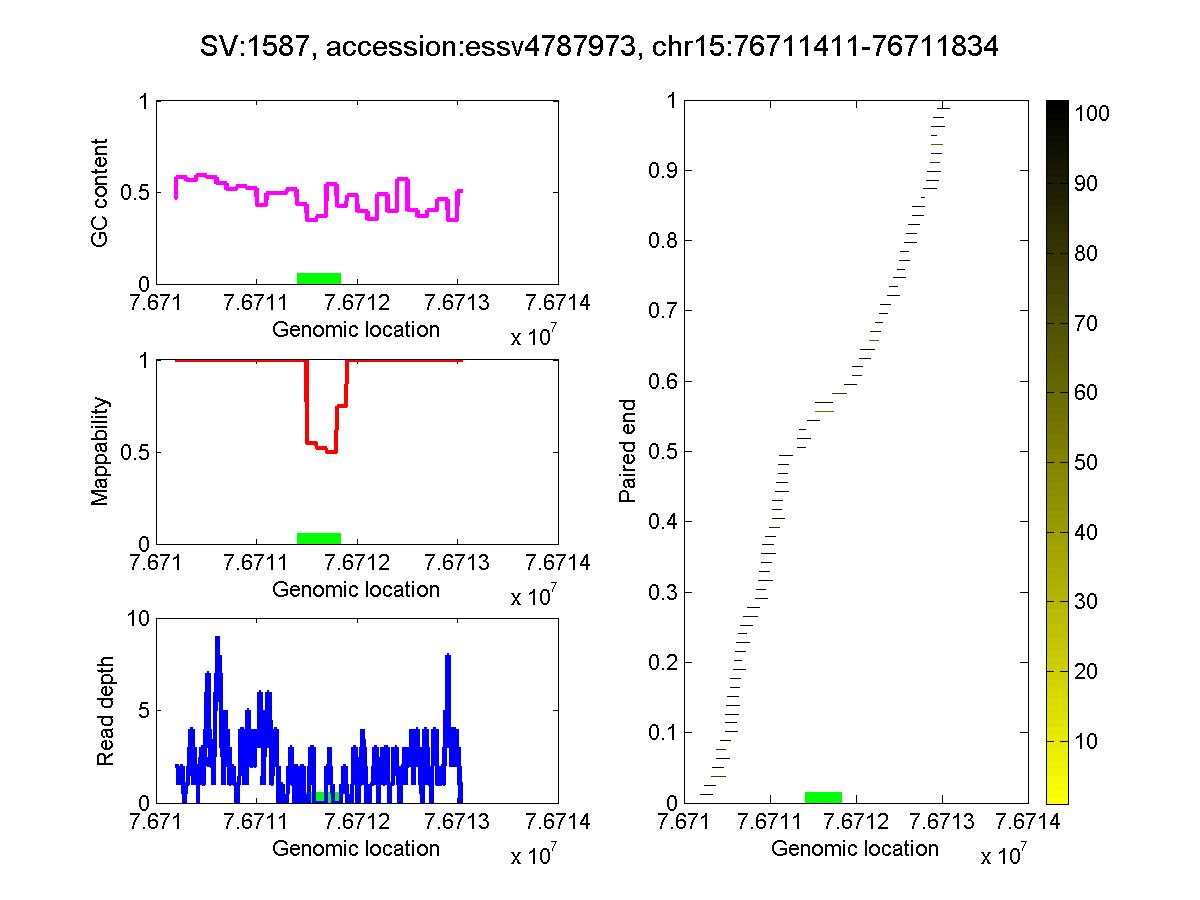

Supplement: Supplementary Materials — Supplementary data are available with this article at http://gr.xjtu.edu.cn/c/document_library/get_file?p_l_id=2403541&folderId=2539941&name=DLFE-115097.zip. Table S1 lists the complete information of suspicious variants and false positives, and the FIG directory contains the validation figures of each false positive. [file 8420547.f1.zip › 8420547.f1/FIG/SV1587.jpg]

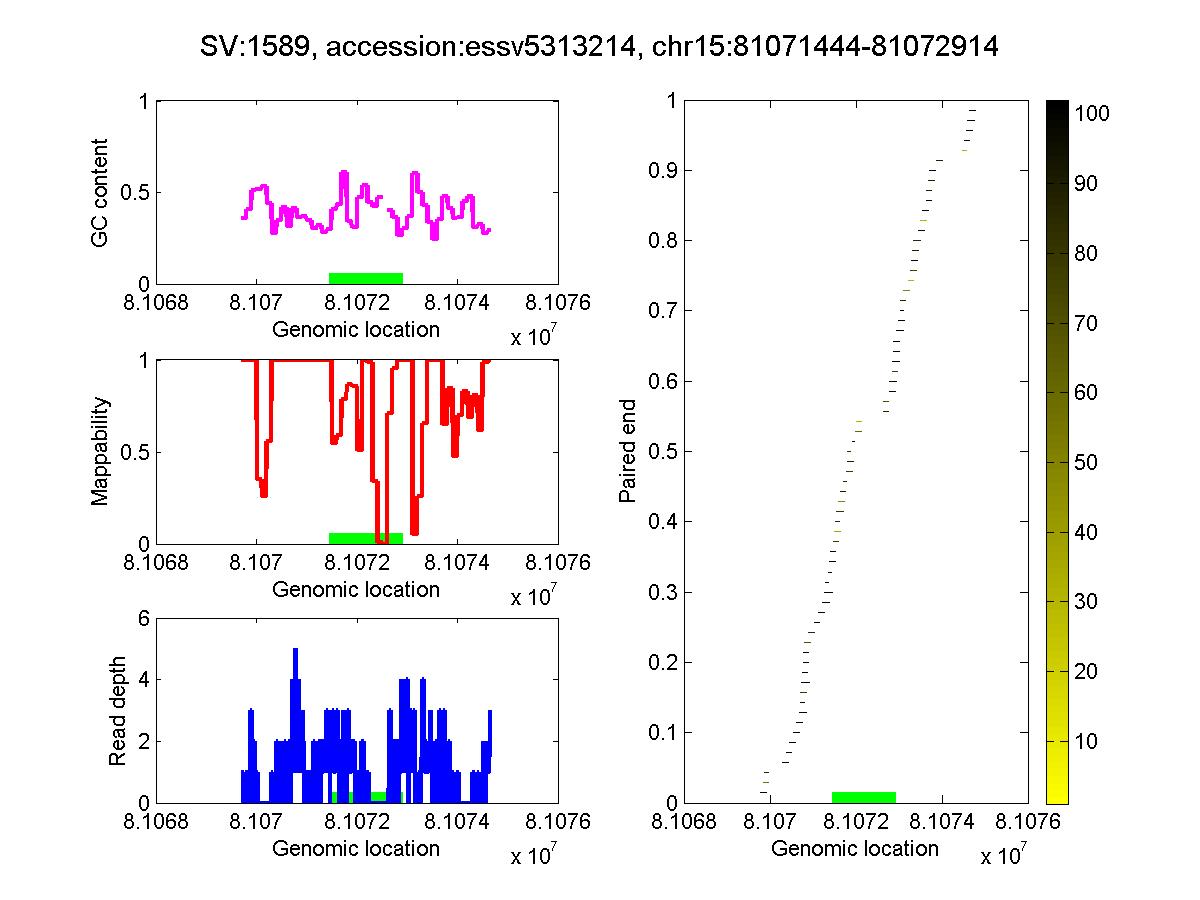

Supplement: Supplementary Materials — Supplementary data are available with this article at http://gr.xjtu.edu.cn/c/document_library/get_file?p_l_id=2403541&folderId=2539941&name=DLFE-115097.zip. Table S1 lists the complete information of suspicious variants and false positives, and the FIG directory contains the validation figures of each false positive. [file 8420547.f1.zip › 8420547.f1/FIG/SV1589.jpg]

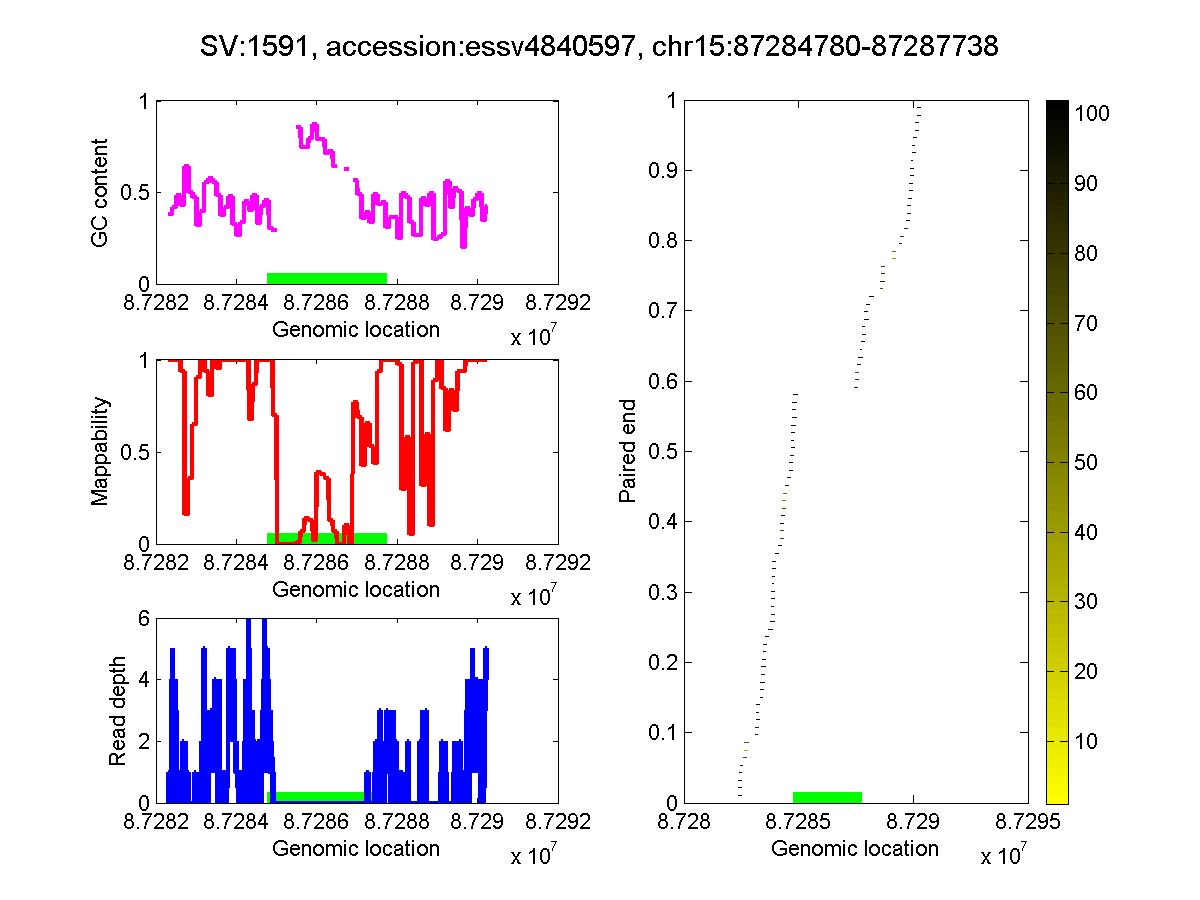

Supplement: Supplementary Materials — Supplementary data are available with this article at http://gr.xjtu.edu.cn/c/document_library/get_file?p_l_id=2403541&folderId=2539941&name=DLFE-115097.zip. Table S1 lists the complete information of suspicious variants and false positives, and the FIG directory contains the validation figures of each false positive. [file 8420547.f1.zip › 8420547.f1/FIG/SV1591.jpg]

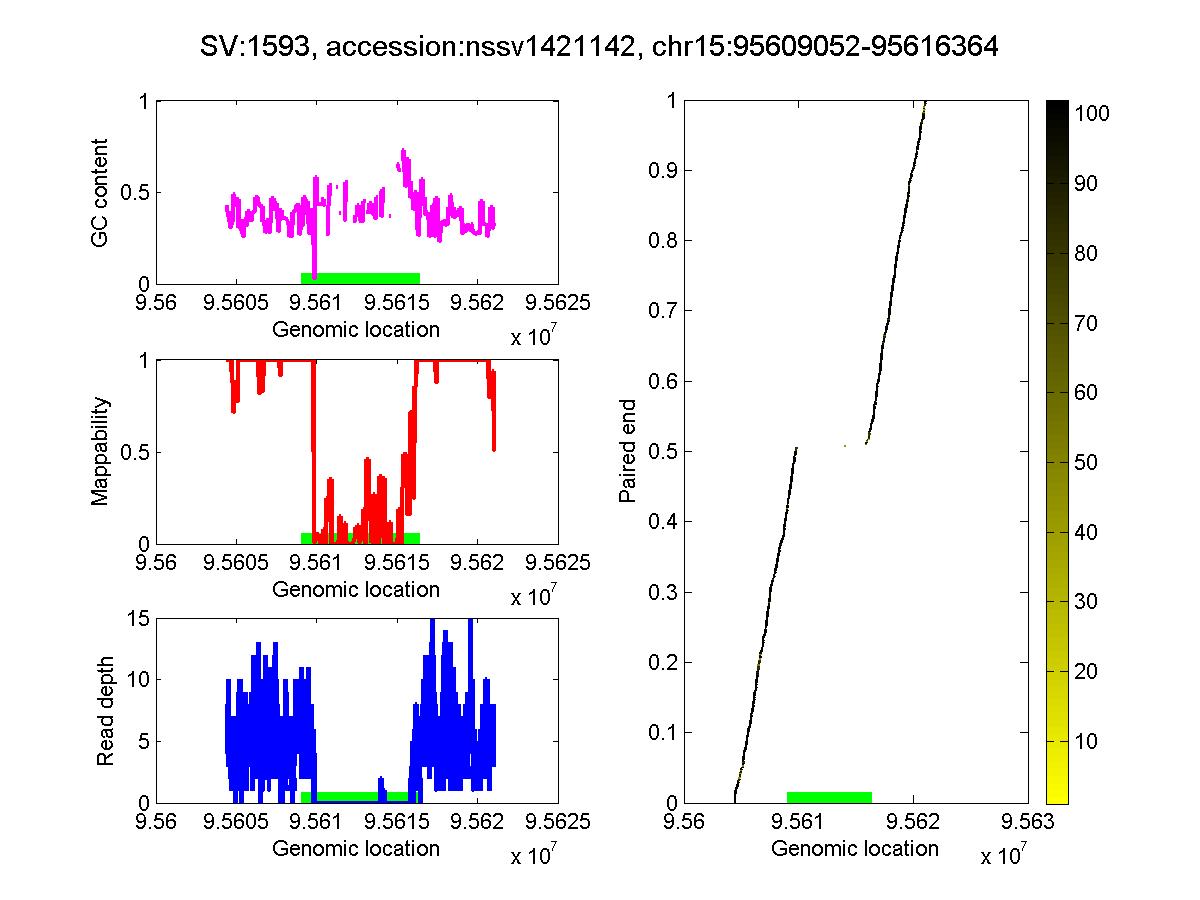

Supplement: Supplementary Materials — Supplementary data are available with this article at http://gr.xjtu.edu.cn/c/document_library/get_file?p_l_id=2403541&folderId=2539941&name=DLFE-115097.zip. Table S1 lists the complete information of suspicious variants and false positives, and the FIG directory contains the validation figures of each false positive. [file 8420547.f1.zip › 8420547.f1/FIG/SV1593.jpg]

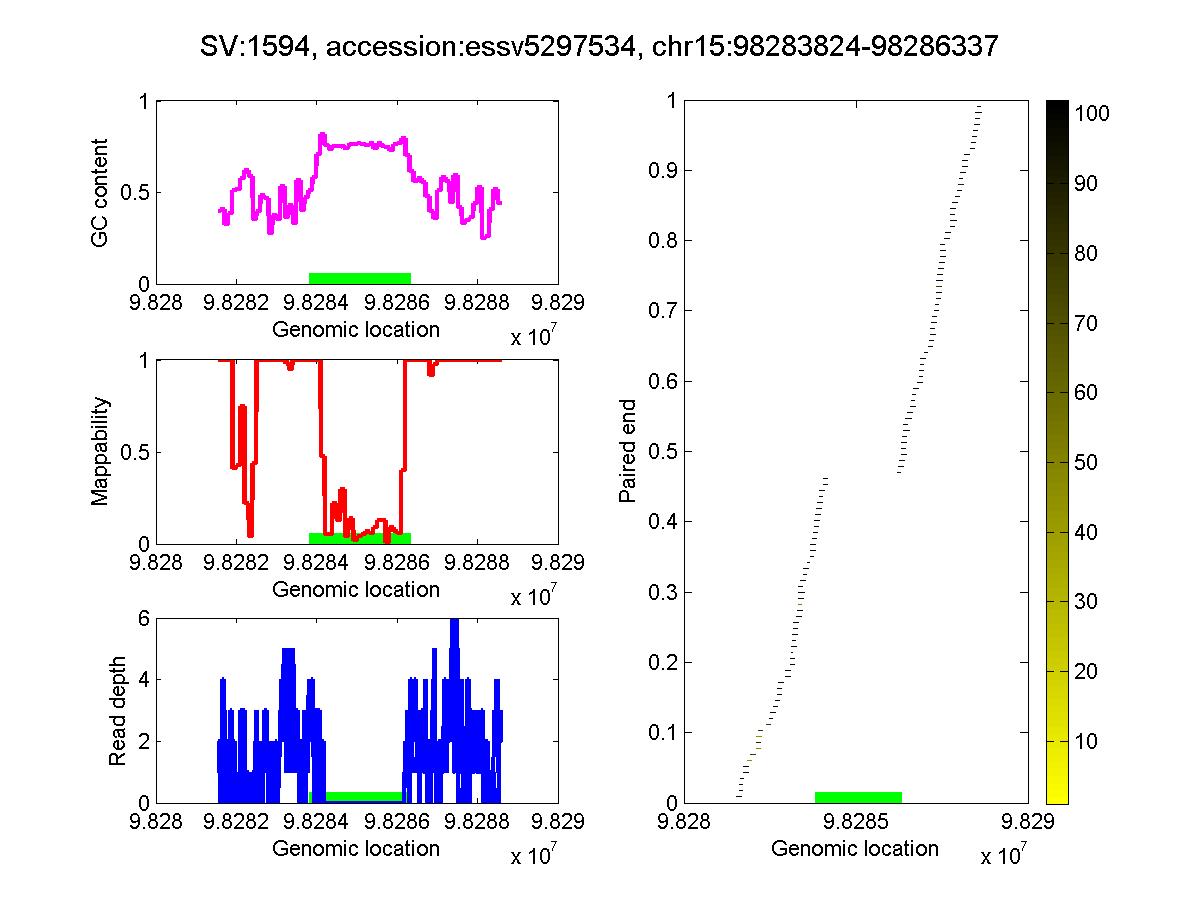

Supplement: Supplementary Materials — Supplementary data are available with this article at http://gr.xjtu.edu.cn/c/document_library/get_file?p_l_id=2403541&folderId=2539941&name=DLFE-115097.zip. Table S1 lists the complete information of suspicious variants and false positives, and the FIG directory contains the validation figures of each false positive. [file 8420547.f1.zip › 8420547.f1/FIG/SV1594.jpg]

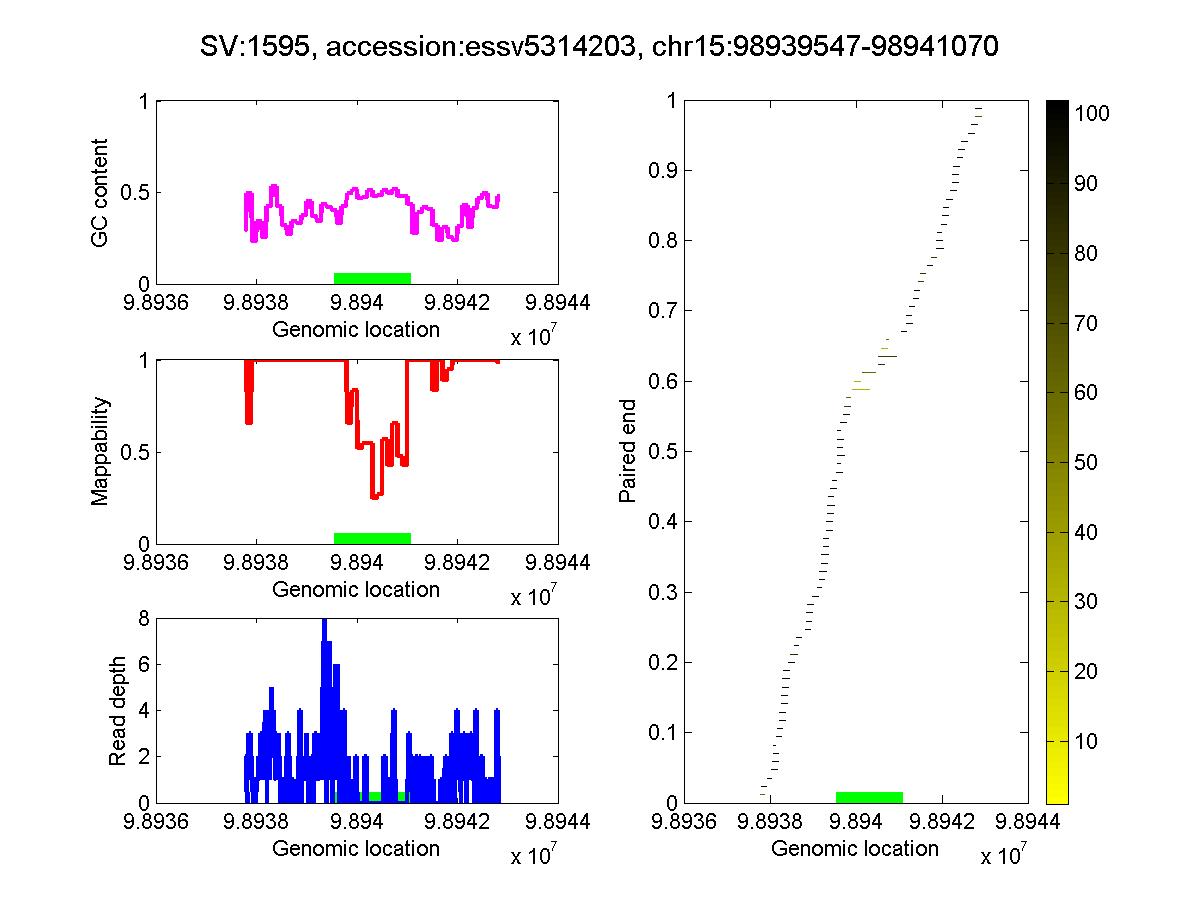

Supplement: Supplementary Materials — Supplementary data are available with this article at http://gr.xjtu.edu.cn/c/document_library/get_file?p_l_id=2403541&folderId=2539941&name=DLFE-115097.zip. Table S1 lists the complete information of suspicious variants and false positives, and the FIG directory contains the validation figures of each false positive. [file 8420547.f1.zip › 8420547.f1/FIG/SV1595.jpg]

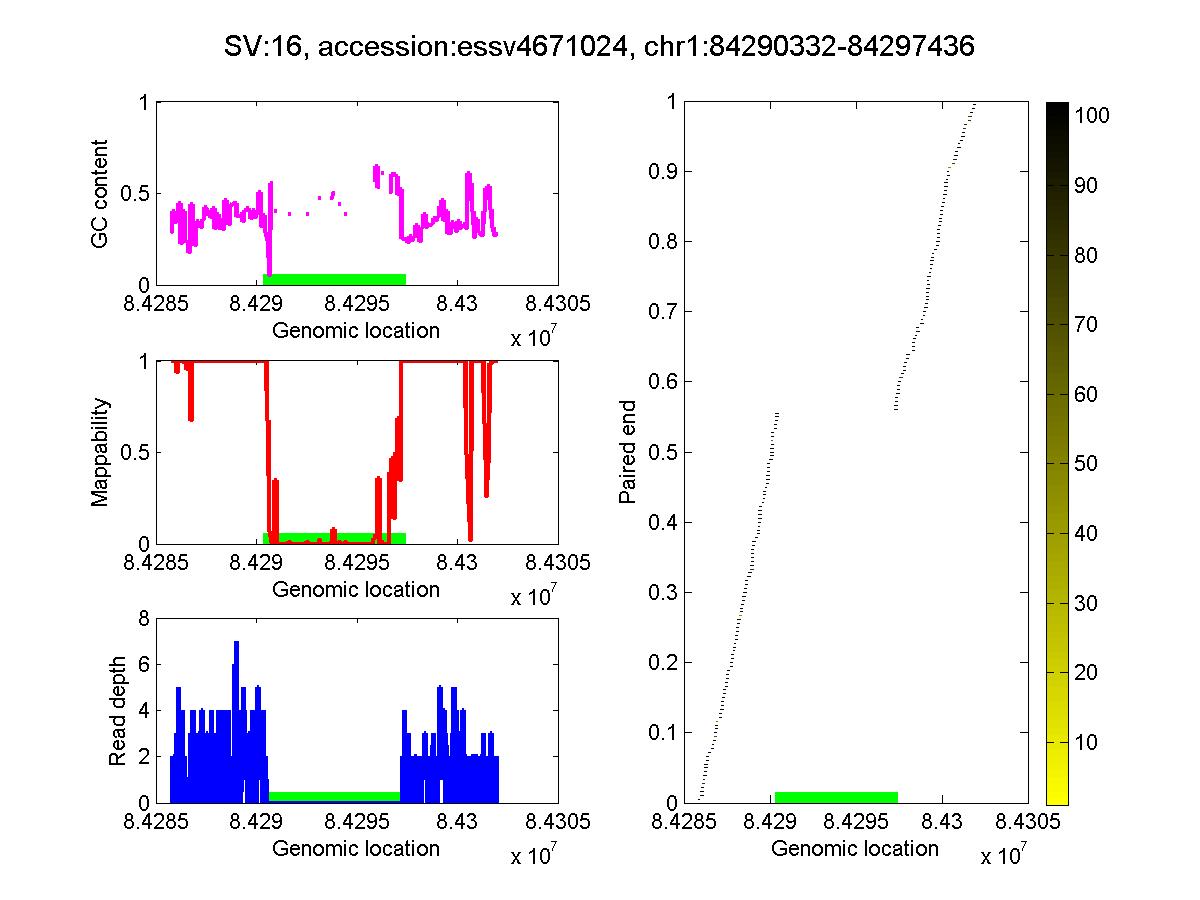

Supplement: Supplementary Materials — Supplementary data are available with this article at http://gr.xjtu.edu.cn/c/document_library/get_file?p_l_id=2403541&folderId=2539941&name=DLFE-115097.zip. Table S1 lists the complete information of suspicious variants and false positives, and the FIG directory contains the validation figures of each false positive. [file 8420547.f1.zip › 8420547.f1/FIG/SV16.jpg]

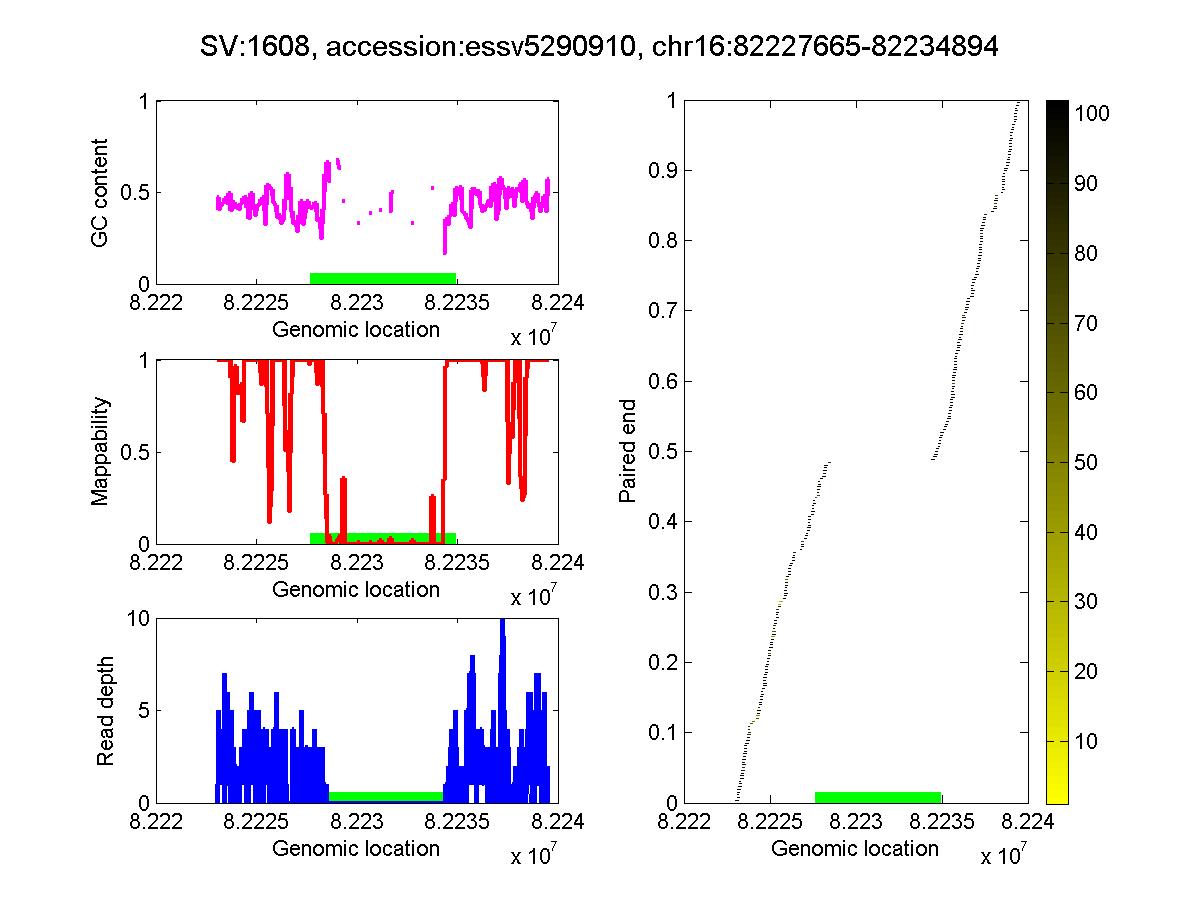

Supplement: Supplementary Materials — Supplementary data are available with this article at http://gr.xjtu.edu.cn/c/document_library/get_file?p_l_id=2403541&folderId=2539941&name=DLFE-115097.zip. Table S1 lists the complete information of suspicious variants and false positives, and the FIG directory contains the validation figures of each false positive. [file 8420547.f1.zip › 8420547.f1/FIG/SV1608.jpg]

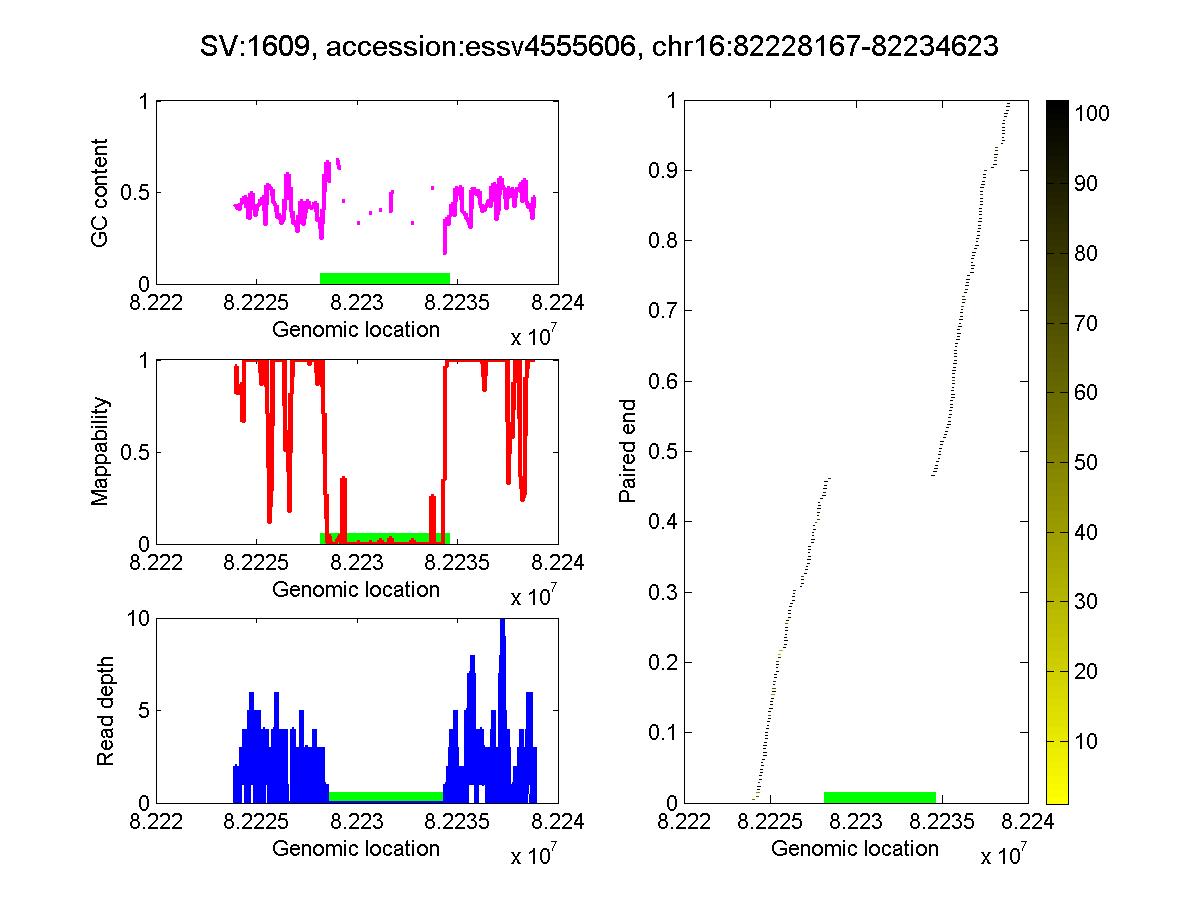

Supplement: Supplementary Materials — Supplementary data are available with this article at http://gr.xjtu.edu.cn/c/document_library/get_file?p_l_id=2403541&folderId=2539941&name=DLFE-115097.zip. Table S1 lists the complete information of suspicious variants and false positives, and the FIG directory contains the validation figures of each false positive. [file 8420547.f1.zip › 8420547.f1/FIG/SV1609.jpg]

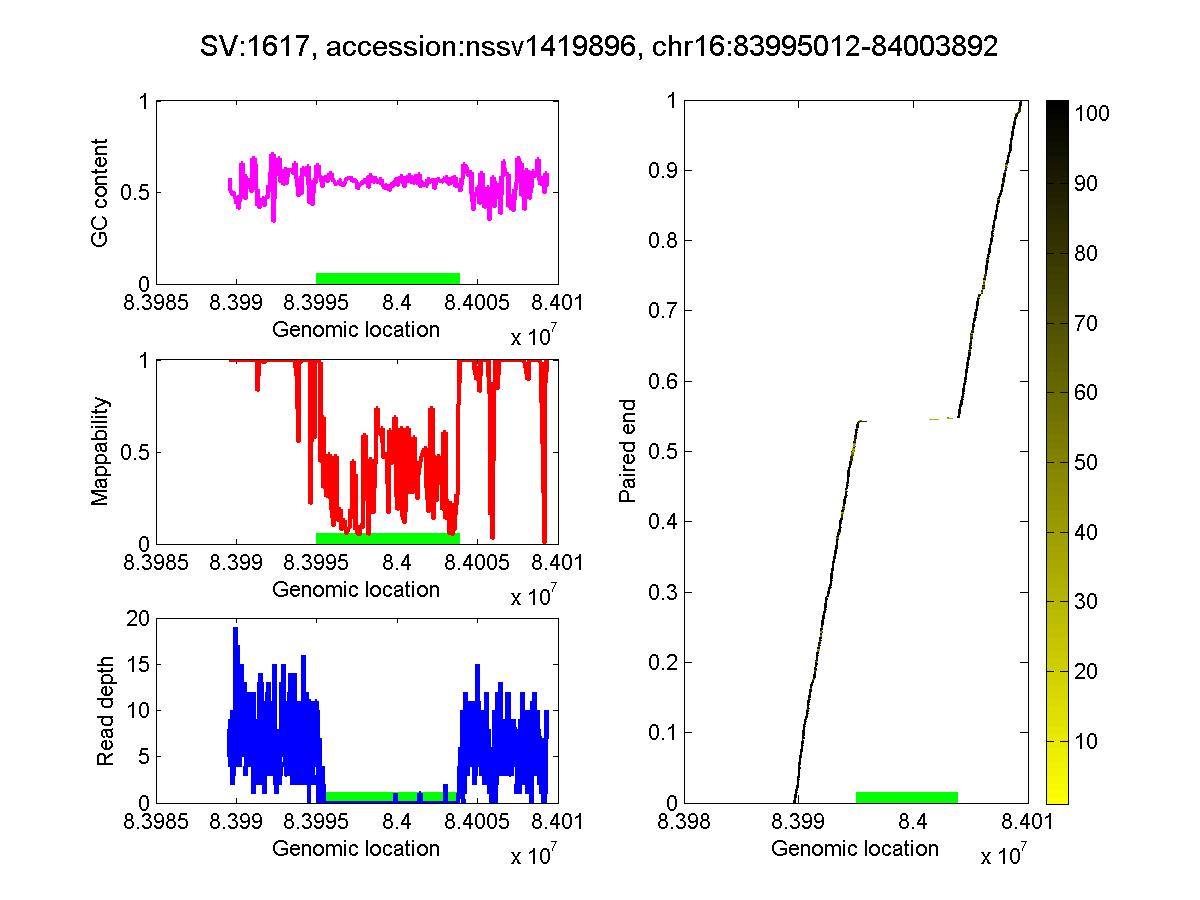

Supplement: Supplementary Materials — Supplementary data are available with this article at http://gr.xjtu.edu.cn/c/document_library/get_file?p_l_id=2403541&folderId=2539941&name=DLFE-115097.zip. Table S1 lists the complete information of suspicious variants and false positives, and the FIG directory contains the validation figures of each false positive. [file 8420547.f1.zip › 8420547.f1/FIG/SV1617.jpg]

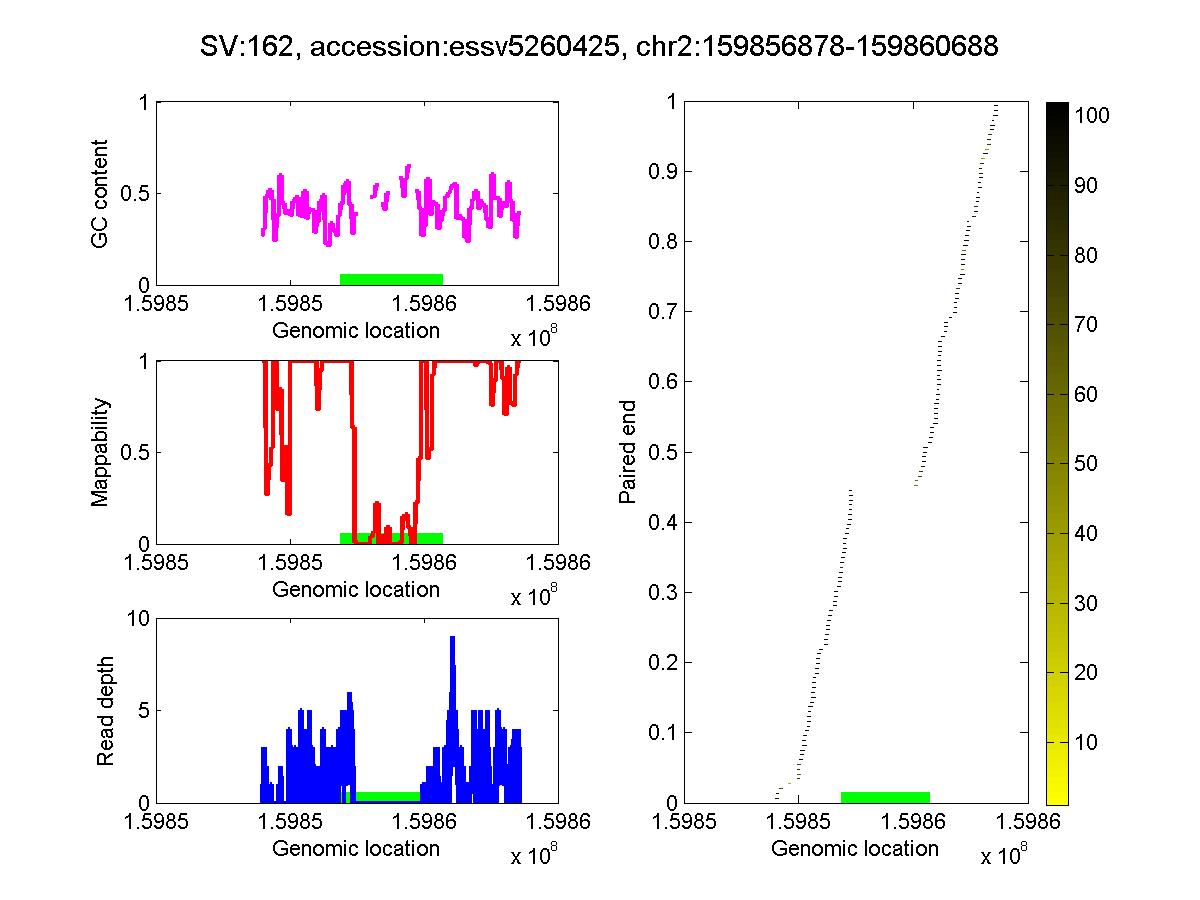

Supplement: Supplementary Materials — Supplementary data are available with this article at http://gr.xjtu.edu.cn/c/document_library/get_file?p_l_id=2403541&folderId=2539941&name=DLFE-115097.zip. Table S1 lists the complete information of suspicious variants and false positives, and the FIG directory contains the validation figures of each false positive. [file 8420547.f1.zip › 8420547.f1/FIG/SV162.jpg]

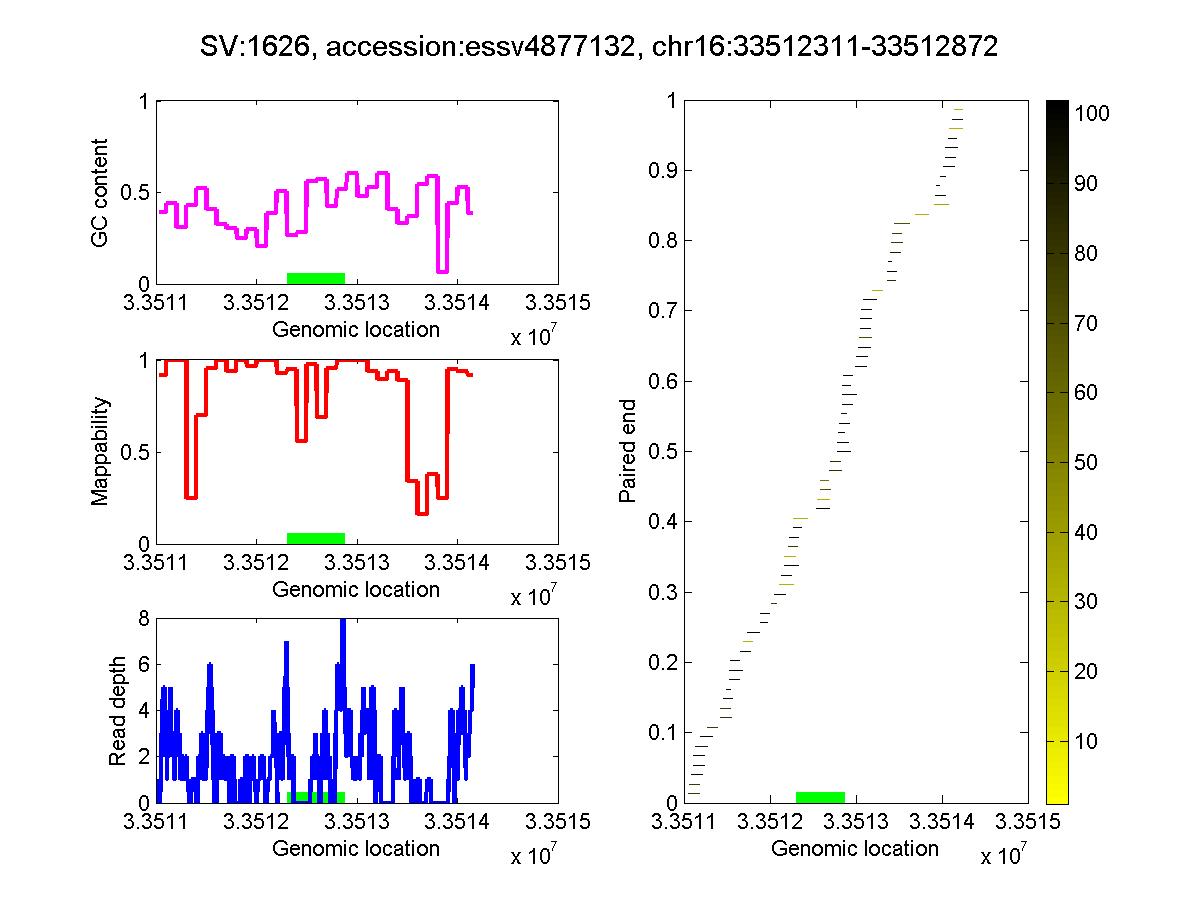

Supplement: Supplementary Materials — Supplementary data are available with this article at http://gr.xjtu.edu.cn/c/document_library/get_file?p_l_id=2403541&folderId=2539941&name=DLFE-115097.zip. Table S1 lists the complete information of suspicious variants and false positives, and the FIG directory contains the validation figures of each false positive. [file 8420547.f1.zip › 8420547.f1/FIG/SV1626.jpg]
