# Supplementary figures and images for: Detection of False-Positive Deletions from the Database of Genomic Variants (part 1 of 2)
Source: Biomed Res Int. 2019 Apr 4;2019:8420547. doi: 10.1155/2019/8420547 (PMC6475568; doi:10.1155/2019/8420547)

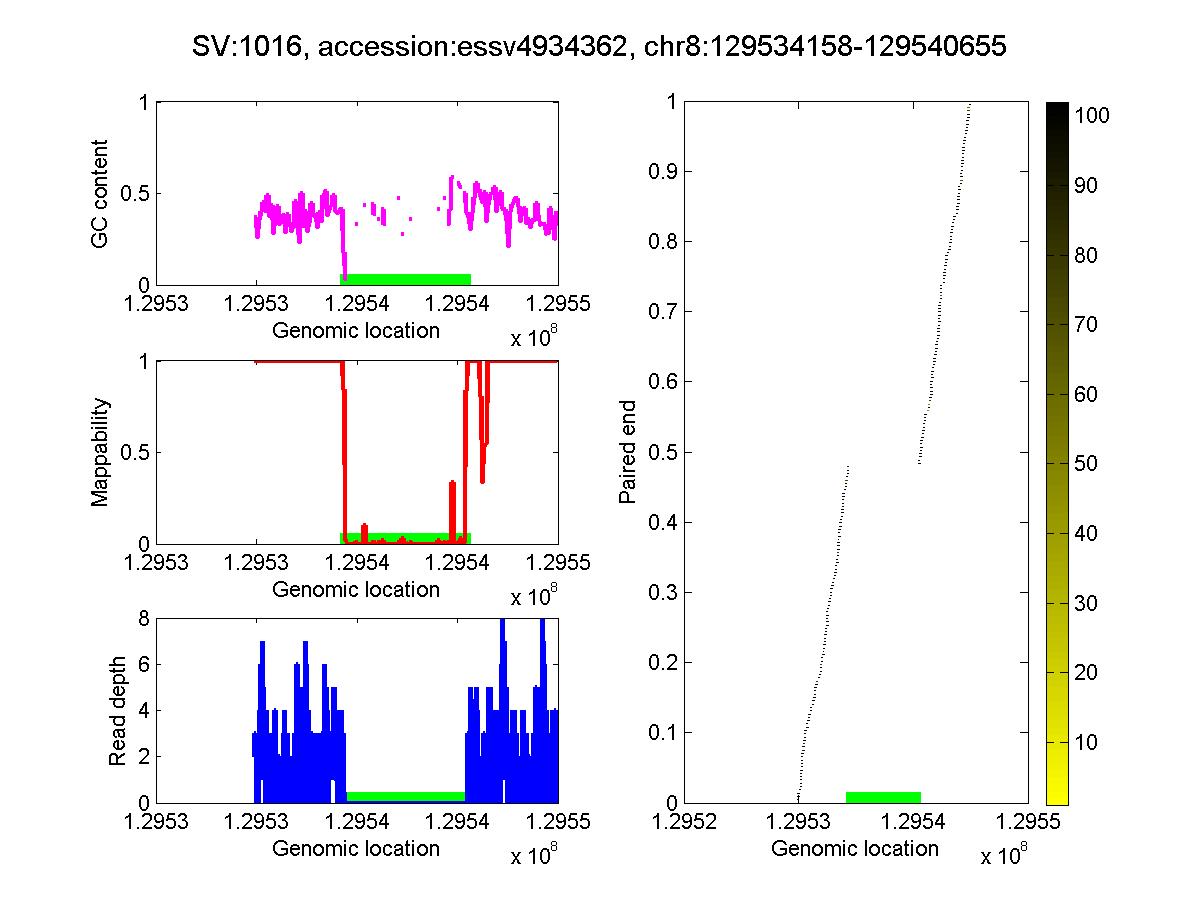

Supplement: Supplementary Materials — Supplementary data are available with this article at http://gr.xjtu.edu.cn/c/document_library/get_file?p_l_id=2403541&folderId=2539941&name=DLFE-115097.zip. Table S1 lists the complete information of suspicious variants and false positives, and the FIG directory contains the validation figures of each false positive. [file 8420547.f1.zip › 8420547.f1/FIG/SV1016.jpg]

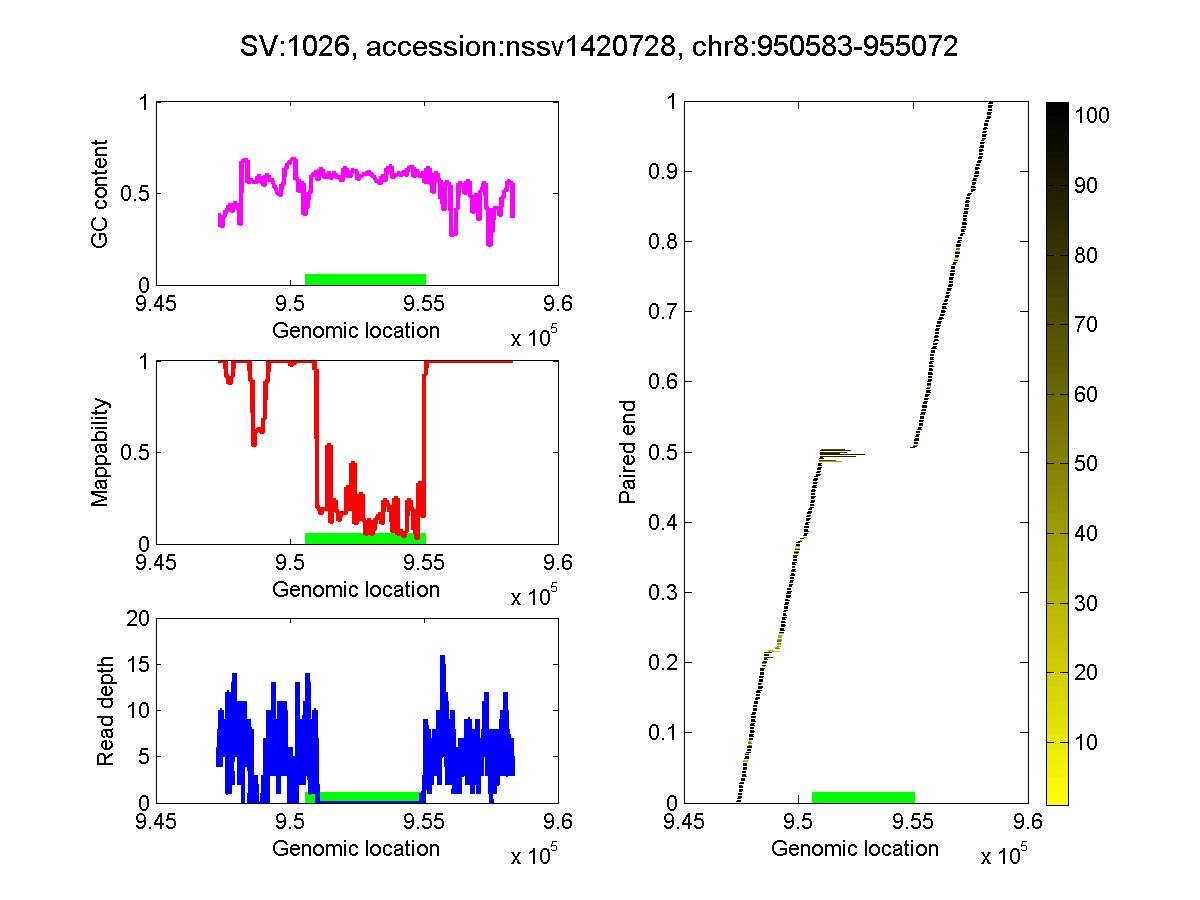

Supplement: Supplementary Materials — Supplementary data are available with this article at http://gr.xjtu.edu.cn/c/document_library/get_file?p_l_id=2403541&folderId=2539941&name=DLFE-115097.zip. Table S1 lists the complete information of suspicious variants and false positives, and the FIG directory contains the validation figures of each false positive. [file 8420547.f1.zip › 8420547.f1/FIG/SV1026.jpg]

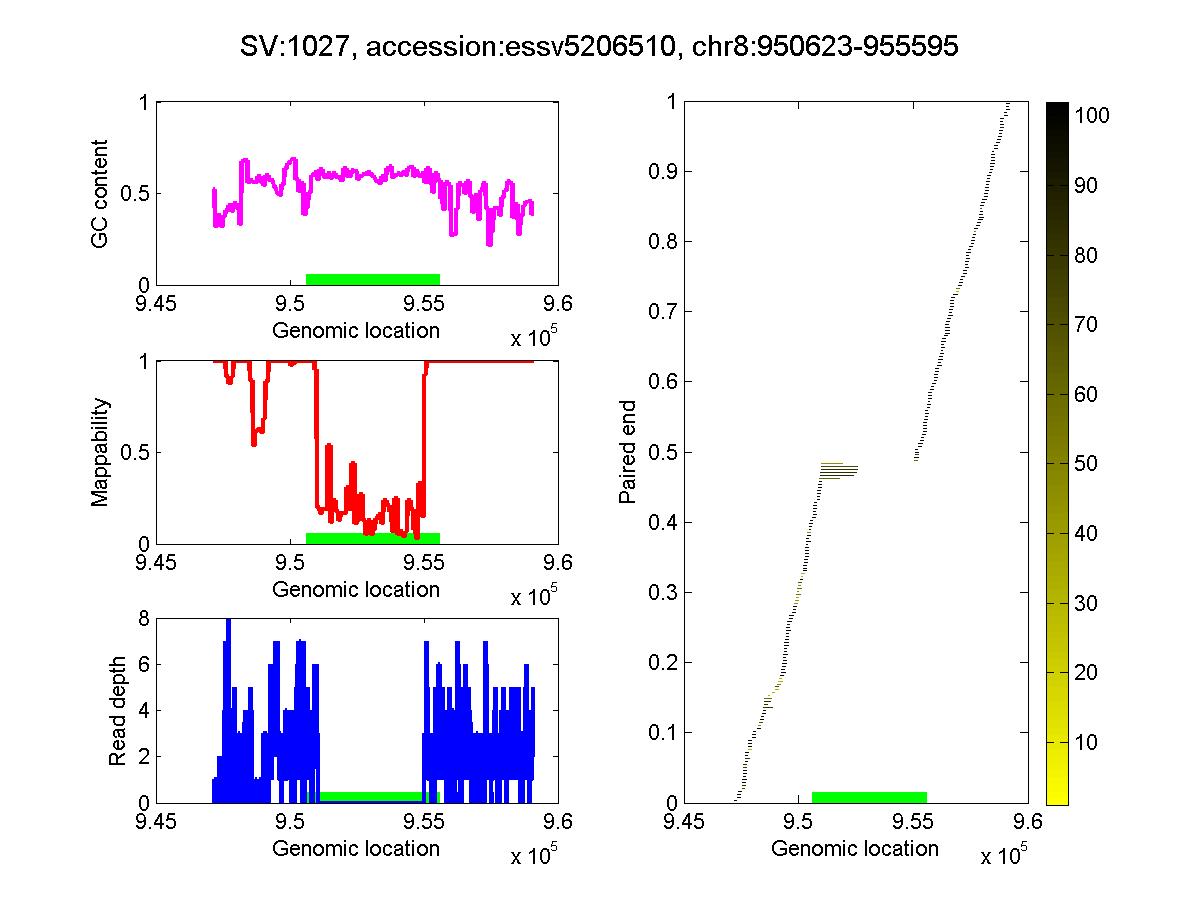

Supplement: Supplementary Materials — Supplementary data are available with this article at http://gr.xjtu.edu.cn/c/document_library/get_file?p_l_id=2403541&folderId=2539941&name=DLFE-115097.zip. Table S1 lists the complete information of suspicious variants and false positives, and the FIG directory contains the validation figures of each false positive. [file 8420547.f1.zip › 8420547.f1/FIG/SV1027.jpg]

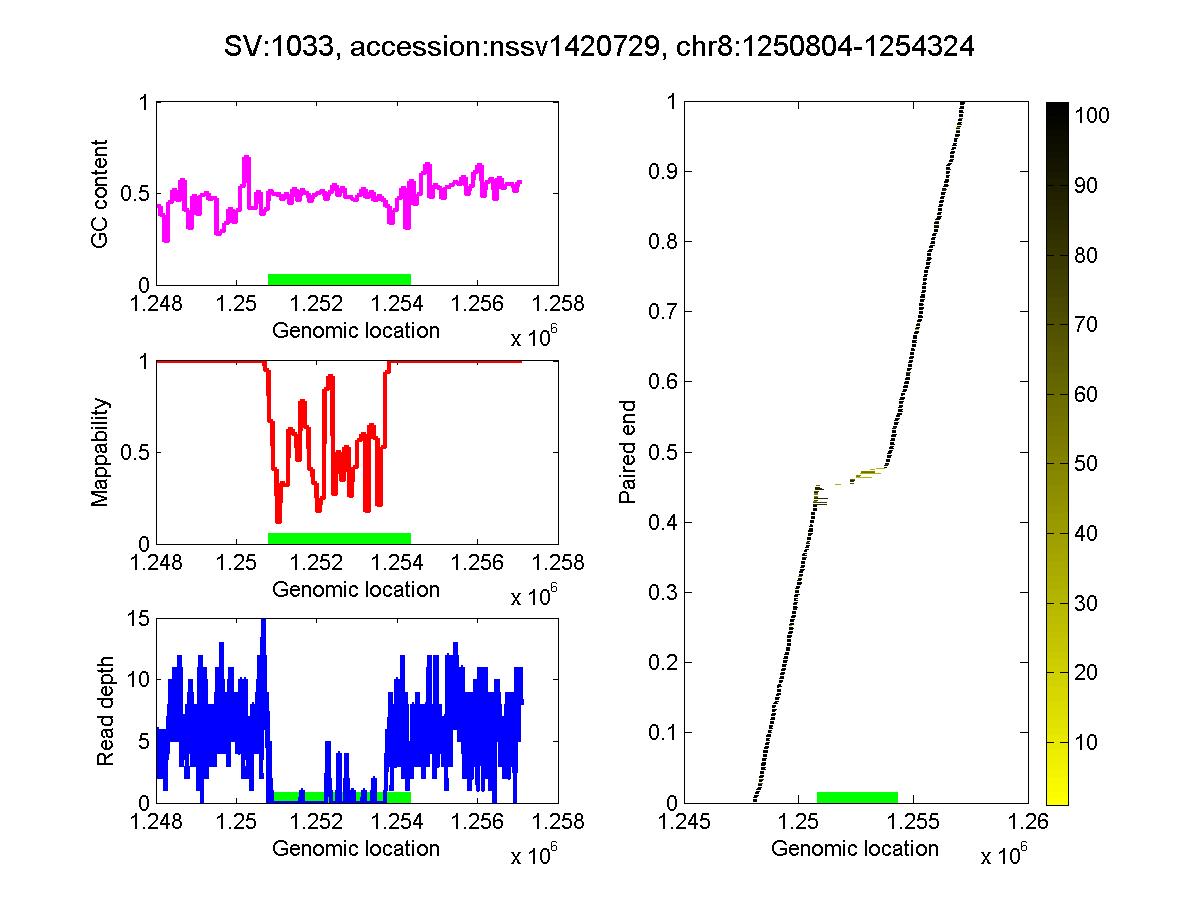

Supplement: Supplementary Materials — Supplementary data are available with this article at http://gr.xjtu.edu.cn/c/document_library/get_file?p_l_id=2403541&folderId=2539941&name=DLFE-115097.zip. Table S1 lists the complete information of suspicious variants and false positives, and the FIG directory contains the validation figures of each false positive. [file 8420547.f1.zip › 8420547.f1/FIG/SV1033.jpg]

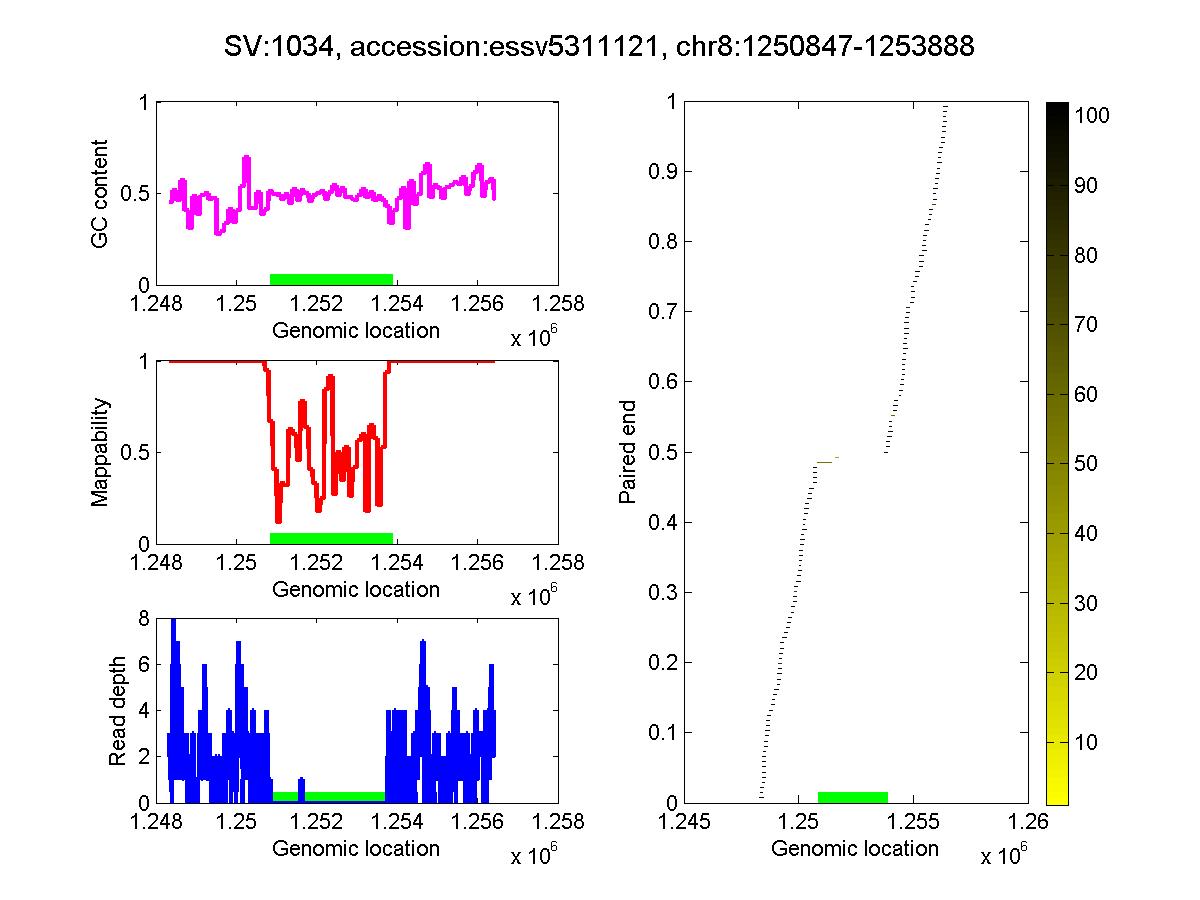

Supplement: Supplementary Materials — Supplementary data are available with this article at http://gr.xjtu.edu.cn/c/document_library/get_file?p_l_id=2403541&folderId=2539941&name=DLFE-115097.zip. Table S1 lists the complete information of suspicious variants and false positives, and the FIG directory contains the validation figures of each false positive. [file 8420547.f1.zip › 8420547.f1/FIG/SV1034.jpg]

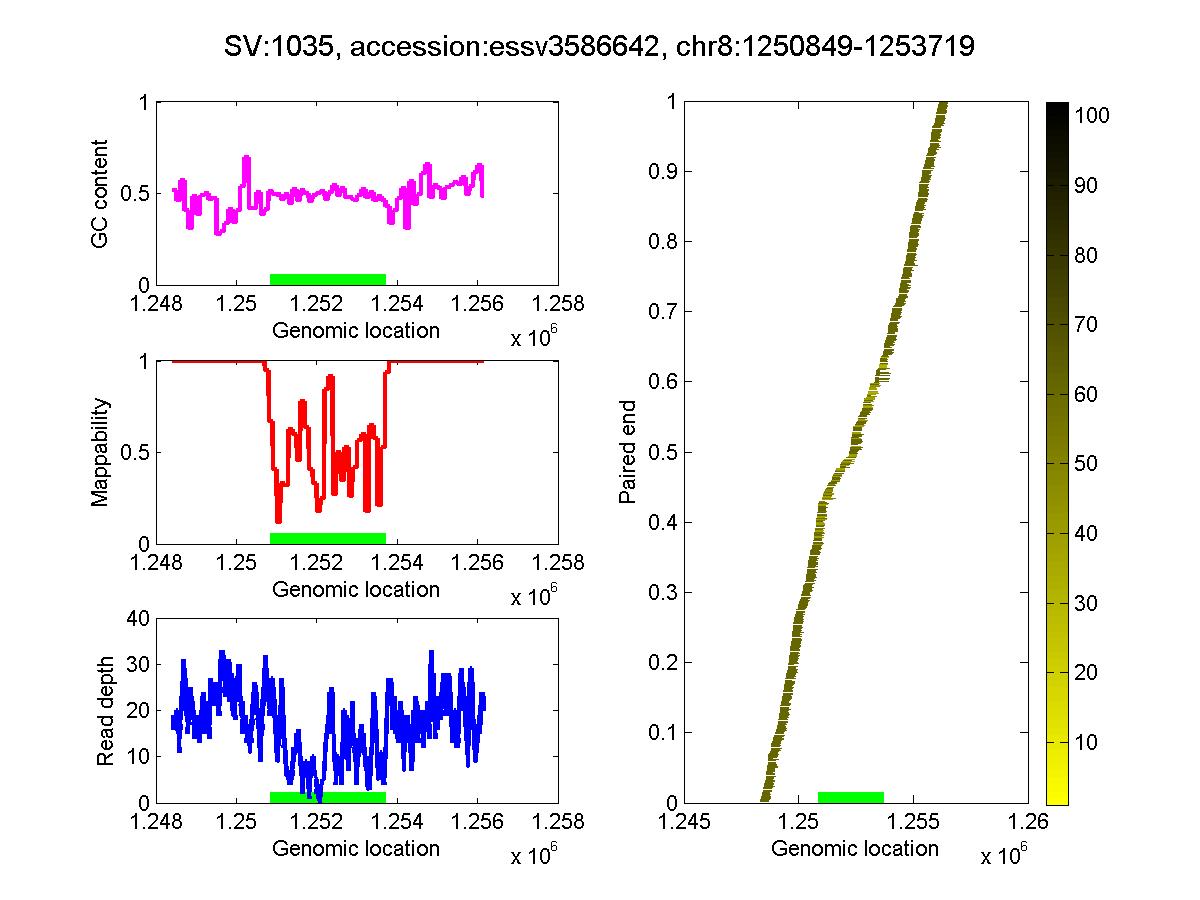

Supplement: Supplementary Materials — Supplementary data are available with this article at http://gr.xjtu.edu.cn/c/document_library/get_file?p_l_id=2403541&folderId=2539941&name=DLFE-115097.zip. Table S1 lists the complete information of suspicious variants and false positives, and the FIG directory contains the validation figures of each false positive. [file 8420547.f1.zip › 8420547.f1/FIG/SV1035.jpg]

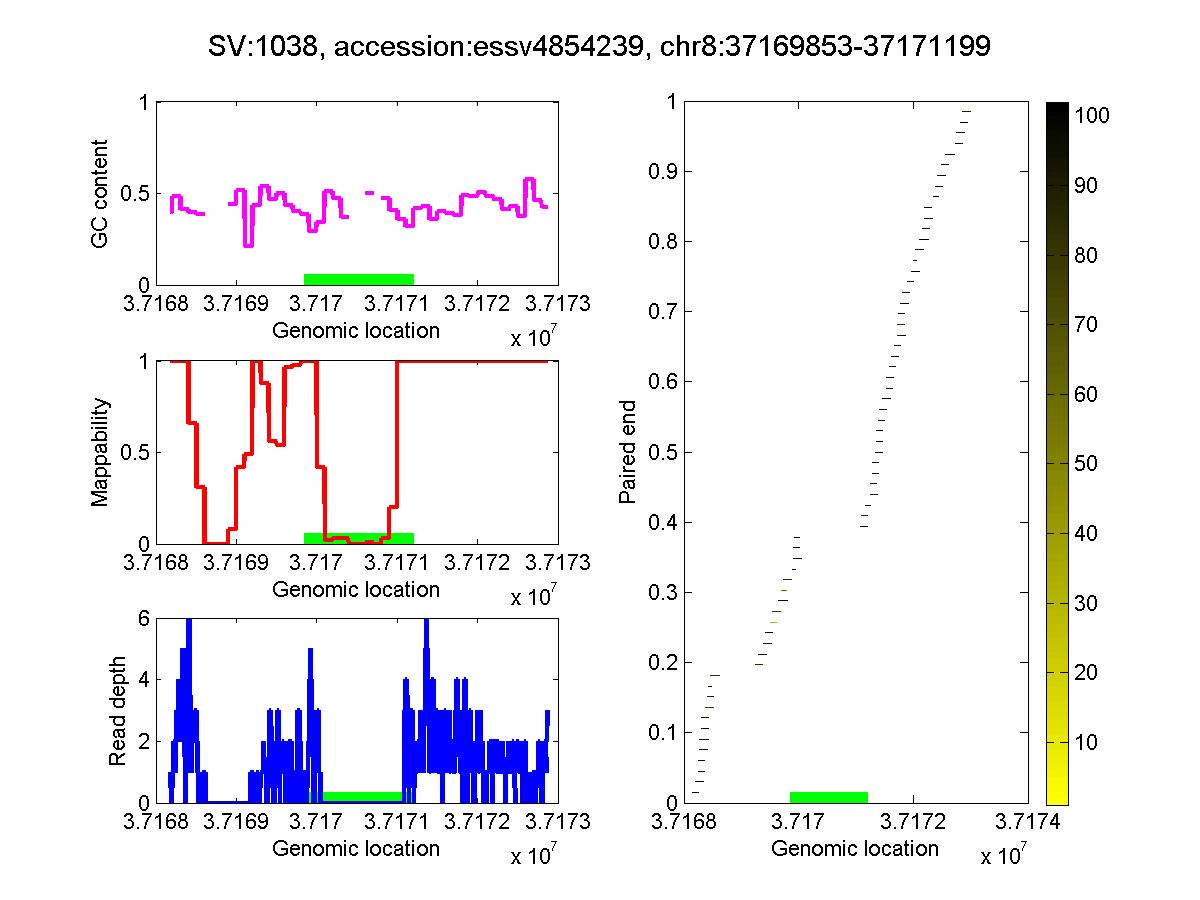

Supplement: Supplementary Materials — Supplementary data are available with this article at http://gr.xjtu.edu.cn/c/document_library/get_file?p_l_id=2403541&folderId=2539941&name=DLFE-115097.zip. Table S1 lists the complete information of suspicious variants and false positives, and the FIG directory contains the validation figures of each false positive. [file 8420547.f1.zip › 8420547.f1/FIG/SV1038.jpg]

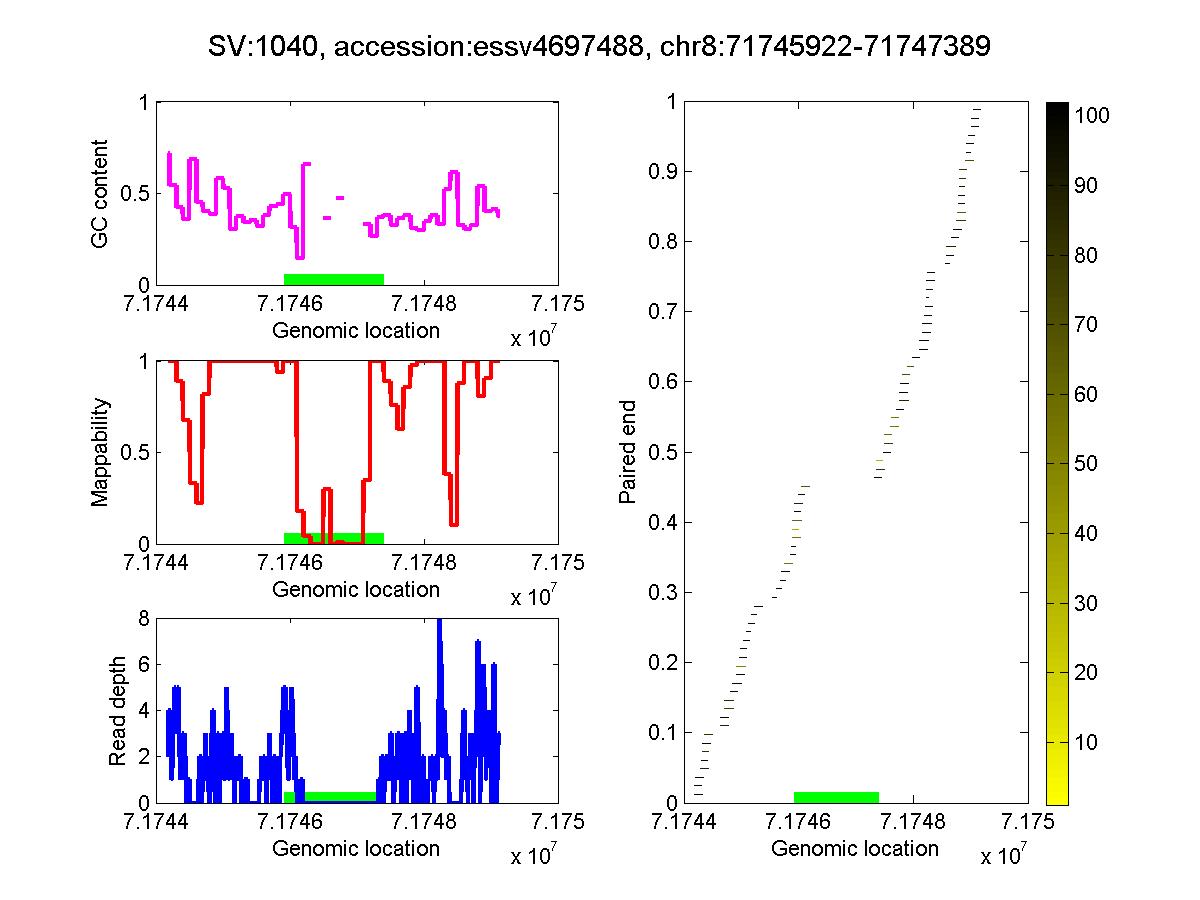

Supplement: Supplementary Materials — Supplementary data are available with this article at http://gr.xjtu.edu.cn/c/document_library/get_file?p_l_id=2403541&folderId=2539941&name=DLFE-115097.zip. Table S1 lists the complete information of suspicious variants and false positives, and the FIG directory contains the validation figures of each false positive. [file 8420547.f1.zip › 8420547.f1/FIG/SV1040.jpg]

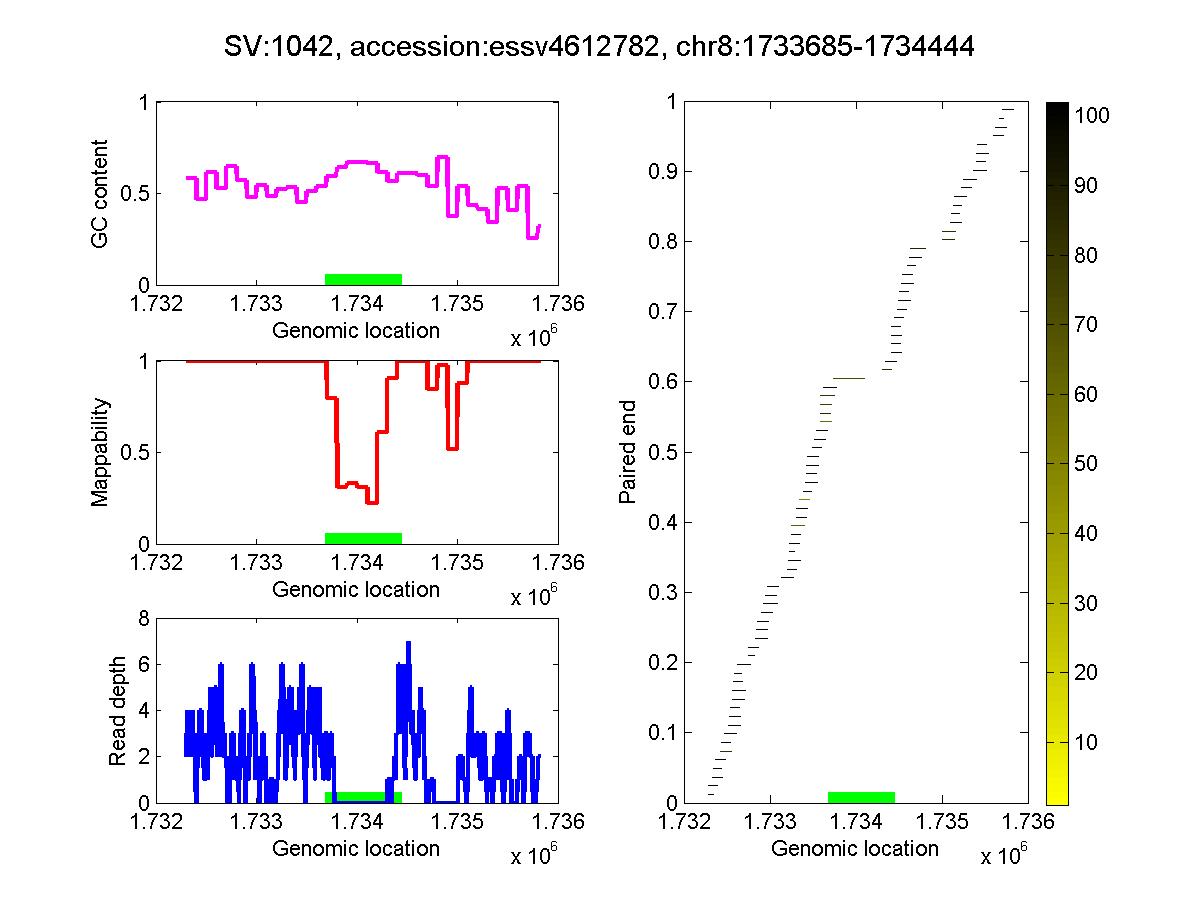

Supplement: Supplementary Materials — Supplementary data are available with this article at http://gr.xjtu.edu.cn/c/document_library/get_file?p_l_id=2403541&folderId=2539941&name=DLFE-115097.zip. Table S1 lists the complete information of suspicious variants and false positives, and the FIG directory contains the validation figures of each false positive. [file 8420547.f1.zip › 8420547.f1/FIG/SV1042.jpg]

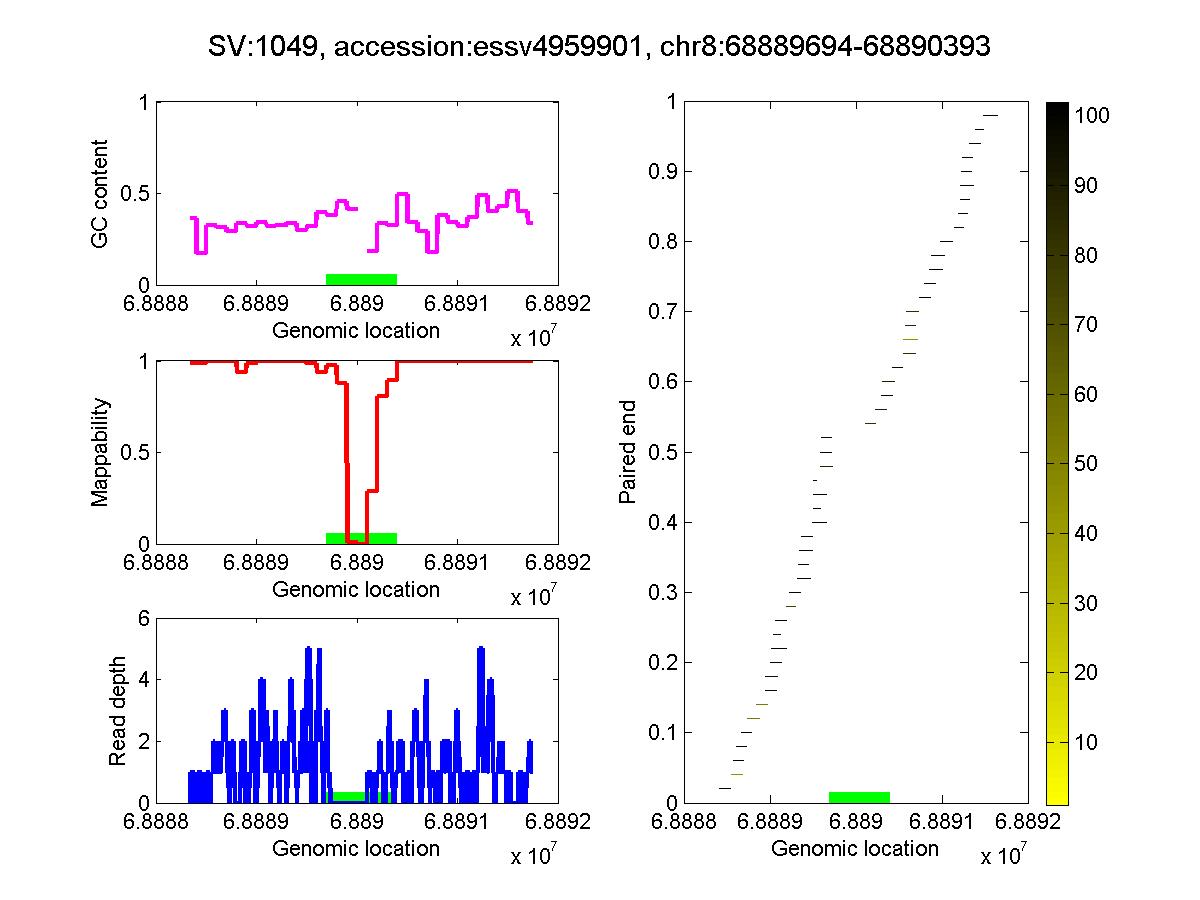

Supplement: Supplementary Materials — Supplementary data are available with this article at http://gr.xjtu.edu.cn/c/document_library/get_file?p_l_id=2403541&folderId=2539941&name=DLFE-115097.zip. Table S1 lists the complete information of suspicious variants and false positives, and the FIG directory contains the validation figures of each false positive. [file 8420547.f1.zip › 8420547.f1/FIG/SV1049.jpg]

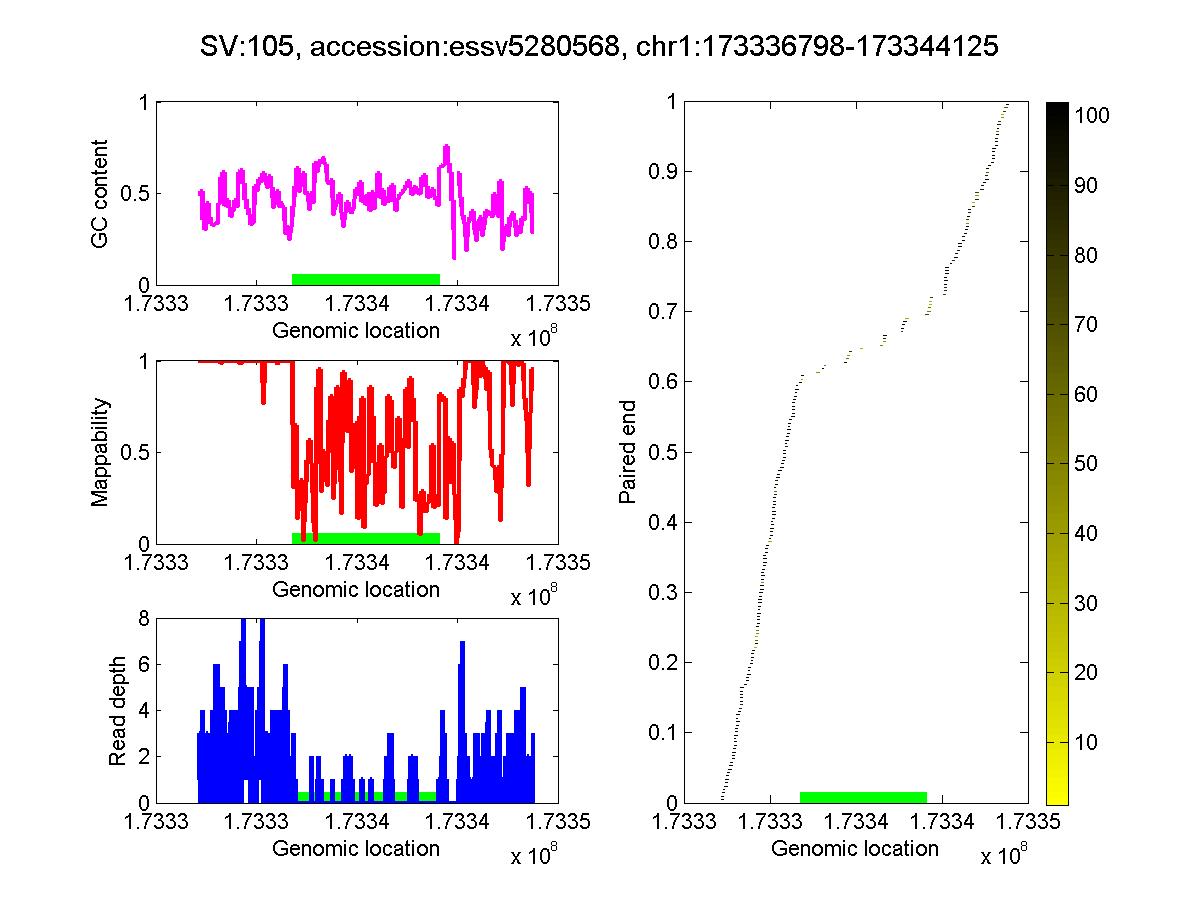

Supplement: Supplementary Materials — Supplementary data are available with this article at http://gr.xjtu.edu.cn/c/document_library/get_file?p_l_id=2403541&folderId=2539941&name=DLFE-115097.zip. Table S1 lists the complete information of suspicious variants and false positives, and the FIG directory contains the validation figures of each false positive. [file 8420547.f1.zip › 8420547.f1/FIG/SV105.jpg]

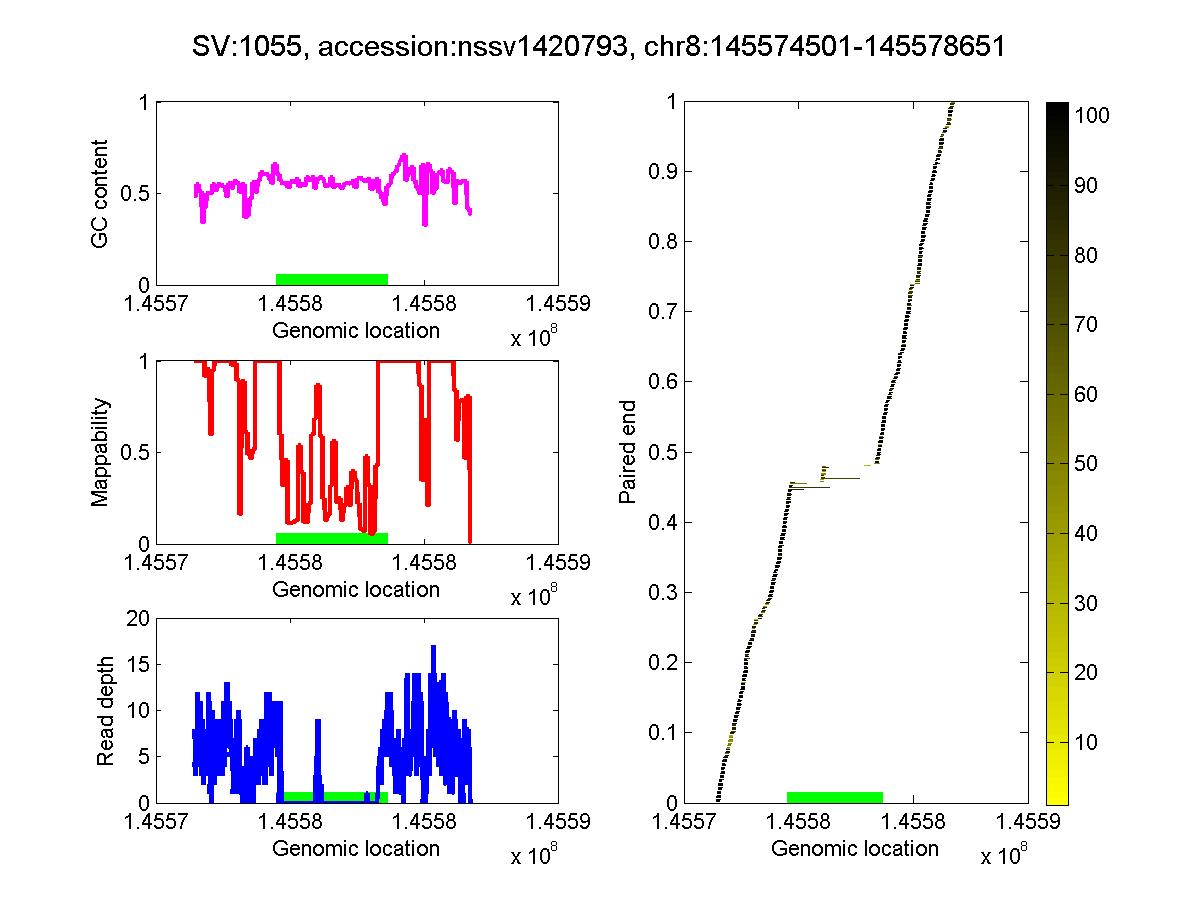

Supplement: Supplementary Materials — Supplementary data are available with this article at http://gr.xjtu.edu.cn/c/document_library/get_file?p_l_id=2403541&folderId=2539941&name=DLFE-115097.zip. Table S1 lists the complete information of suspicious variants and false positives, and the FIG directory contains the validation figures of each false positive. [file 8420547.f1.zip › 8420547.f1/FIG/SV1055.jpg]

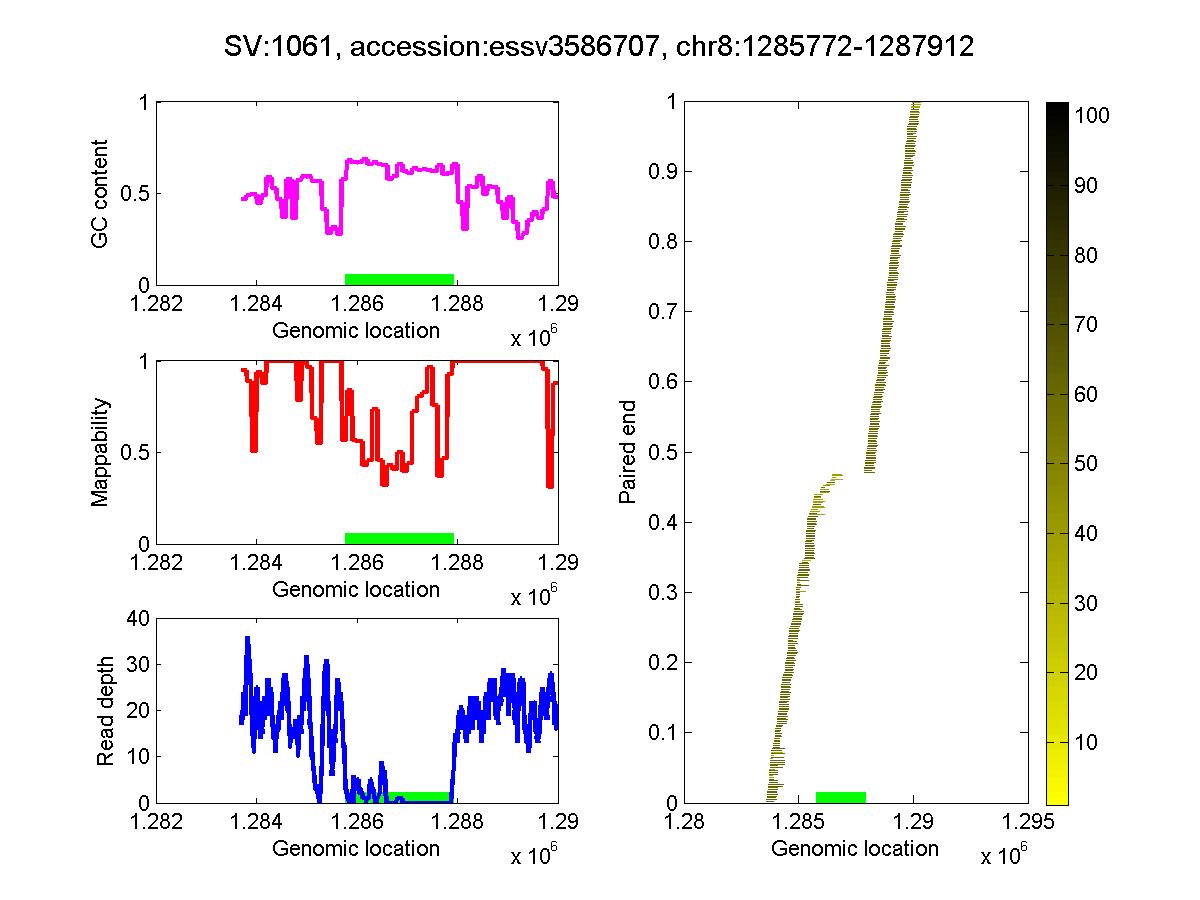

Supplement: Supplementary Materials — Supplementary data are available with this article at http://gr.xjtu.edu.cn/c/document_library/get_file?p_l_id=2403541&folderId=2539941&name=DLFE-115097.zip. Table S1 lists the complete information of suspicious variants and false positives, and the FIG directory contains the validation figures of each false positive. [file 8420547.f1.zip › 8420547.f1/FIG/SV1061.jpg]

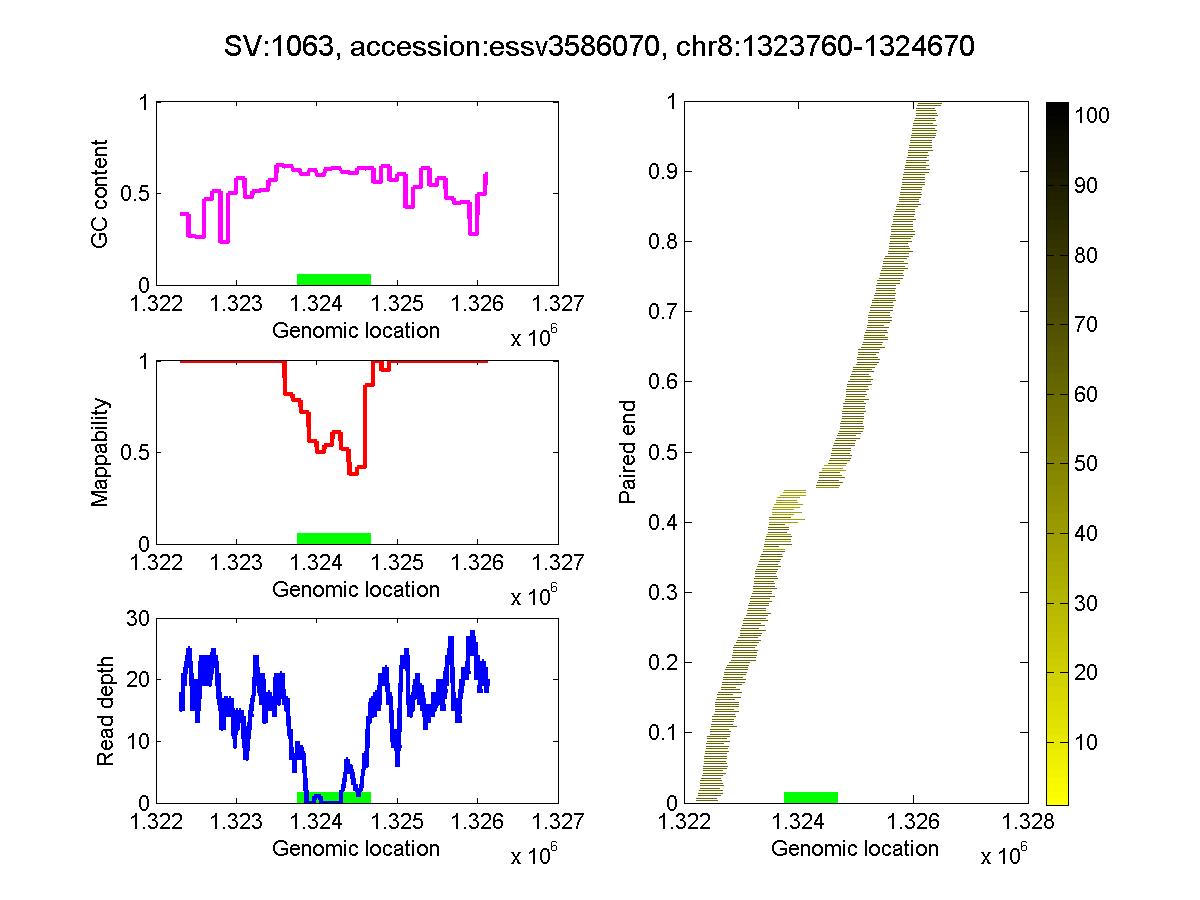

Supplement: Supplementary Materials — Supplementary data are available with this article at http://gr.xjtu.edu.cn/c/document_library/get_file?p_l_id=2403541&folderId=2539941&name=DLFE-115097.zip. Table S1 lists the complete information of suspicious variants and false positives, and the FIG directory contains the validation figures of each false positive. [file 8420547.f1.zip › 8420547.f1/FIG/SV1063.jpg]

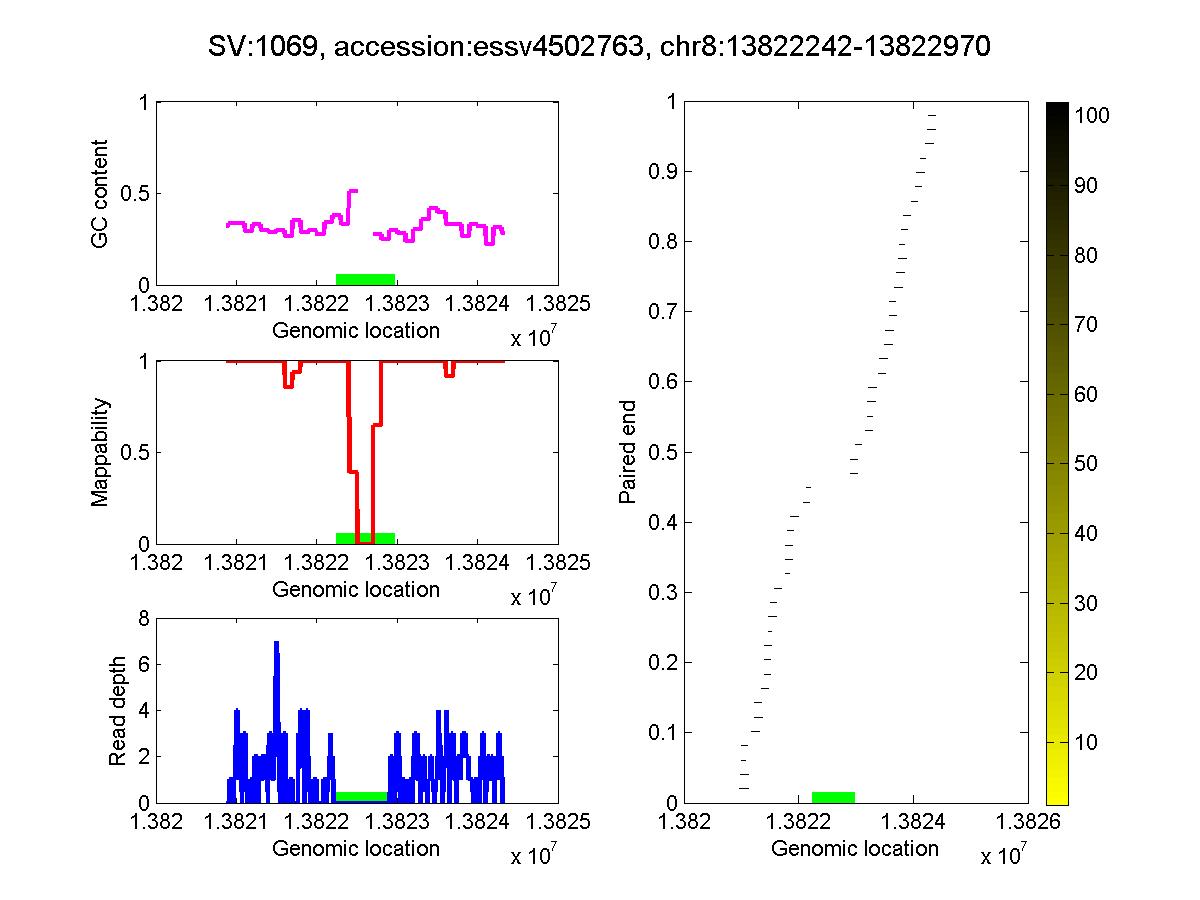

Supplement: Supplementary Materials — Supplementary data are available with this article at http://gr.xjtu.edu.cn/c/document_library/get_file?p_l_id=2403541&folderId=2539941&name=DLFE-115097.zip. Table S1 lists the complete information of suspicious variants and false positives, and the FIG directory contains the validation figures of each false positive. [file 8420547.f1.zip › 8420547.f1/FIG/SV1069.jpg]

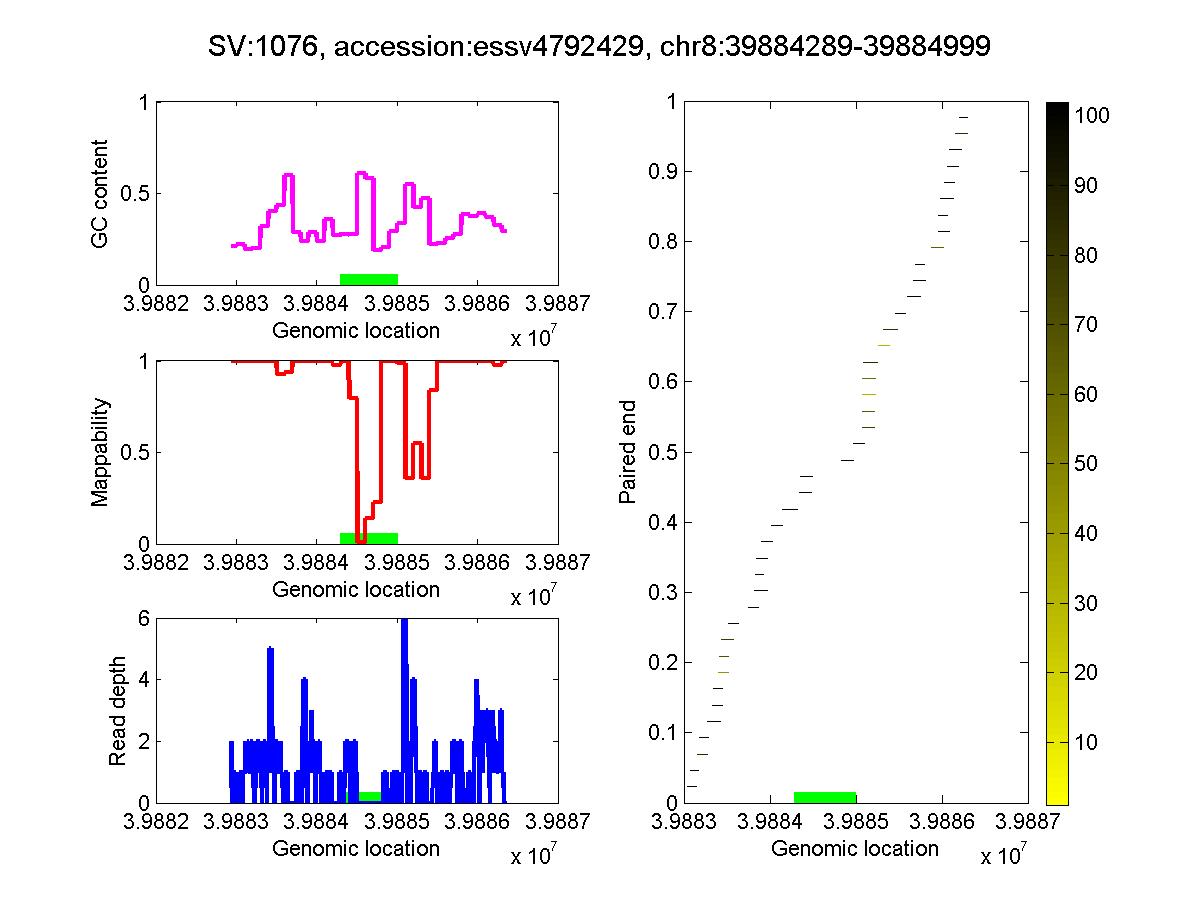

Supplement: Supplementary Materials — Supplementary data are available with this article at http://gr.xjtu.edu.cn/c/document_library/get_file?p_l_id=2403541&folderId=2539941&name=DLFE-115097.zip. Table S1 lists the complete information of suspicious variants and false positives, and the FIG directory contains the validation figures of each false positive. [file 8420547.f1.zip › 8420547.f1/FIG/SV1076.jpg]

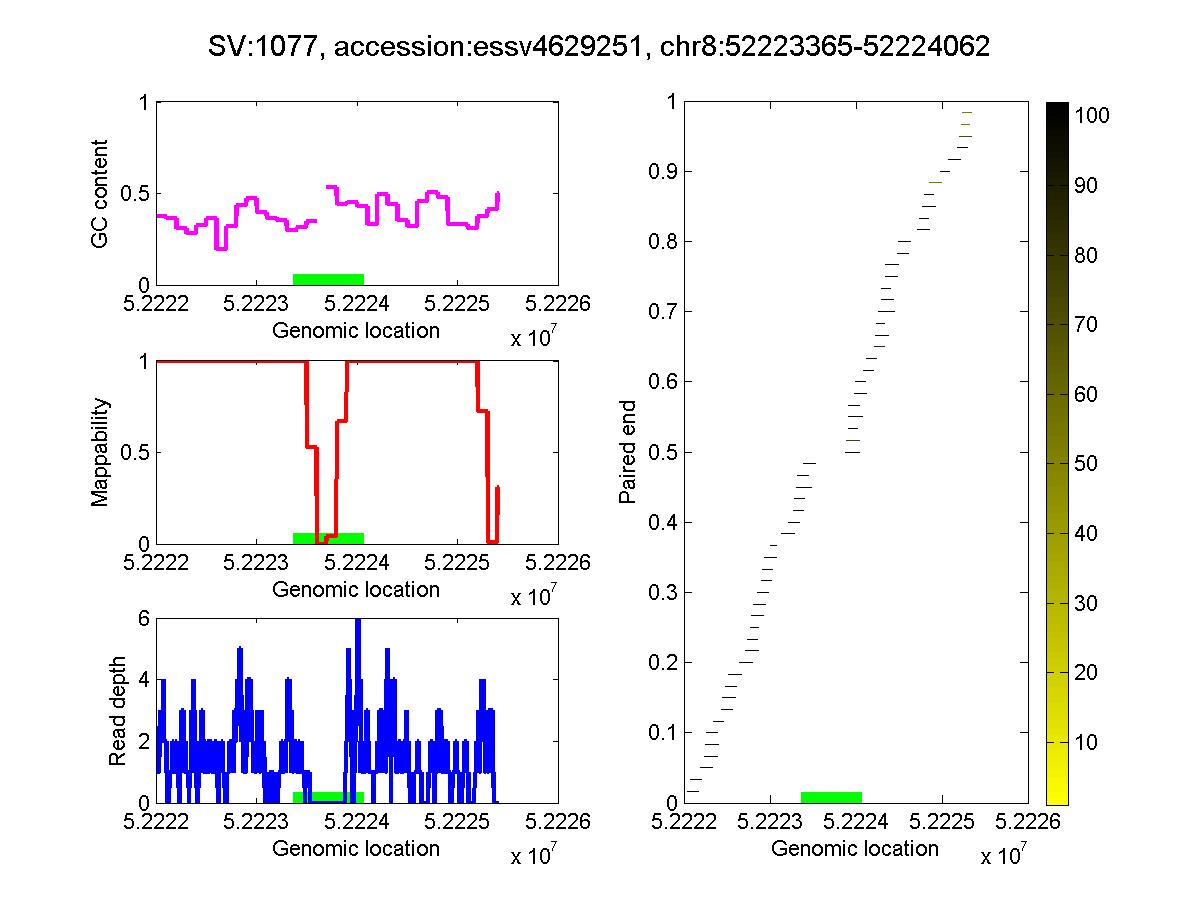

Supplement: Supplementary Materials — Supplementary data are available with this article at http://gr.xjtu.edu.cn/c/document_library/get_file?p_l_id=2403541&folderId=2539941&name=DLFE-115097.zip. Table S1 lists the complete information of suspicious variants and false positives, and the FIG directory contains the validation figures of each false positive. [file 8420547.f1.zip › 8420547.f1/FIG/SV1077.jpg]

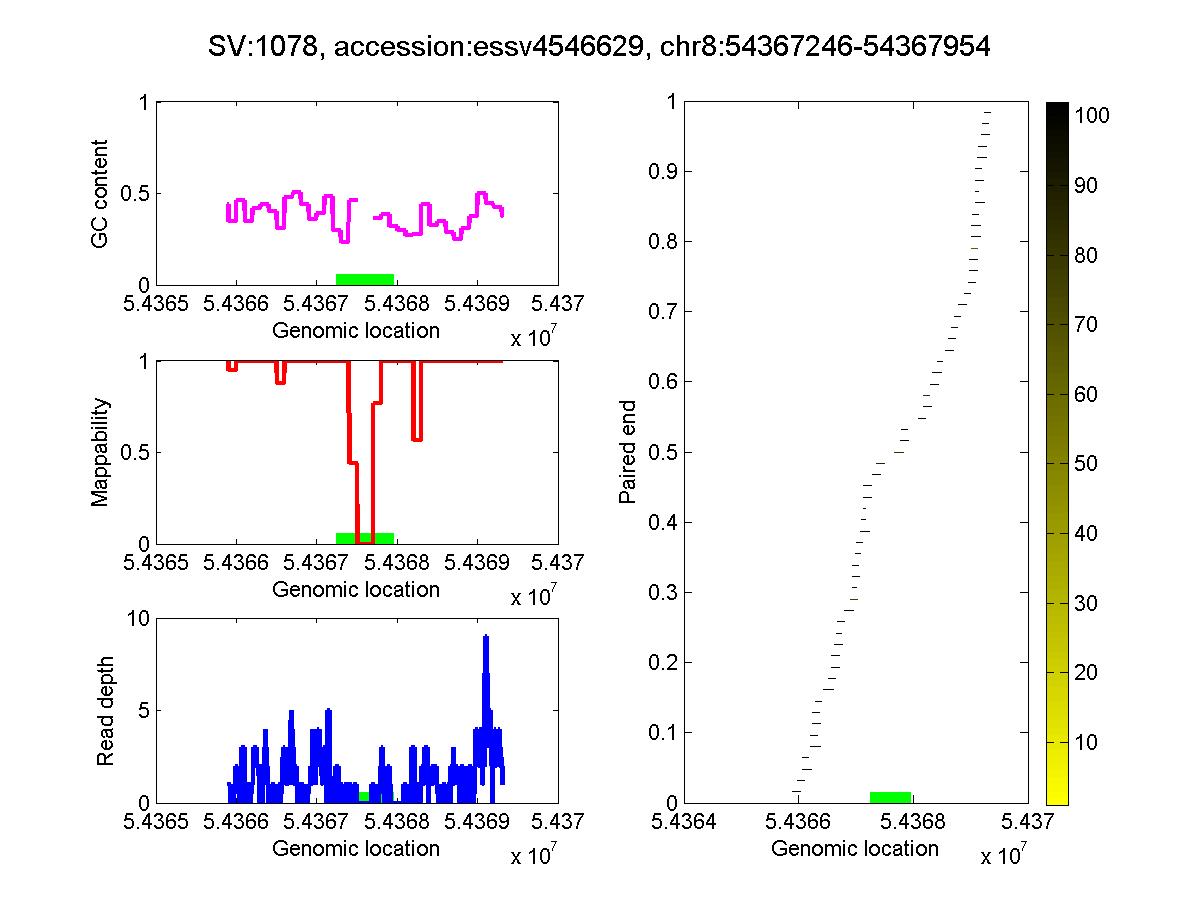

Supplement: Supplementary Materials — Supplementary data are available with this article at http://gr.xjtu.edu.cn/c/document_library/get_file?p_l_id=2403541&folderId=2539941&name=DLFE-115097.zip. Table S1 lists the complete information of suspicious variants and false positives, and the FIG directory contains the validation figures of each false positive. [file 8420547.f1.zip › 8420547.f1/FIG/SV1078.jpg]

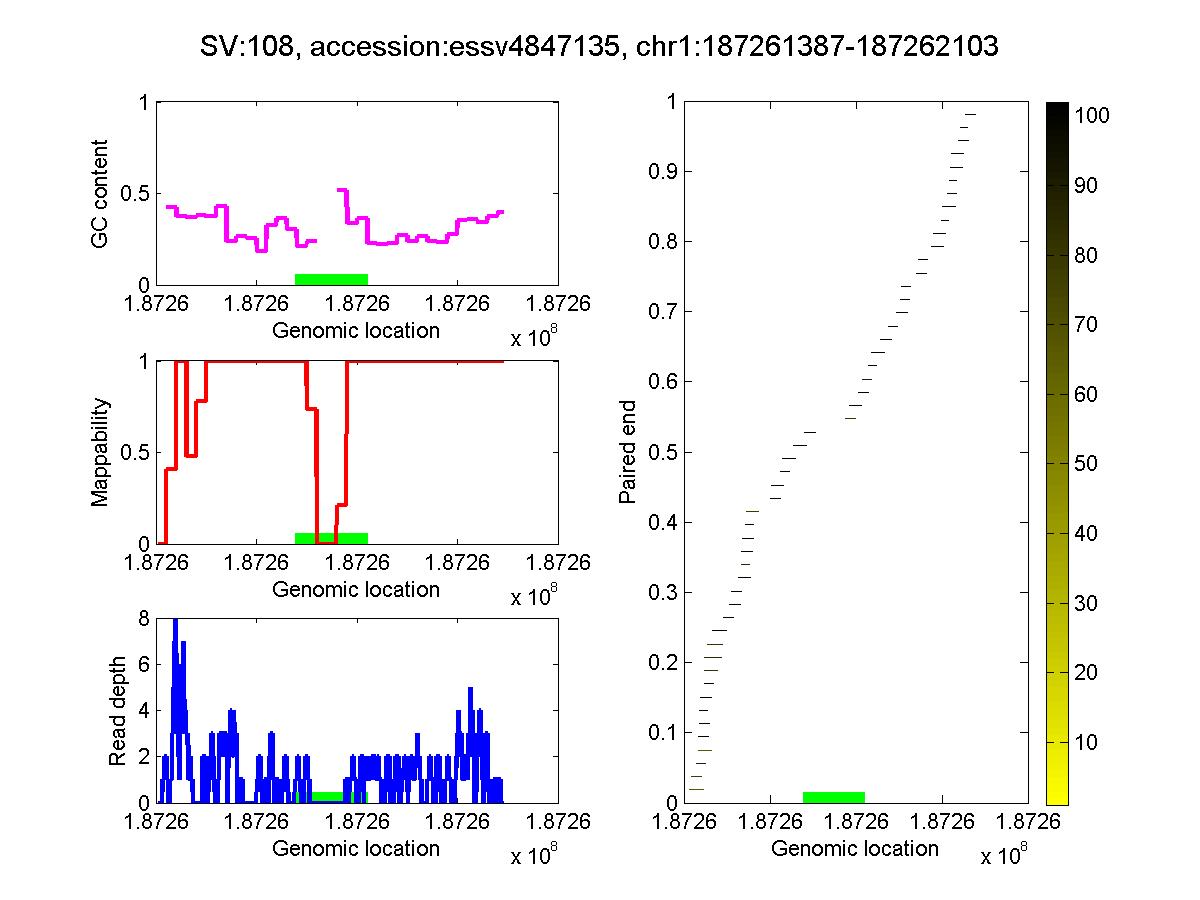

Supplement: Supplementary Materials — Supplementary data are available with this article at http://gr.xjtu.edu.cn/c/document_library/get_file?p_l_id=2403541&folderId=2539941&name=DLFE-115097.zip. Table S1 lists the complete information of suspicious variants and false positives, and the FIG directory contains the validation figures of each false positive. [file 8420547.f1.zip › 8420547.f1/FIG/SV108.jpg]

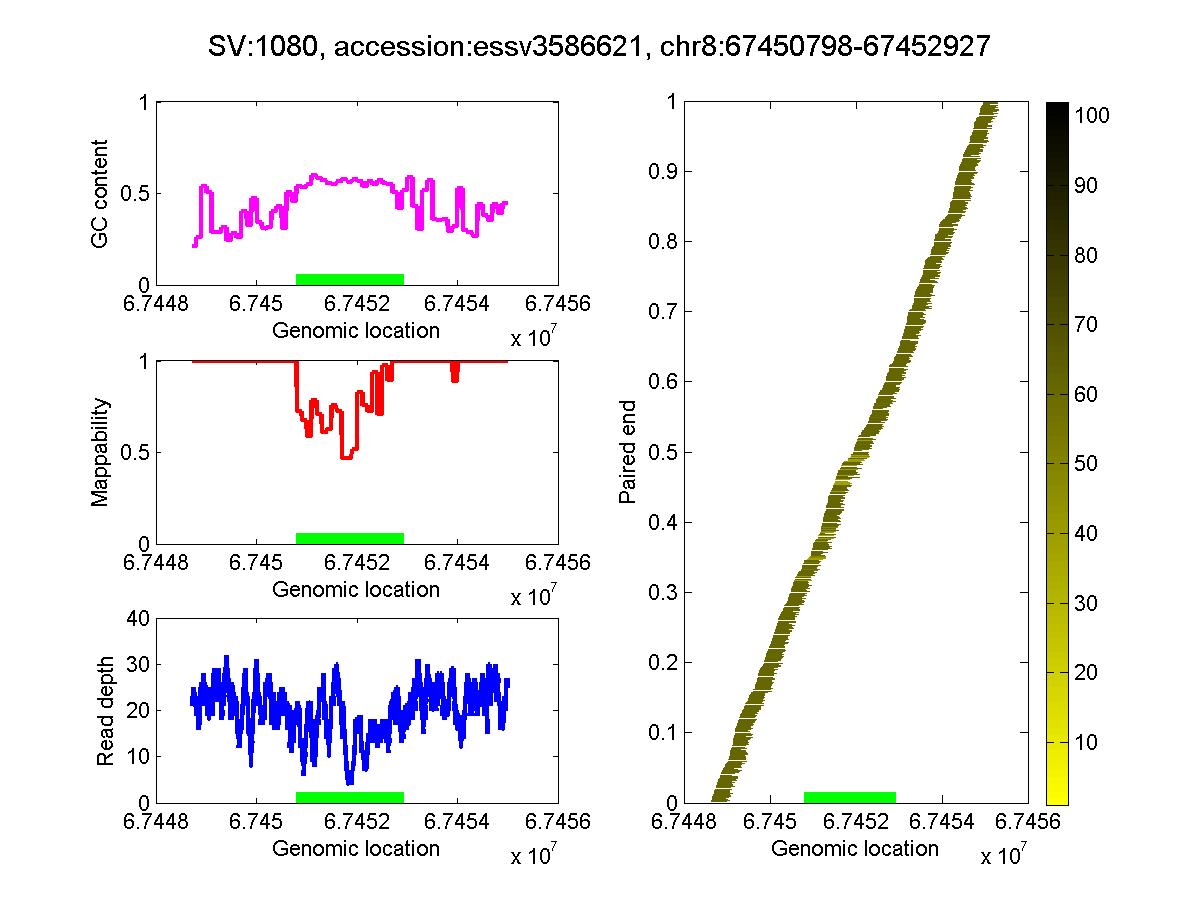

Supplement: Supplementary Materials — Supplementary data are available with this article at http://gr.xjtu.edu.cn/c/document_library/get_file?p_l_id=2403541&folderId=2539941&name=DLFE-115097.zip. Table S1 lists the complete information of suspicious variants and false positives, and the FIG directory contains the validation figures of each false positive. [file 8420547.f1.zip › 8420547.f1/FIG/SV1080.jpg]

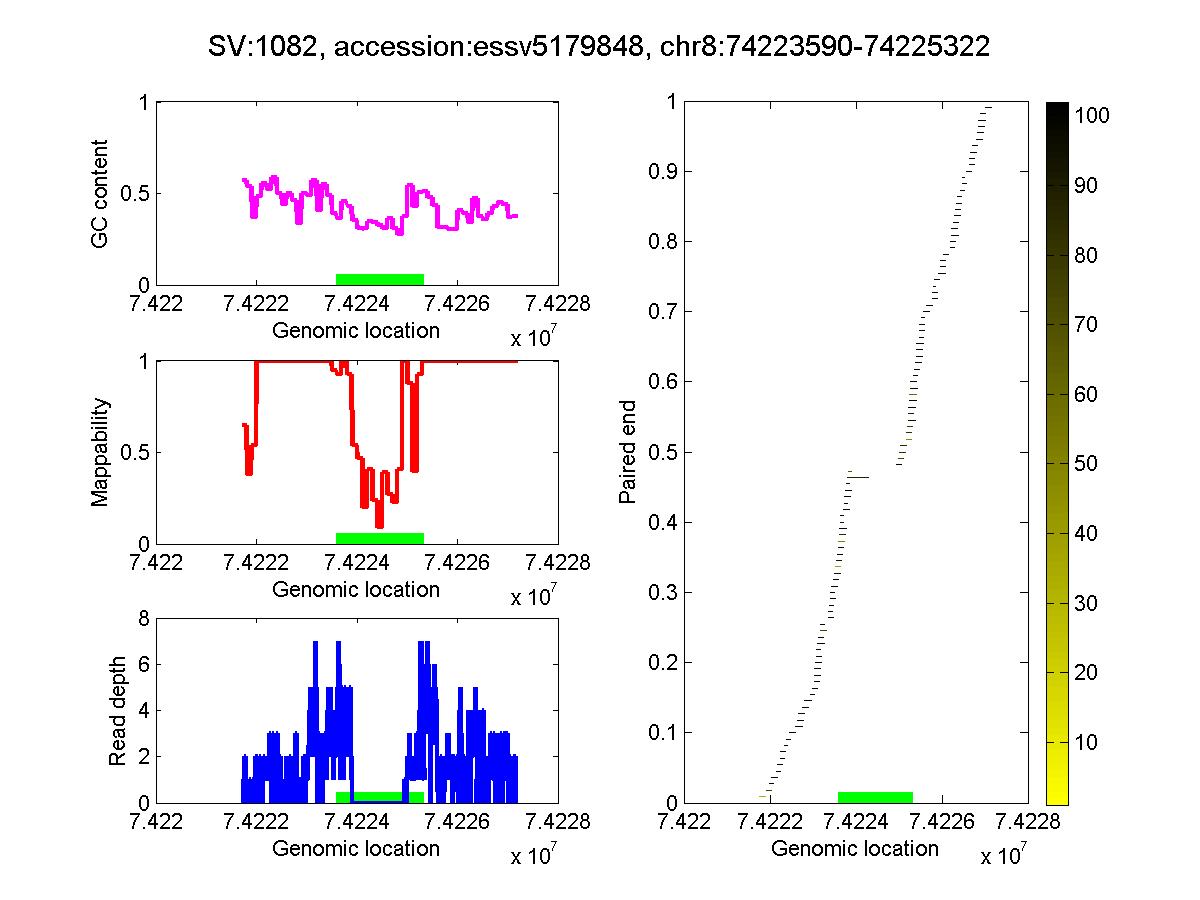

Supplement: Supplementary Materials — Supplementary data are available with this article at http://gr.xjtu.edu.cn/c/document_library/get_file?p_l_id=2403541&folderId=2539941&name=DLFE-115097.zip. Table S1 lists the complete information of suspicious variants and false positives, and the FIG directory contains the validation figures of each false positive. [file 8420547.f1.zip › 8420547.f1/FIG/SV1082.jpg]

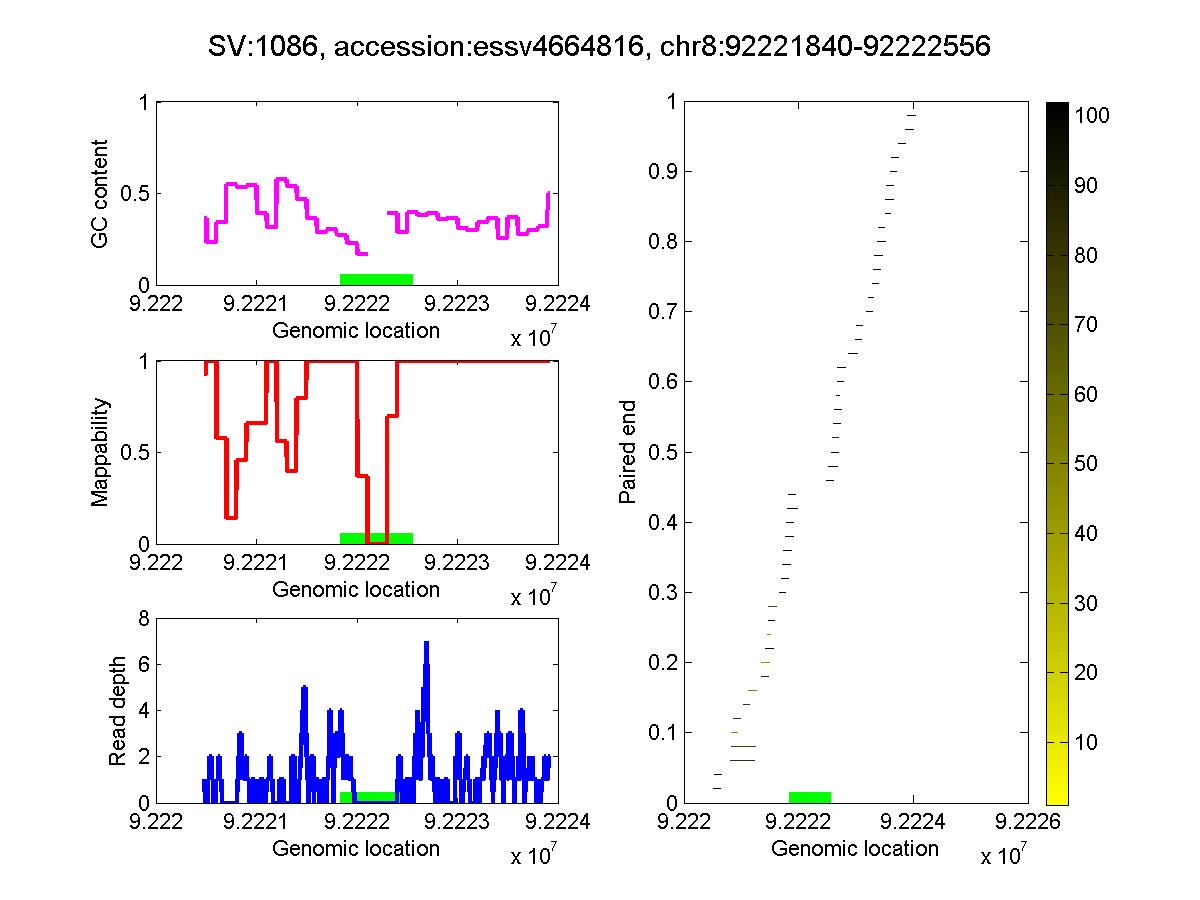

Supplement: Supplementary Materials — Supplementary data are available with this article at http://gr.xjtu.edu.cn/c/document_library/get_file?p_l_id=2403541&folderId=2539941&name=DLFE-115097.zip. Table S1 lists the complete information of suspicious variants and false positives, and the FIG directory contains the validation figures of each false positive. [file 8420547.f1.zip › 8420547.f1/FIG/SV1086.jpg]

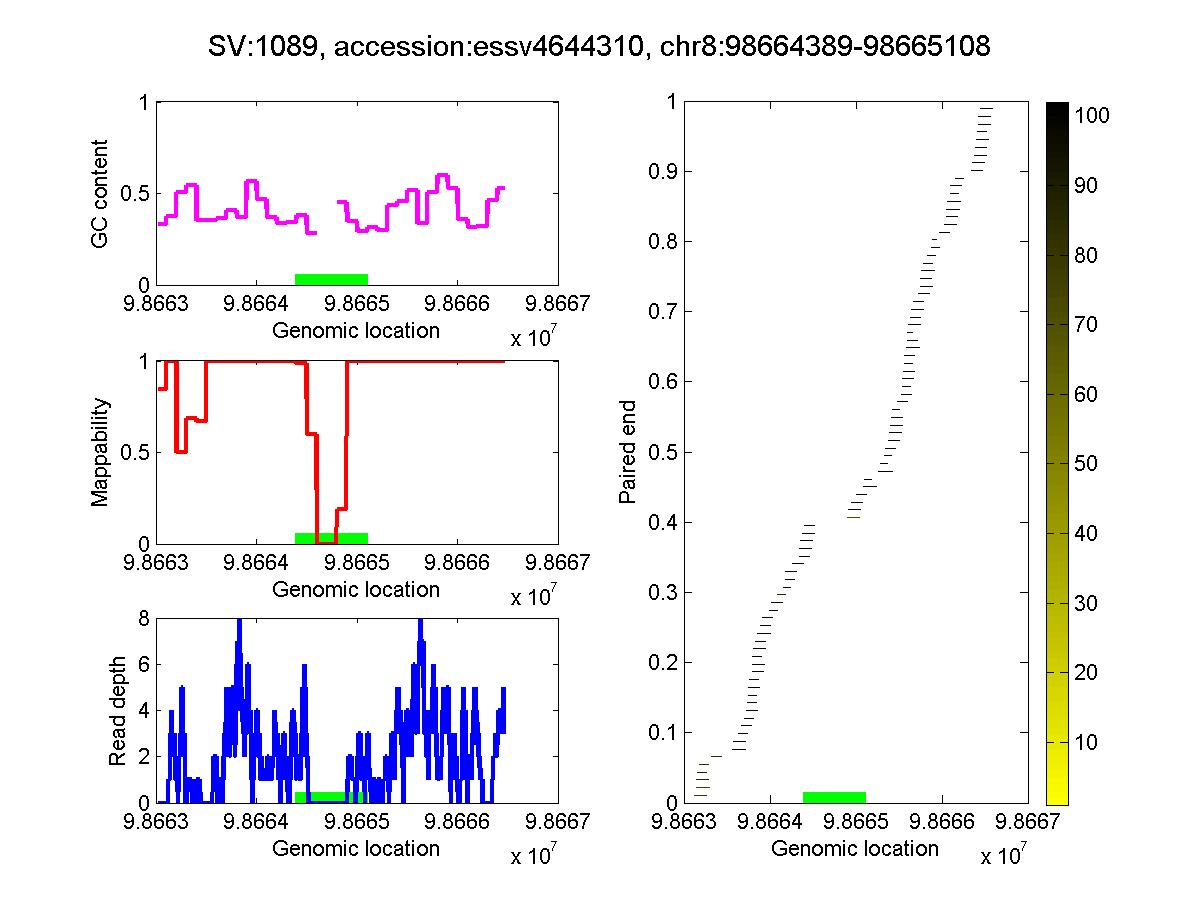

Supplement: Supplementary Materials — Supplementary data are available with this article at http://gr.xjtu.edu.cn/c/document_library/get_file?p_l_id=2403541&folderId=2539941&name=DLFE-115097.zip. Table S1 lists the complete information of suspicious variants and false positives, and the FIG directory contains the validation figures of each false positive. [file 8420547.f1.zip › 8420547.f1/FIG/SV1089.jpg]

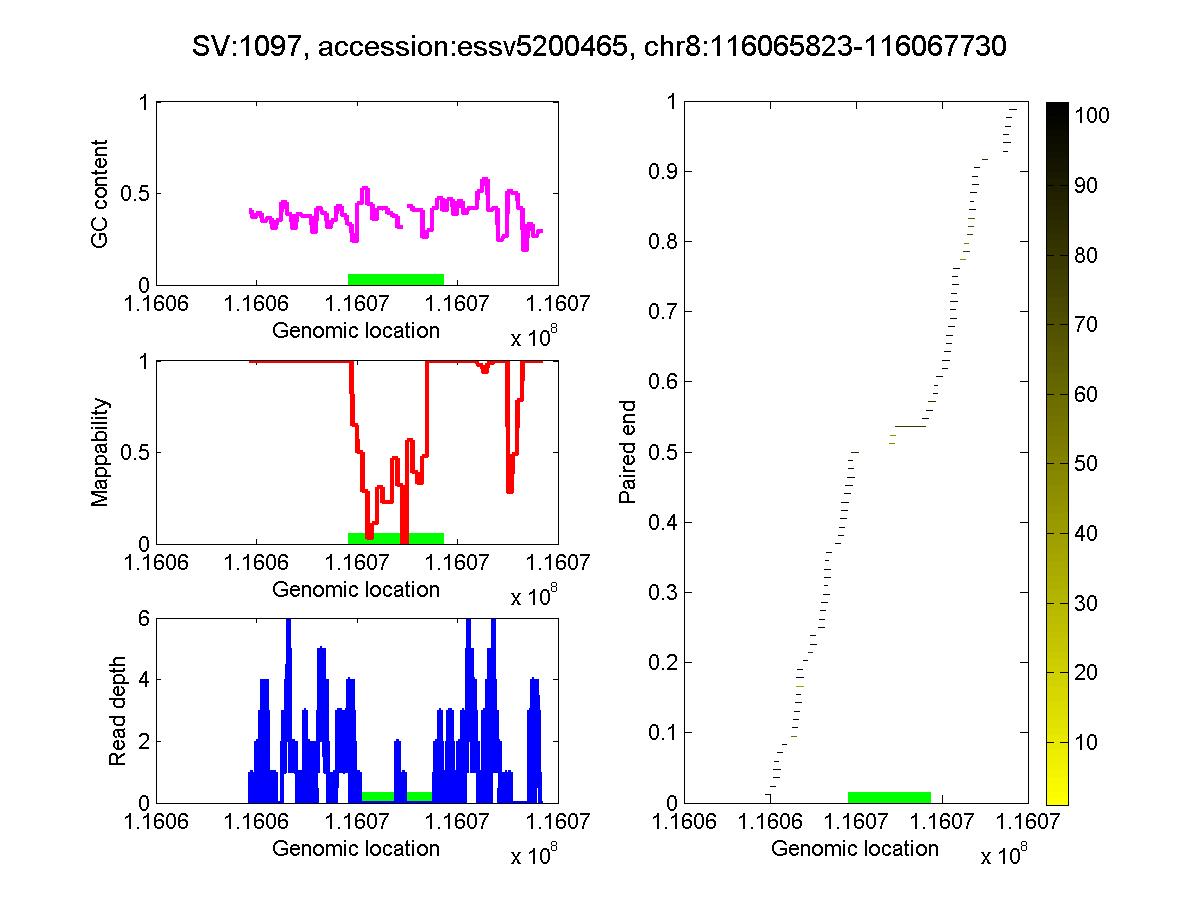

Supplement: Supplementary Materials — Supplementary data are available with this article at http://gr.xjtu.edu.cn/c/document_library/get_file?p_l_id=2403541&folderId=2539941&name=DLFE-115097.zip. Table S1 lists the complete information of suspicious variants and false positives, and the FIG directory contains the validation figures of each false positive. [file 8420547.f1.zip › 8420547.f1/FIG/SV1097.jpg]

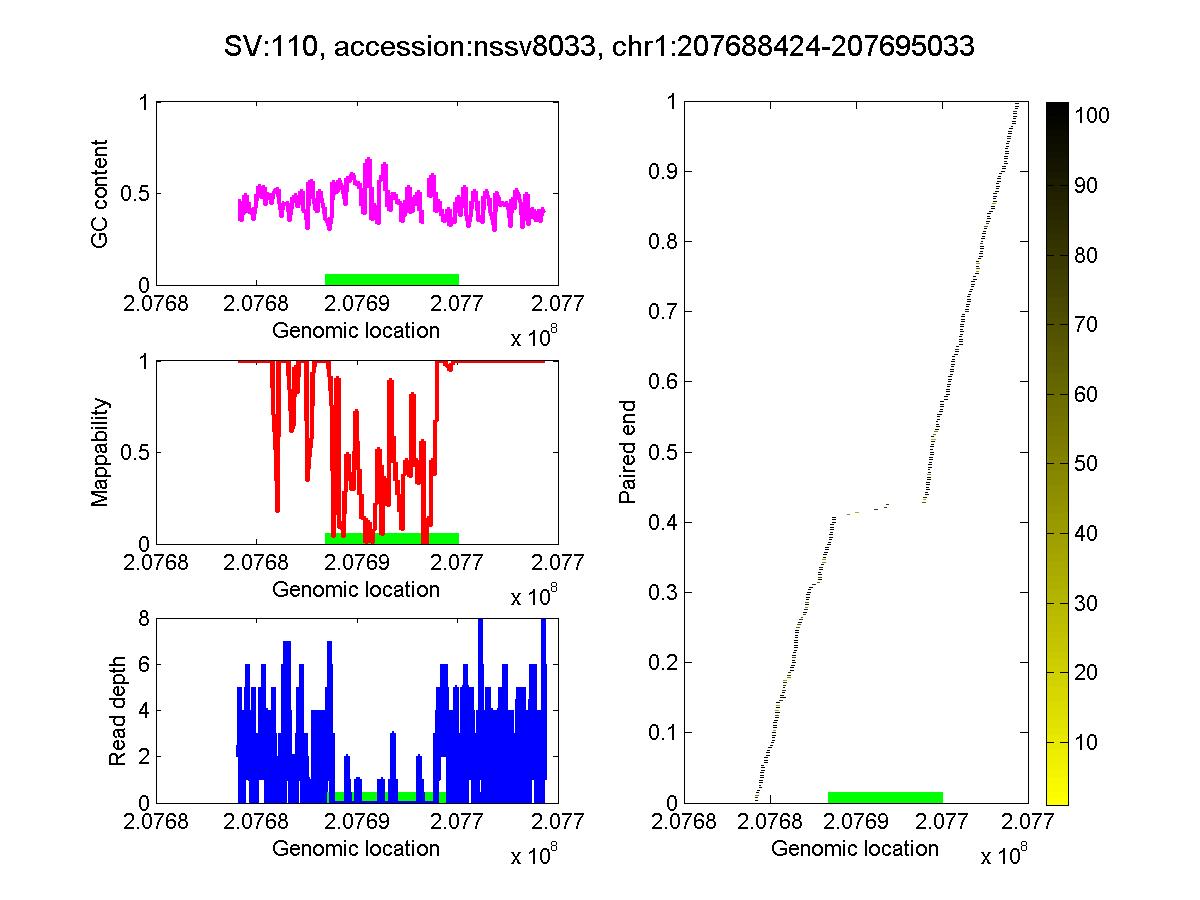

Supplement: Supplementary Materials — Supplementary data are available with this article at http://gr.xjtu.edu.cn/c/document_library/get_file?p_l_id=2403541&folderId=2539941&name=DLFE-115097.zip. Table S1 lists the complete information of suspicious variants and false positives, and the FIG directory contains the validation figures of each false positive. [file 8420547.f1.zip › 8420547.f1/FIG/SV110.jpg]

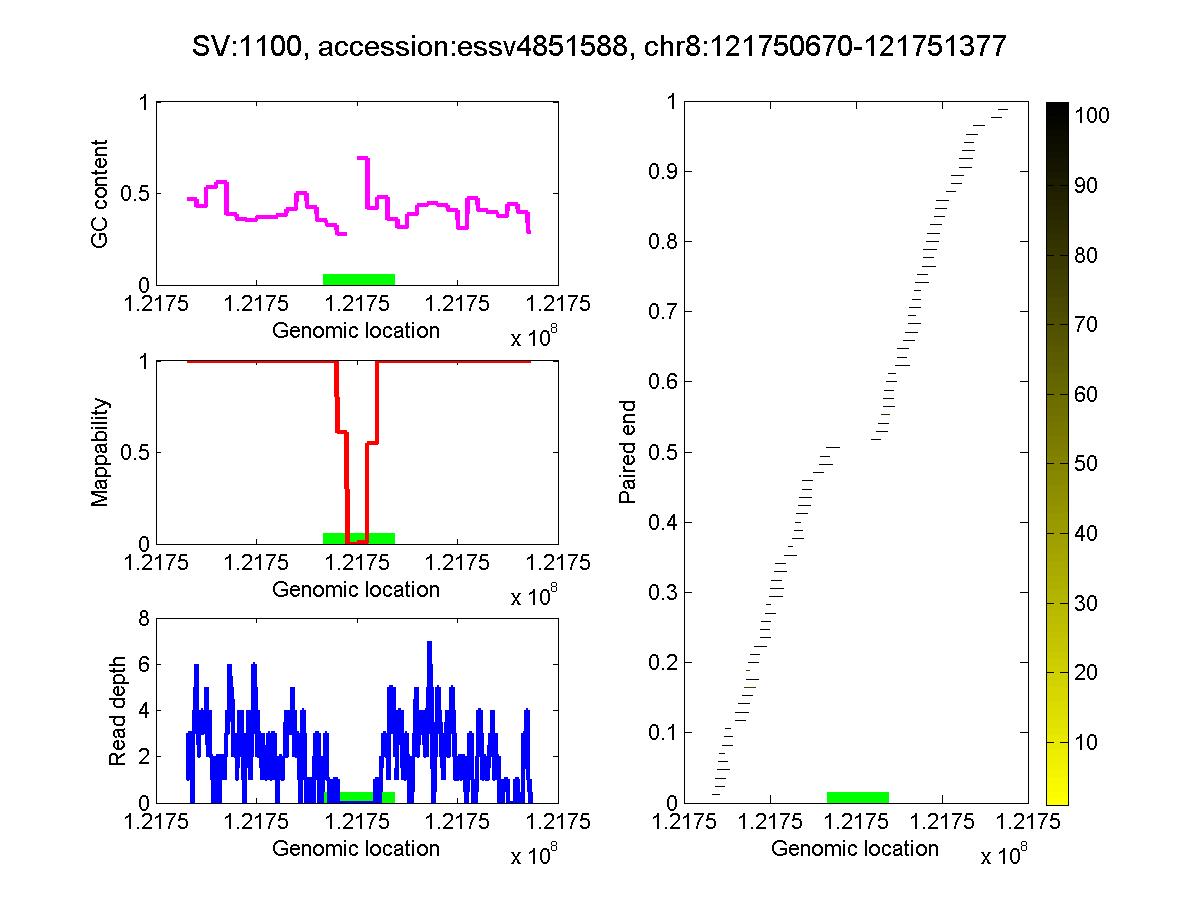

Supplement: Supplementary Materials — Supplementary data are available with this article at http://gr.xjtu.edu.cn/c/document_library/get_file?p_l_id=2403541&folderId=2539941&name=DLFE-115097.zip. Table S1 lists the complete information of suspicious variants and false positives, and the FIG directory contains the validation figures of each false positive. [file 8420547.f1.zip › 8420547.f1/FIG/SV1100.jpg]

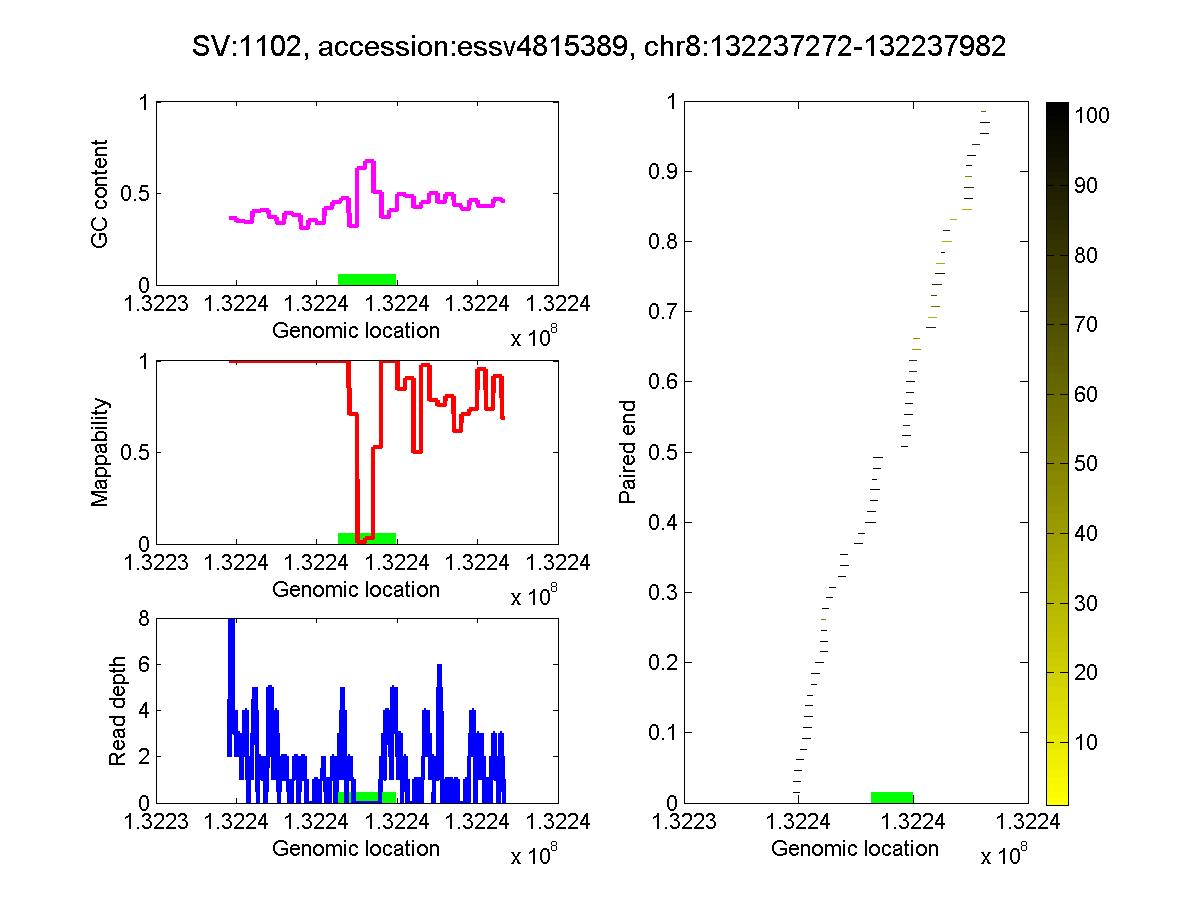

Supplement: Supplementary Materials — Supplementary data are available with this article at http://gr.xjtu.edu.cn/c/document_library/get_file?p_l_id=2403541&folderId=2539941&name=DLFE-115097.zip. Table S1 lists the complete information of suspicious variants and false positives, and the FIG directory contains the validation figures of each false positive. [file 8420547.f1.zip › 8420547.f1/FIG/SV1102.jpg]

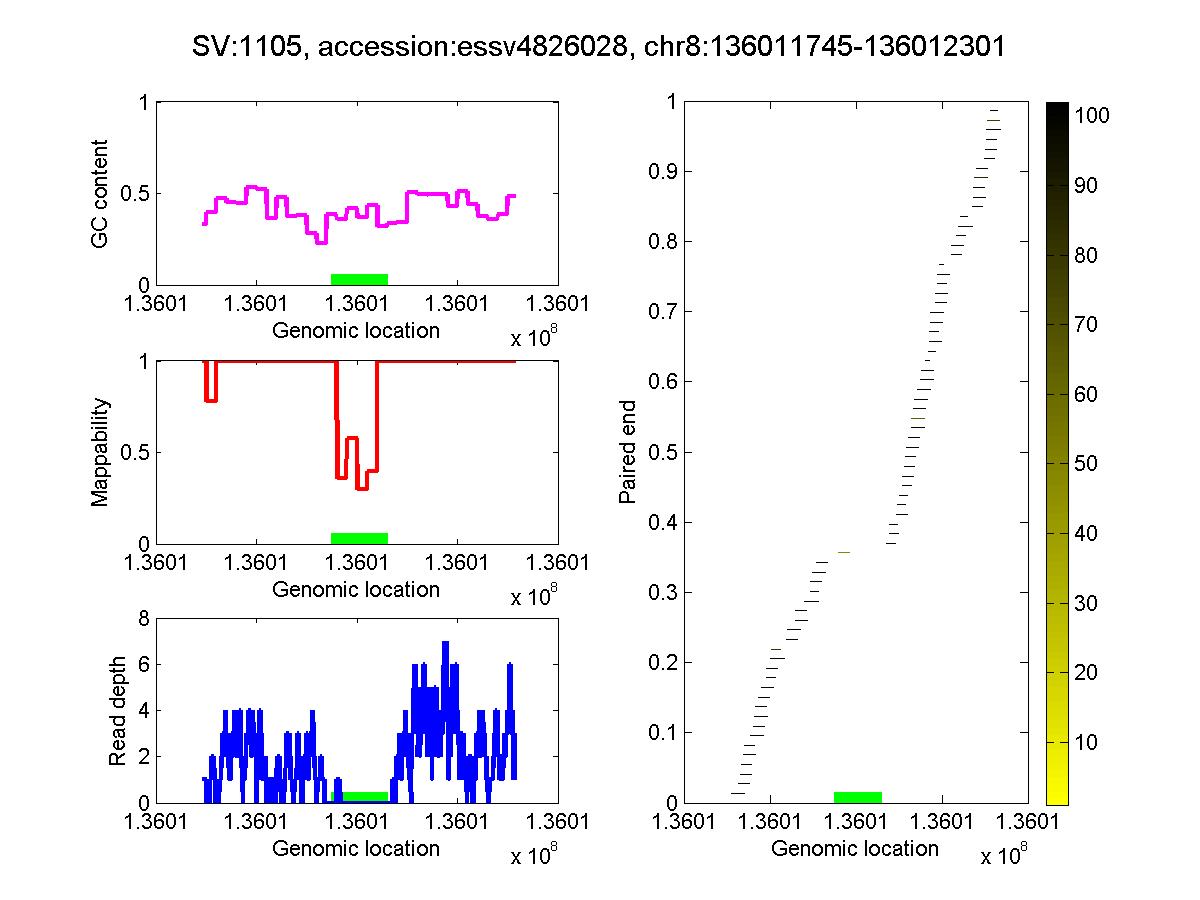

Supplement: Supplementary Materials — Supplementary data are available with this article at http://gr.xjtu.edu.cn/c/document_library/get_file?p_l_id=2403541&folderId=2539941&name=DLFE-115097.zip. Table S1 lists the complete information of suspicious variants and false positives, and the FIG directory contains the validation figures of each false positive. [file 8420547.f1.zip › 8420547.f1/FIG/SV1105.jpg]

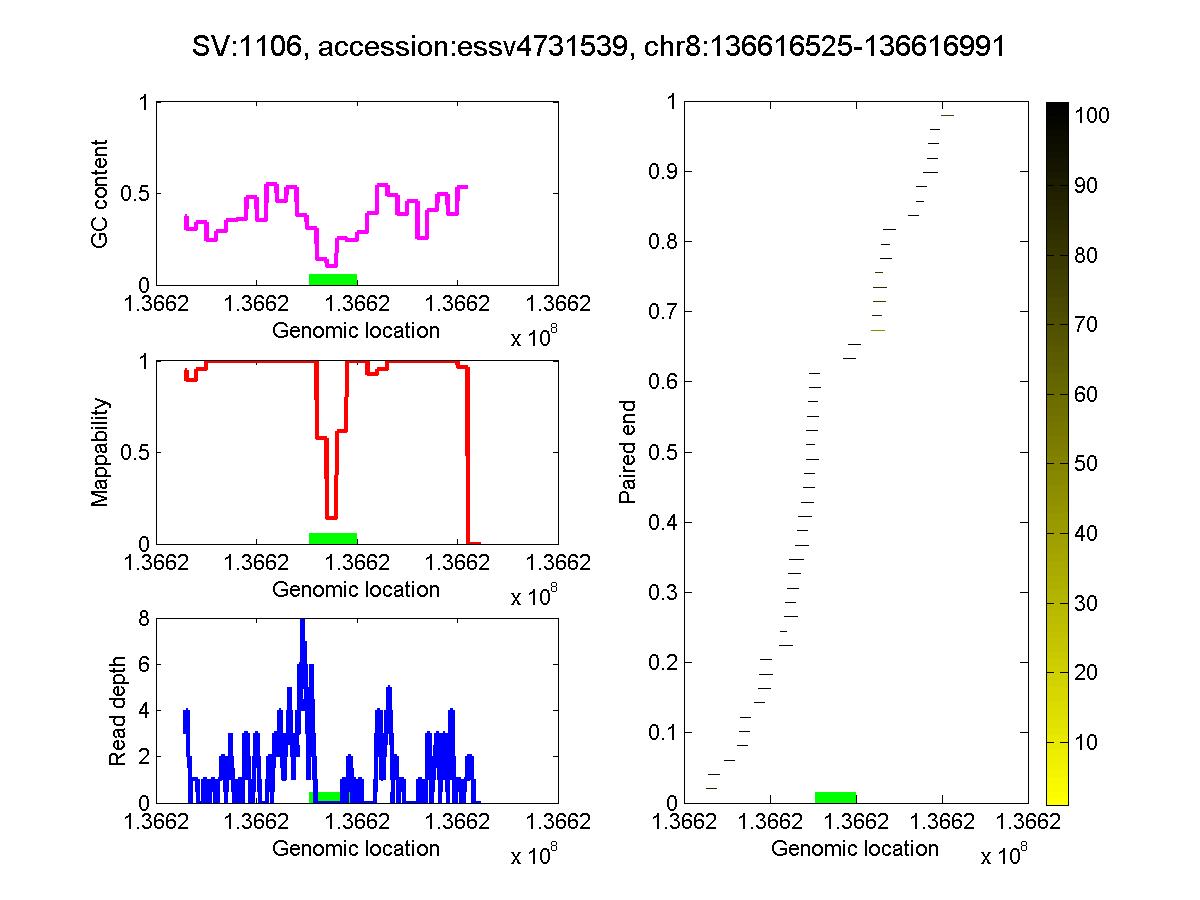

Supplement: Supplementary Materials — Supplementary data are available with this article at http://gr.xjtu.edu.cn/c/document_library/get_file?p_l_id=2403541&folderId=2539941&name=DLFE-115097.zip. Table S1 lists the complete information of suspicious variants and false positives, and the FIG directory contains the validation figures of each false positive. [file 8420547.f1.zip › 8420547.f1/FIG/SV1106.jpg]

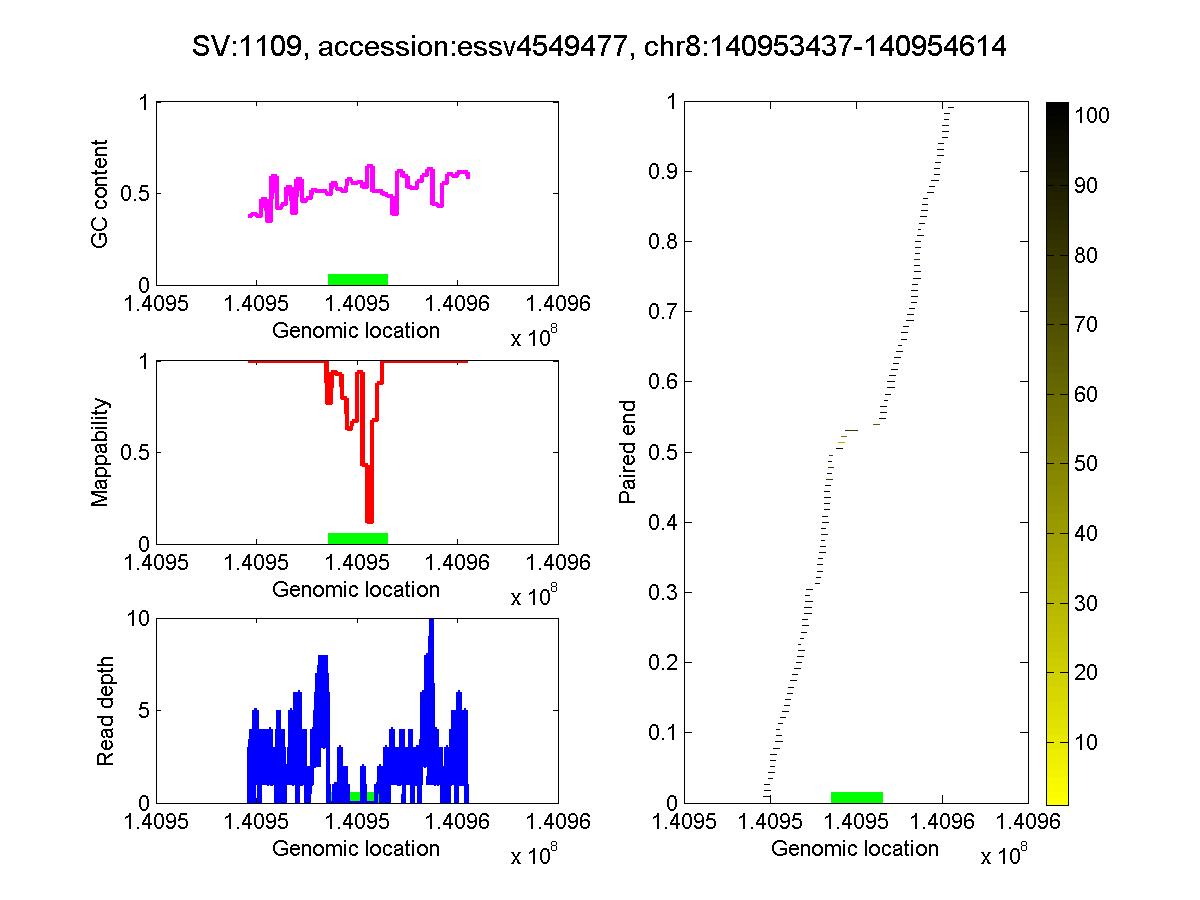

Supplement: Supplementary Materials — Supplementary data are available with this article at http://gr.xjtu.edu.cn/c/document_library/get_file?p_l_id=2403541&folderId=2539941&name=DLFE-115097.zip. Table S1 lists the complete information of suspicious variants and false positives, and the FIG directory contains the validation figures of each false positive. [file 8420547.f1.zip › 8420547.f1/FIG/SV1109.jpg]

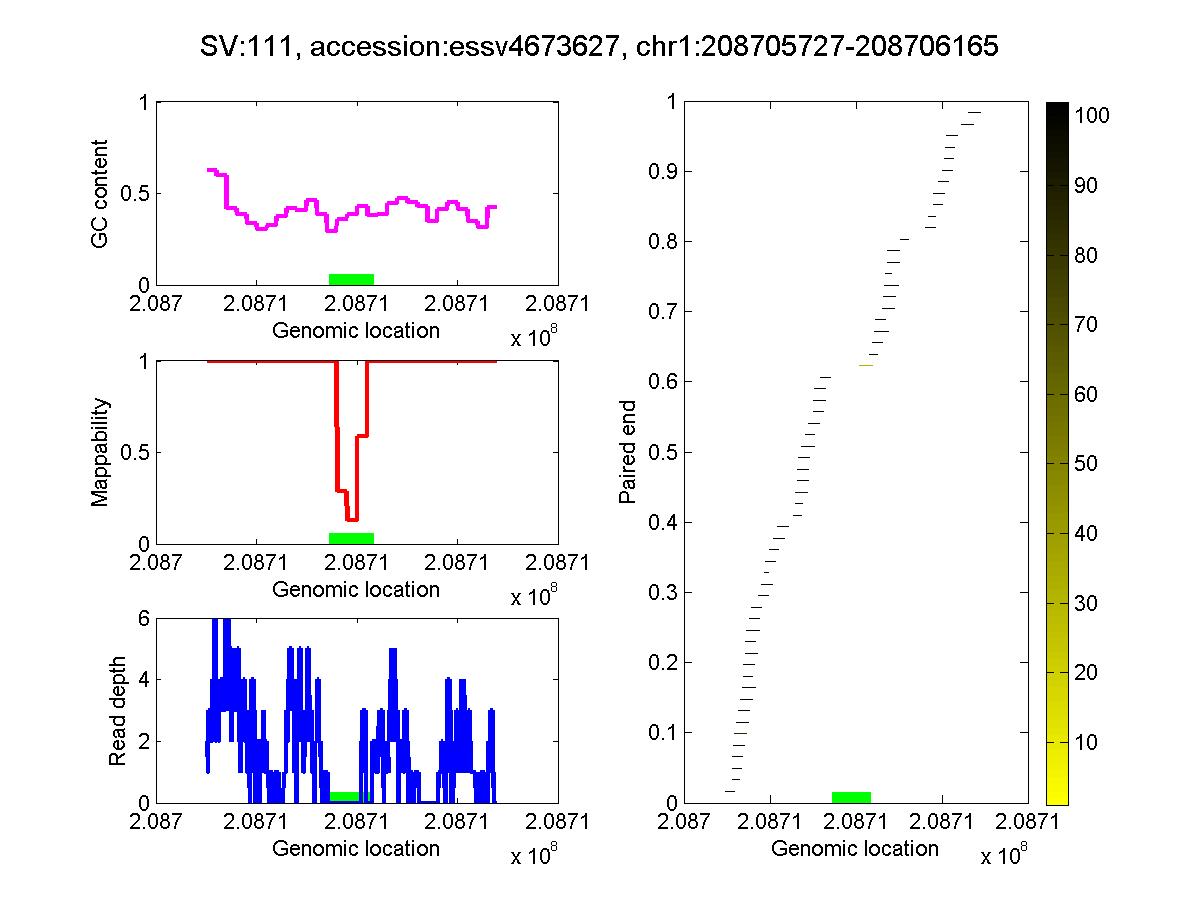

Supplement: Supplementary Materials — Supplementary data are available with this article at http://gr.xjtu.edu.cn/c/document_library/get_file?p_l_id=2403541&folderId=2539941&name=DLFE-115097.zip. Table S1 lists the complete information of suspicious variants and false positives, and the FIG directory contains the validation figures of each false positive. [file 8420547.f1.zip › 8420547.f1/FIG/SV111.jpg]

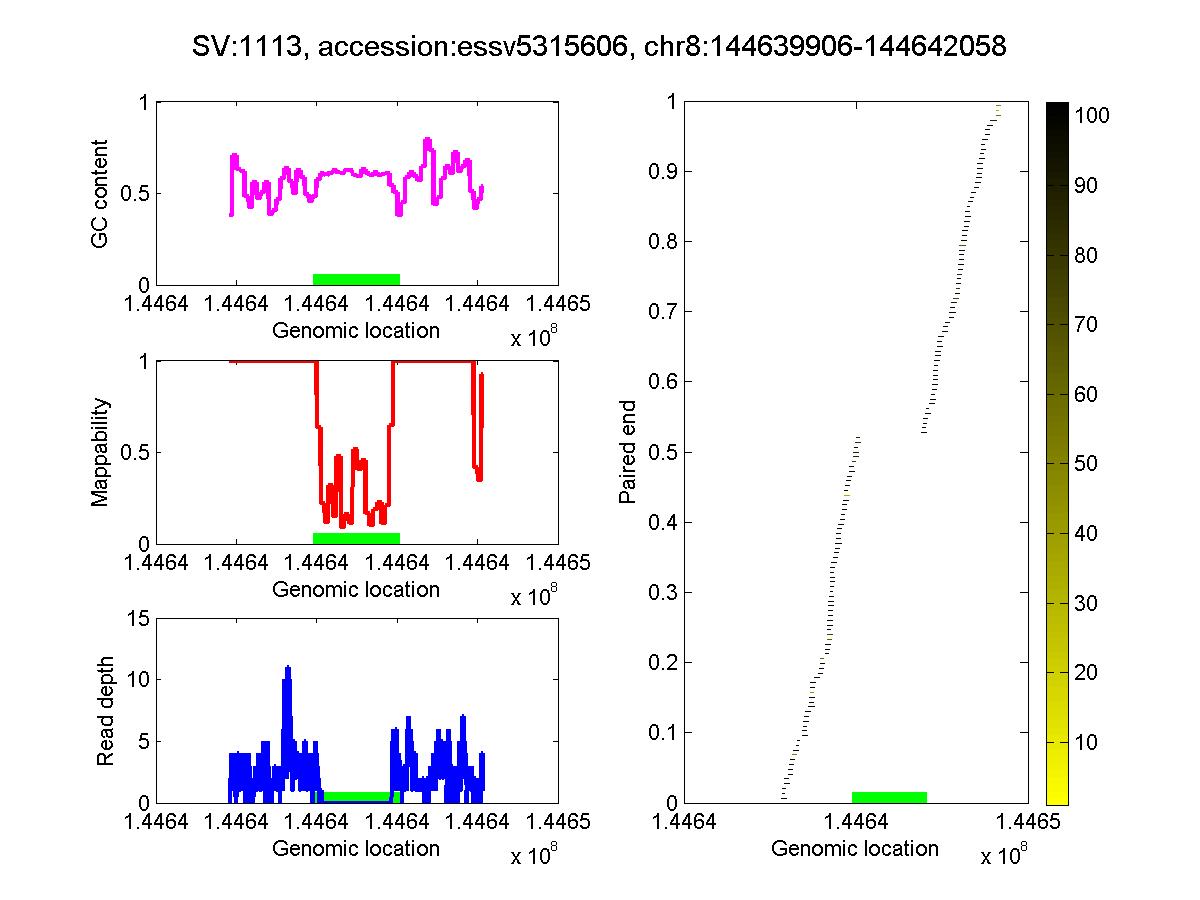

Supplement: Supplementary Materials — Supplementary data are available with this article at http://gr.xjtu.edu.cn/c/document_library/get_file?p_l_id=2403541&folderId=2539941&name=DLFE-115097.zip. Table S1 lists the complete information of suspicious variants and false positives, and the FIG directory contains the validation figures of each false positive. [file 8420547.f1.zip › 8420547.f1/FIG/SV1113.jpg]

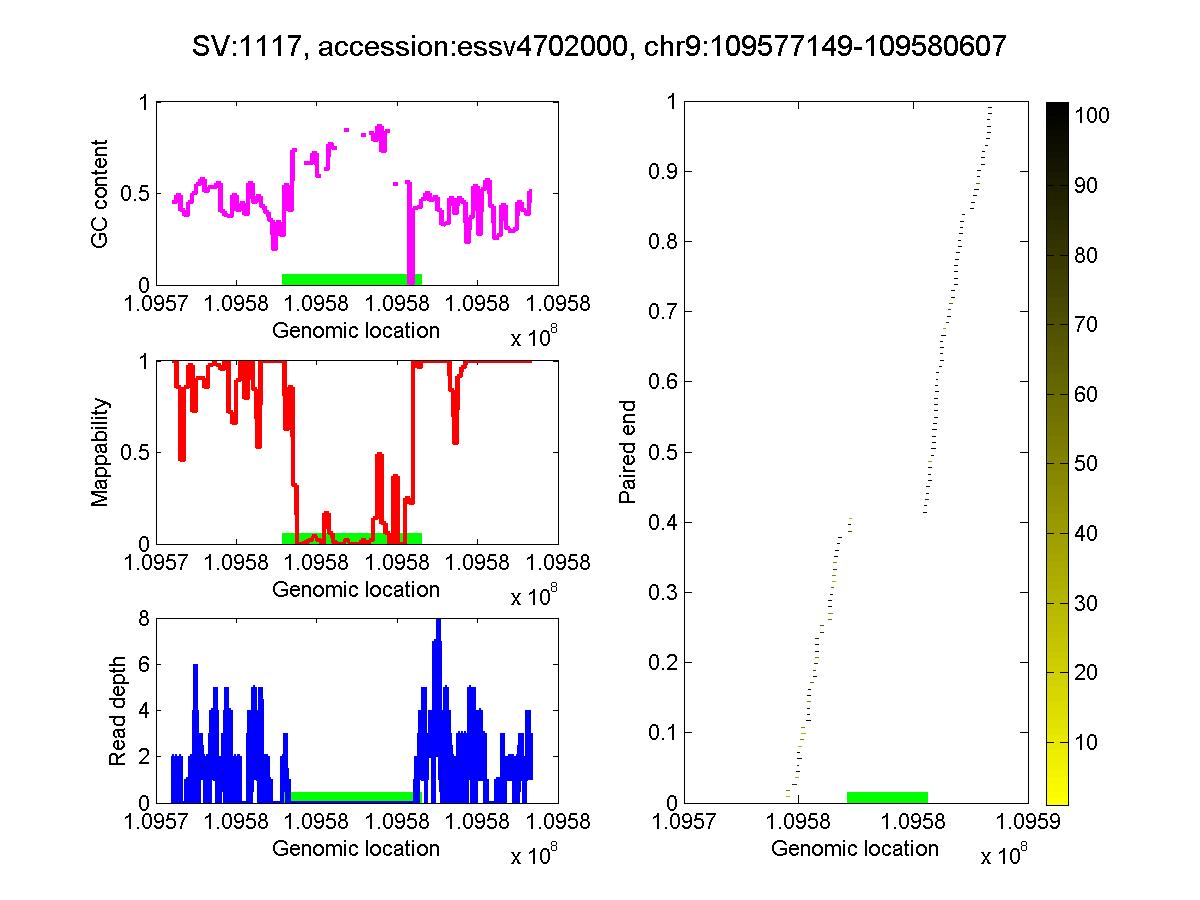

Supplement: Supplementary Materials — Supplementary data are available with this article at http://gr.xjtu.edu.cn/c/document_library/get_file?p_l_id=2403541&folderId=2539941&name=DLFE-115097.zip. Table S1 lists the complete information of suspicious variants and false positives, and the FIG directory contains the validation figures of each false positive. [file 8420547.f1.zip › 8420547.f1/FIG/SV1117.jpg]

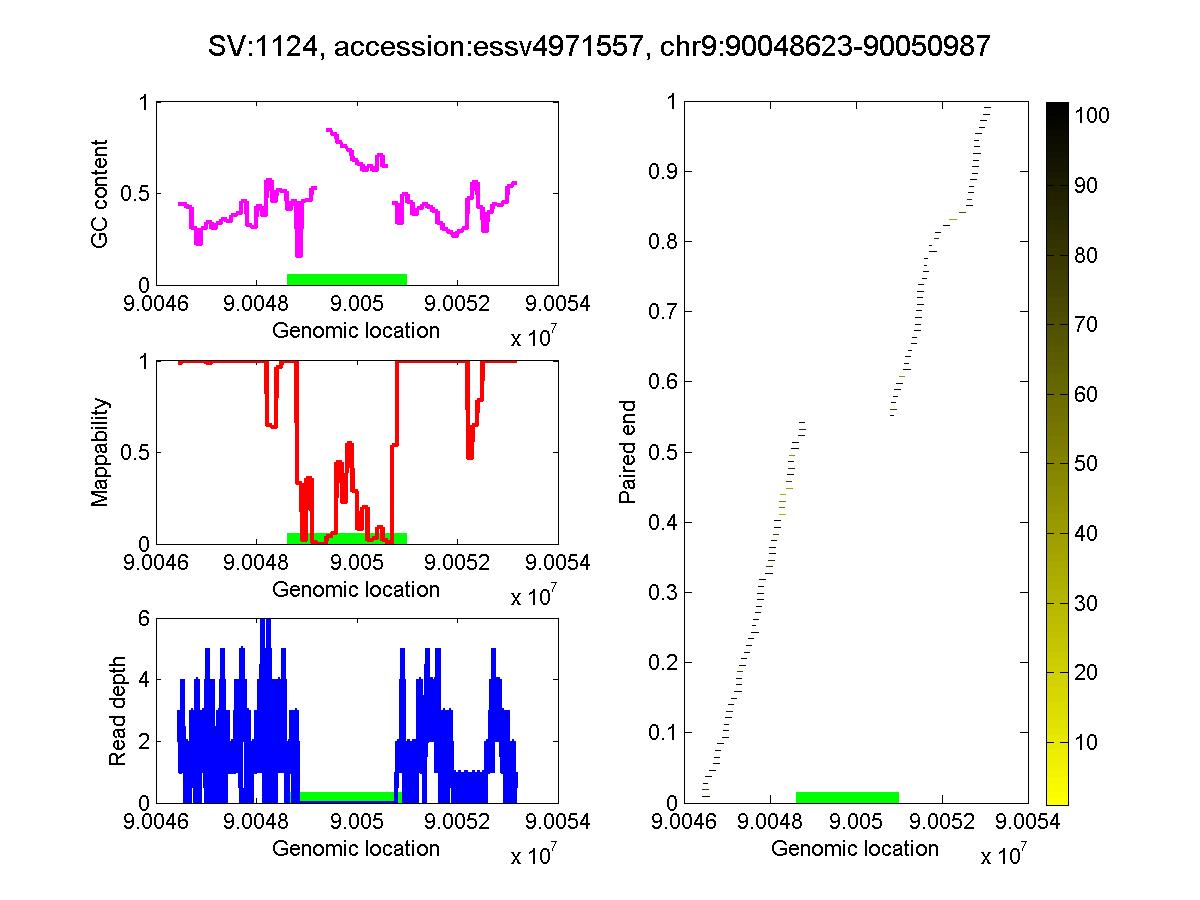

Supplement: Supplementary Materials — Supplementary data are available with this article at http://gr.xjtu.edu.cn/c/document_library/get_file?p_l_id=2403541&folderId=2539941&name=DLFE-115097.zip. Table S1 lists the complete information of suspicious variants and false positives, and the FIG directory contains the validation figures of each false positive. [file 8420547.f1.zip › 8420547.f1/FIG/SV1124.jpg]

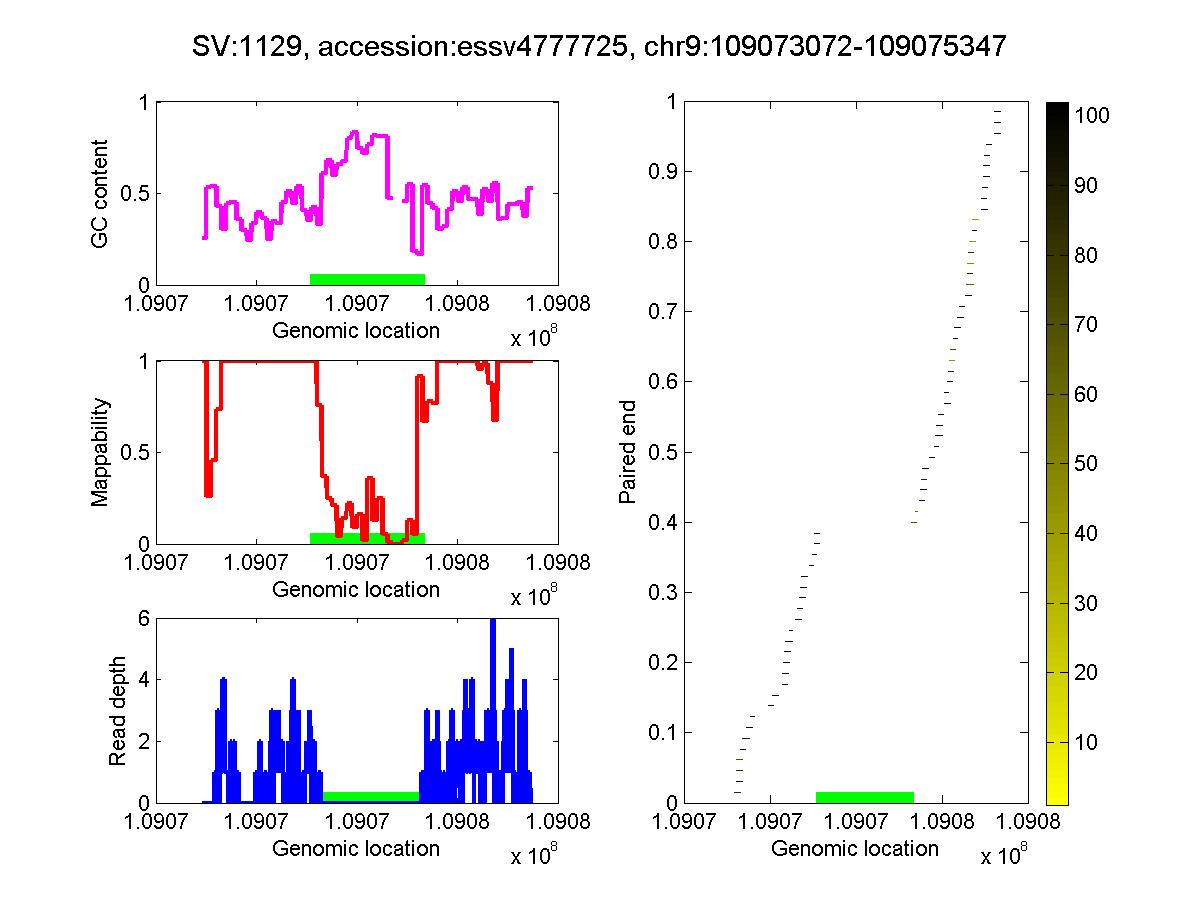

Supplement: Supplementary Materials — Supplementary data are available with this article at http://gr.xjtu.edu.cn/c/document_library/get_file?p_l_id=2403541&folderId=2539941&name=DLFE-115097.zip. Table S1 lists the complete information of suspicious variants and false positives, and the FIG directory contains the validation figures of each false positive. [file 8420547.f1.zip › 8420547.f1/FIG/SV1129.jpg]

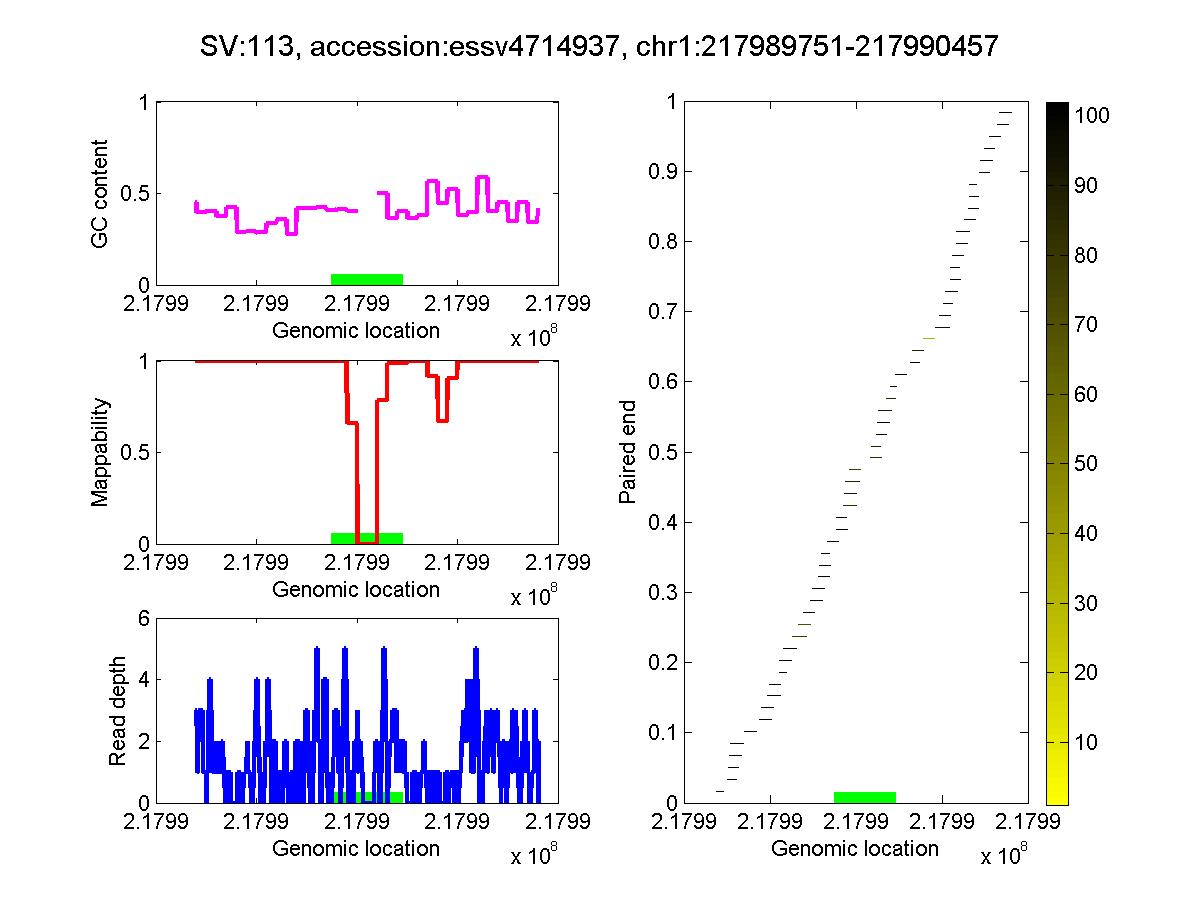

Supplement: Supplementary Materials — Supplementary data are available with this article at http://gr.xjtu.edu.cn/c/document_library/get_file?p_l_id=2403541&folderId=2539941&name=DLFE-115097.zip. Table S1 lists the complete information of suspicious variants and false positives, and the FIG directory contains the validation figures of each false positive. [file 8420547.f1.zip › 8420547.f1/FIG/SV113.jpg]

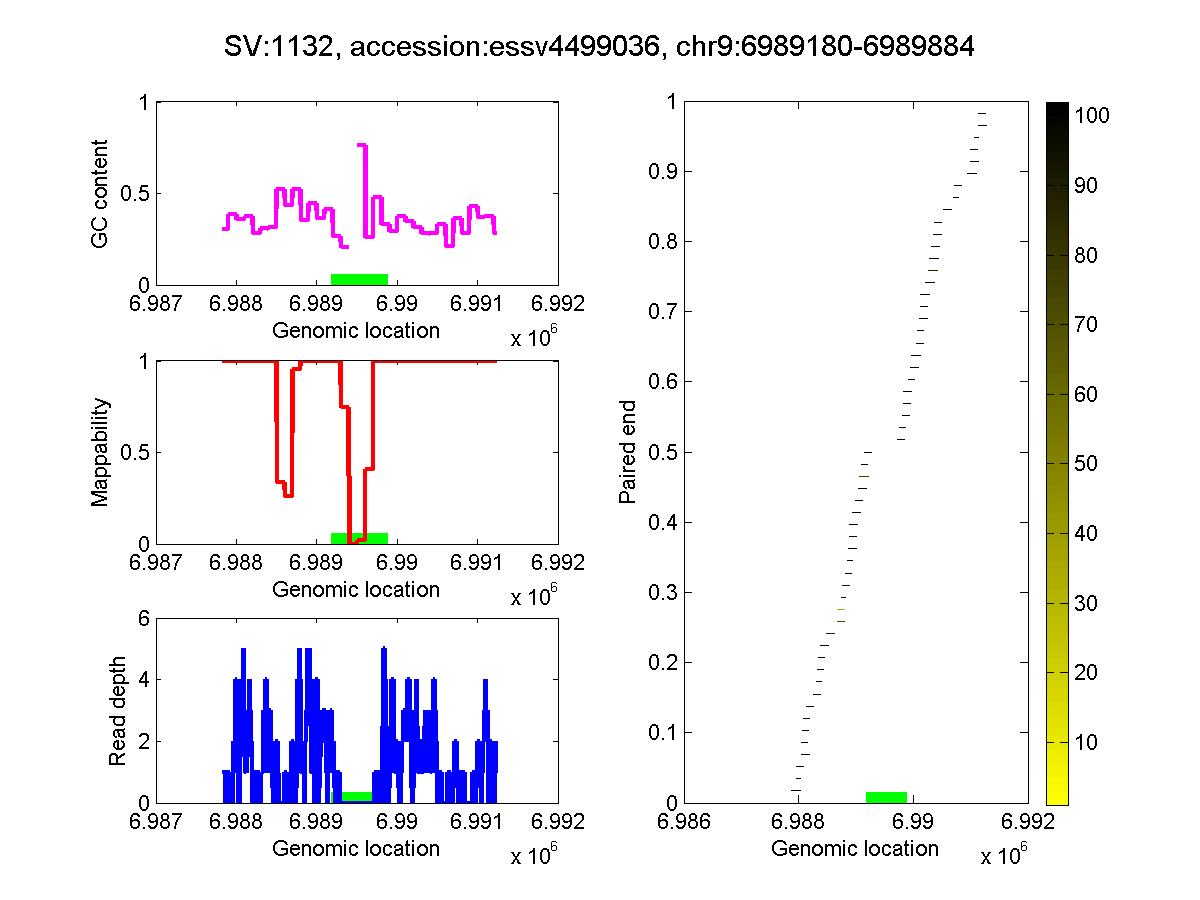

Supplement: Supplementary Materials — Supplementary data are available with this article at http://gr.xjtu.edu.cn/c/document_library/get_file?p_l_id=2403541&folderId=2539941&name=DLFE-115097.zip. Table S1 lists the complete information of suspicious variants and false positives, and the FIG directory contains the validation figures of each false positive. [file 8420547.f1.zip › 8420547.f1/FIG/SV1132.jpg]

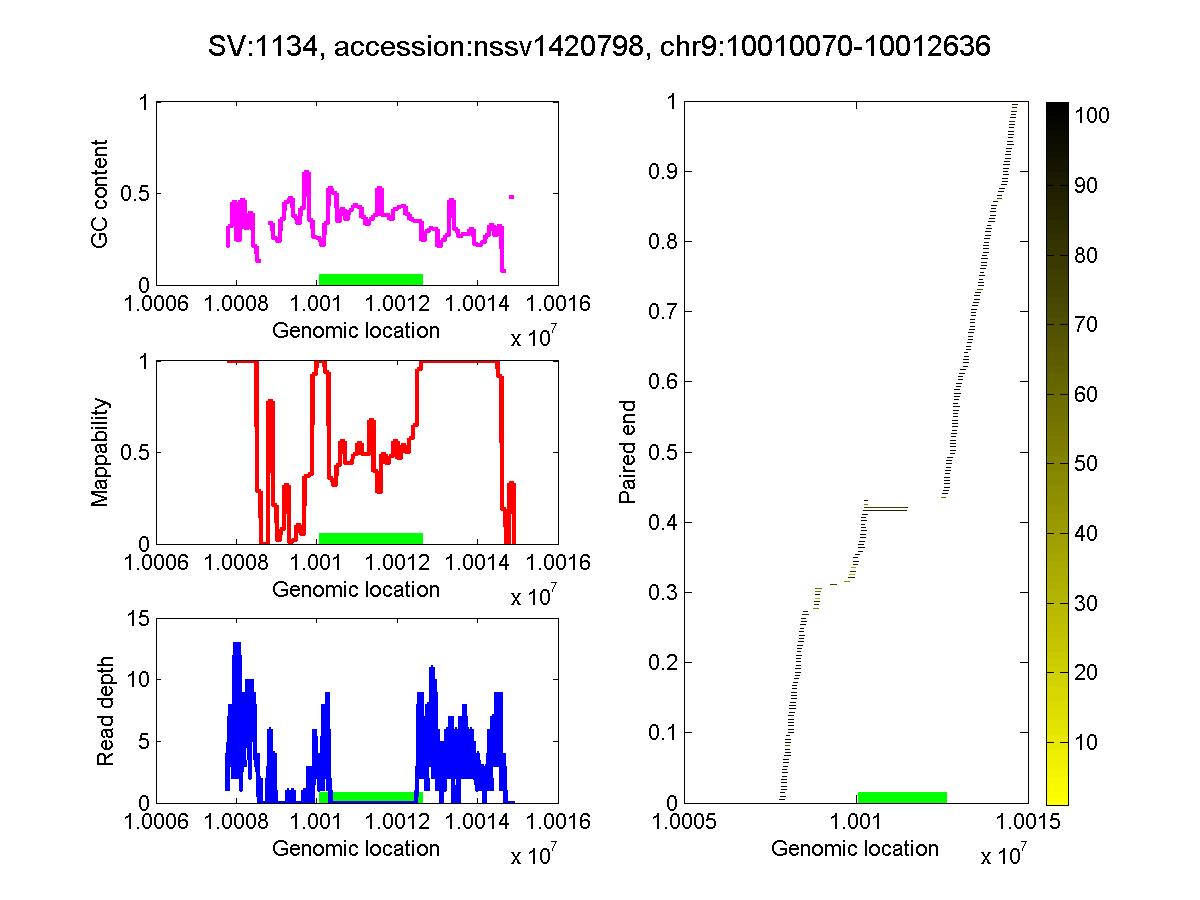

Supplement: Supplementary Materials — Supplementary data are available with this article at http://gr.xjtu.edu.cn/c/document_library/get_file?p_l_id=2403541&folderId=2539941&name=DLFE-115097.zip. Table S1 lists the complete information of suspicious variants and false positives, and the FIG directory contains the validation figures of each false positive. [file 8420547.f1.zip › 8420547.f1/FIG/SV1134.jpg]

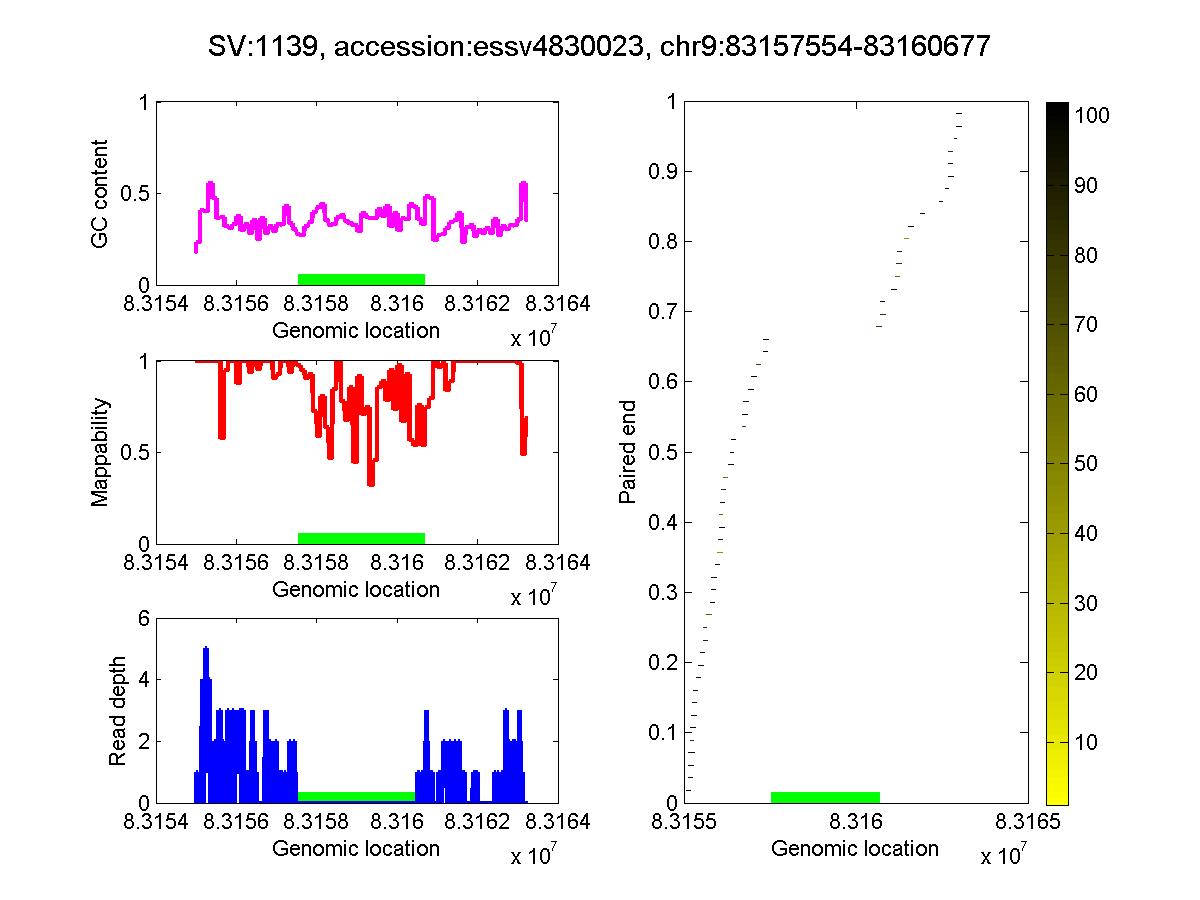

Supplement: Supplementary Materials — Supplementary data are available with this article at http://gr.xjtu.edu.cn/c/document_library/get_file?p_l_id=2403541&folderId=2539941&name=DLFE-115097.zip. Table S1 lists the complete information of suspicious variants and false positives, and the FIG directory contains the validation figures of each false positive. [file 8420547.f1.zip › 8420547.f1/FIG/SV1139.jpg]

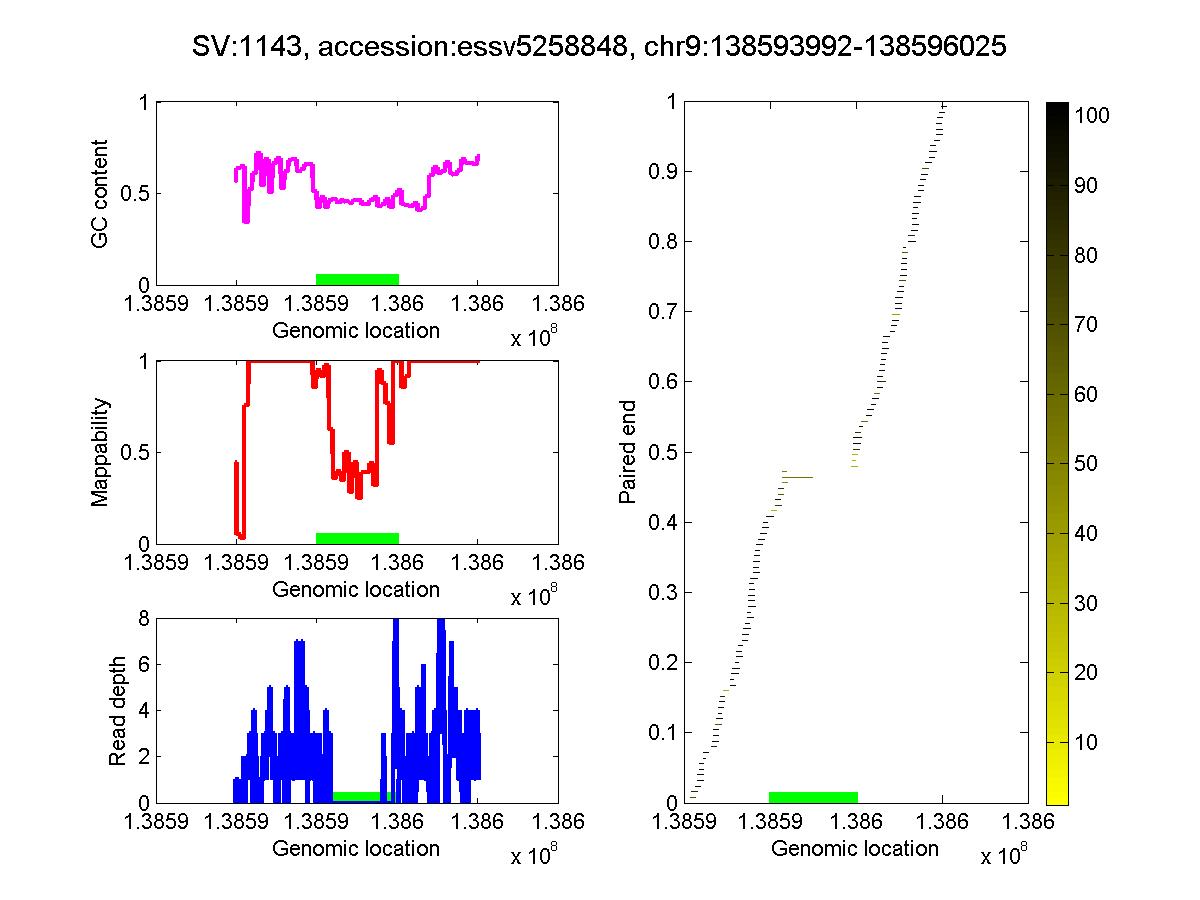

Supplement: Supplementary Materials — Supplementary data are available with this article at http://gr.xjtu.edu.cn/c/document_library/get_file?p_l_id=2403541&folderId=2539941&name=DLFE-115097.zip. Table S1 lists the complete information of suspicious variants and false positives, and the FIG directory contains the validation figures of each false positive. [file 8420547.f1.zip › 8420547.f1/FIG/SV1143.jpg]

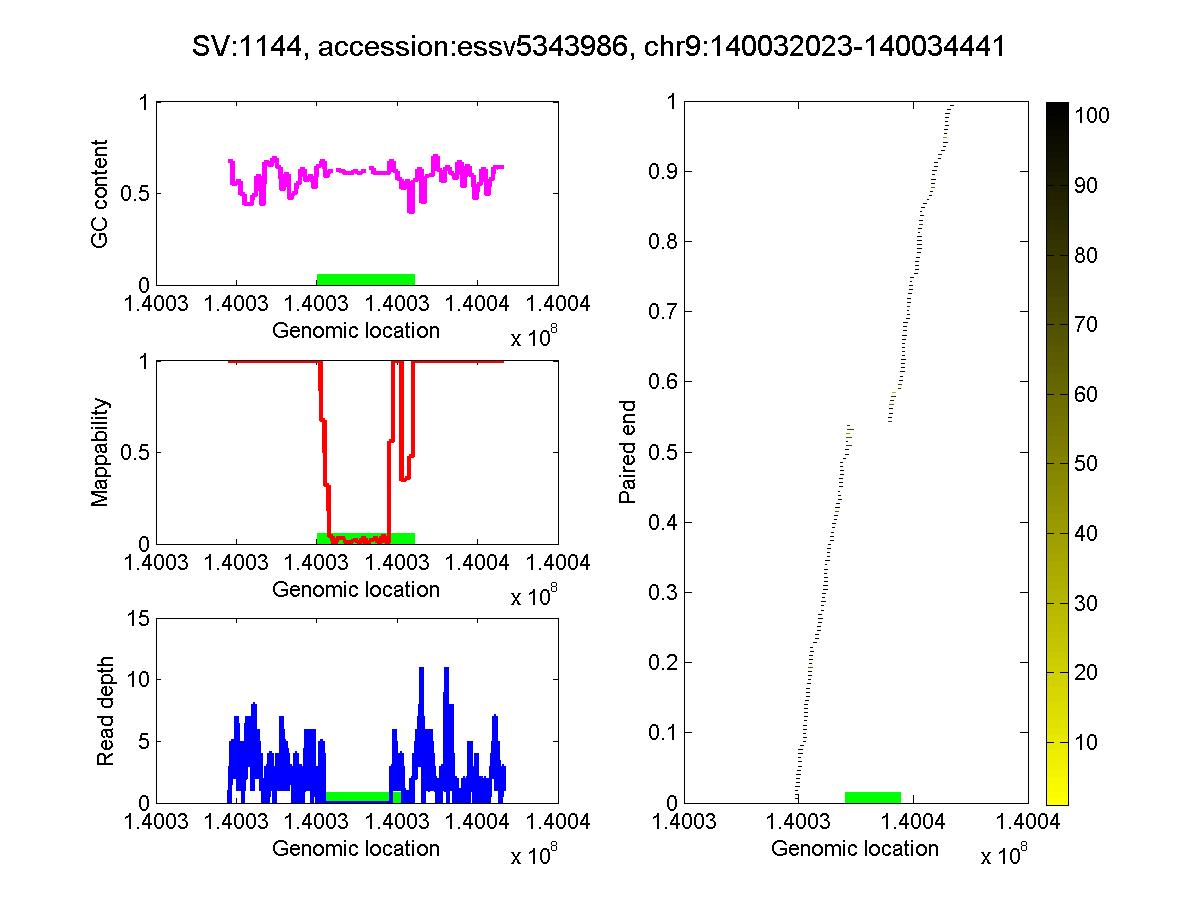

Supplement: Supplementary Materials — Supplementary data are available with this article at http://gr.xjtu.edu.cn/c/document_library/get_file?p_l_id=2403541&folderId=2539941&name=DLFE-115097.zip. Table S1 lists the complete information of suspicious variants and false positives, and the FIG directory contains the validation figures of each false positive. [file 8420547.f1.zip › 8420547.f1/FIG/SV1144.jpg]

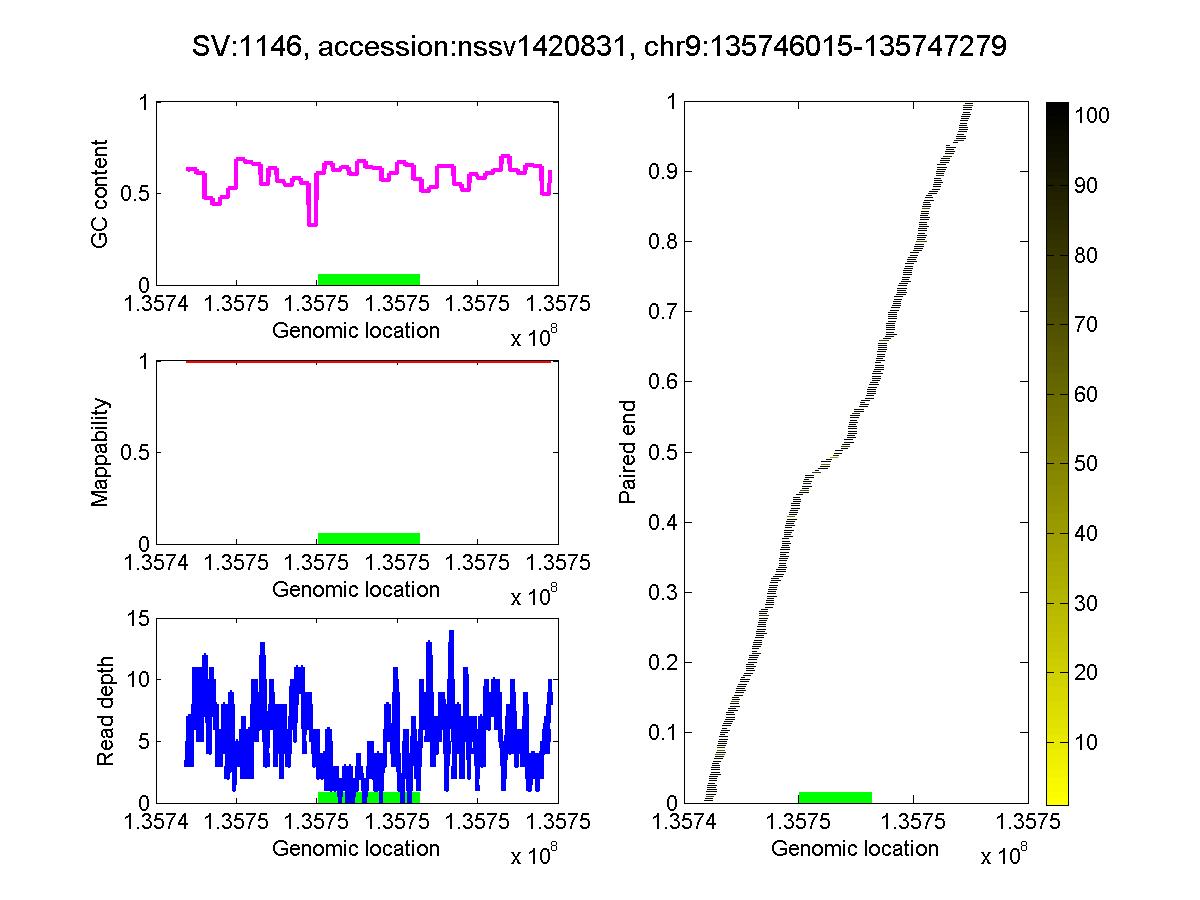

Supplement: Supplementary Materials — Supplementary data are available with this article at http://gr.xjtu.edu.cn/c/document_library/get_file?p_l_id=2403541&folderId=2539941&name=DLFE-115097.zip. Table S1 lists the complete information of suspicious variants and false positives, and the FIG directory contains the validation figures of each false positive. [file 8420547.f1.zip › 8420547.f1/FIG/SV1146.jpg]

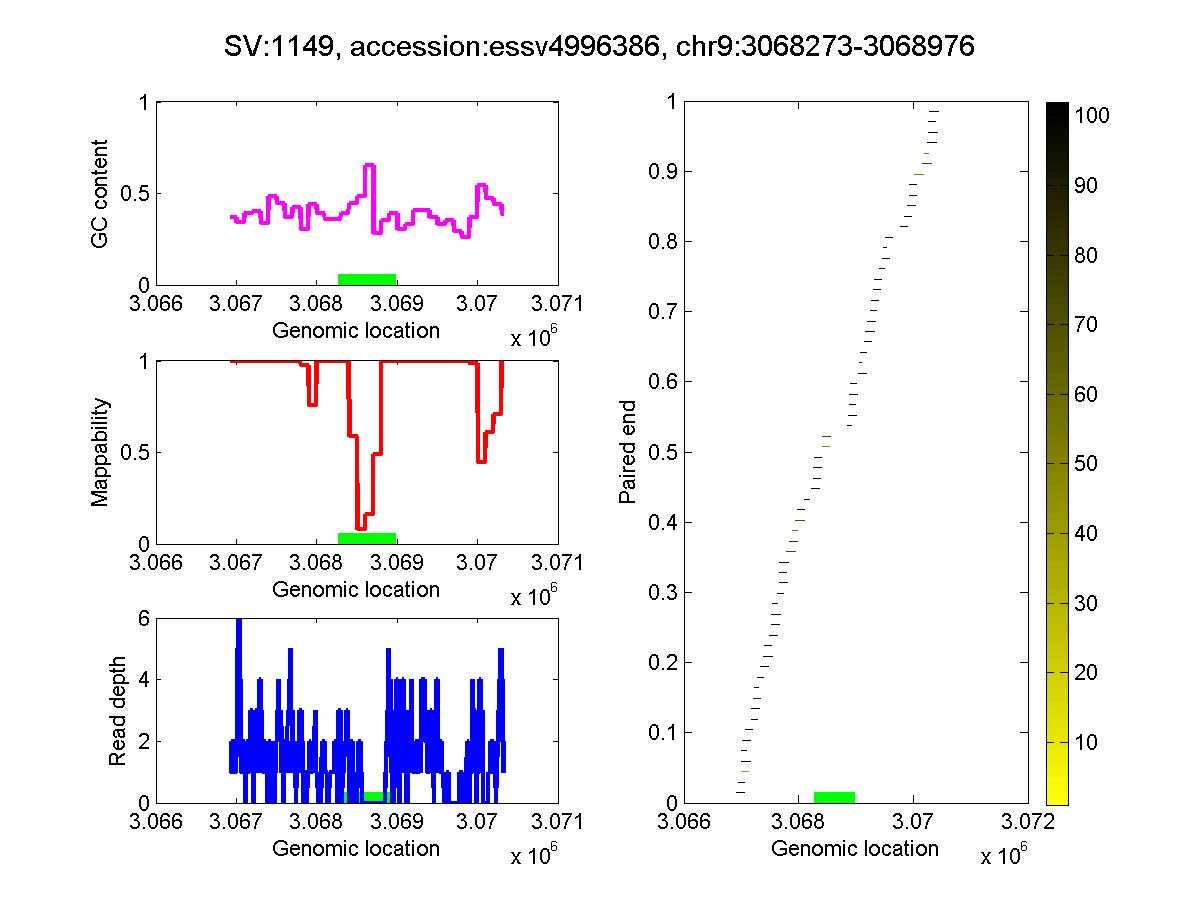

Supplement: Supplementary Materials — Supplementary data are available with this article at http://gr.xjtu.edu.cn/c/document_library/get_file?p_l_id=2403541&folderId=2539941&name=DLFE-115097.zip. Table S1 lists the complete information of suspicious variants and false positives, and the FIG directory contains the validation figures of each false positive. [file 8420547.f1.zip › 8420547.f1/FIG/SV1149.jpg]

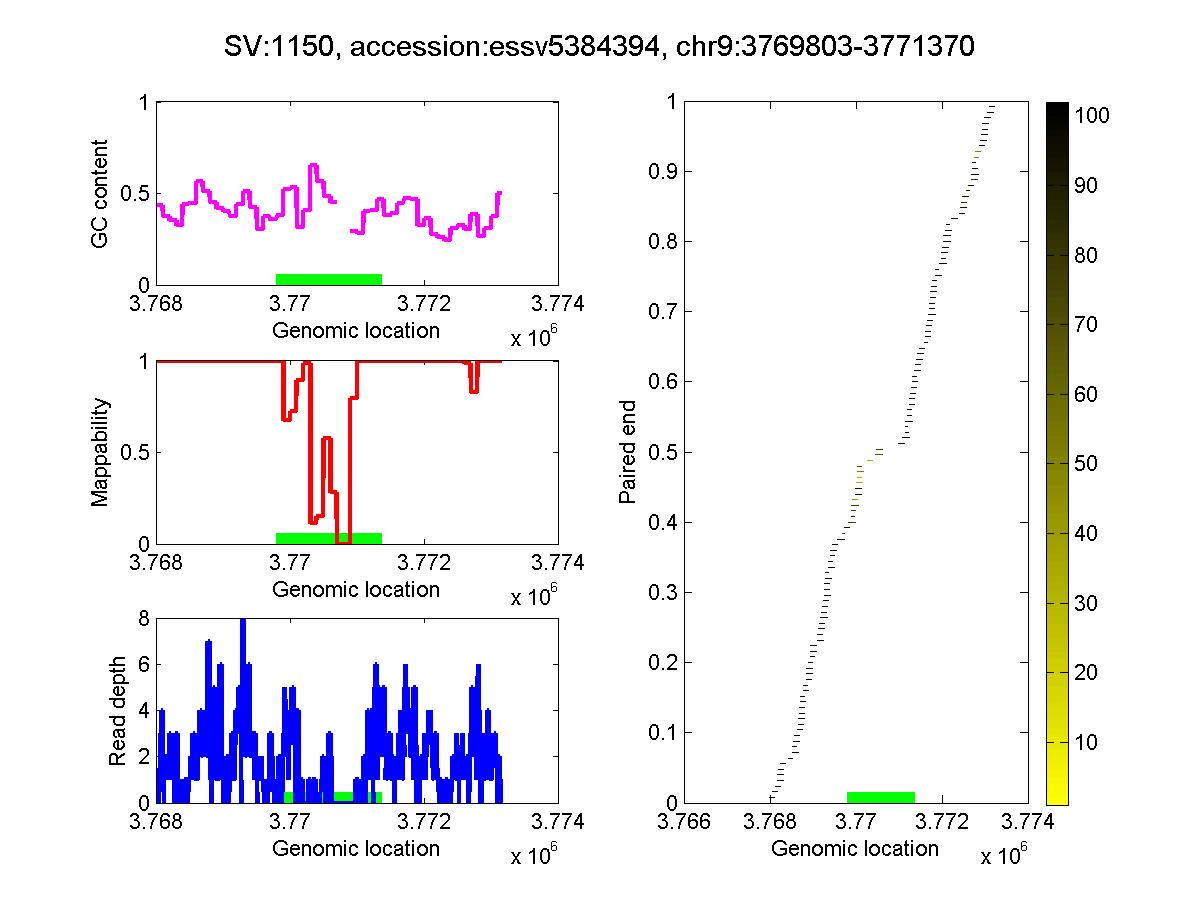

Supplement: Supplementary Materials — Supplementary data are available with this article at http://gr.xjtu.edu.cn/c/document_library/get_file?p_l_id=2403541&folderId=2539941&name=DLFE-115097.zip. Table S1 lists the complete information of suspicious variants and false positives, and the FIG directory contains the validation figures of each false positive. [file 8420547.f1.zip › 8420547.f1/FIG/SV1150.jpg]

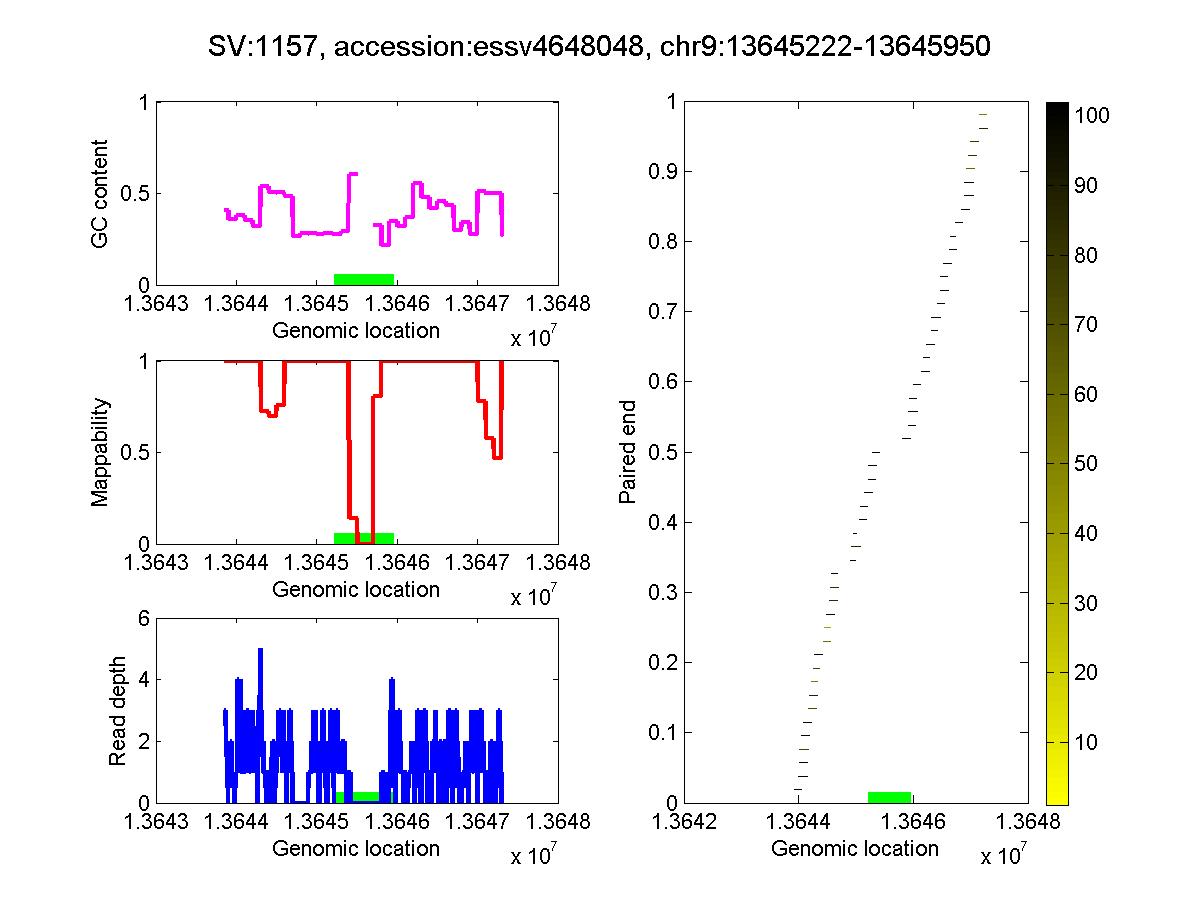

Supplement: Supplementary Materials — Supplementary data are available with this article at http://gr.xjtu.edu.cn/c/document_library/get_file?p_l_id=2403541&folderId=2539941&name=DLFE-115097.zip. Table S1 lists the complete information of suspicious variants and false positives, and the FIG directory contains the validation figures of each false positive. [file 8420547.f1.zip › 8420547.f1/FIG/SV1157.jpg]

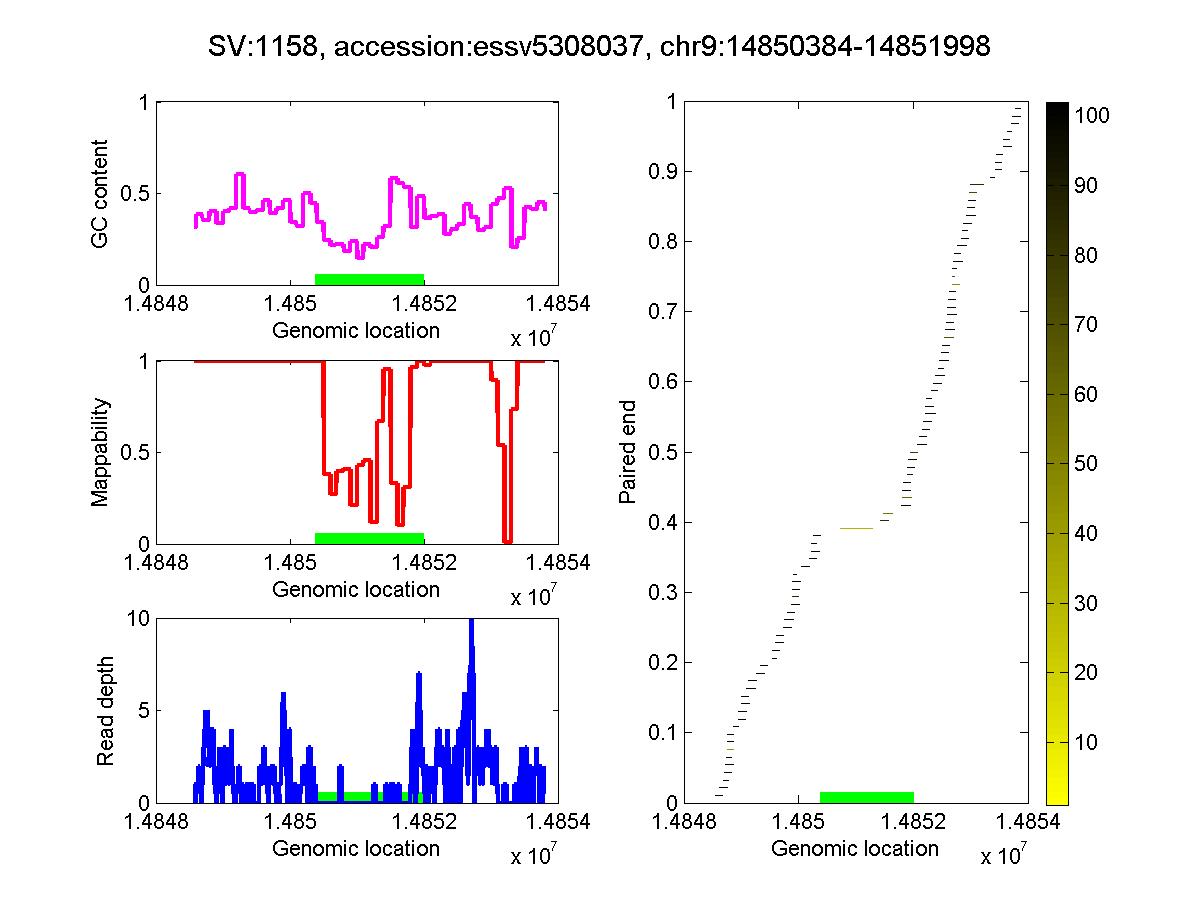

Supplement: Supplementary Materials — Supplementary data are available with this article at http://gr.xjtu.edu.cn/c/document_library/get_file?p_l_id=2403541&folderId=2539941&name=DLFE-115097.zip. Table S1 lists the complete information of suspicious variants and false positives, and the FIG directory contains the validation figures of each false positive. [file 8420547.f1.zip › 8420547.f1/FIG/SV1158.jpg]

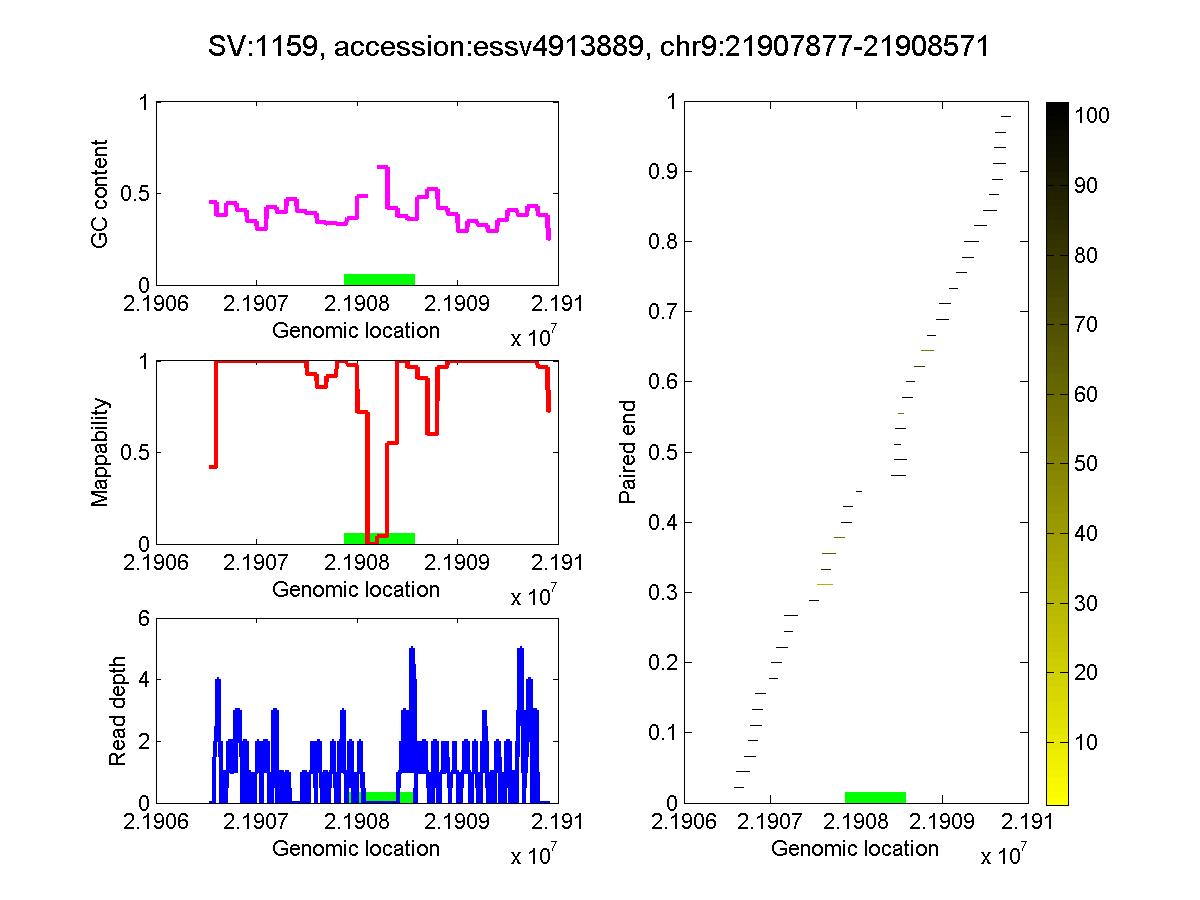

Supplement: Supplementary Materials — Supplementary data are available with this article at http://gr.xjtu.edu.cn/c/document_library/get_file?p_l_id=2403541&folderId=2539941&name=DLFE-115097.zip. Table S1 lists the complete information of suspicious variants and false positives, and the FIG directory contains the validation figures of each false positive. [file 8420547.f1.zip › 8420547.f1/FIG/SV1159.jpg]

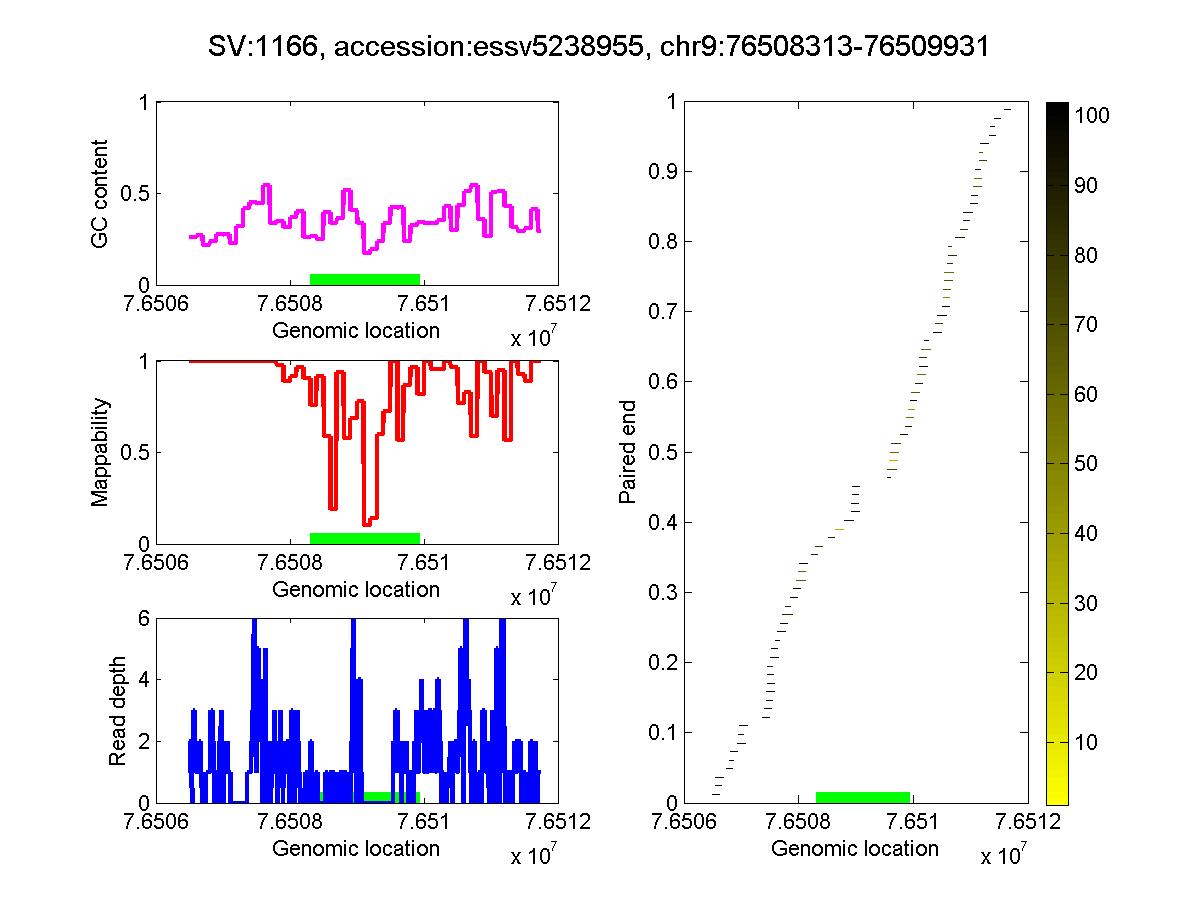

Supplement: Supplementary Materials — Supplementary data are available with this article at http://gr.xjtu.edu.cn/c/document_library/get_file?p_l_id=2403541&folderId=2539941&name=DLFE-115097.zip. Table S1 lists the complete information of suspicious variants and false positives, and the FIG directory contains the validation figures of each false positive. [file 8420547.f1.zip › 8420547.f1/FIG/SV1166.jpg]

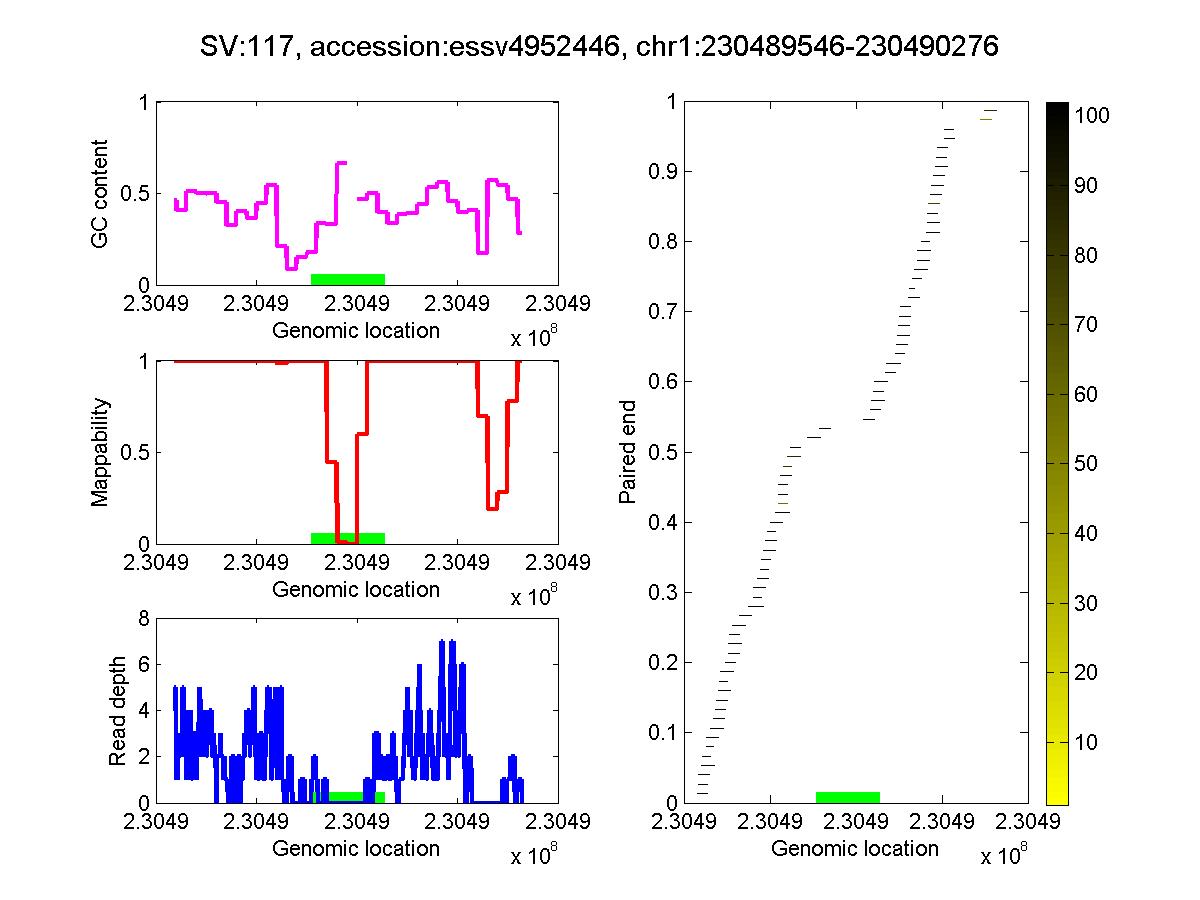

Supplement: Supplementary Materials — Supplementary data are available with this article at http://gr.xjtu.edu.cn/c/document_library/get_file?p_l_id=2403541&folderId=2539941&name=DLFE-115097.zip. Table S1 lists the complete information of suspicious variants and false positives, and the FIG directory contains the validation figures of each false positive. [file 8420547.f1.zip › 8420547.f1/FIG/SV117.jpg]

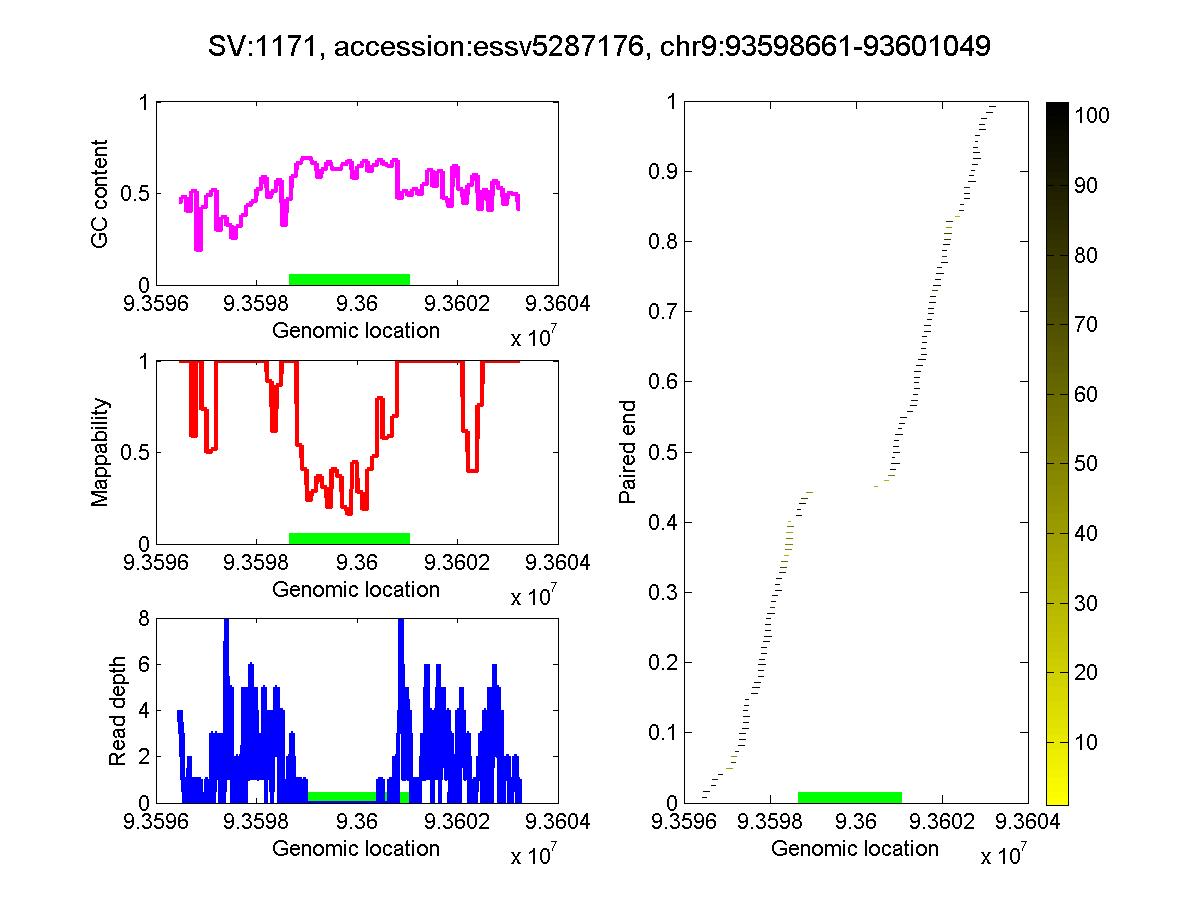

Supplement: Supplementary Materials — Supplementary data are available with this article at http://gr.xjtu.edu.cn/c/document_library/get_file?p_l_id=2403541&folderId=2539941&name=DLFE-115097.zip. Table S1 lists the complete information of suspicious variants and false positives, and the FIG directory contains the validation figures of each false positive. [file 8420547.f1.zip › 8420547.f1/FIG/SV1171.jpg]

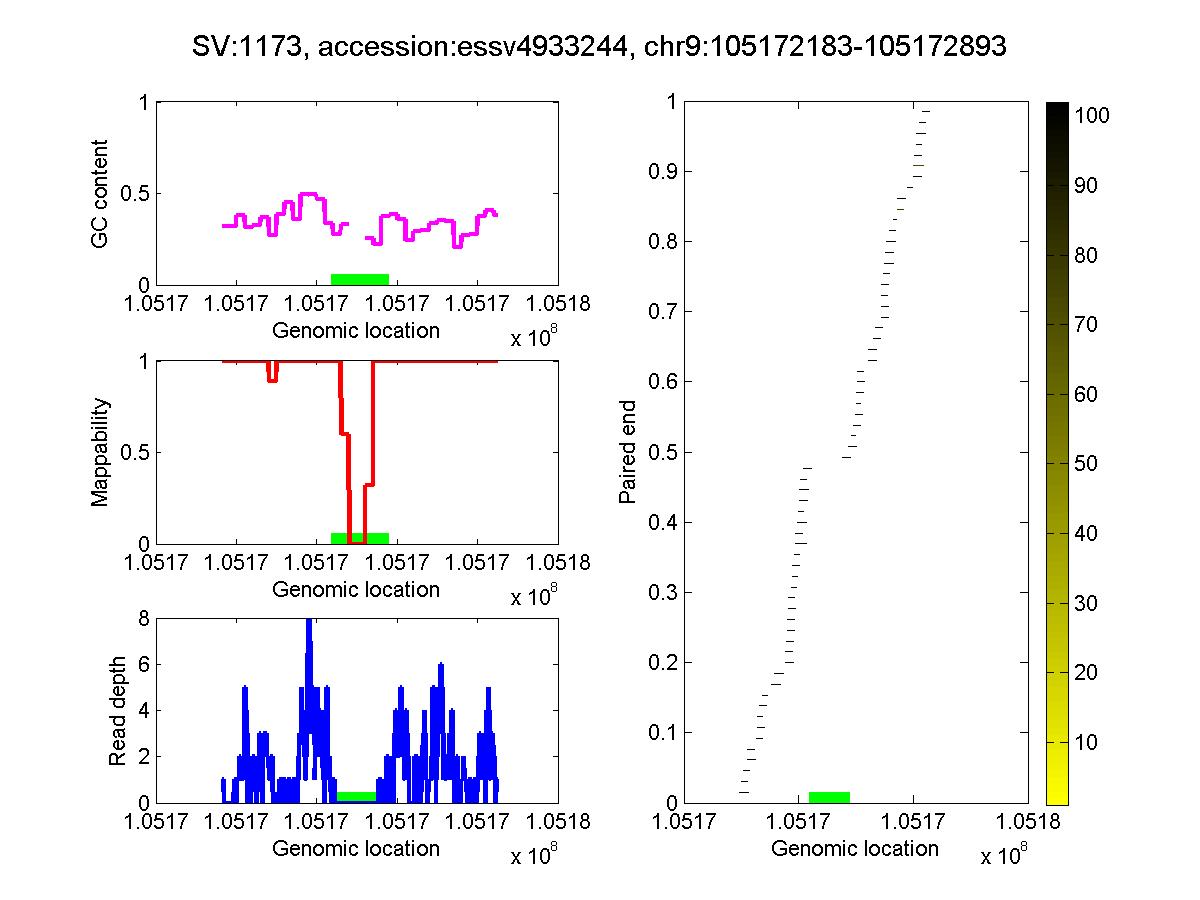

Supplement: Supplementary Materials — Supplementary data are available with this article at http://gr.xjtu.edu.cn/c/document_library/get_file?p_l_id=2403541&folderId=2539941&name=DLFE-115097.zip. Table S1 lists the complete information of suspicious variants and false positives, and the FIG directory contains the validation figures of each false positive. [file 8420547.f1.zip › 8420547.f1/FIG/SV1173.jpg]

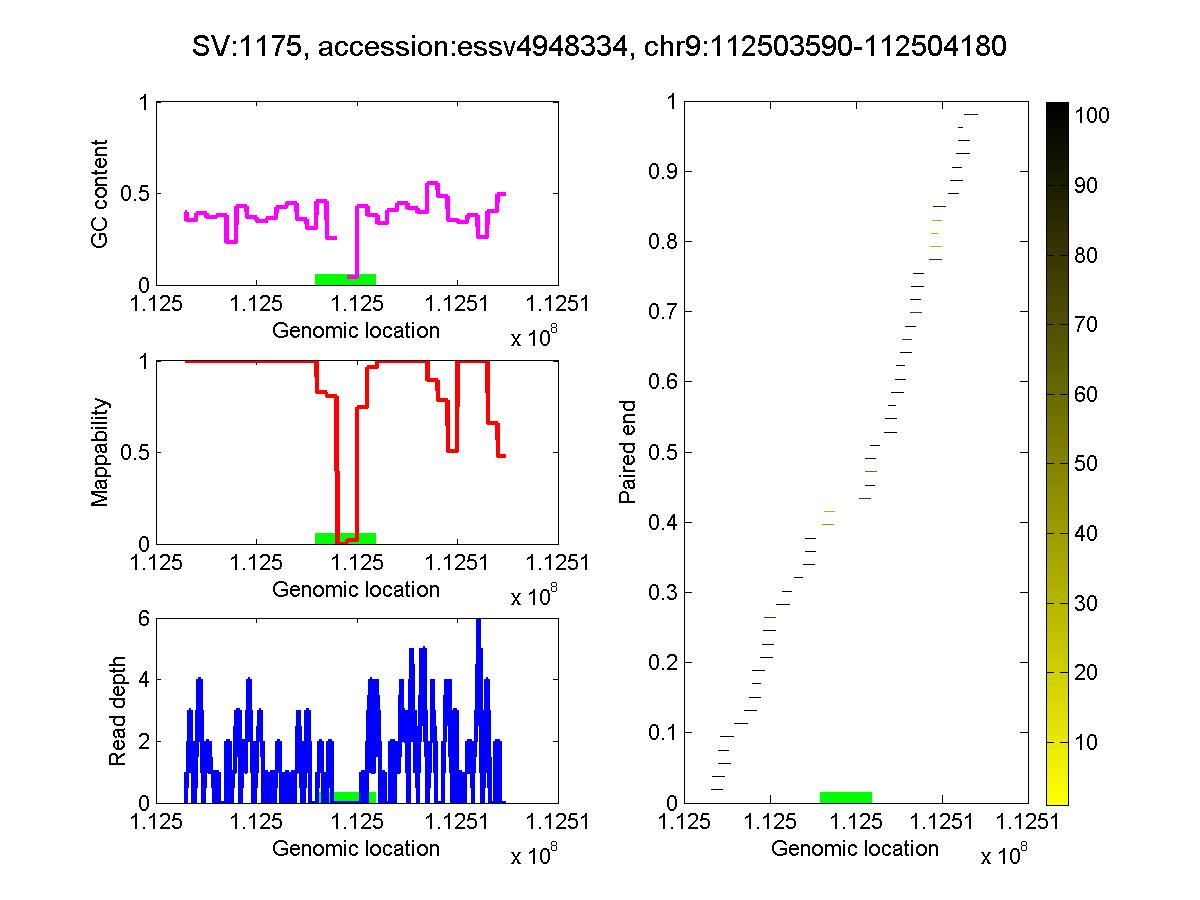

Supplement: Supplementary Materials — Supplementary data are available with this article at http://gr.xjtu.edu.cn/c/document_library/get_file?p_l_id=2403541&folderId=2539941&name=DLFE-115097.zip. Table S1 lists the complete information of suspicious variants and false positives, and the FIG directory contains the validation figures of each false positive. [file 8420547.f1.zip › 8420547.f1/FIG/SV1175.jpg]

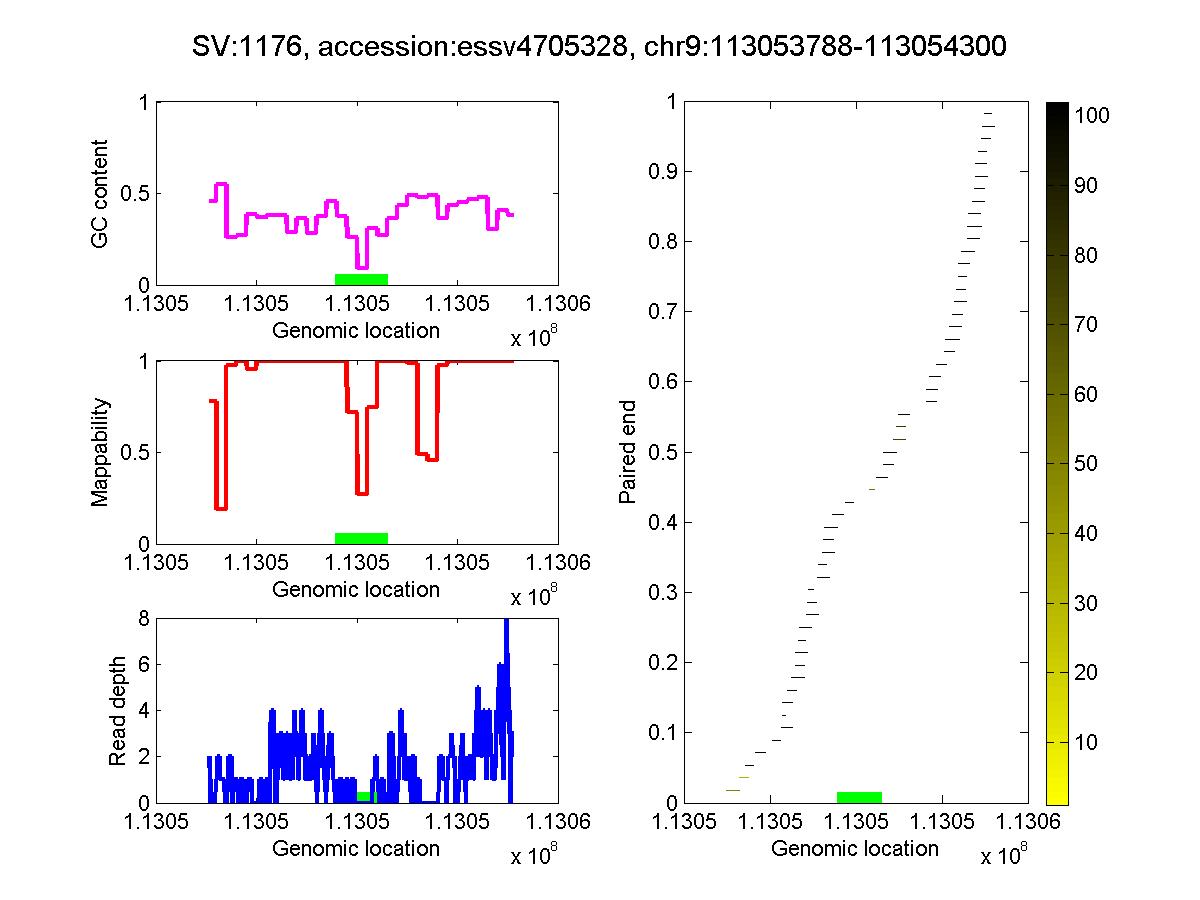

Supplement: Supplementary Materials — Supplementary data are available with this article at http://gr.xjtu.edu.cn/c/document_library/get_file?p_l_id=2403541&folderId=2539941&name=DLFE-115097.zip. Table S1 lists the complete information of suspicious variants and false positives, and the FIG directory contains the validation figures of each false positive. [file 8420547.f1.zip › 8420547.f1/FIG/SV1176.jpg]

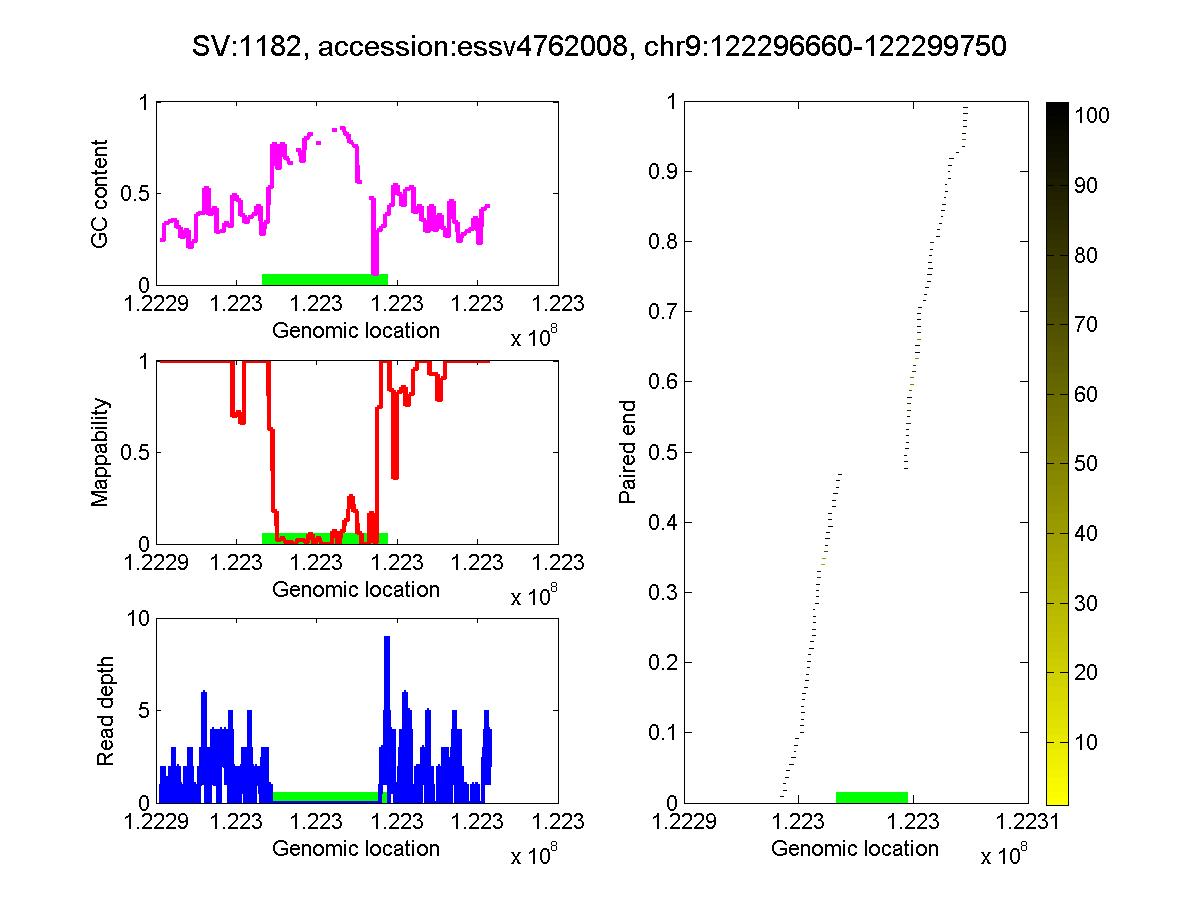

Supplement: Supplementary Materials — Supplementary data are available with this article at http://gr.xjtu.edu.cn/c/document_library/get_file?p_l_id=2403541&folderId=2539941&name=DLFE-115097.zip. Table S1 lists the complete information of suspicious variants and false positives, and the FIG directory contains the validation figures of each false positive. [file 8420547.f1.zip › 8420547.f1/FIG/SV1182.jpg]

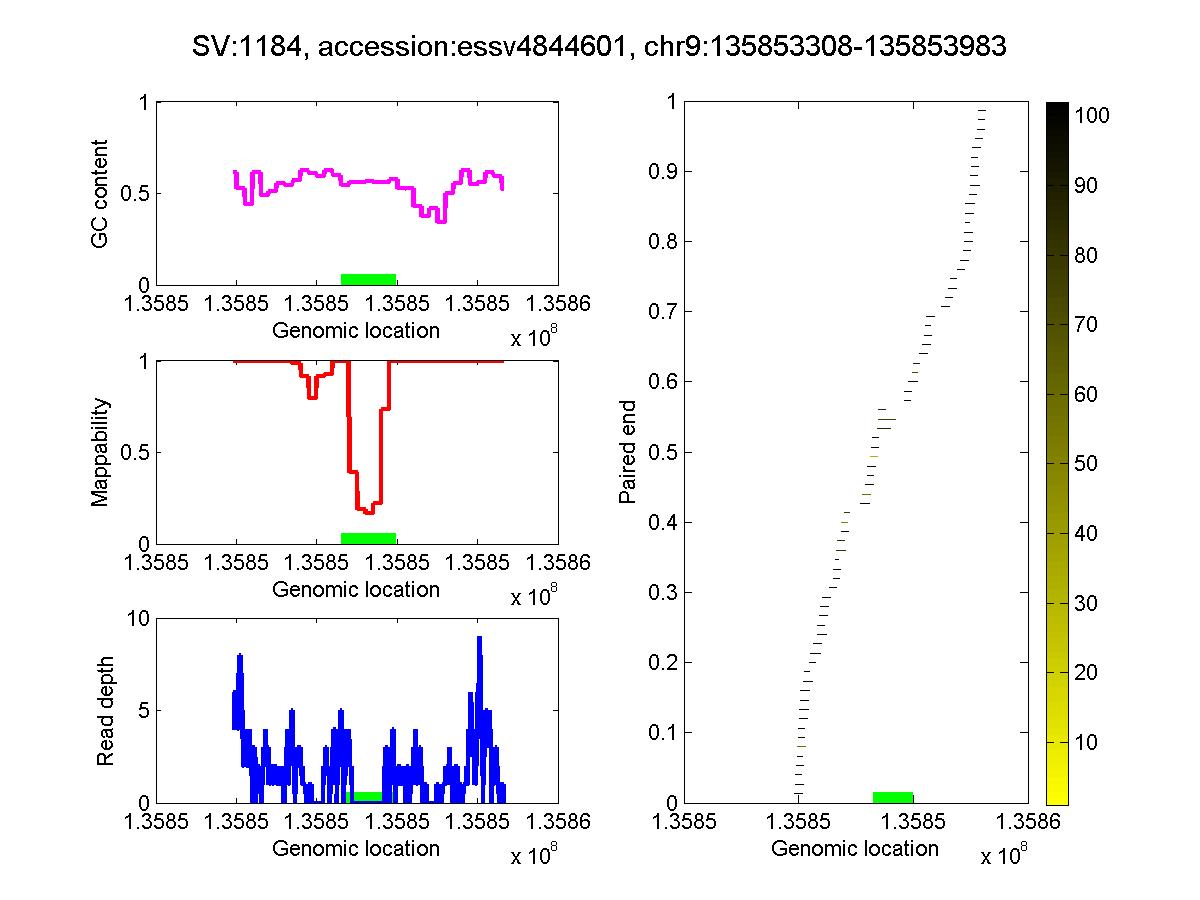

Supplement: Supplementary Materials — Supplementary data are available with this article at http://gr.xjtu.edu.cn/c/document_library/get_file?p_l_id=2403541&folderId=2539941&name=DLFE-115097.zip. Table S1 lists the complete information of suspicious variants and false positives, and the FIG directory contains the validation figures of each false positive. [file 8420547.f1.zip › 8420547.f1/FIG/SV1184.jpg]

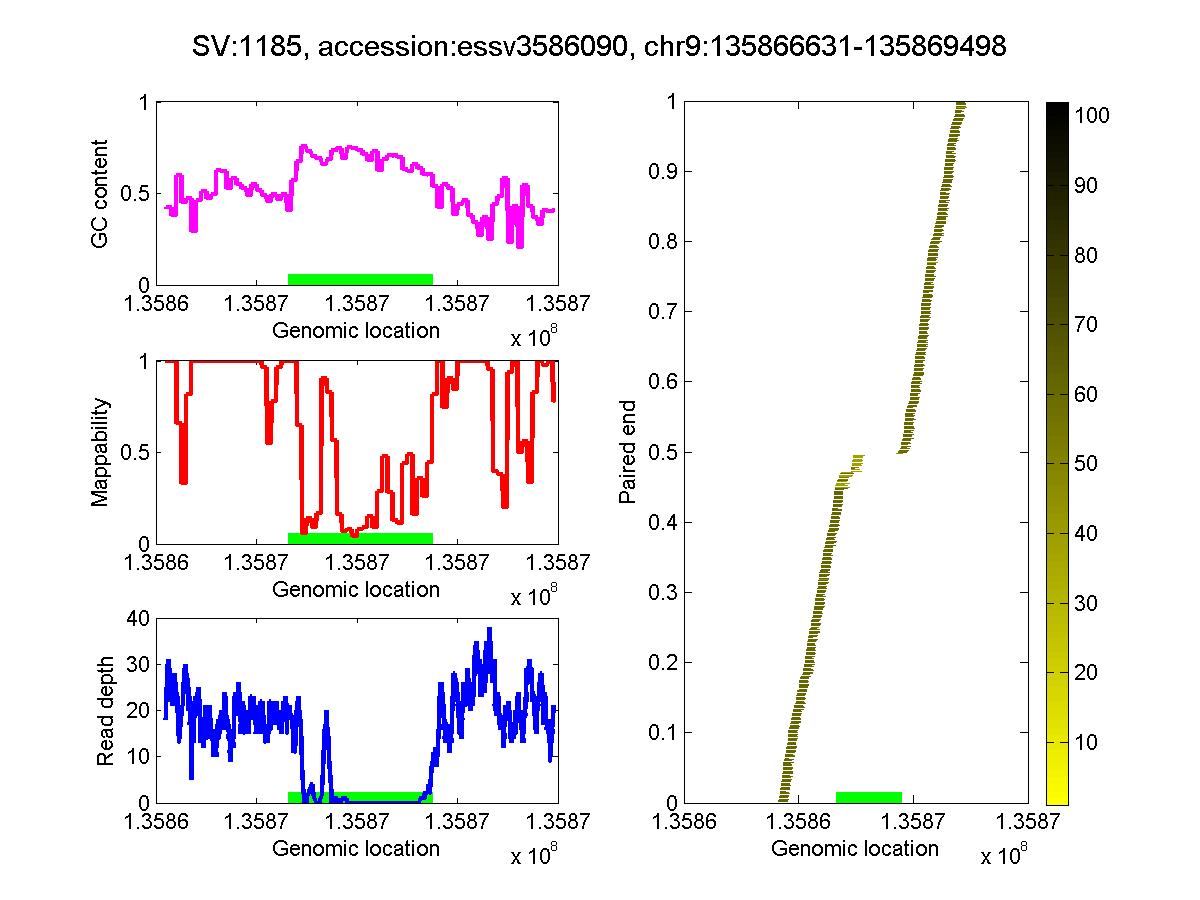

Supplement: Supplementary Materials — Supplementary data are available with this article at http://gr.xjtu.edu.cn/c/document_library/get_file?p_l_id=2403541&folderId=2539941&name=DLFE-115097.zip. Table S1 lists the complete information of suspicious variants and false positives, and the FIG directory contains the validation figures of each false positive. [file 8420547.f1.zip › 8420547.f1/FIG/SV1185.jpg]

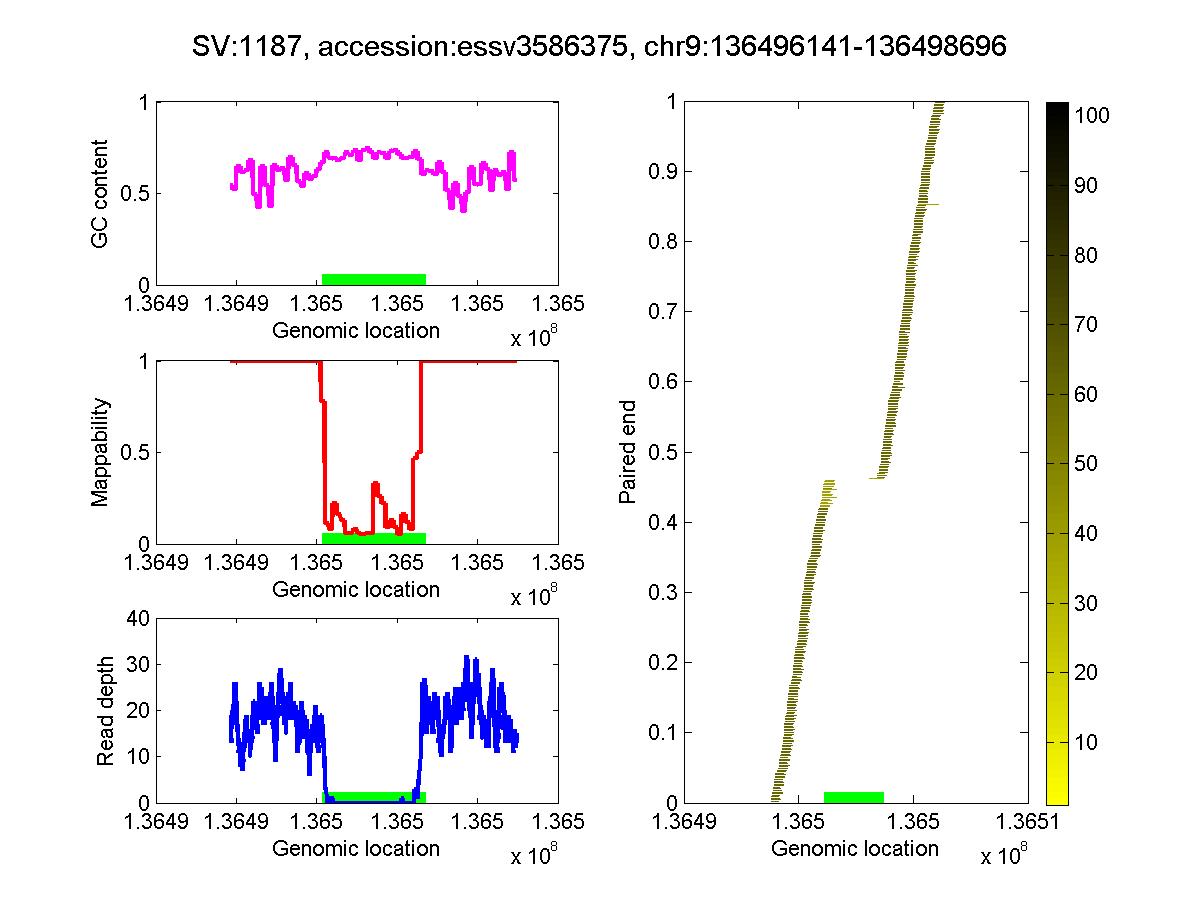

Supplement: Supplementary Materials — Supplementary data are available with this article at http://gr.xjtu.edu.cn/c/document_library/get_file?p_l_id=2403541&folderId=2539941&name=DLFE-115097.zip. Table S1 lists the complete information of suspicious variants and false positives, and the FIG directory contains the validation figures of each false positive. [file 8420547.f1.zip › 8420547.f1/FIG/SV1187.jpg]

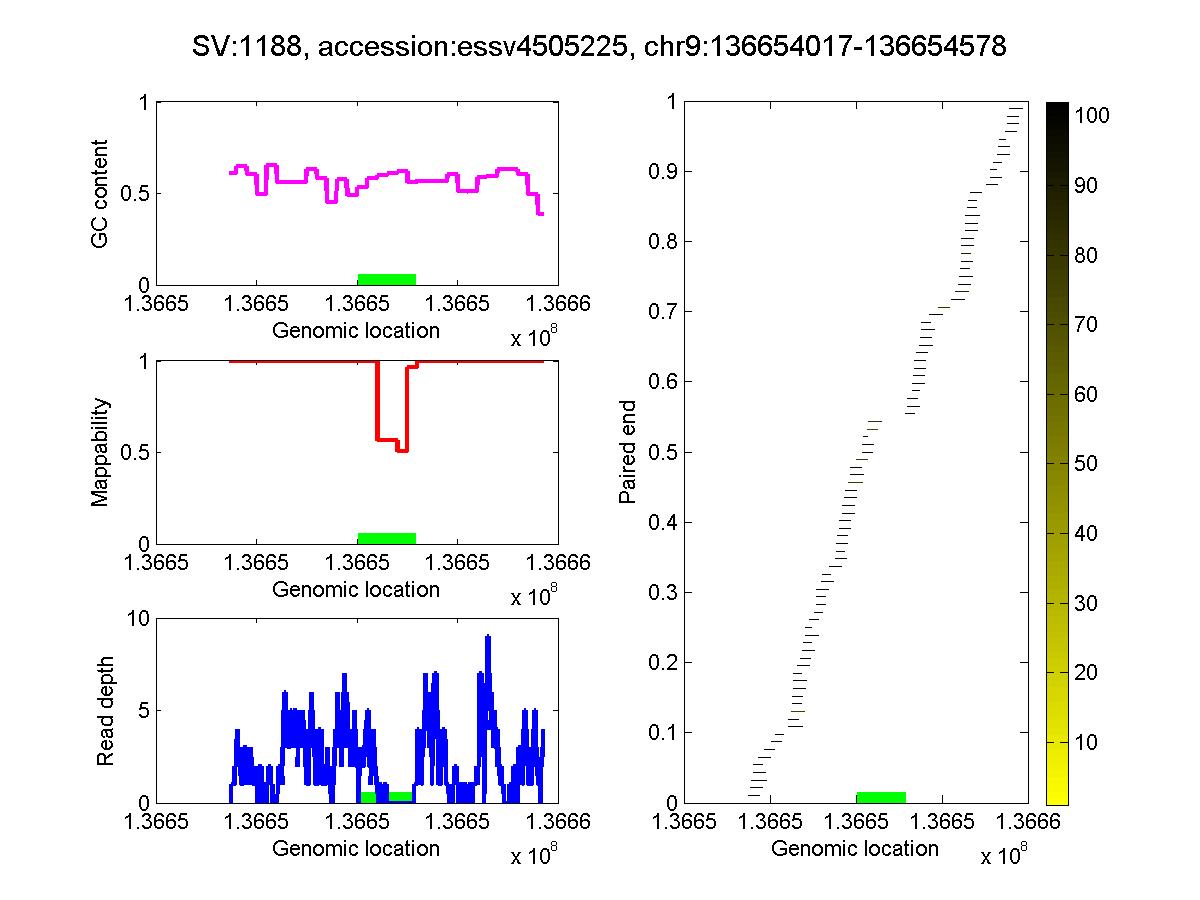

Supplement: Supplementary Materials — Supplementary data are available with this article at http://gr.xjtu.edu.cn/c/document_library/get_file?p_l_id=2403541&folderId=2539941&name=DLFE-115097.zip. Table S1 lists the complete information of suspicious variants and false positives, and the FIG directory contains the validation figures of each false positive. [file 8420547.f1.zip › 8420547.f1/FIG/SV1188.jpg]

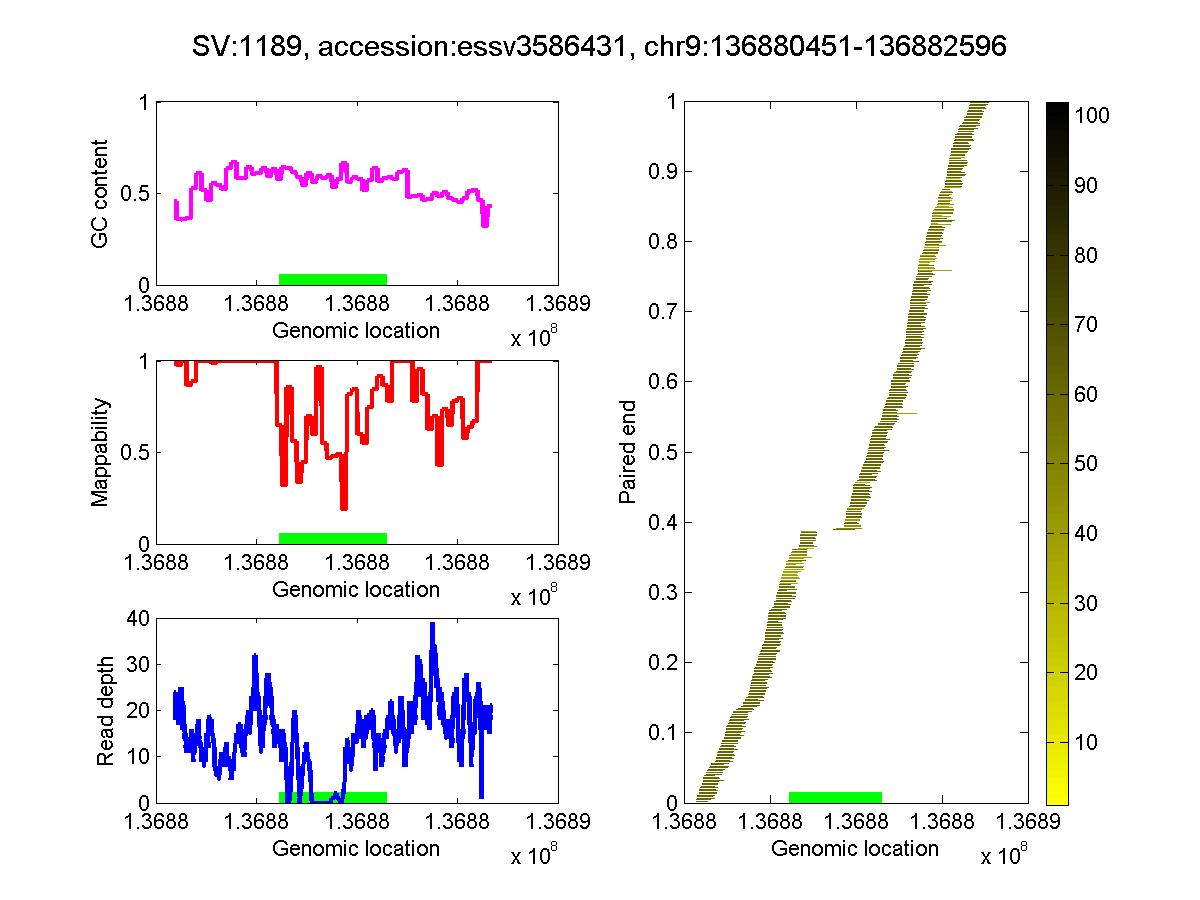

Supplement: Supplementary Materials — Supplementary data are available with this article at http://gr.xjtu.edu.cn/c/document_library/get_file?p_l_id=2403541&folderId=2539941&name=DLFE-115097.zip. Table S1 lists the complete information of suspicious variants and false positives, and the FIG directory contains the validation figures of each false positive. [file 8420547.f1.zip › 8420547.f1/FIG/SV1189.jpg]

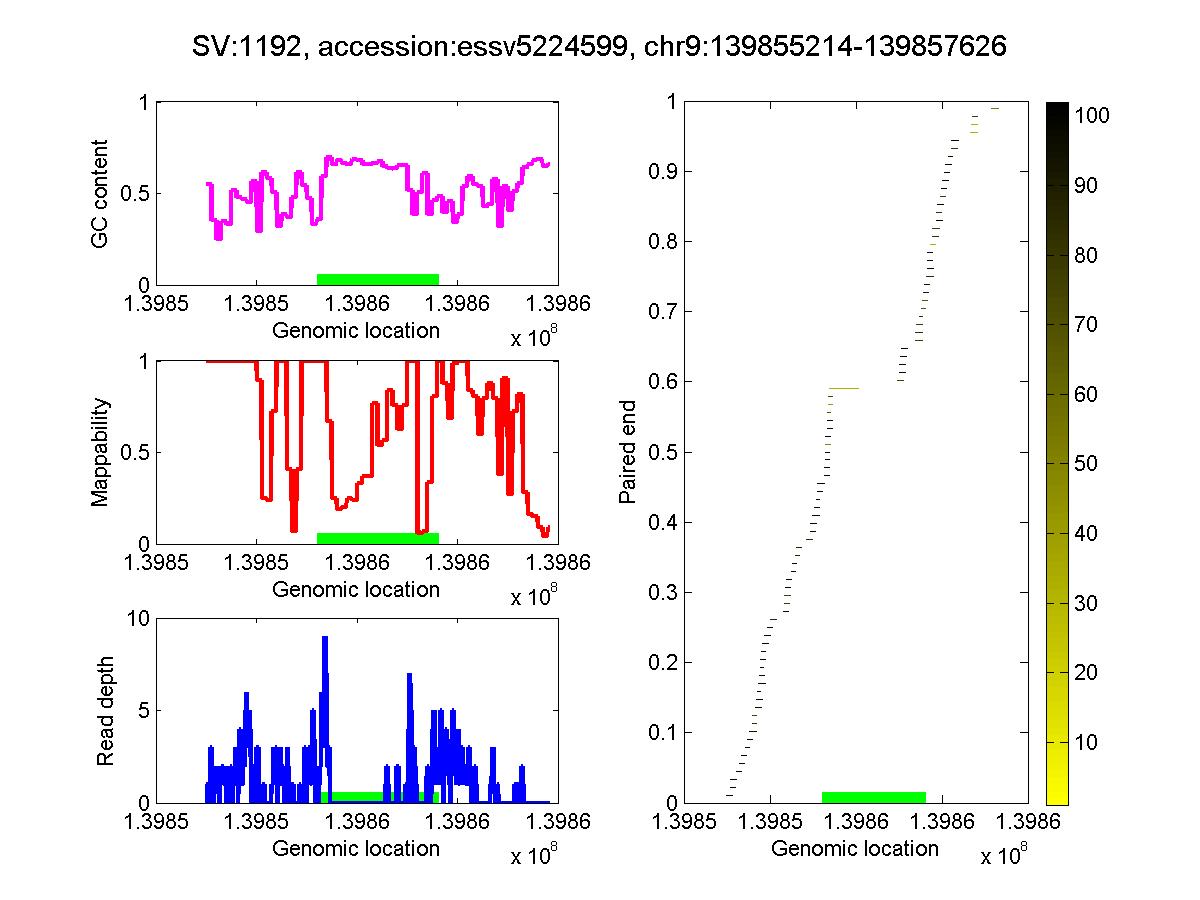

Supplement: Supplementary Materials — Supplementary data are available with this article at http://gr.xjtu.edu.cn/c/document_library/get_file?p_l_id=2403541&folderId=2539941&name=DLFE-115097.zip. Table S1 lists the complete information of suspicious variants and false positives, and the FIG directory contains the validation figures of each false positive. [file 8420547.f1.zip › 8420547.f1/FIG/SV1192.jpg]

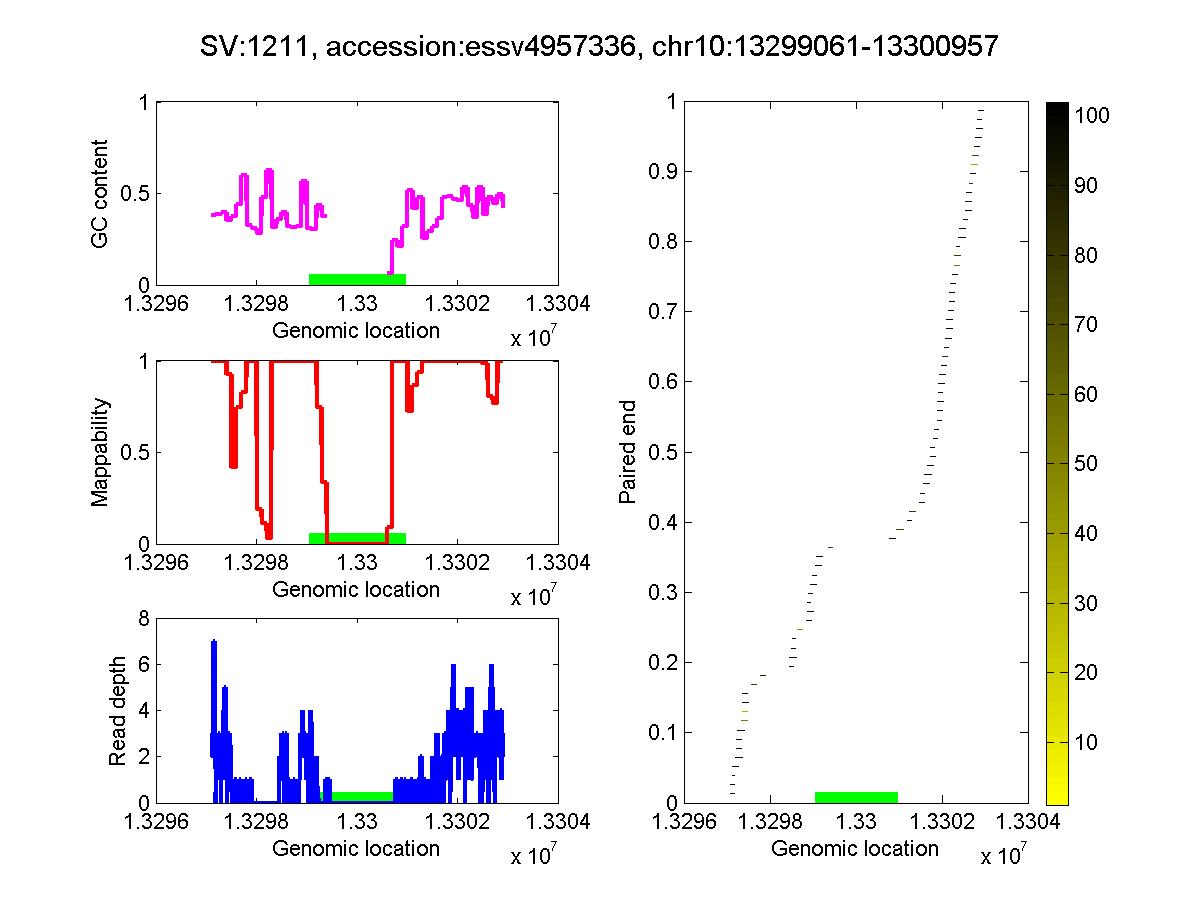

Supplement: Supplementary Materials — Supplementary data are available with this article at http://gr.xjtu.edu.cn/c/document_library/get_file?p_l_id=2403541&folderId=2539941&name=DLFE-115097.zip. Table S1 lists the complete information of suspicious variants and false positives, and the FIG directory contains the validation figures of each false positive. [file 8420547.f1.zip › 8420547.f1/FIG/SV1211.jpg]

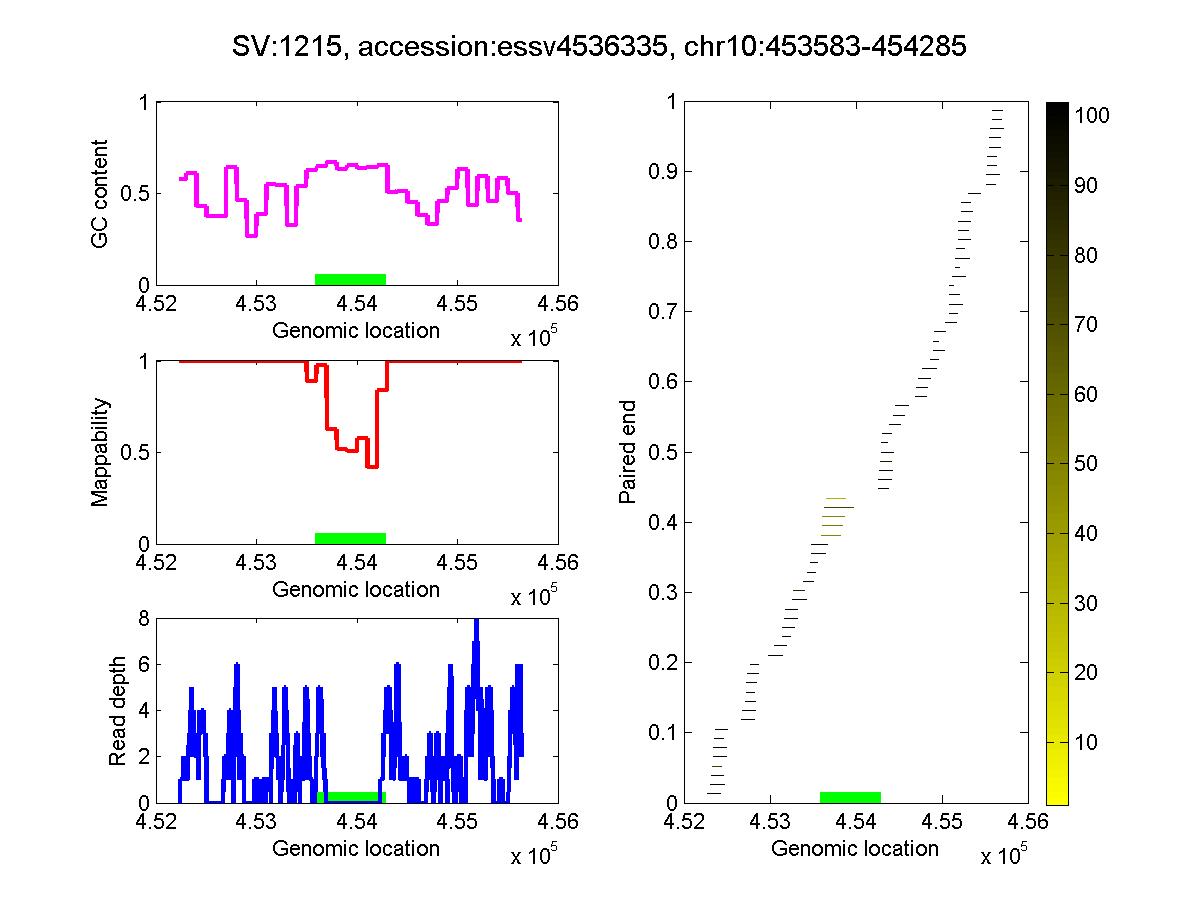

Supplement: Supplementary Materials — Supplementary data are available with this article at http://gr.xjtu.edu.cn/c/document_library/get_file?p_l_id=2403541&folderId=2539941&name=DLFE-115097.zip. Table S1 lists the complete information of suspicious variants and false positives, and the FIG directory contains the validation figures of each false positive. [file 8420547.f1.zip › 8420547.f1/FIG/SV1215.jpg]

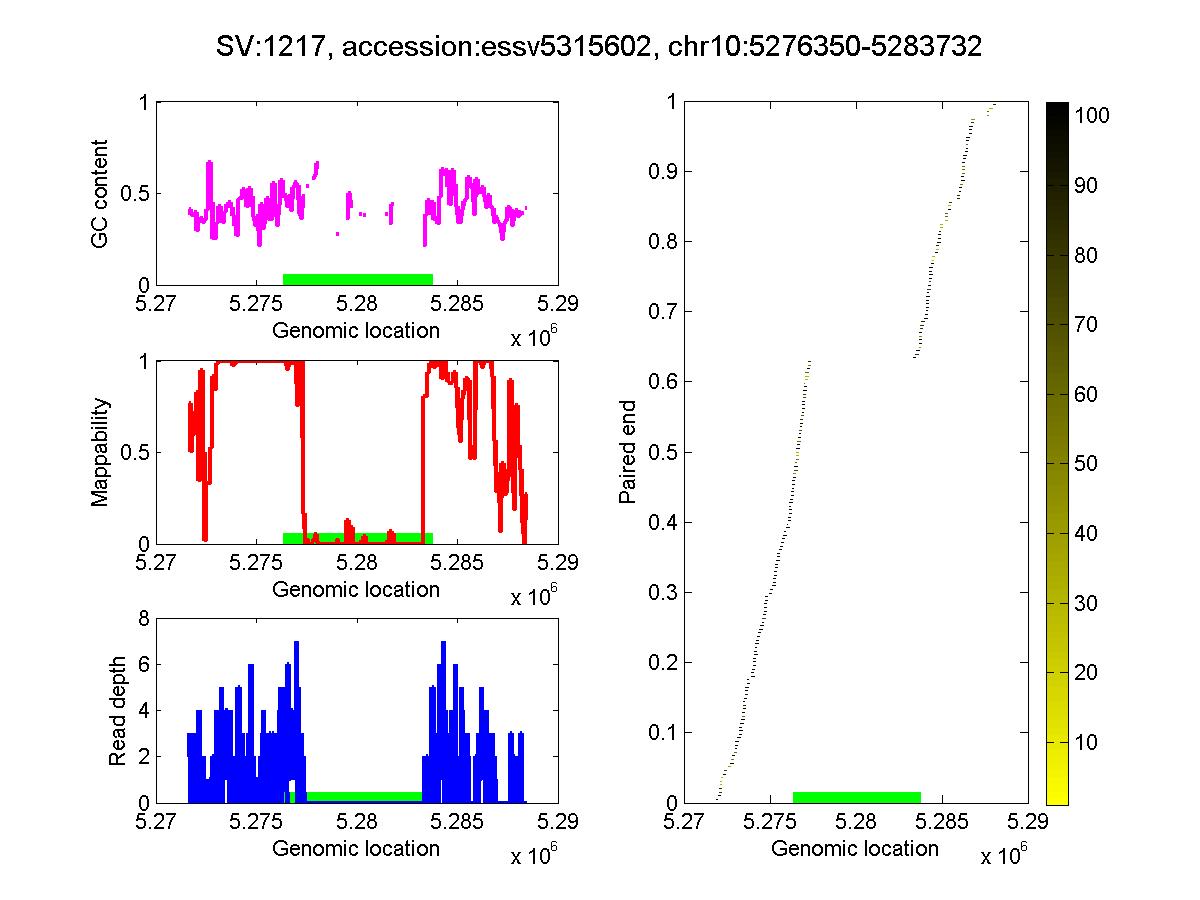

Supplement: Supplementary Materials — Supplementary data are available with this article at http://gr.xjtu.edu.cn/c/document_library/get_file?p_l_id=2403541&folderId=2539941&name=DLFE-115097.zip. Table S1 lists the complete information of suspicious variants and false positives, and the FIG directory contains the validation figures of each false positive. [file 8420547.f1.zip › 8420547.f1/FIG/SV1217.jpg]

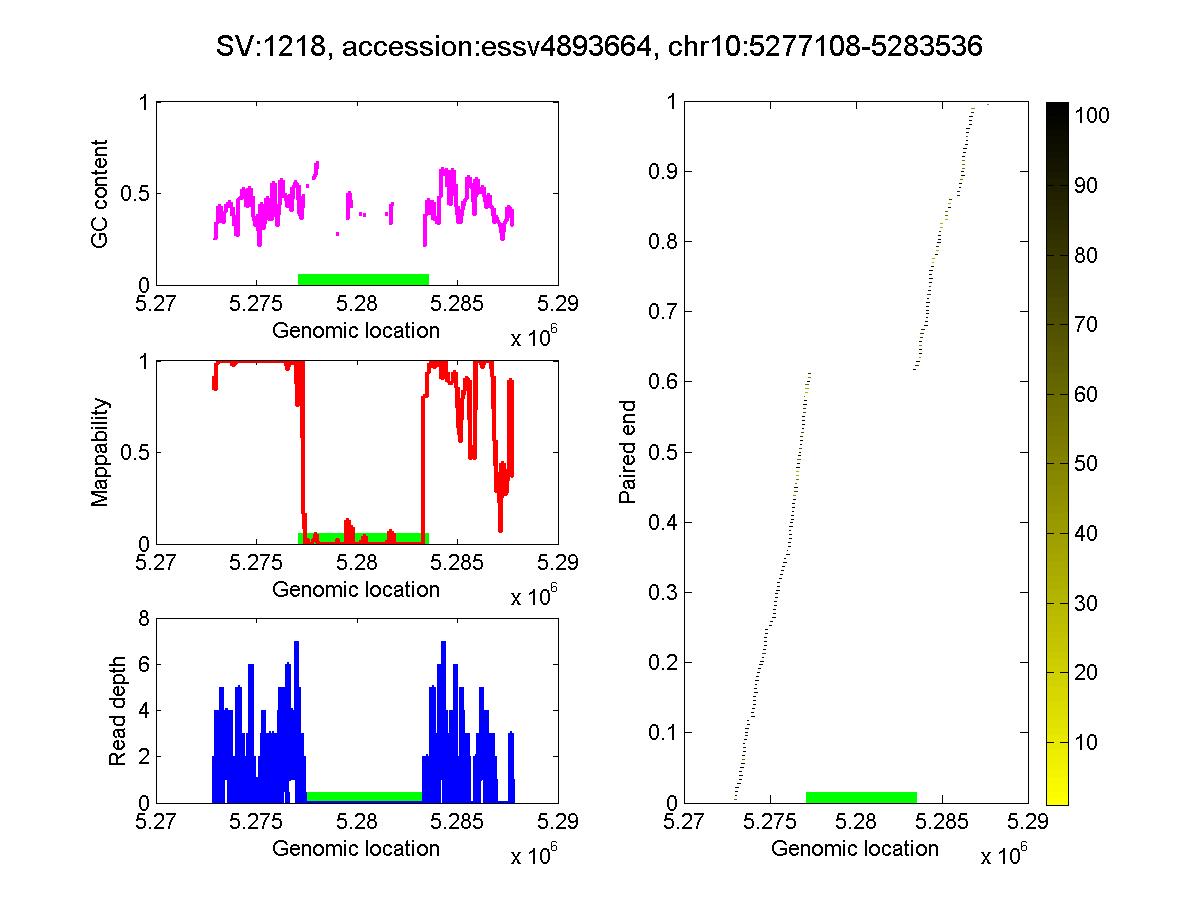

Supplement: Supplementary Materials — Supplementary data are available with this article at http://gr.xjtu.edu.cn/c/document_library/get_file?p_l_id=2403541&folderId=2539941&name=DLFE-115097.zip. Table S1 lists the complete information of suspicious variants and false positives, and the FIG directory contains the validation figures of each false positive. [file 8420547.f1.zip › 8420547.f1/FIG/SV1218.jpg]

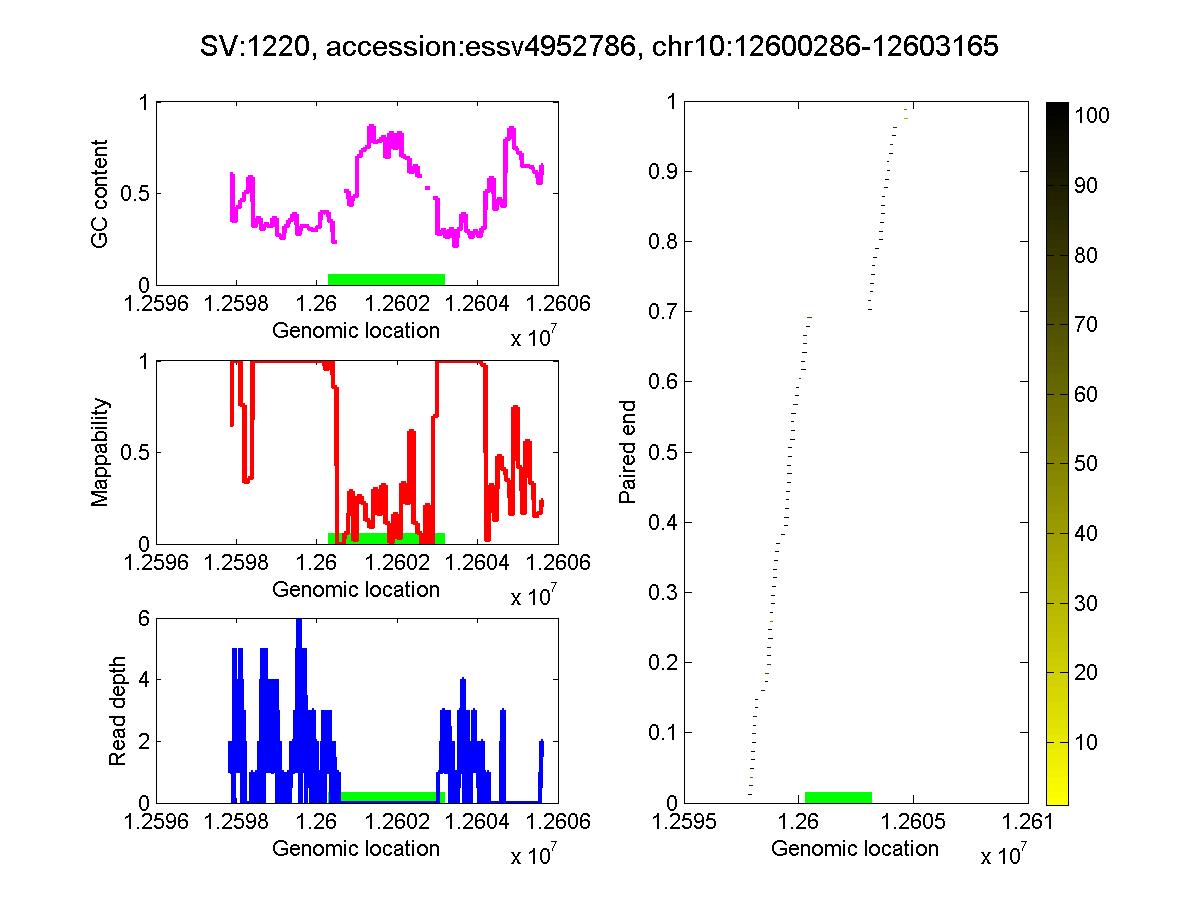

Supplement: Supplementary Materials — Supplementary data are available with this article at http://gr.xjtu.edu.cn/c/document_library/get_file?p_l_id=2403541&folderId=2539941&name=DLFE-115097.zip. Table S1 lists the complete information of suspicious variants and false positives, and the FIG directory contains the validation figures of each false positive. [file 8420547.f1.zip › 8420547.f1/FIG/SV1220.jpg]

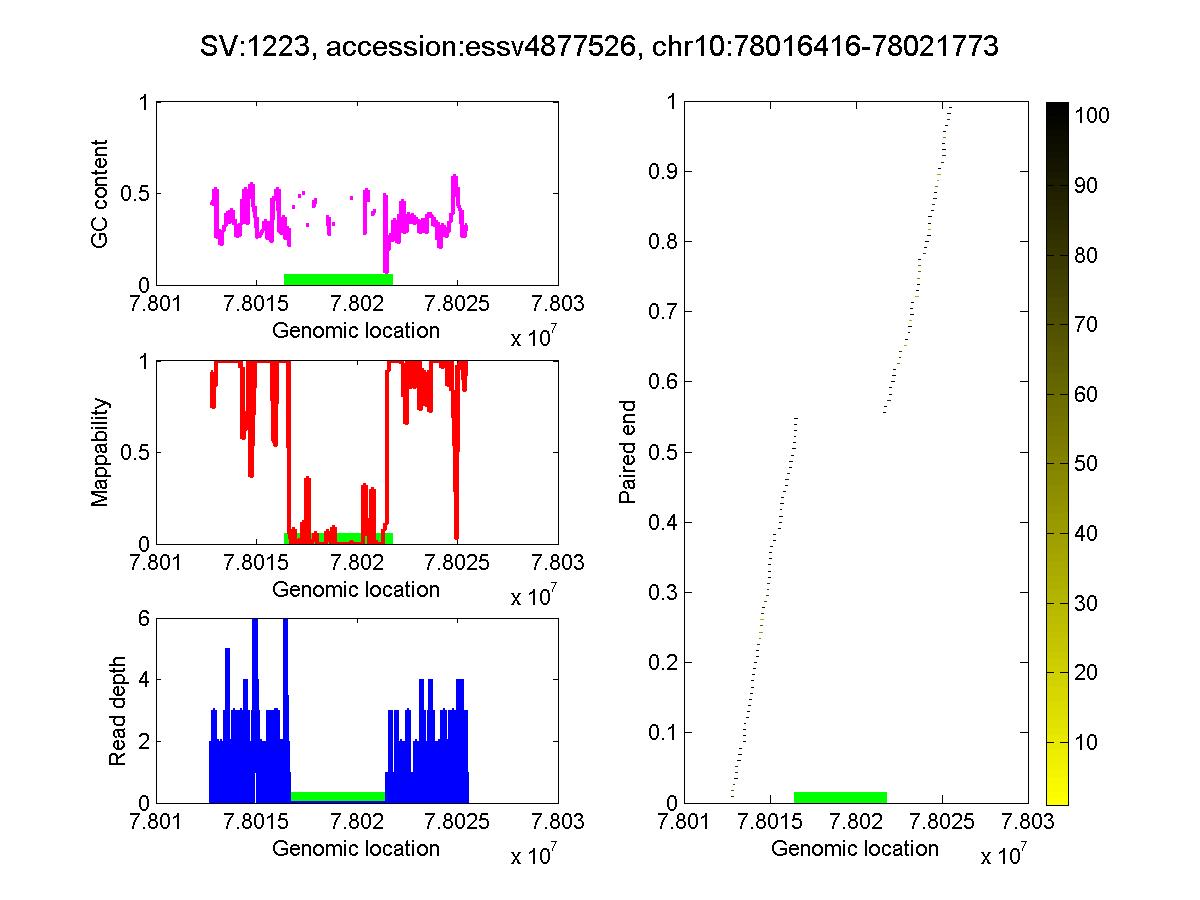

Supplement: Supplementary Materials — Supplementary data are available with this article at http://gr.xjtu.edu.cn/c/document_library/get_file?p_l_id=2403541&folderId=2539941&name=DLFE-115097.zip. Table S1 lists the complete information of suspicious variants and false positives, and the FIG directory contains the validation figures of each false positive. [file 8420547.f1.zip › 8420547.f1/FIG/SV1223.jpg]

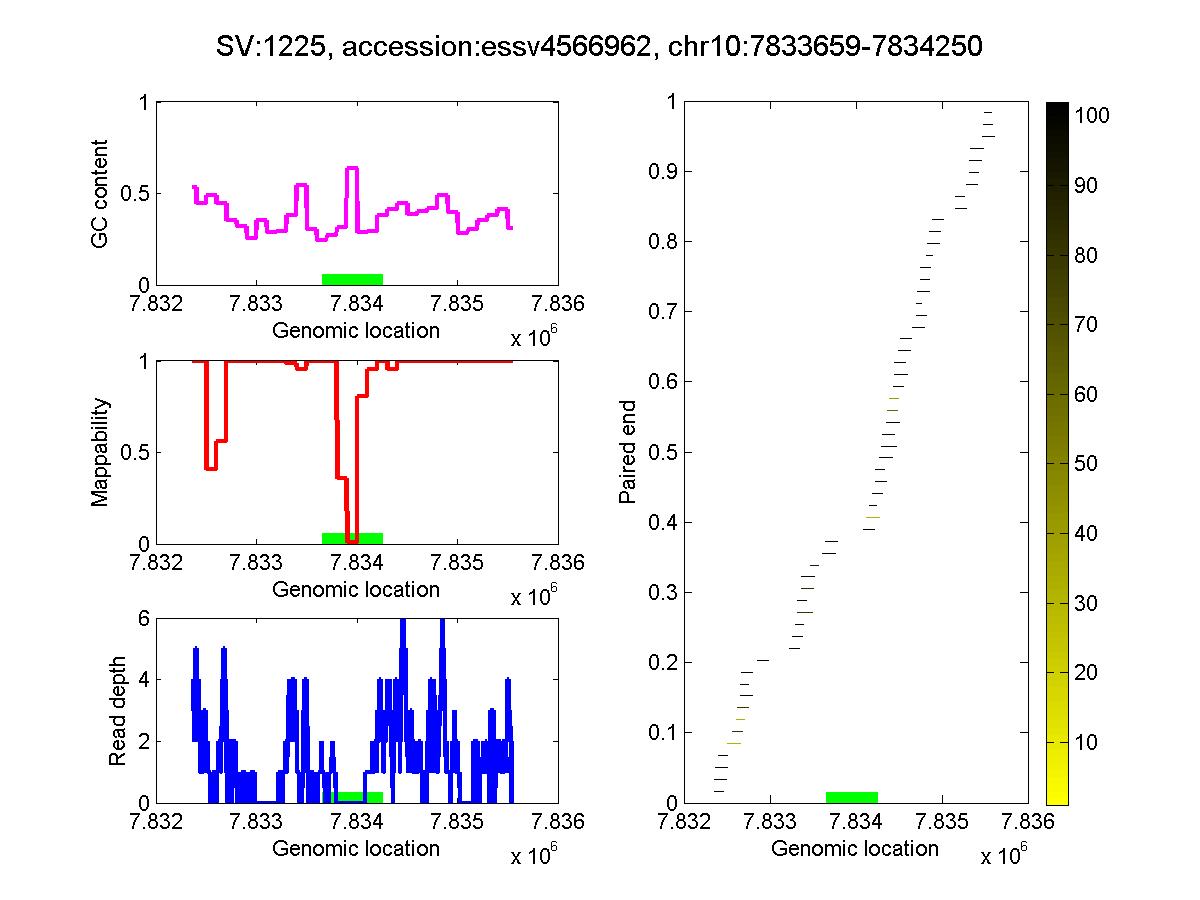

Supplement: Supplementary Materials — Supplementary data are available with this article at http://gr.xjtu.edu.cn/c/document_library/get_file?p_l_id=2403541&folderId=2539941&name=DLFE-115097.zip. Table S1 lists the complete information of suspicious variants and false positives, and the FIG directory contains the validation figures of each false positive. [file 8420547.f1.zip › 8420547.f1/FIG/SV1225.jpg]

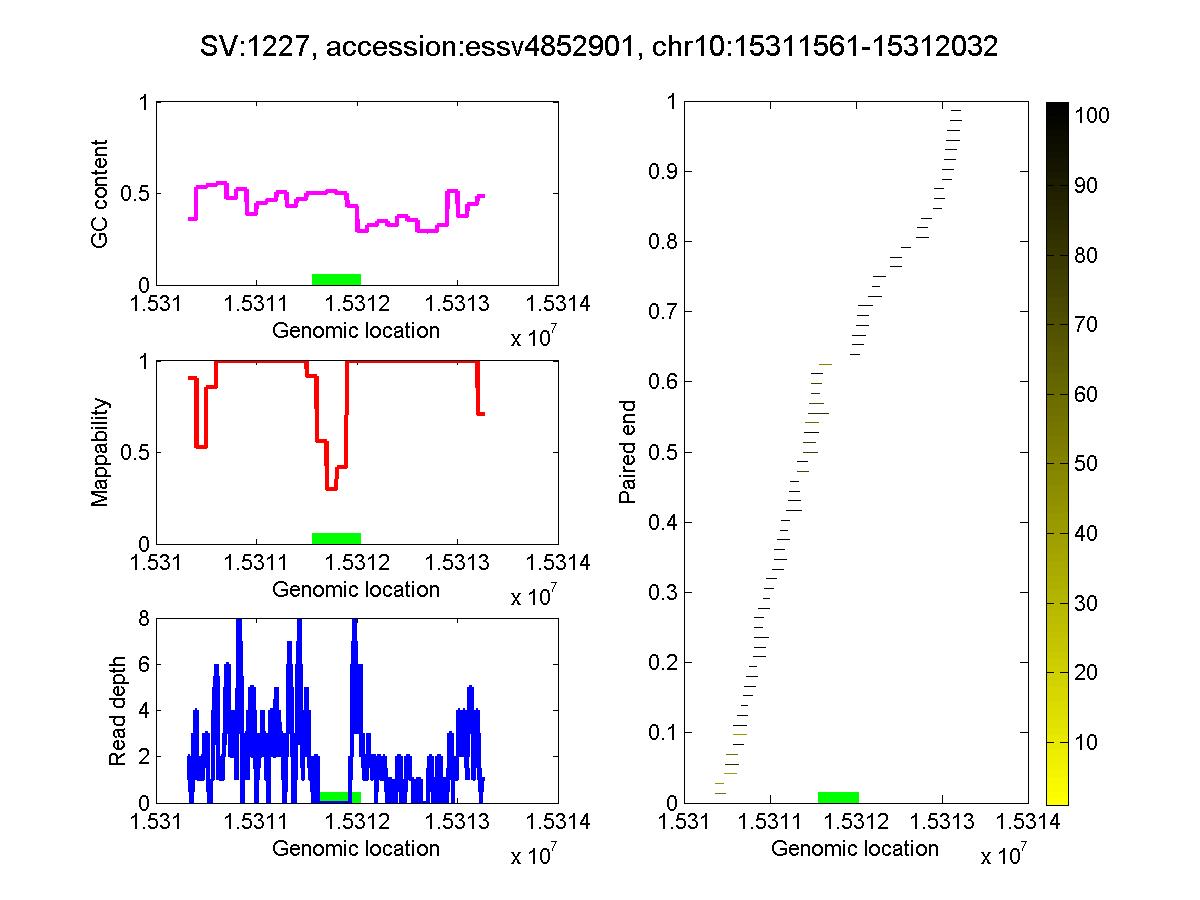

Supplement: Supplementary Materials — Supplementary data are available with this article at http://gr.xjtu.edu.cn/c/document_library/get_file?p_l_id=2403541&folderId=2539941&name=DLFE-115097.zip. Table S1 lists the complete information of suspicious variants and false positives, and the FIG directory contains the validation figures of each false positive. [file 8420547.f1.zip › 8420547.f1/FIG/SV1227.jpg]

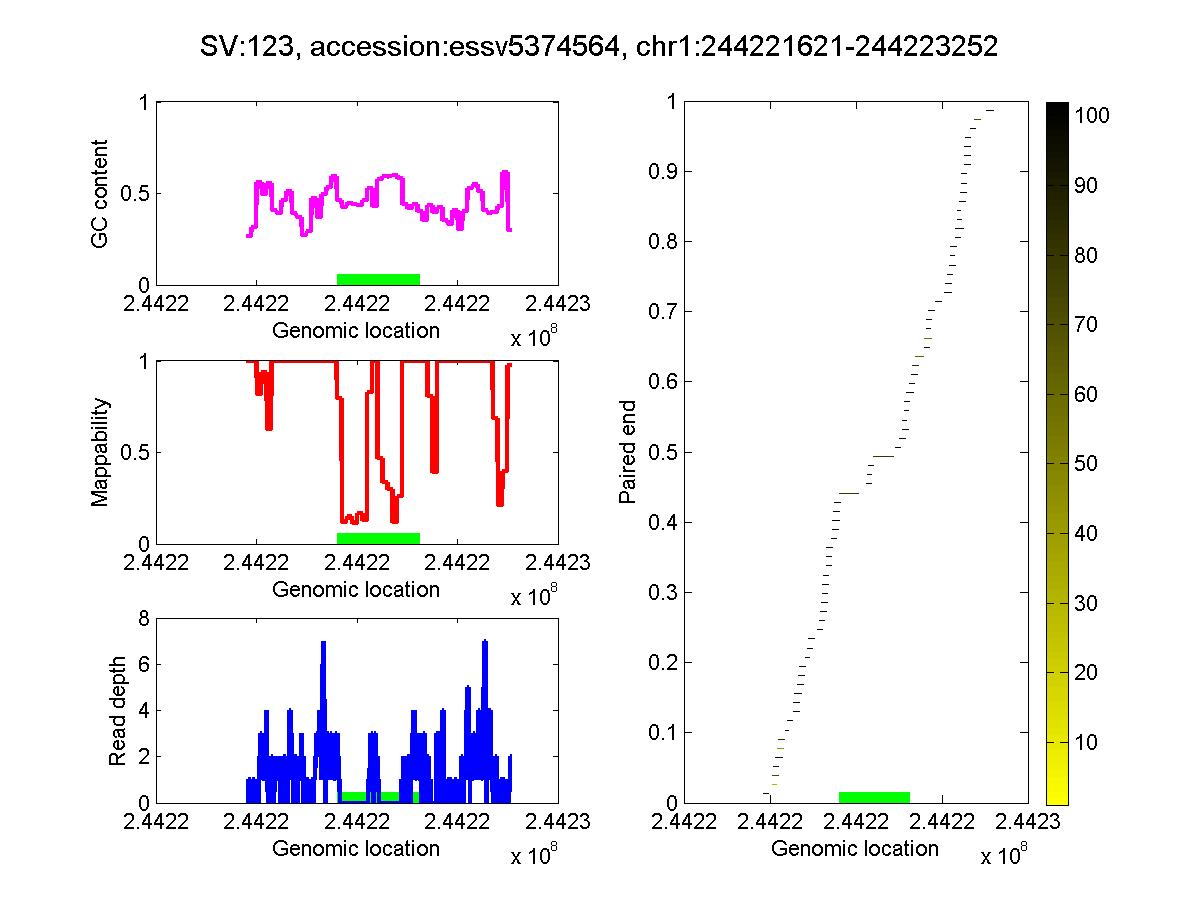

Supplement: Supplementary Materials — Supplementary data are available with this article at http://gr.xjtu.edu.cn/c/document_library/get_file?p_l_id=2403541&folderId=2539941&name=DLFE-115097.zip. Table S1 lists the complete information of suspicious variants and false positives, and the FIG directory contains the validation figures of each false positive. [file 8420547.f1.zip › 8420547.f1/FIG/SV123.jpg]

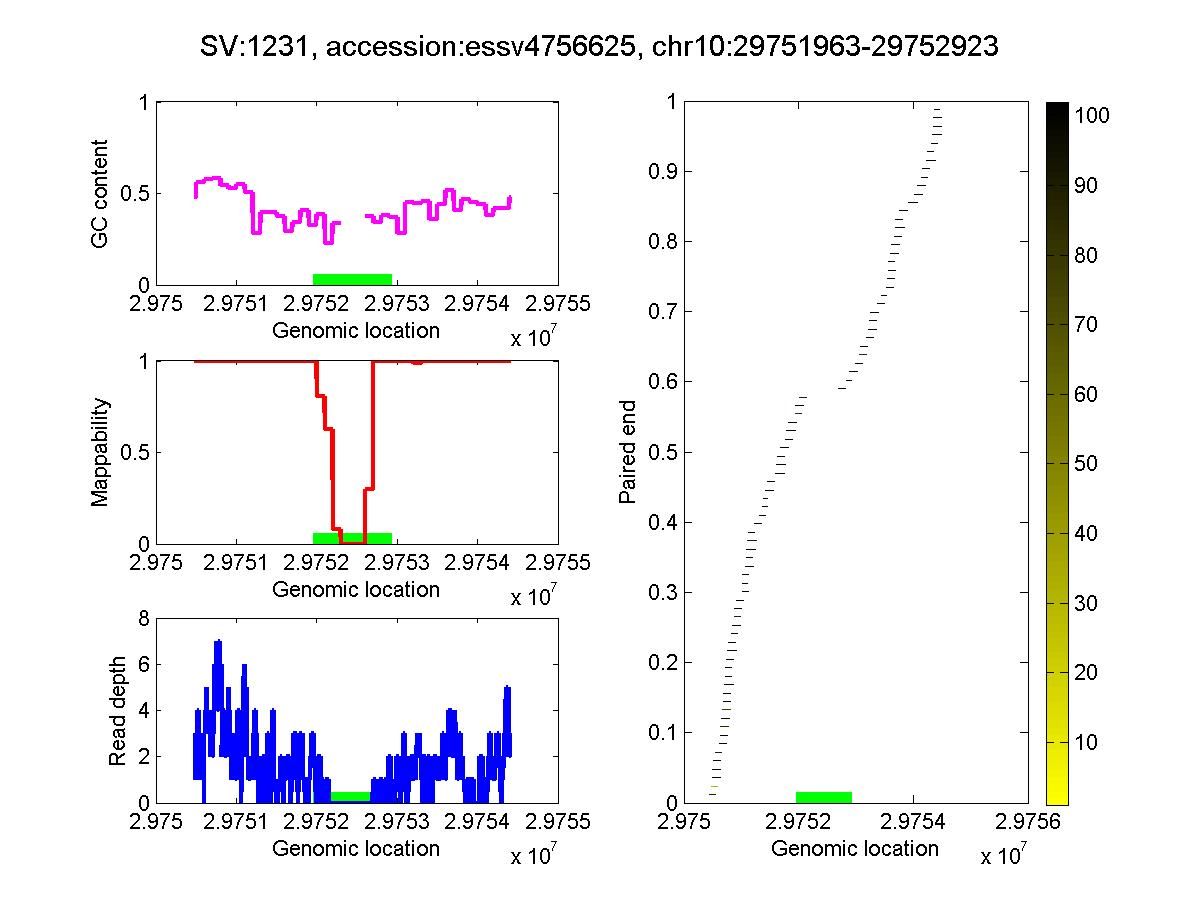

Supplement: Supplementary Materials — Supplementary data are available with this article at http://gr.xjtu.edu.cn/c/document_library/get_file?p_l_id=2403541&folderId=2539941&name=DLFE-115097.zip. Table S1 lists the complete information of suspicious variants and false positives, and the FIG directory contains the validation figures of each false positive. [file 8420547.f1.zip › 8420547.f1/FIG/SV1231.jpg]

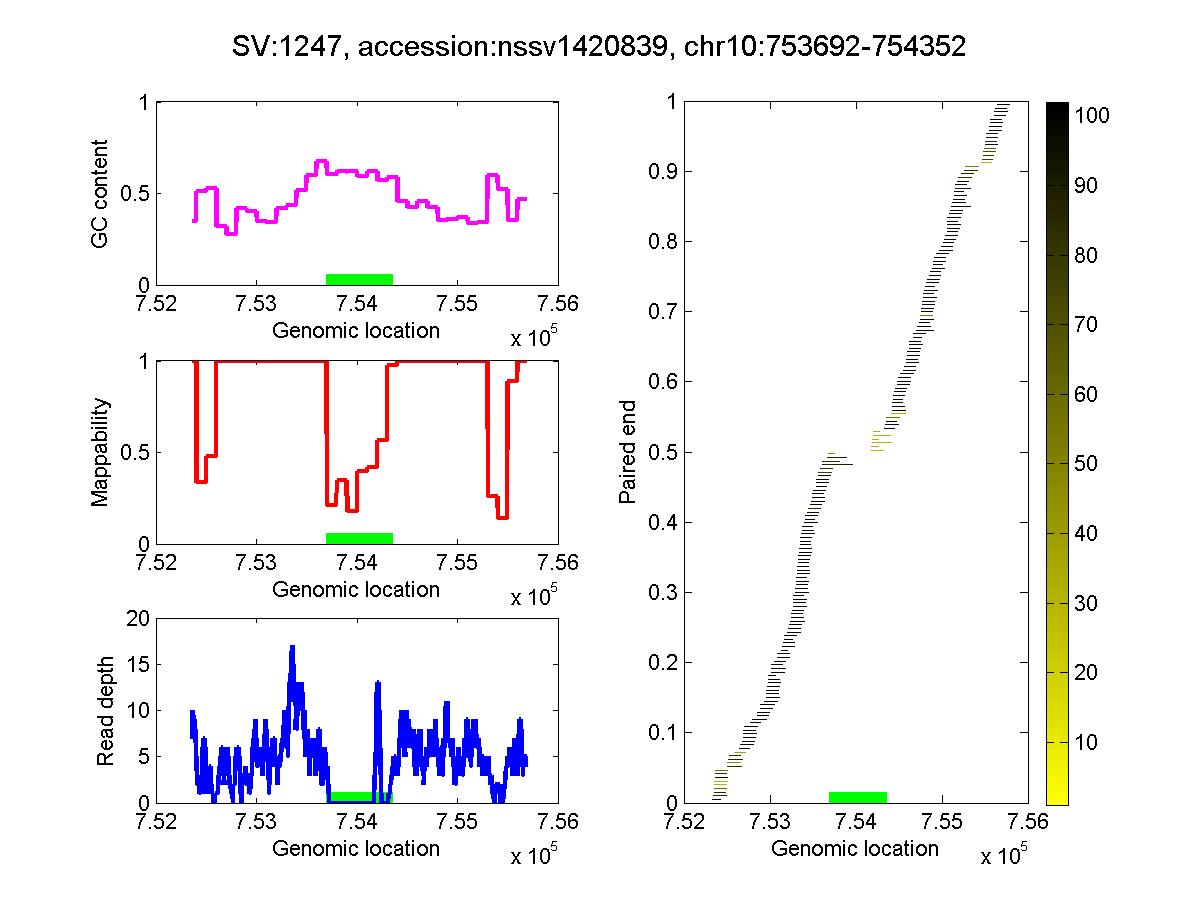

Supplement: Supplementary Materials — Supplementary data are available with this article at http://gr.xjtu.edu.cn/c/document_library/get_file?p_l_id=2403541&folderId=2539941&name=DLFE-115097.zip. Table S1 lists the complete information of suspicious variants and false positives, and the FIG directory contains the validation figures of each false positive. [file 8420547.f1.zip › 8420547.f1/FIG/SV1247.jpg]

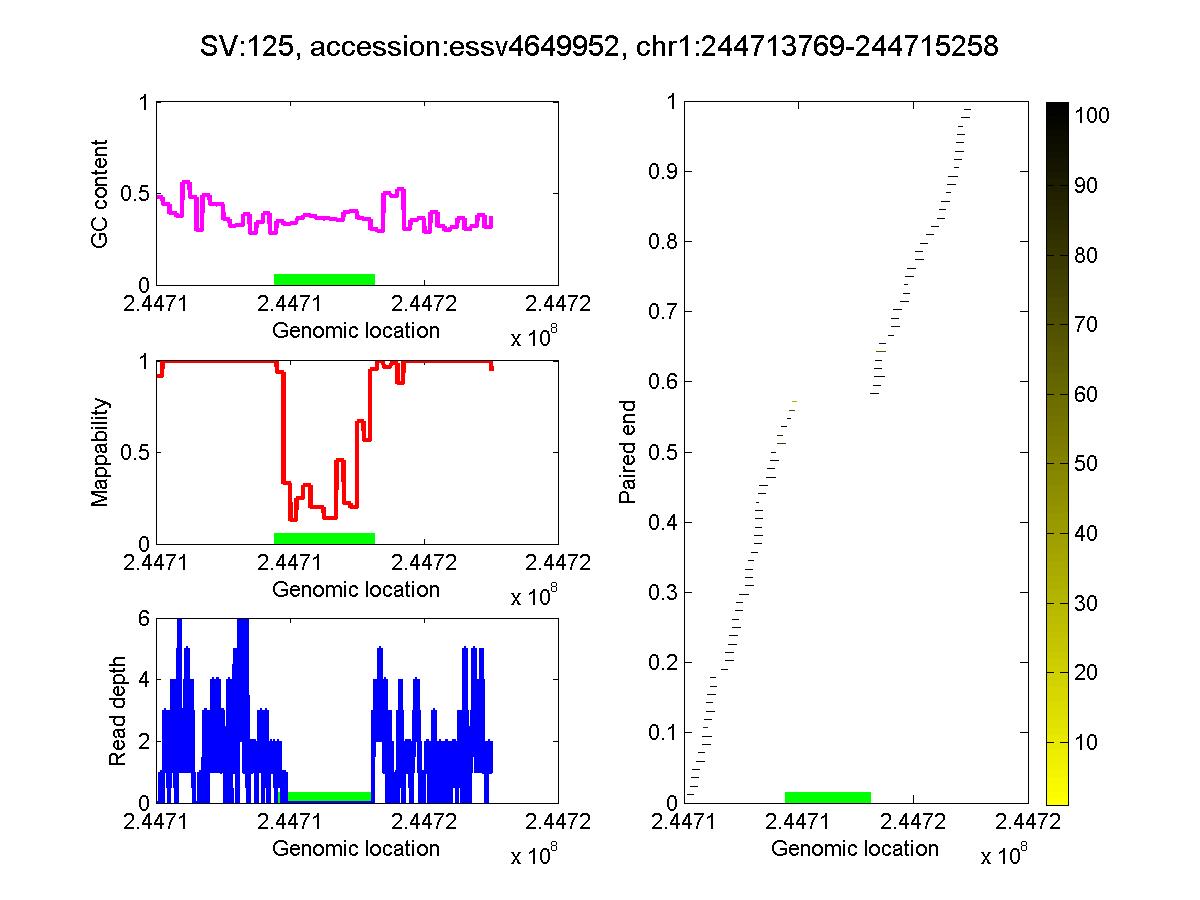

Supplement: Supplementary Materials — Supplementary data are available with this article at http://gr.xjtu.edu.cn/c/document_library/get_file?p_l_id=2403541&folderId=2539941&name=DLFE-115097.zip. Table S1 lists the complete information of suspicious variants and false positives, and the FIG directory contains the validation figures of each false positive. [file 8420547.f1.zip › 8420547.f1/FIG/SV125.jpg]

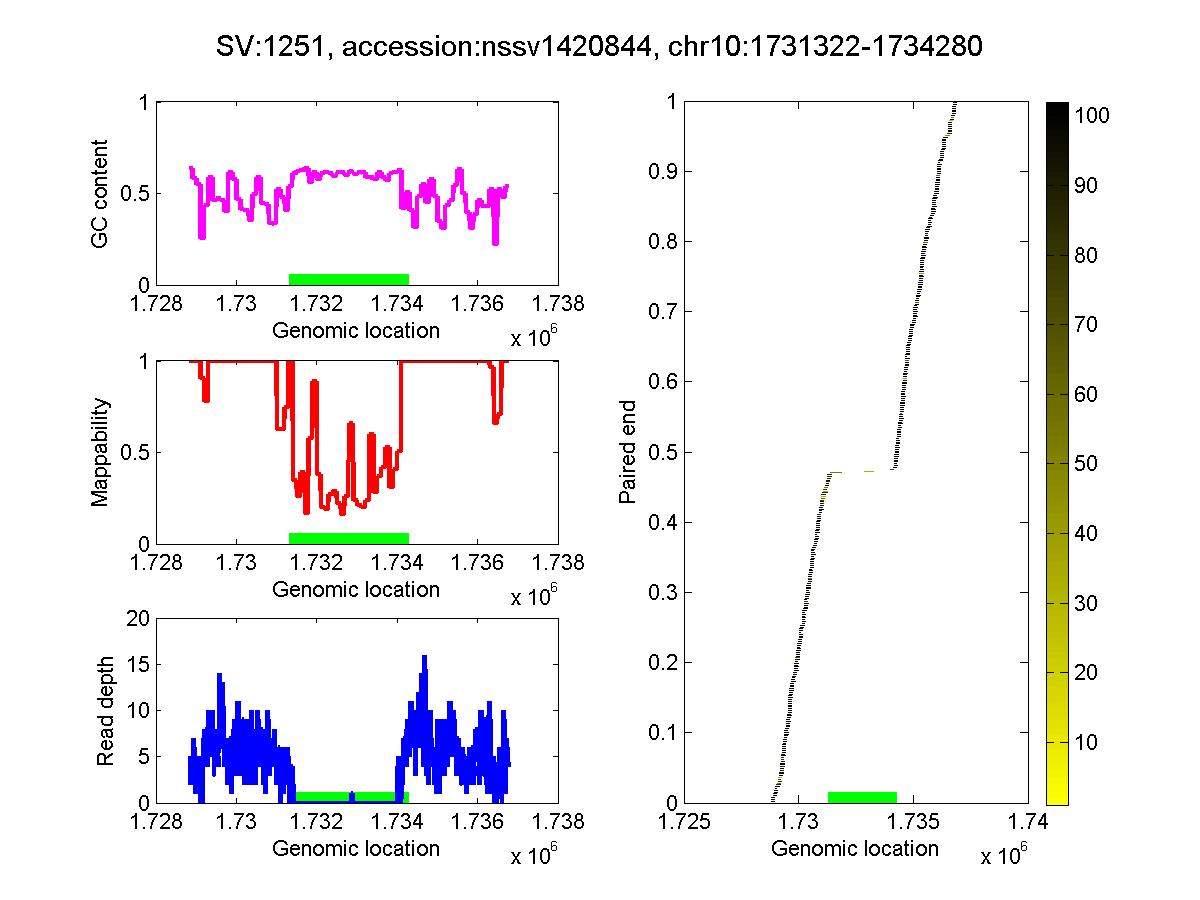

Supplement: Supplementary Materials — Supplementary data are available with this article at http://gr.xjtu.edu.cn/c/document_library/get_file?p_l_id=2403541&folderId=2539941&name=DLFE-115097.zip. Table S1 lists the complete information of suspicious variants and false positives, and the FIG directory contains the validation figures of each false positive. [file 8420547.f1.zip › 8420547.f1/FIG/SV1251.jpg]

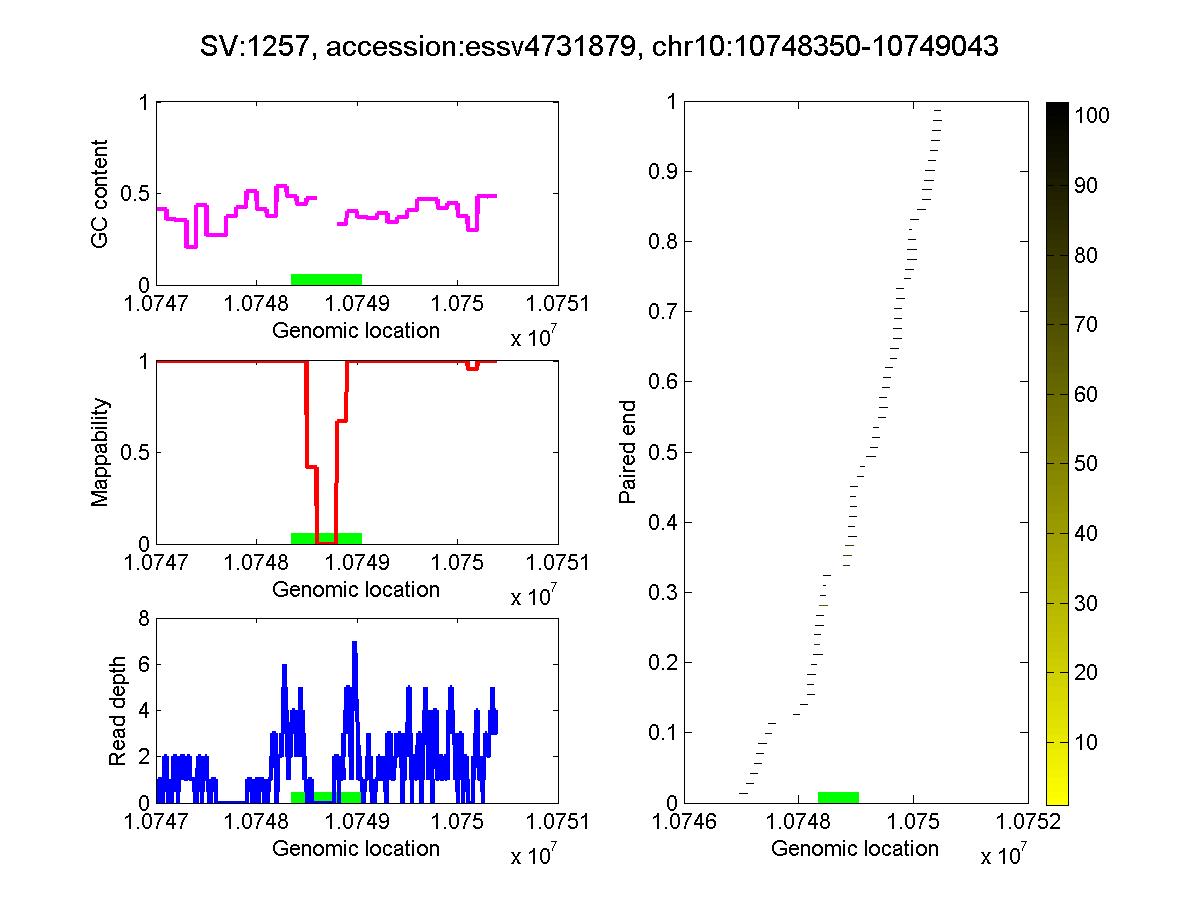

Supplement: Supplementary Materials — Supplementary data are available with this article at http://gr.xjtu.edu.cn/c/document_library/get_file?p_l_id=2403541&folderId=2539941&name=DLFE-115097.zip. Table S1 lists the complete information of suspicious variants and false positives, and the FIG directory contains the validation figures of each false positive. [file 8420547.f1.zip › 8420547.f1/FIG/SV1257.jpg]

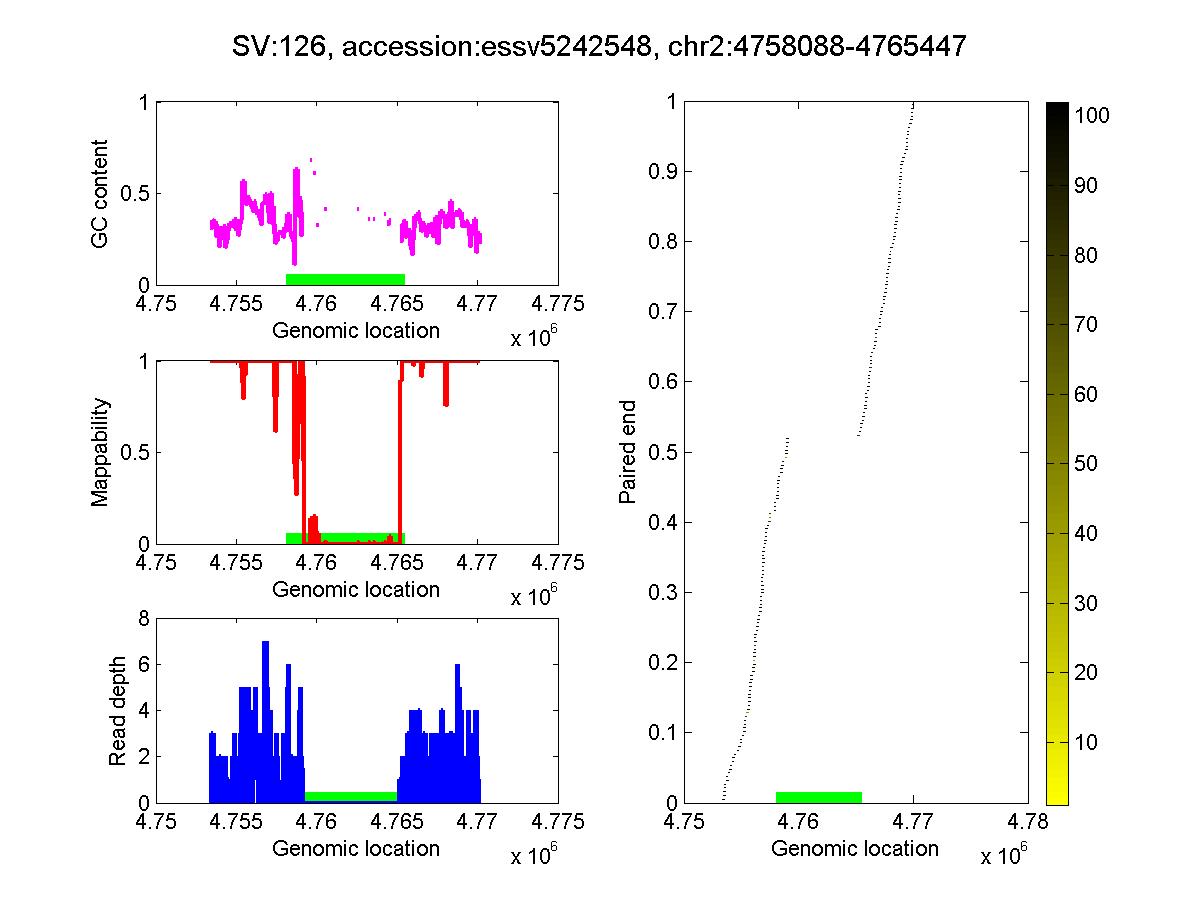

Supplement: Supplementary Materials — Supplementary data are available with this article at http://gr.xjtu.edu.cn/c/document_library/get_file?p_l_id=2403541&folderId=2539941&name=DLFE-115097.zip. Table S1 lists the complete information of suspicious variants and false positives, and the FIG directory contains the validation figures of each false positive. [file 8420547.f1.zip › 8420547.f1/FIG/SV126.jpg]

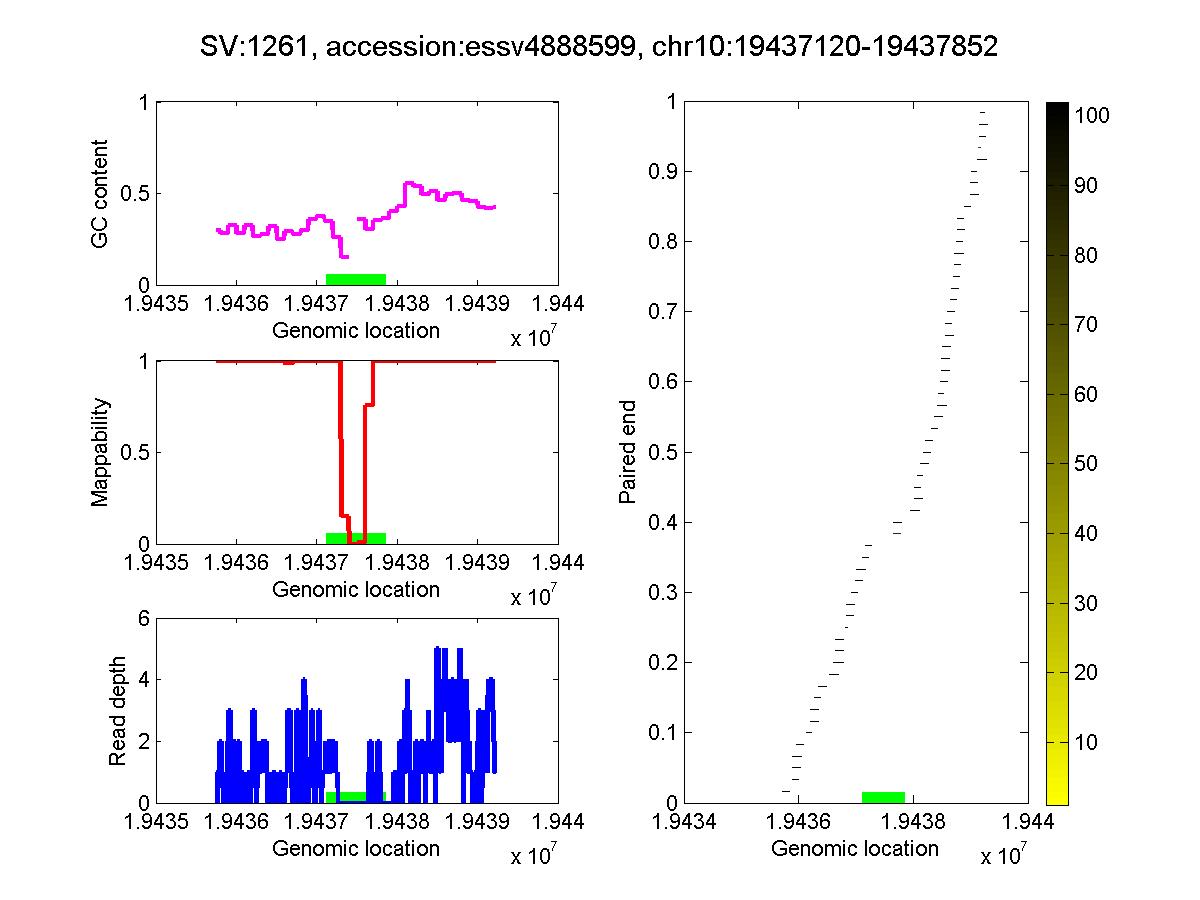

Supplement: Supplementary Materials — Supplementary data are available with this article at http://gr.xjtu.edu.cn/c/document_library/get_file?p_l_id=2403541&folderId=2539941&name=DLFE-115097.zip. Table S1 lists the complete information of suspicious variants and false positives, and the FIG directory contains the validation figures of each false positive. [file 8420547.f1.zip › 8420547.f1/FIG/SV1261.jpg]

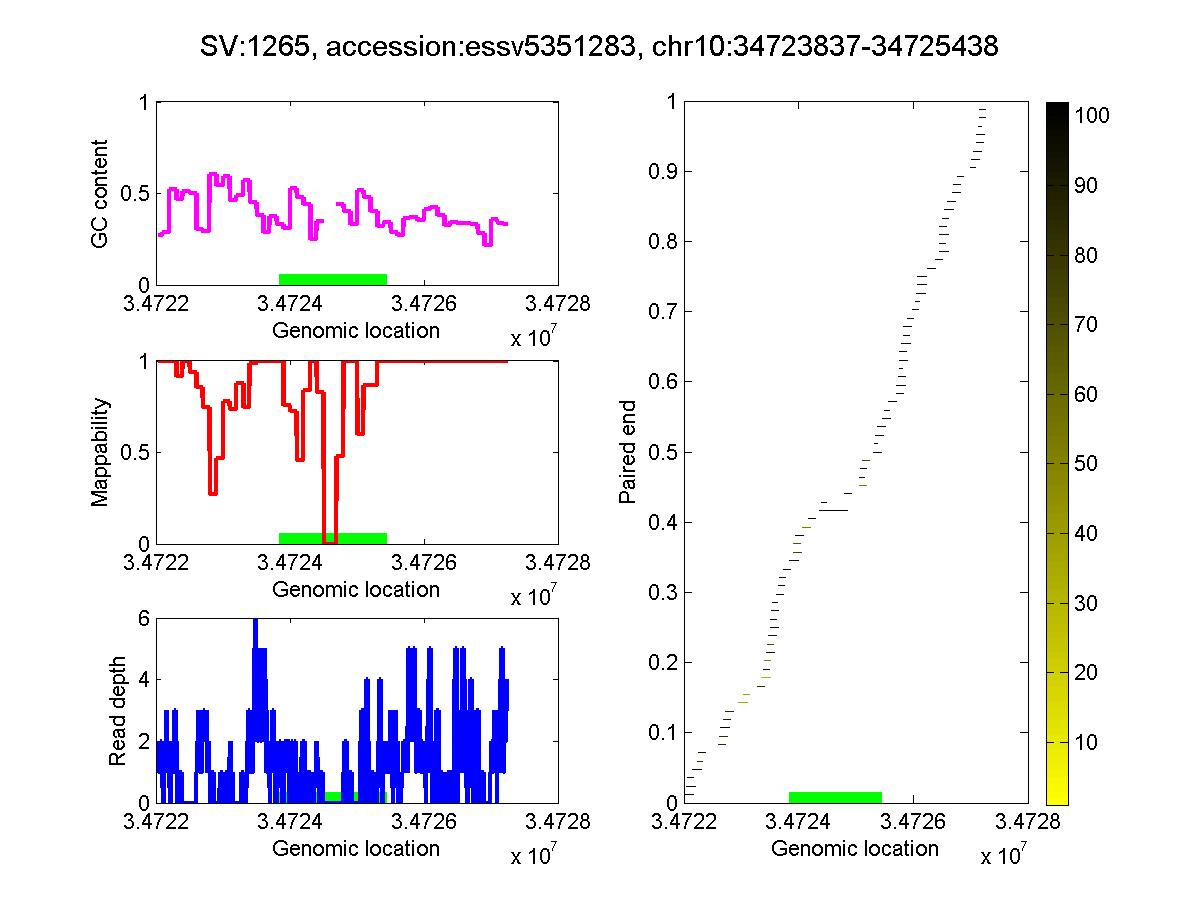

Supplement: Supplementary Materials — Supplementary data are available with this article at http://gr.xjtu.edu.cn/c/document_library/get_file?p_l_id=2403541&folderId=2539941&name=DLFE-115097.zip. Table S1 lists the complete information of suspicious variants and false positives, and the FIG directory contains the validation figures of each false positive. [file 8420547.f1.zip › 8420547.f1/FIG/SV1265.jpg]

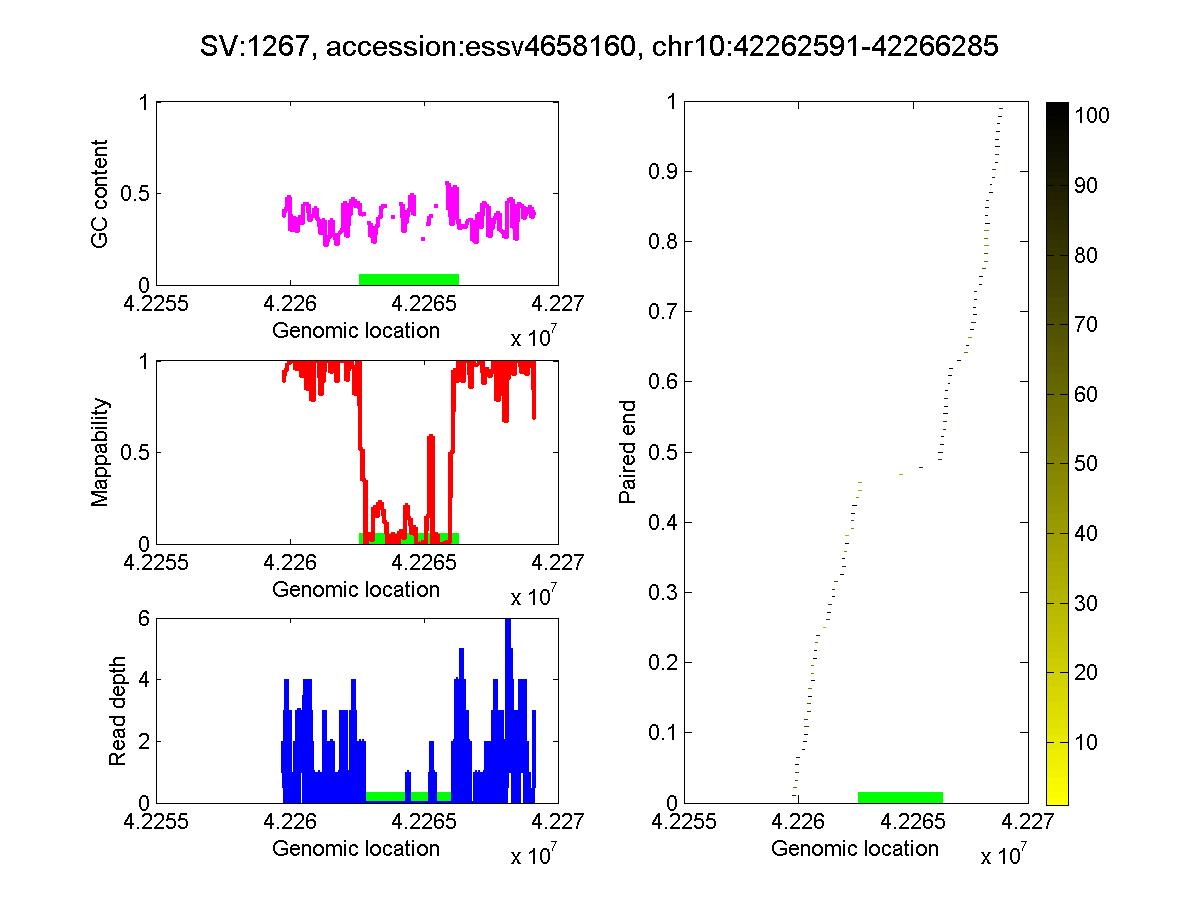

Supplement: Supplementary Materials — Supplementary data are available with this article at http://gr.xjtu.edu.cn/c/document_library/get_file?p_l_id=2403541&folderId=2539941&name=DLFE-115097.zip. Table S1 lists the complete information of suspicious variants and false positives, and the FIG directory contains the validation figures of each false positive. [file 8420547.f1.zip › 8420547.f1/FIG/SV1267.jpg]

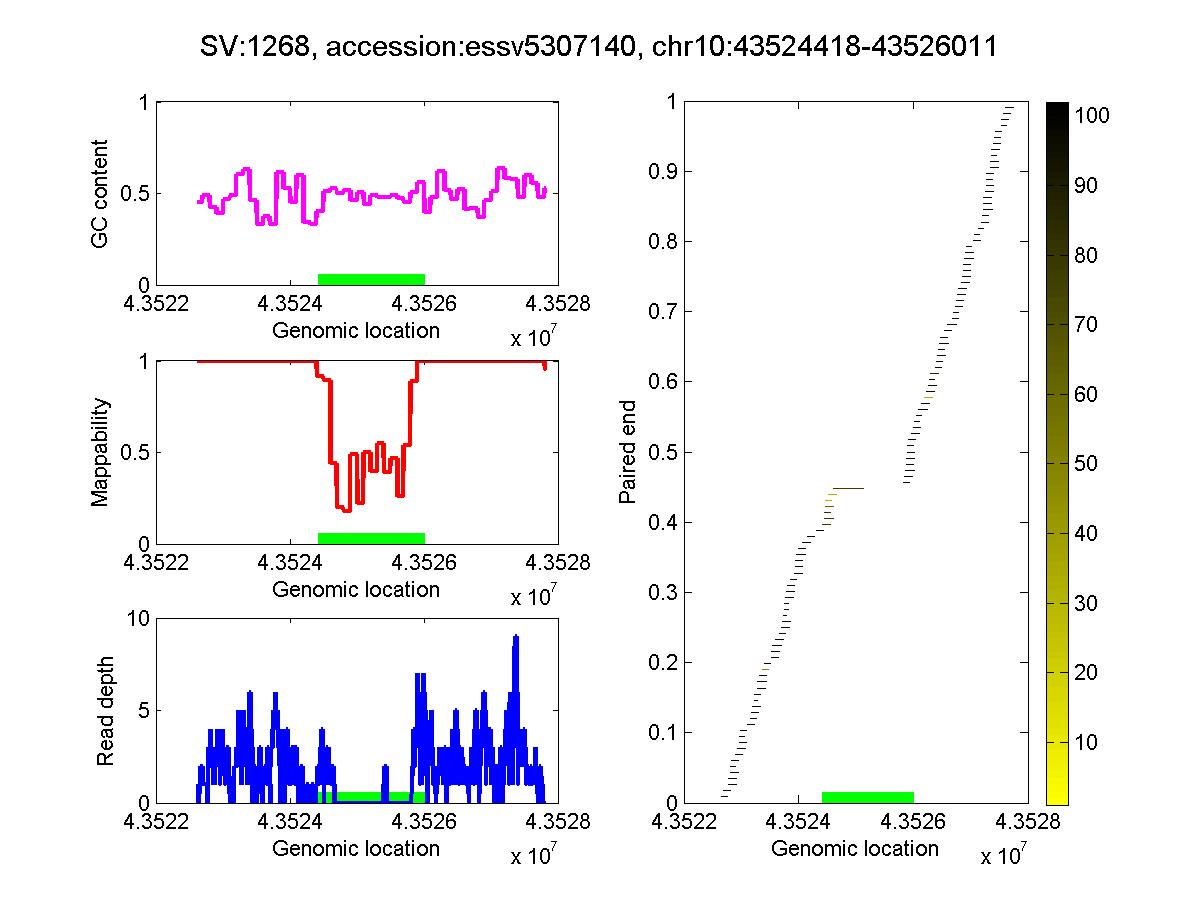

Supplement: Supplementary Materials — Supplementary data are available with this article at http://gr.xjtu.edu.cn/c/document_library/get_file?p_l_id=2403541&folderId=2539941&name=DLFE-115097.zip. Table S1 lists the complete information of suspicious variants and false positives, and the FIG directory contains the validation figures of each false positive. [file 8420547.f1.zip › 8420547.f1/FIG/SV1268.jpg]

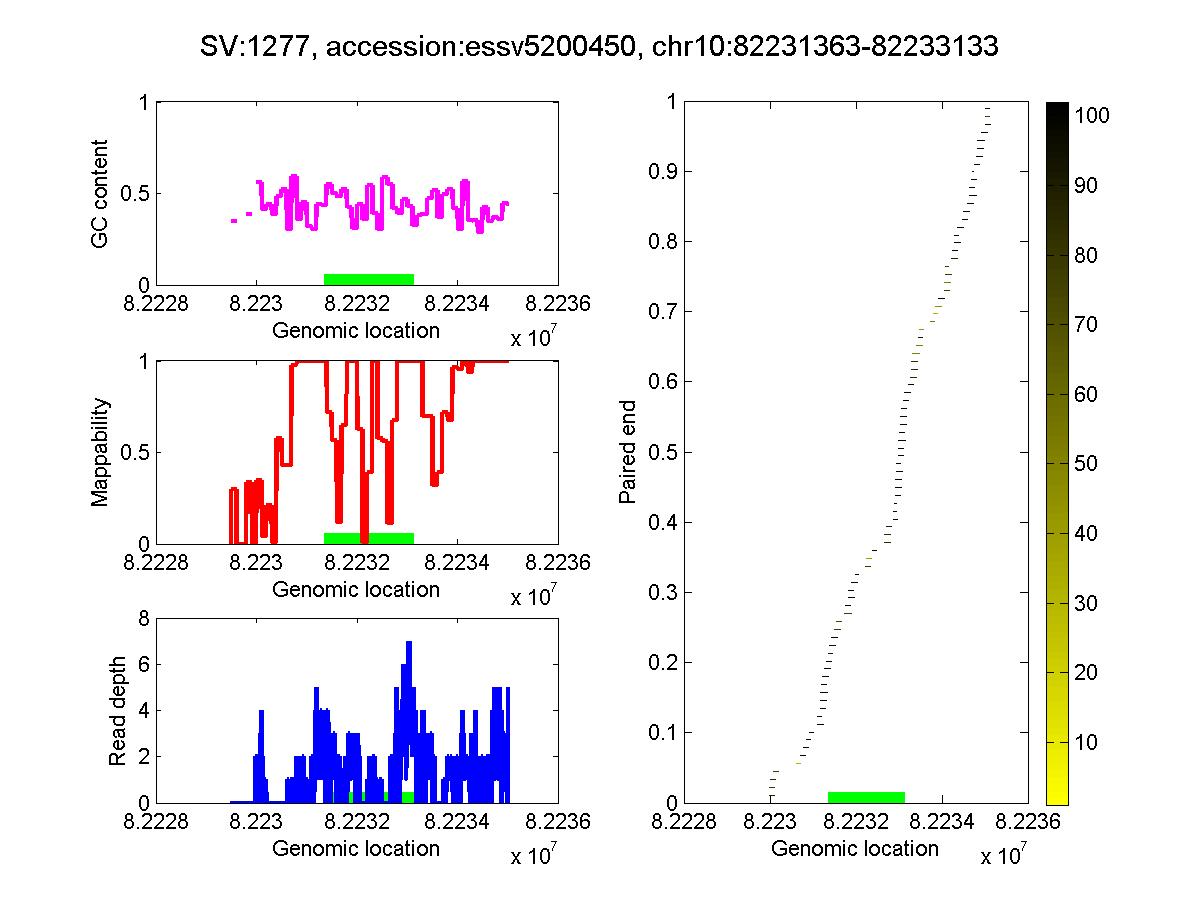

Supplement: Supplementary Materials — Supplementary data are available with this article at http://gr.xjtu.edu.cn/c/document_library/get_file?p_l_id=2403541&folderId=2539941&name=DLFE-115097.zip. Table S1 lists the complete information of suspicious variants and false positives, and the FIG directory contains the validation figures of each false positive. [file 8420547.f1.zip › 8420547.f1/FIG/SV1277.jpg]

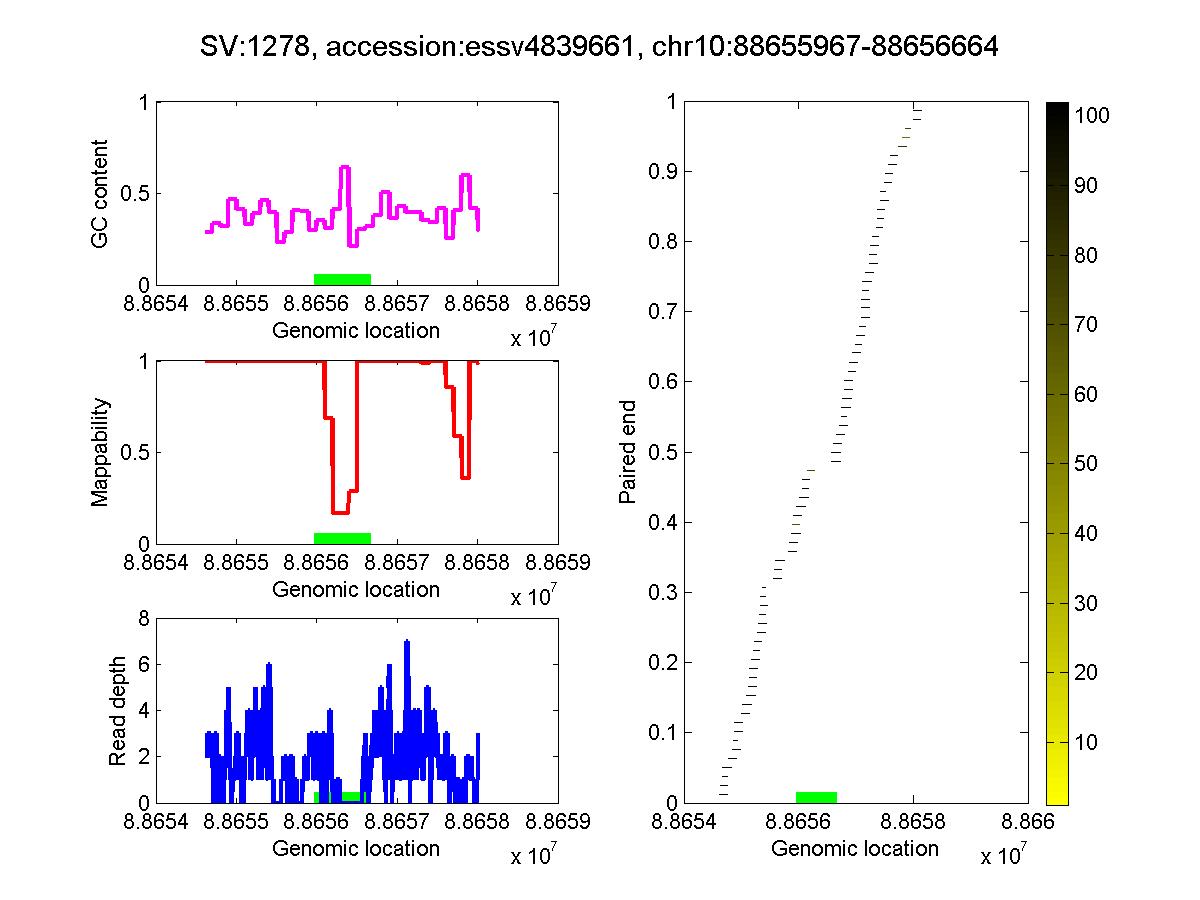

Supplement: Supplementary Materials — Supplementary data are available with this article at http://gr.xjtu.edu.cn/c/document_library/get_file?p_l_id=2403541&folderId=2539941&name=DLFE-115097.zip. Table S1 lists the complete information of suspicious variants and false positives, and the FIG directory contains the validation figures of each false positive. [file 8420547.f1.zip › 8420547.f1/FIG/SV1278.jpg]

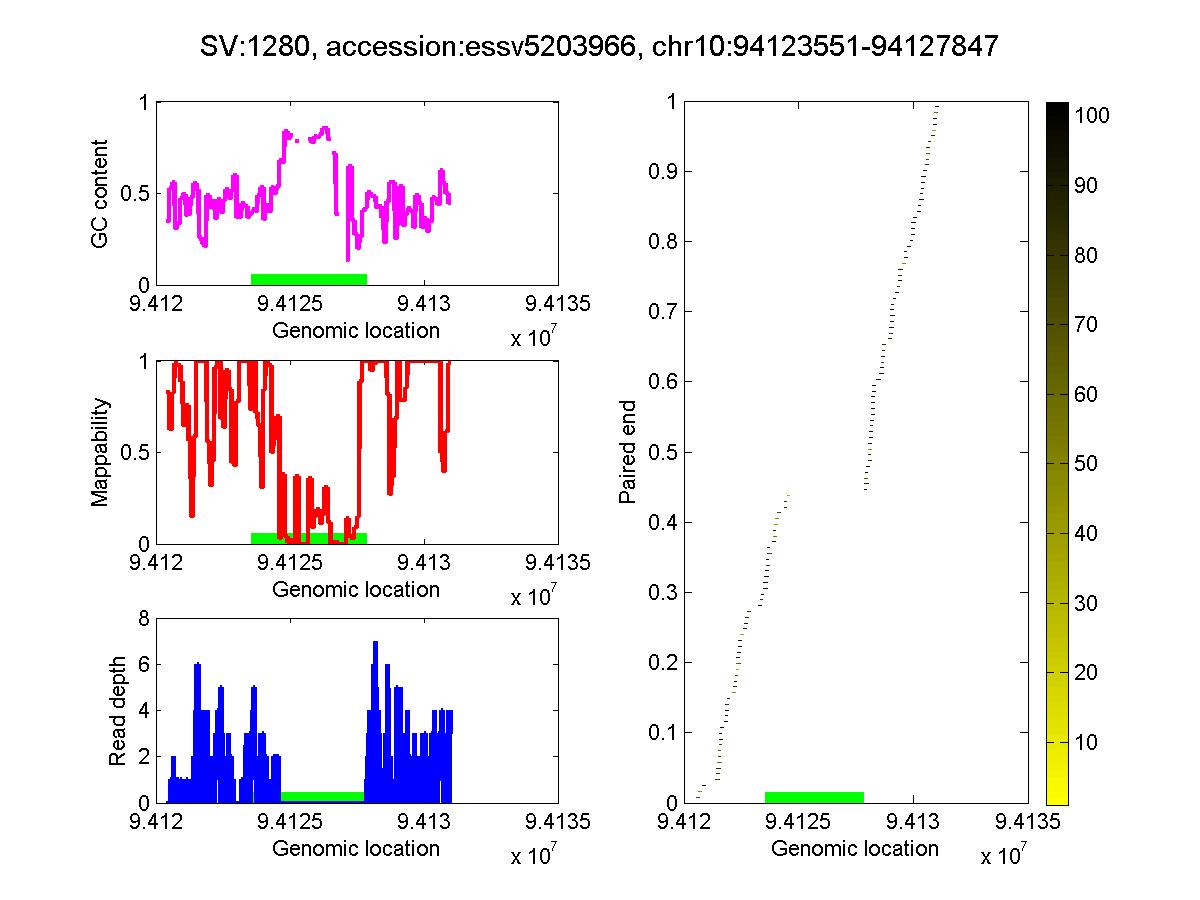

Supplement: Supplementary Materials — Supplementary data are available with this article at http://gr.xjtu.edu.cn/c/document_library/get_file?p_l_id=2403541&folderId=2539941&name=DLFE-115097.zip. Table S1 lists the complete information of suspicious variants and false positives, and the FIG directory contains the validation figures of each false positive. [file 8420547.f1.zip › 8420547.f1/FIG/SV1280.jpg]

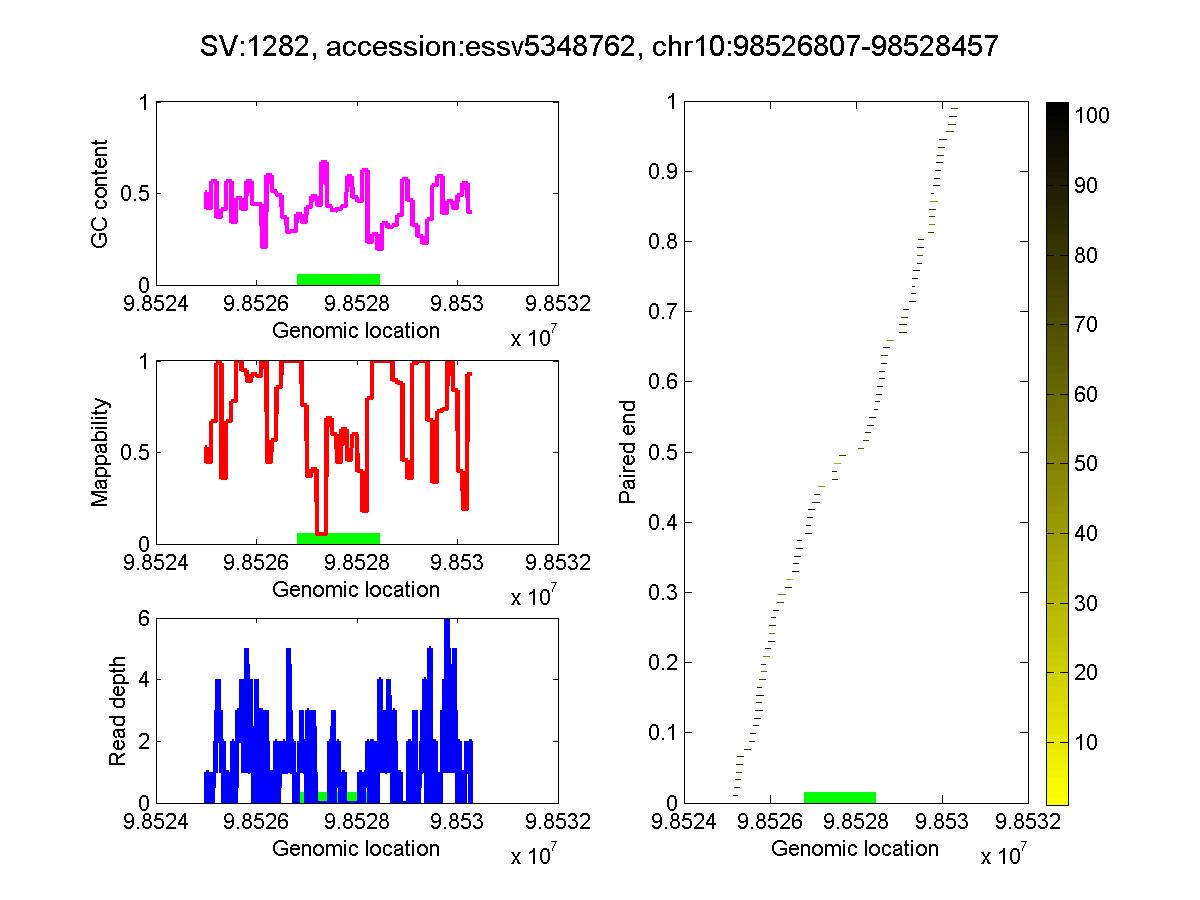

Supplement: Supplementary Materials — Supplementary data are available with this article at http://gr.xjtu.edu.cn/c/document_library/get_file?p_l_id=2403541&folderId=2539941&name=DLFE-115097.zip. Table S1 lists the complete information of suspicious variants and false positives, and the FIG directory contains the validation figures of each false positive. [file 8420547.f1.zip › 8420547.f1/FIG/SV1282.jpg]

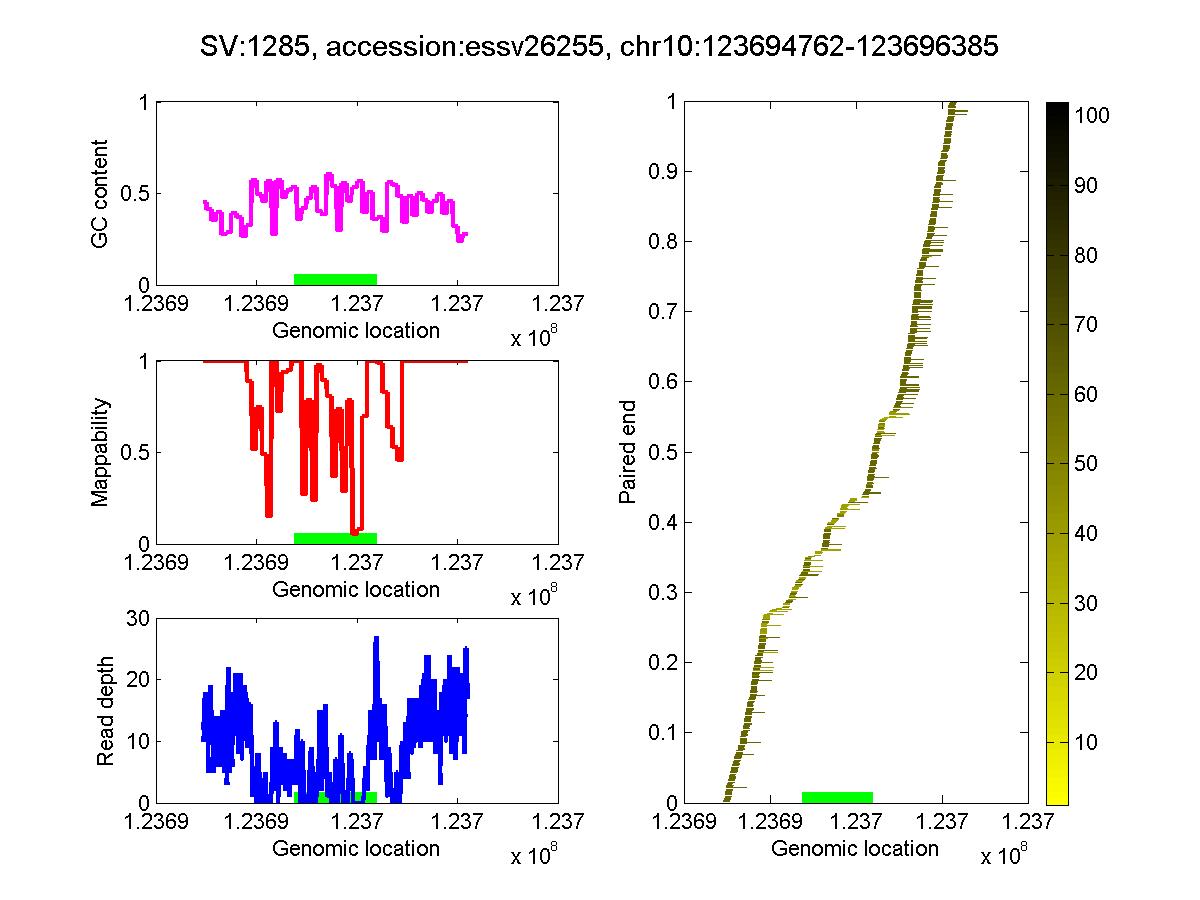

Supplement: Supplementary Materials — Supplementary data are available with this article at http://gr.xjtu.edu.cn/c/document_library/get_file?p_l_id=2403541&folderId=2539941&name=DLFE-115097.zip. Table S1 lists the complete information of suspicious variants and false positives, and the FIG directory contains the validation figures of each false positive. [file 8420547.f1.zip › 8420547.f1/FIG/SV1285.jpg]

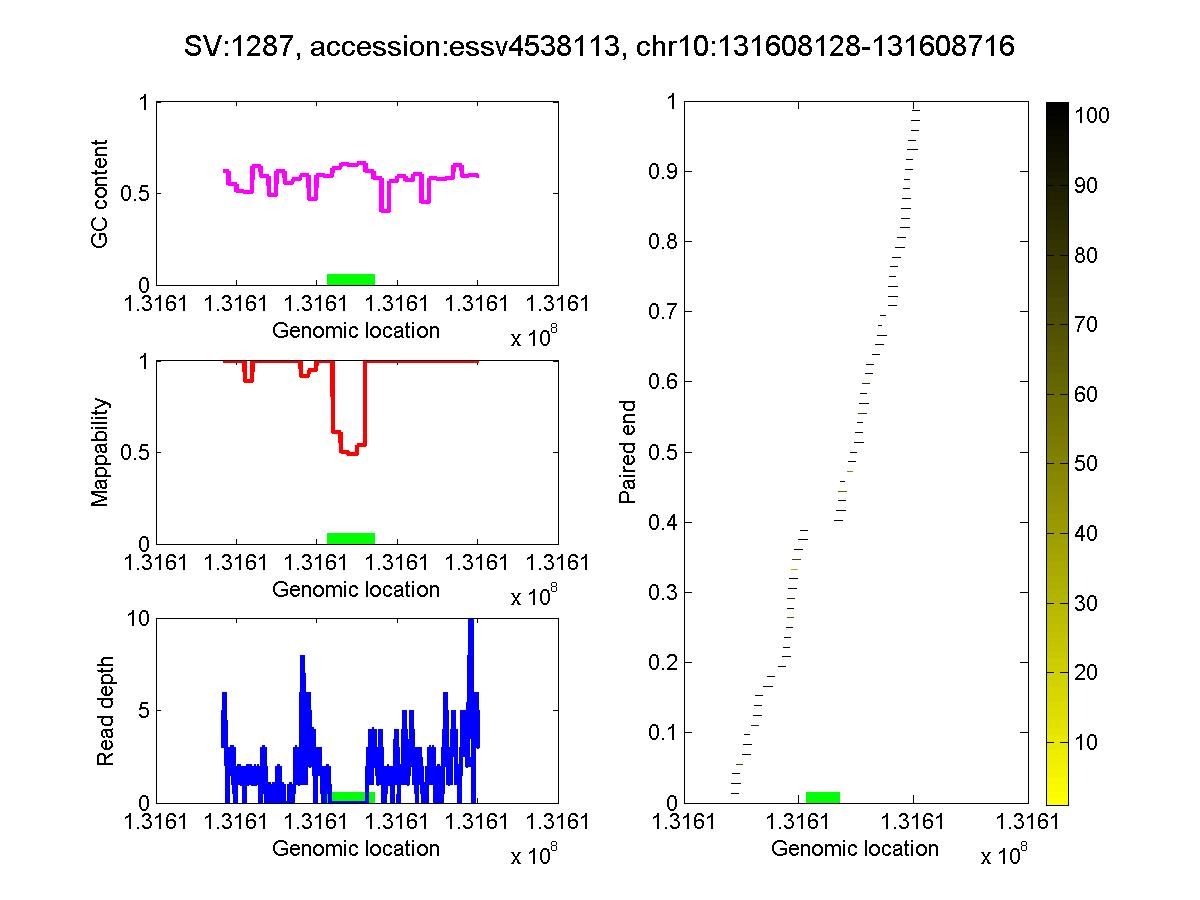

Supplement: Supplementary Materials — Supplementary data are available with this article at http://gr.xjtu.edu.cn/c/document_library/get_file?p_l_id=2403541&folderId=2539941&name=DLFE-115097.zip. Table S1 lists the complete information of suspicious variants and false positives, and the FIG directory contains the validation figures of each false positive. [file 8420547.f1.zip › 8420547.f1/FIG/SV1287.jpg]

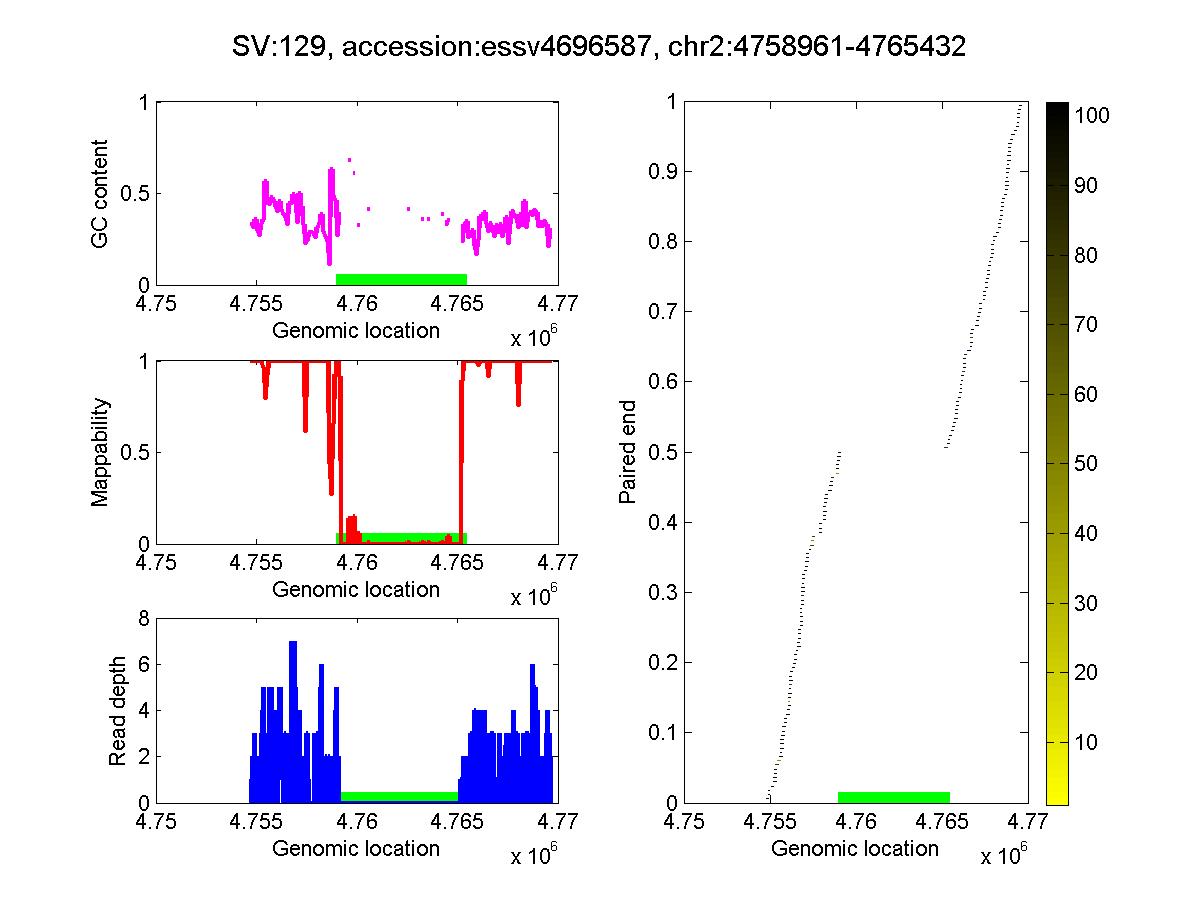

Supplement: Supplementary Materials — Supplementary data are available with this article at http://gr.xjtu.edu.cn/c/document_library/get_file?p_l_id=2403541&folderId=2539941&name=DLFE-115097.zip. Table S1 lists the complete information of suspicious variants and false positives, and the FIG directory contains the validation figures of each false positive. [file 8420547.f1.zip › 8420547.f1/FIG/SV129.jpg]

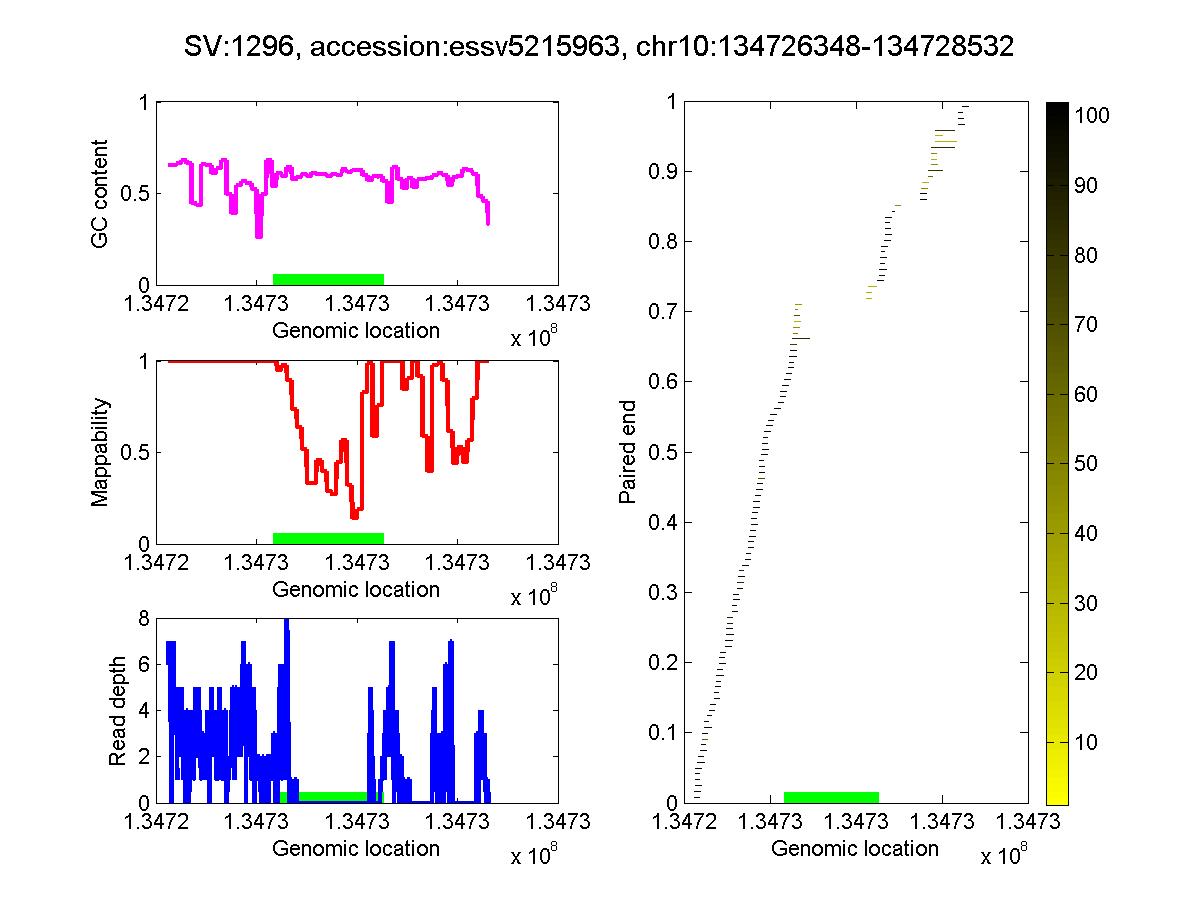

Supplement: Supplementary Materials — Supplementary data are available with this article at http://gr.xjtu.edu.cn/c/document_library/get_file?p_l_id=2403541&folderId=2539941&name=DLFE-115097.zip. Table S1 lists the complete information of suspicious variants and false positives, and the FIG directory contains the validation figures of each false positive. [file 8420547.f1.zip › 8420547.f1/FIG/SV1296.jpg]

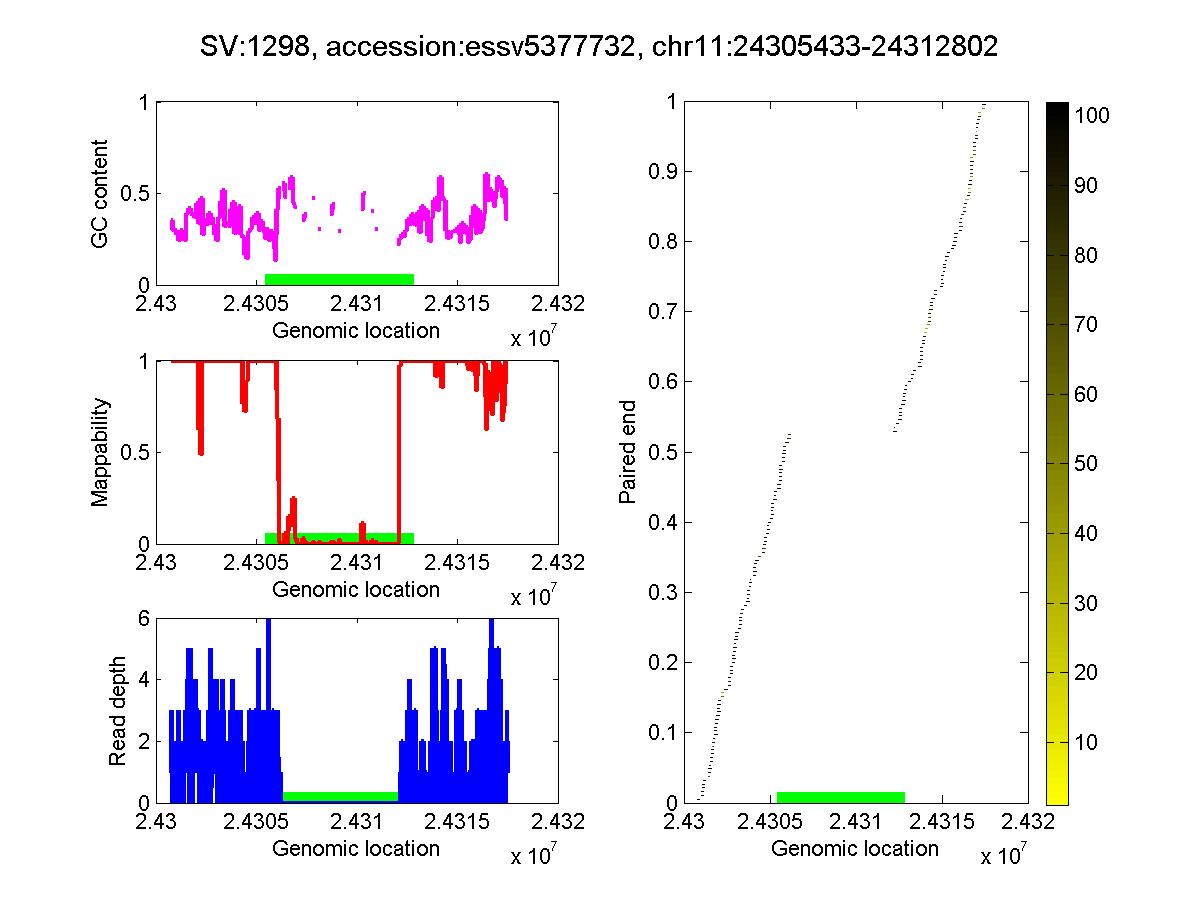

Supplement: Supplementary Materials — Supplementary data are available with this article at http://gr.xjtu.edu.cn/c/document_library/get_file?p_l_id=2403541&folderId=2539941&name=DLFE-115097.zip. Table S1 lists the complete information of suspicious variants and false positives, and the FIG directory contains the validation figures of each false positive. [file 8420547.f1.zip › 8420547.f1/FIG/SV1298.jpg]

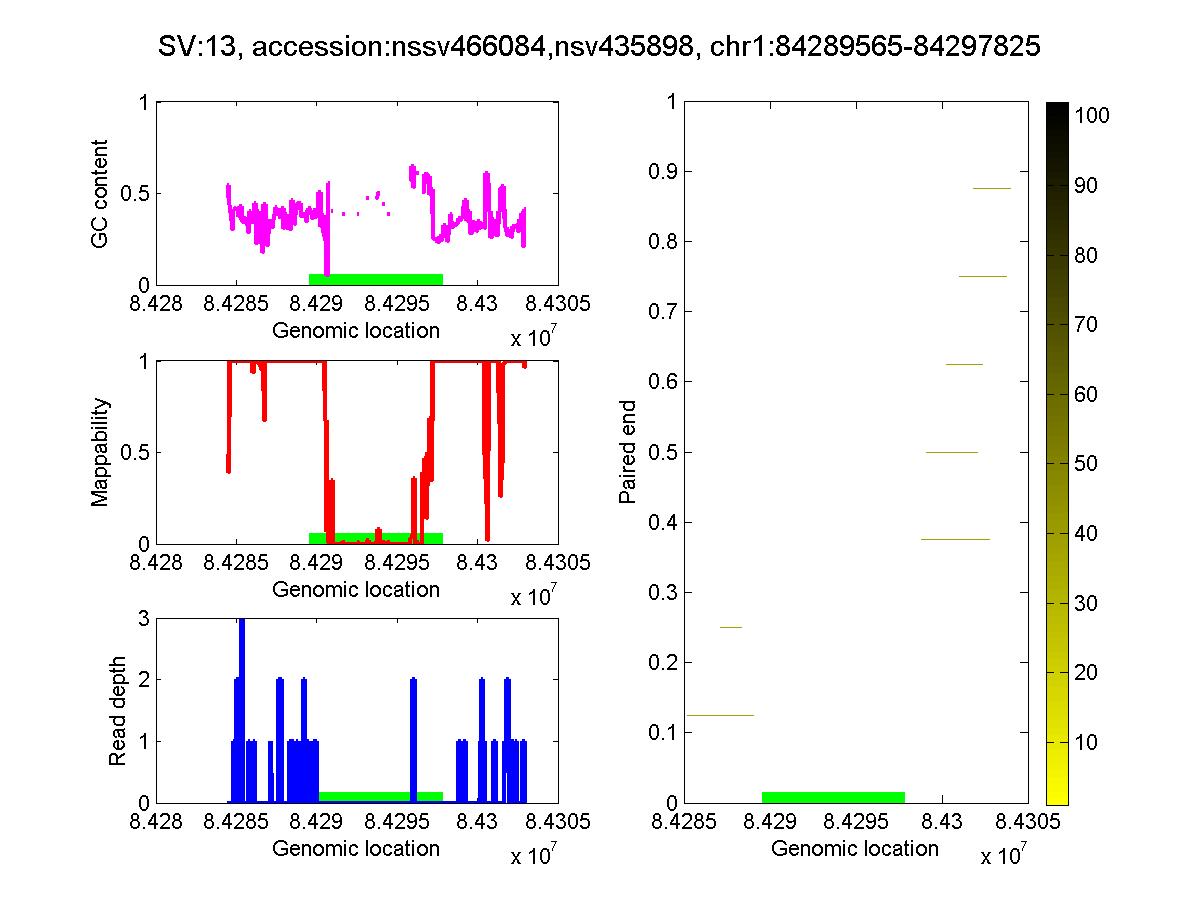

Supplement: Supplementary Materials — Supplementary data are available with this article at http://gr.xjtu.edu.cn/c/document_library/get_file?p_l_id=2403541&folderId=2539941&name=DLFE-115097.zip. Table S1 lists the complete information of suspicious variants and false positives, and the FIG directory contains the validation figures of each false positive. [file 8420547.f1.zip › 8420547.f1/FIG/SV13.jpg]

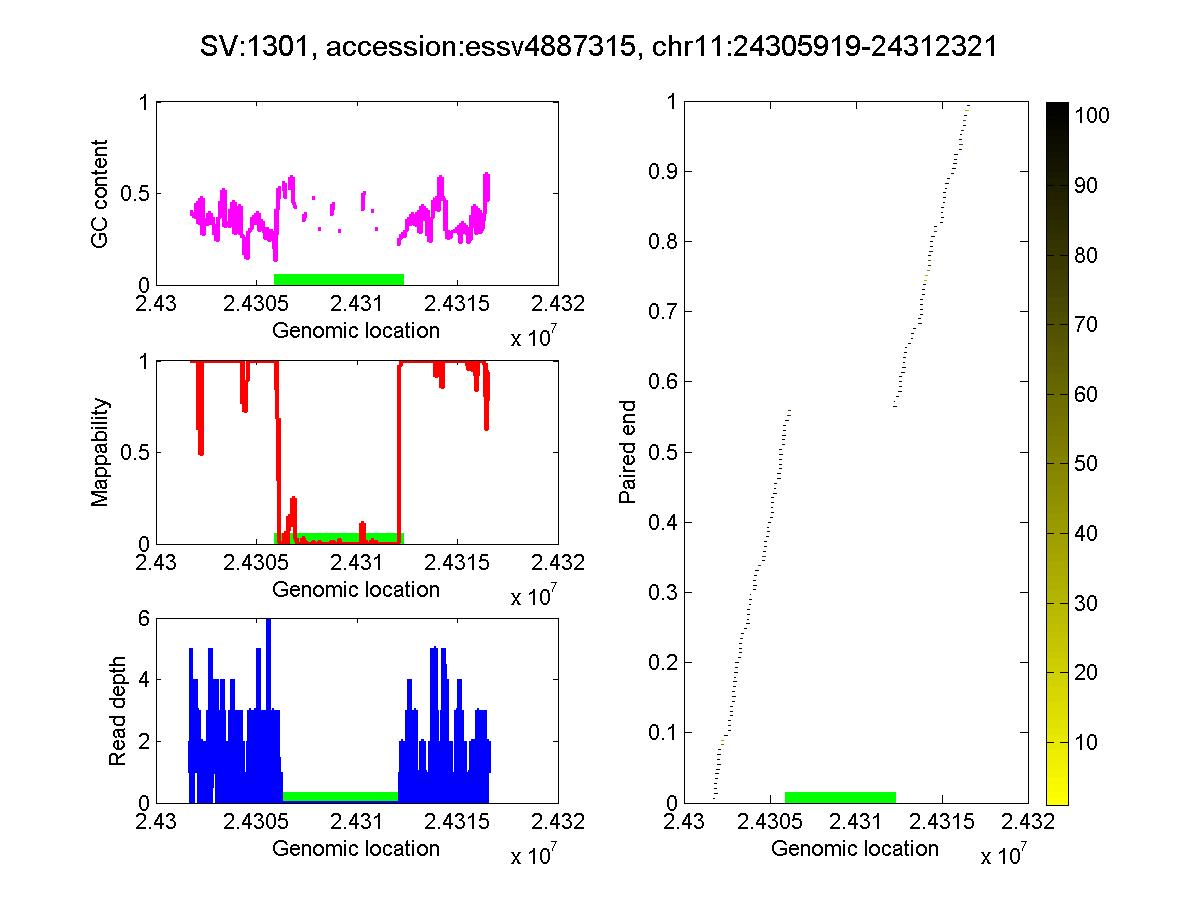

Supplement: Supplementary Materials — Supplementary data are available with this article at http://gr.xjtu.edu.cn/c/document_library/get_file?p_l_id=2403541&folderId=2539941&name=DLFE-115097.zip. Table S1 lists the complete information of suspicious variants and false positives, and the FIG directory contains the validation figures of each false positive. [file 8420547.f1.zip › 8420547.f1/FIG/SV1301.jpg]

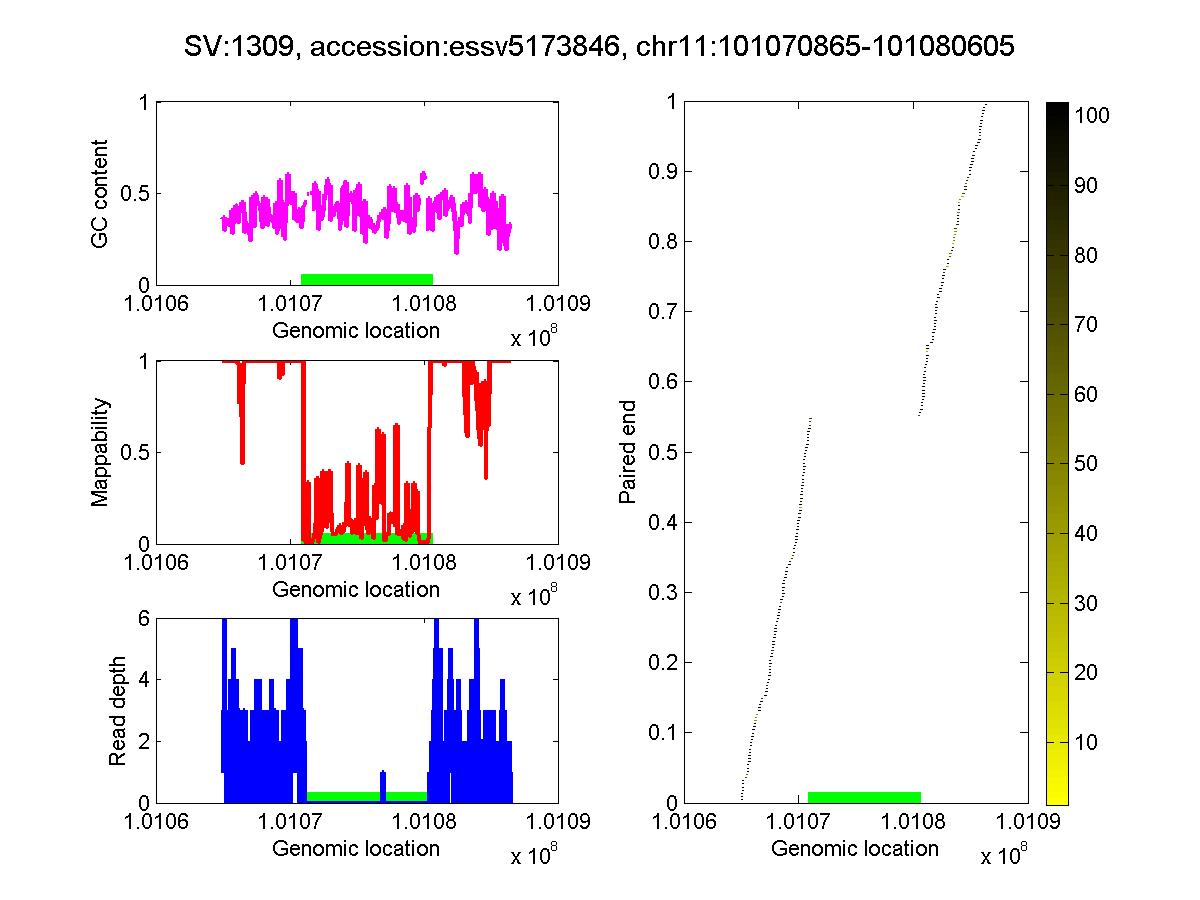

Supplement: Supplementary Materials — Supplementary data are available with this article at http://gr.xjtu.edu.cn/c/document_library/get_file?p_l_id=2403541&folderId=2539941&name=DLFE-115097.zip. Table S1 lists the complete information of suspicious variants and false positives, and the FIG directory contains the validation figures of each false positive. [file 8420547.f1.zip › 8420547.f1/FIG/SV1309.jpg]

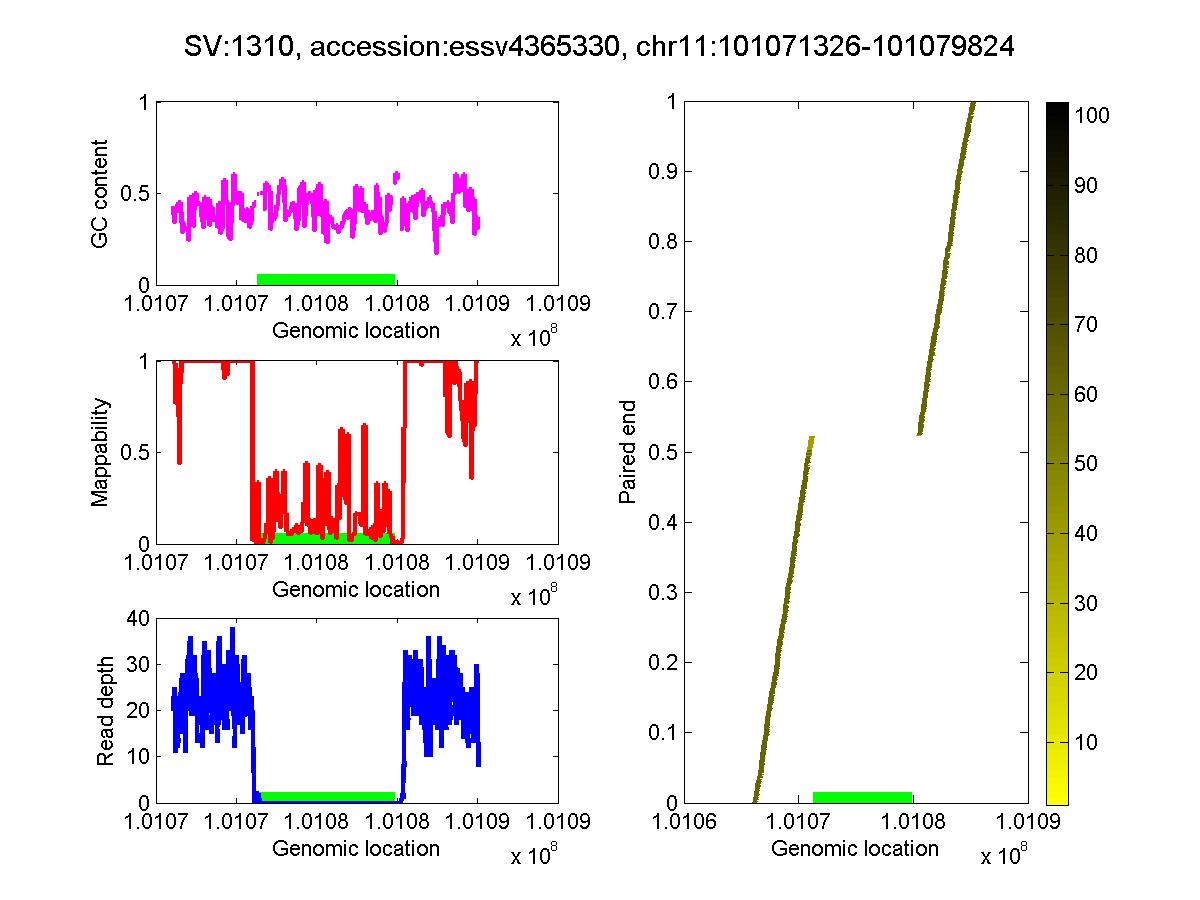

Supplement: Supplementary Materials — Supplementary data are available with this article at http://gr.xjtu.edu.cn/c/document_library/get_file?p_l_id=2403541&folderId=2539941&name=DLFE-115097.zip. Table S1 lists the complete information of suspicious variants and false positives, and the FIG directory contains the validation figures of each false positive. [file 8420547.f1.zip › 8420547.f1/FIG/SV1310.jpg]

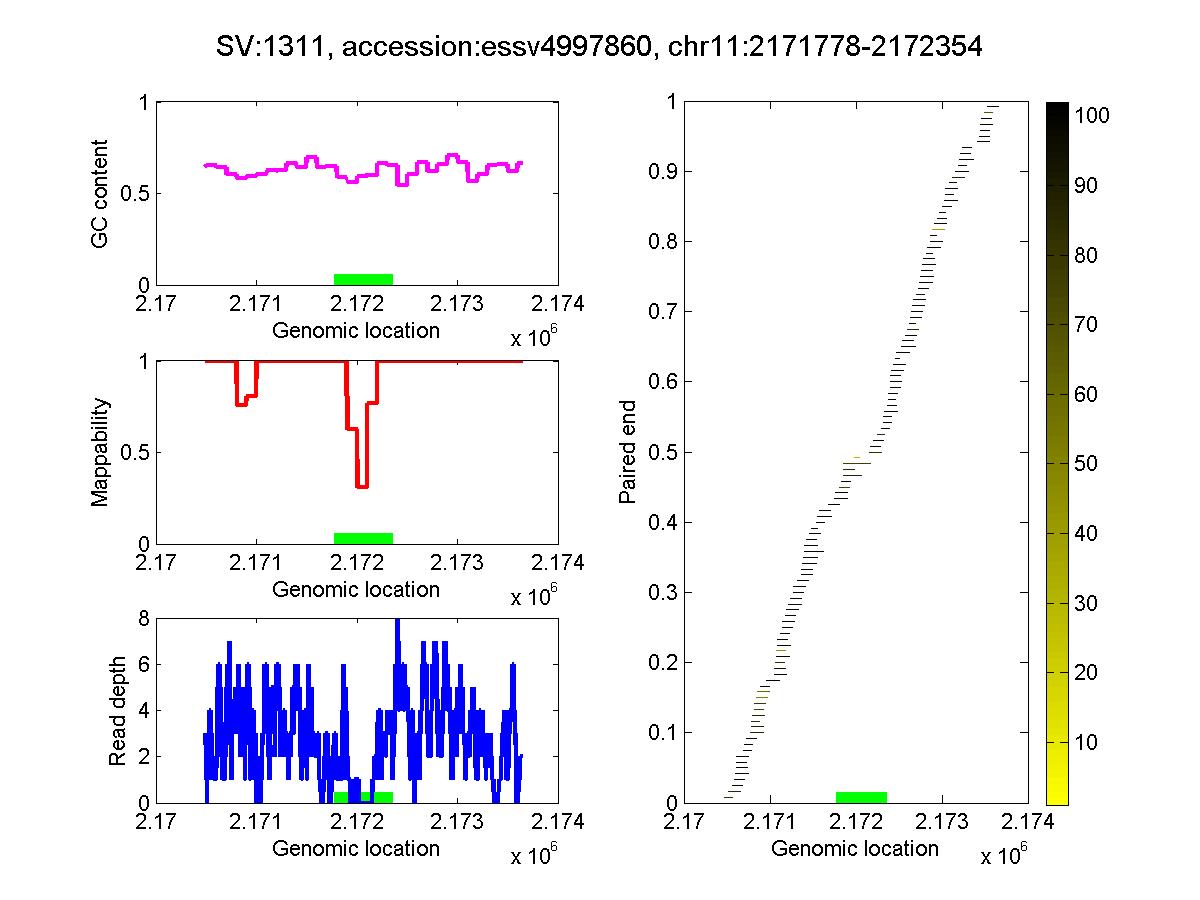

Supplement: Supplementary Materials — Supplementary data are available with this article at http://gr.xjtu.edu.cn/c/document_library/get_file?p_l_id=2403541&folderId=2539941&name=DLFE-115097.zip. Table S1 lists the complete information of suspicious variants and false positives, and the FIG directory contains the validation figures of each false positive. [file 8420547.f1.zip › 8420547.f1/FIG/SV1311.jpg]

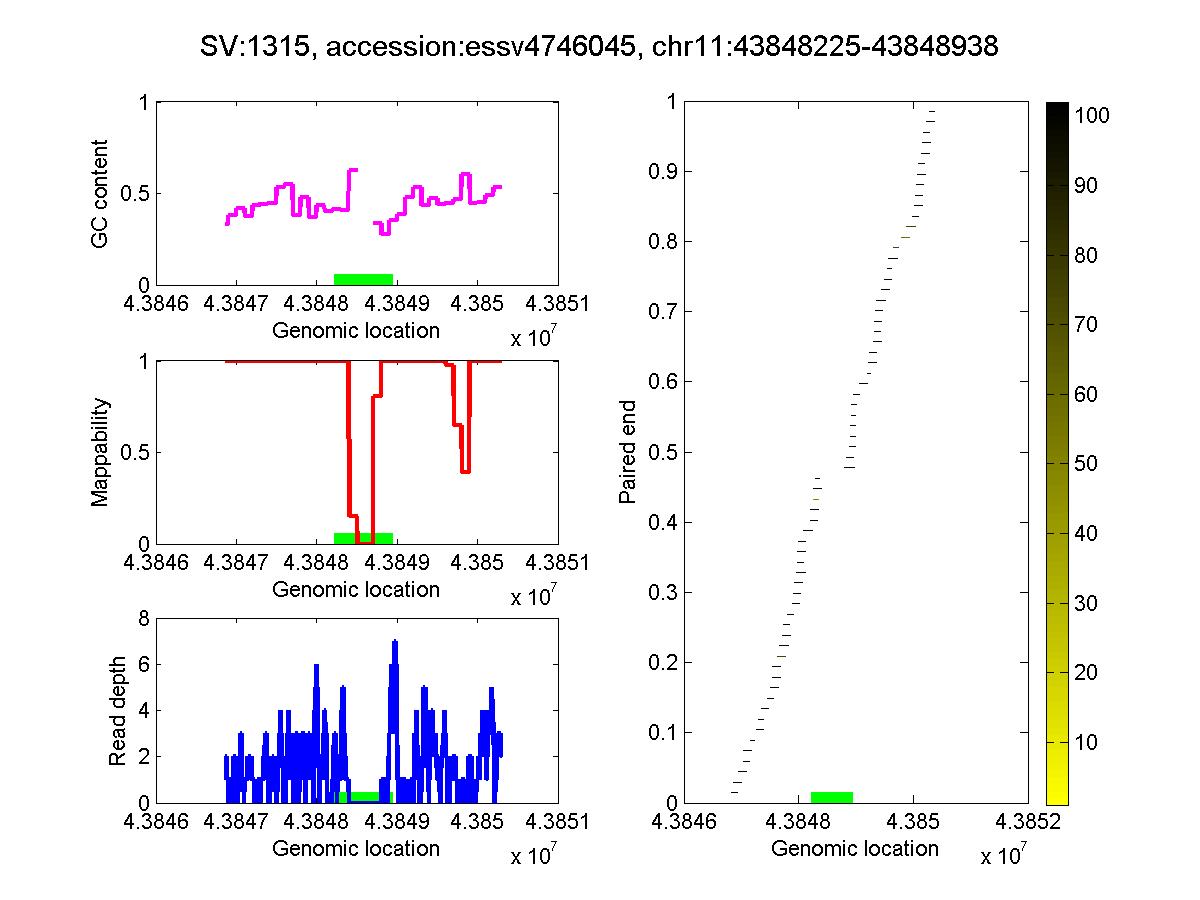

Supplement: Supplementary Materials — Supplementary data are available with this article at http://gr.xjtu.edu.cn/c/document_library/get_file?p_l_id=2403541&folderId=2539941&name=DLFE-115097.zip. Table S1 lists the complete information of suspicious variants and false positives, and the FIG directory contains the validation figures of each false positive. [file 8420547.f1.zip › 8420547.f1/FIG/SV1315.jpg]

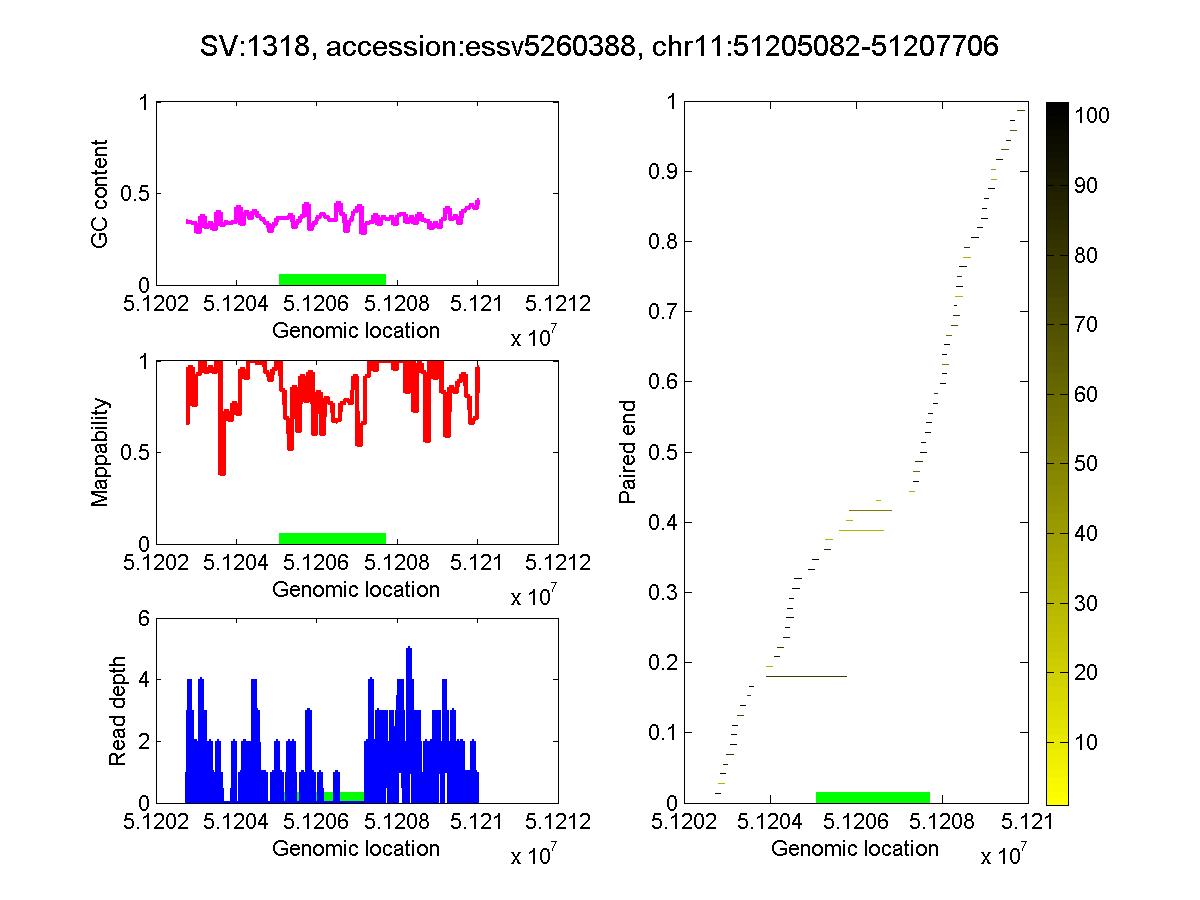

Supplement: Supplementary Materials — Supplementary data are available with this article at http://gr.xjtu.edu.cn/c/document_library/get_file?p_l_id=2403541&folderId=2539941&name=DLFE-115097.zip. Table S1 lists the complete information of suspicious variants and false positives, and the FIG directory contains the validation figures of each false positive. [file 8420547.f1.zip › 8420547.f1/FIG/SV1318.jpg]

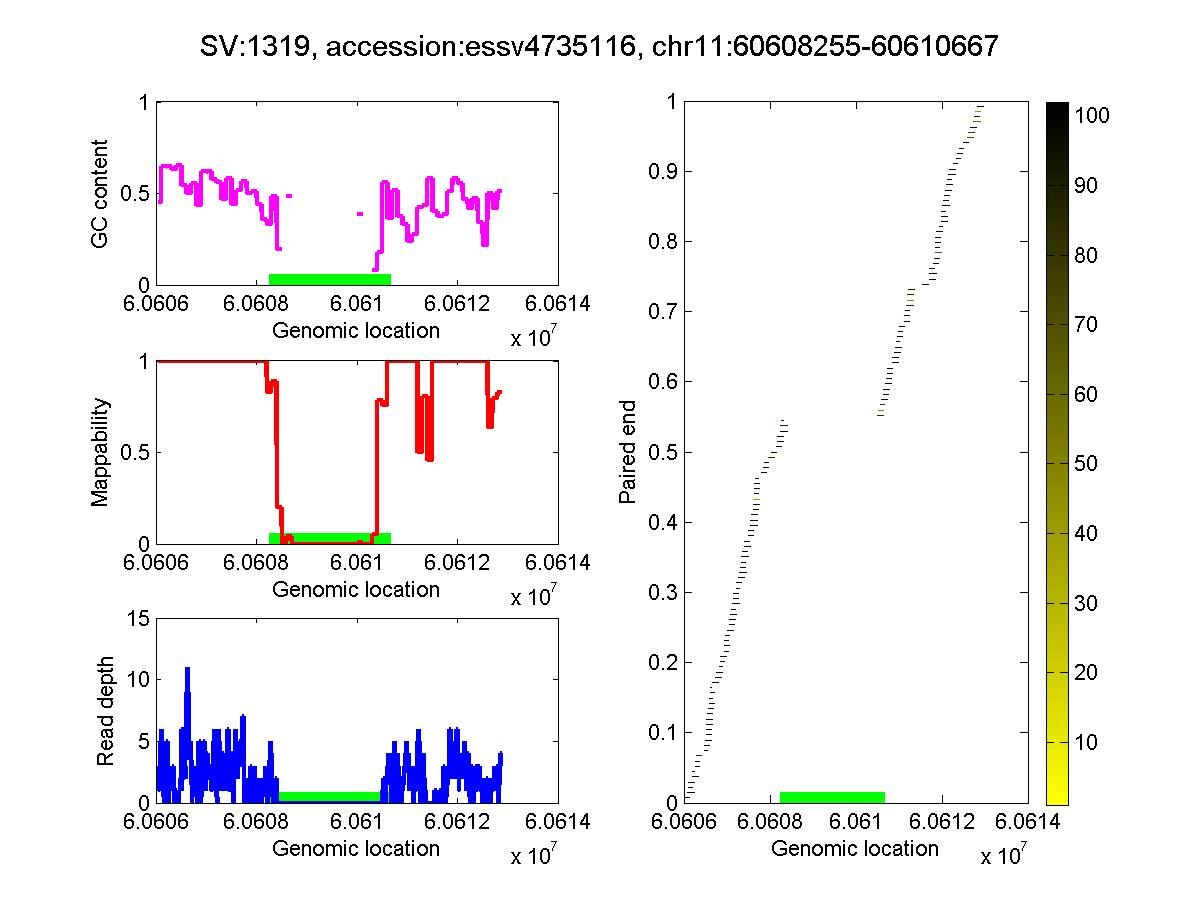

Supplement: Supplementary Materials — Supplementary data are available with this article at http://gr.xjtu.edu.cn/c/document_library/get_file?p_l_id=2403541&folderId=2539941&name=DLFE-115097.zip. Table S1 lists the complete information of suspicious variants and false positives, and the FIG directory contains the validation figures of each false positive. [file 8420547.f1.zip › 8420547.f1/FIG/SV1319.jpg]

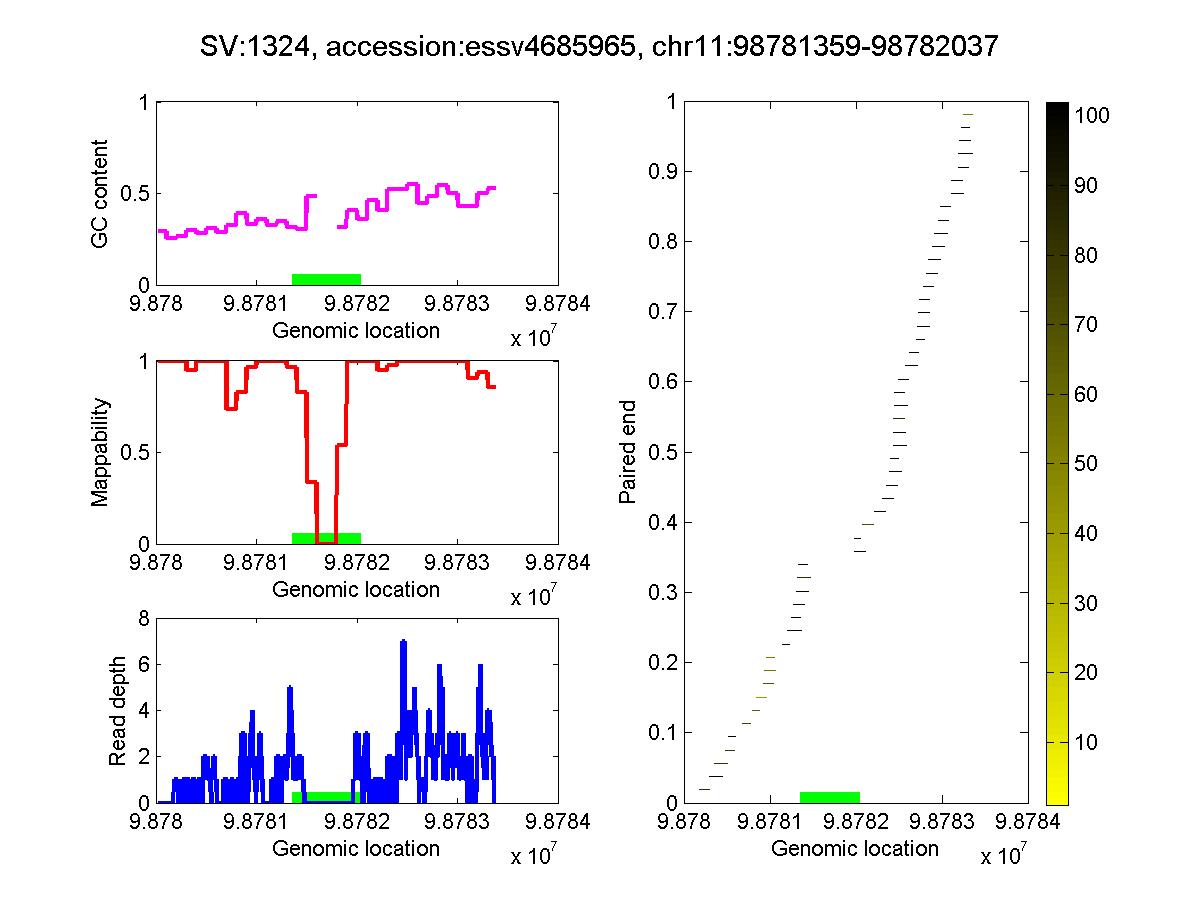

Supplement: Supplementary Materials — Supplementary data are available with this article at http://gr.xjtu.edu.cn/c/document_library/get_file?p_l_id=2403541&folderId=2539941&name=DLFE-115097.zip. Table S1 lists the complete information of suspicious variants and false positives, and the FIG directory contains the validation figures of each false positive. [file 8420547.f1.zip › 8420547.f1/FIG/SV1324.jpg]

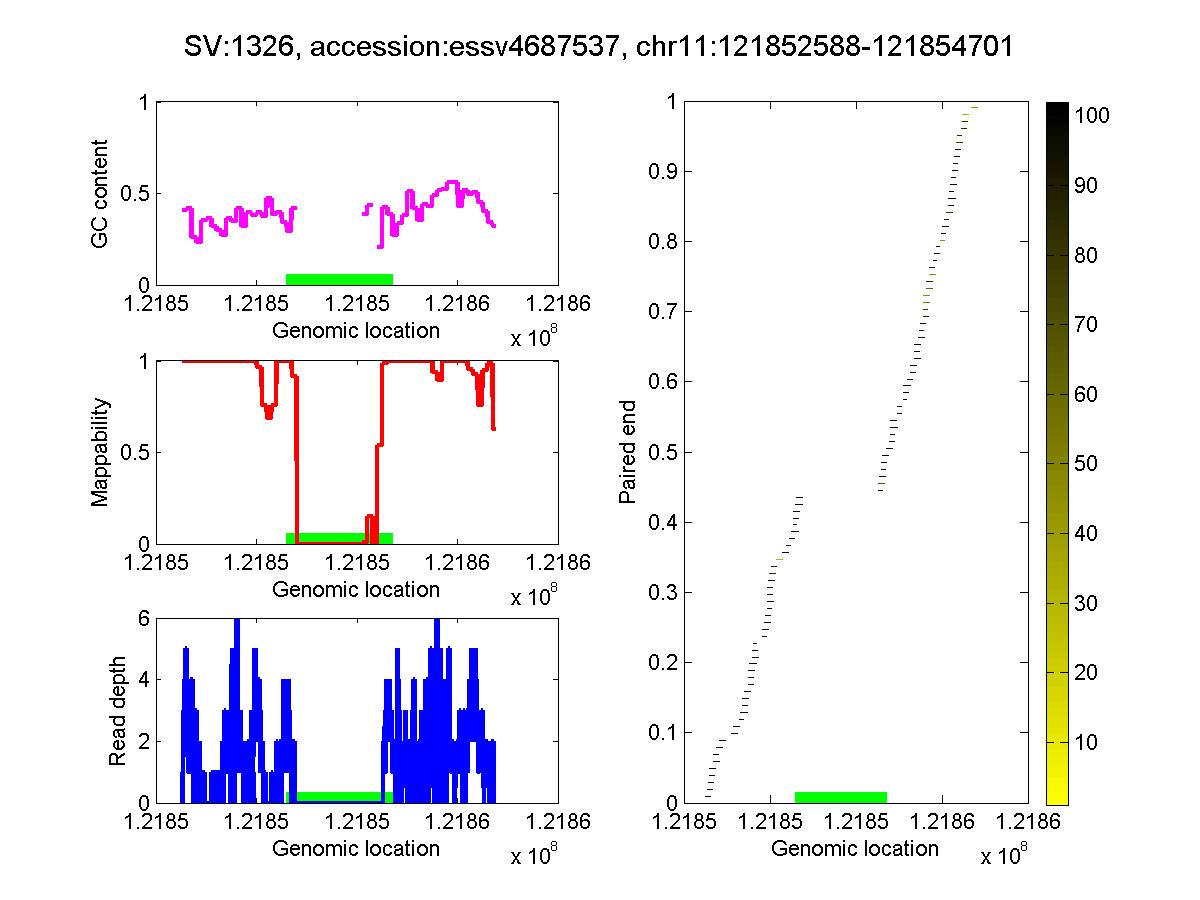

Supplement: Supplementary Materials — Supplementary data are available with this article at http://gr.xjtu.edu.cn/c/document_library/get_file?p_l_id=2403541&folderId=2539941&name=DLFE-115097.zip. Table S1 lists the complete information of suspicious variants and false positives, and the FIG directory contains the validation figures of each false positive. [file 8420547.f1.zip › 8420547.f1/FIG/SV1326.jpg]

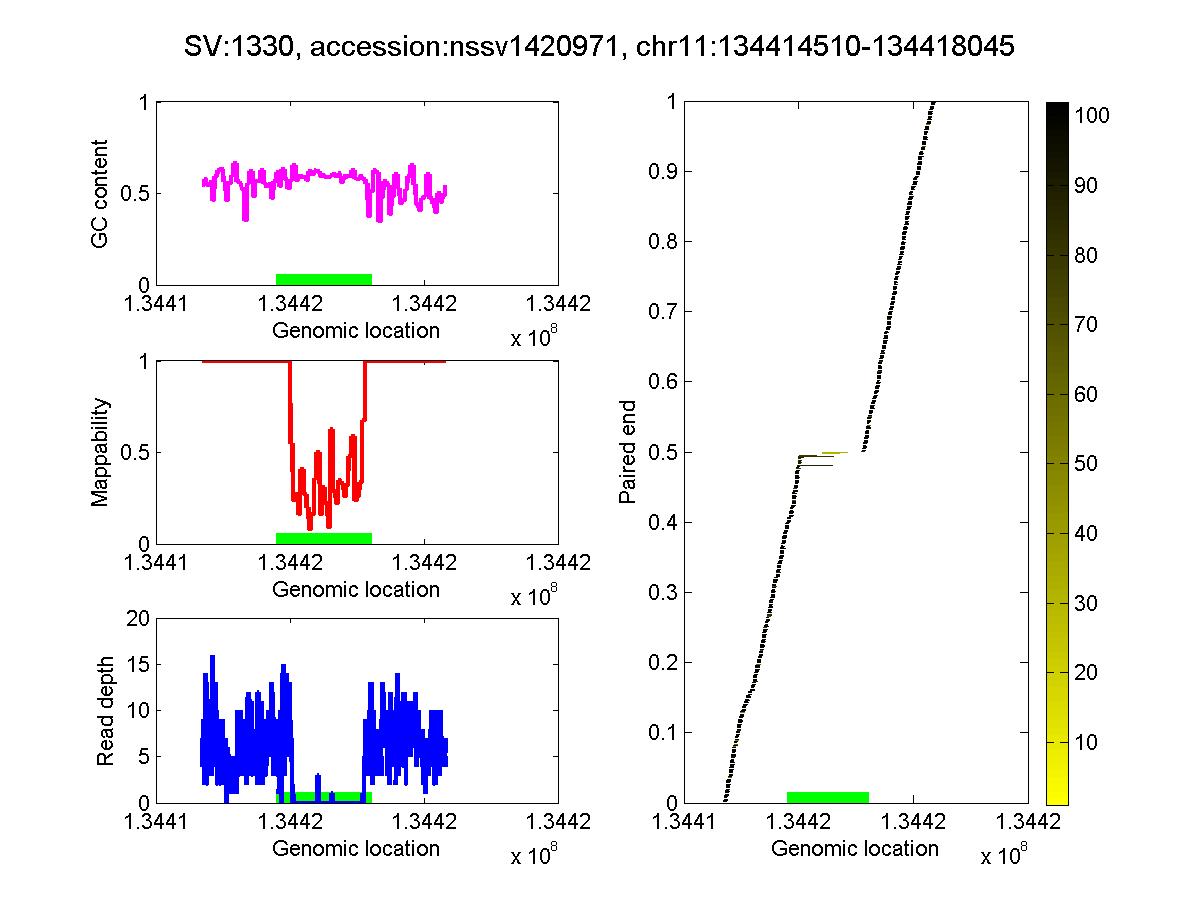

Supplement: Supplementary Materials — Supplementary data are available with this article at http://gr.xjtu.edu.cn/c/document_library/get_file?p_l_id=2403541&folderId=2539941&name=DLFE-115097.zip. Table S1 lists the complete information of suspicious variants and false positives, and the FIG directory contains the validation figures of each false positive. [file 8420547.f1.zip › 8420547.f1/FIG/SV1330.jpg]

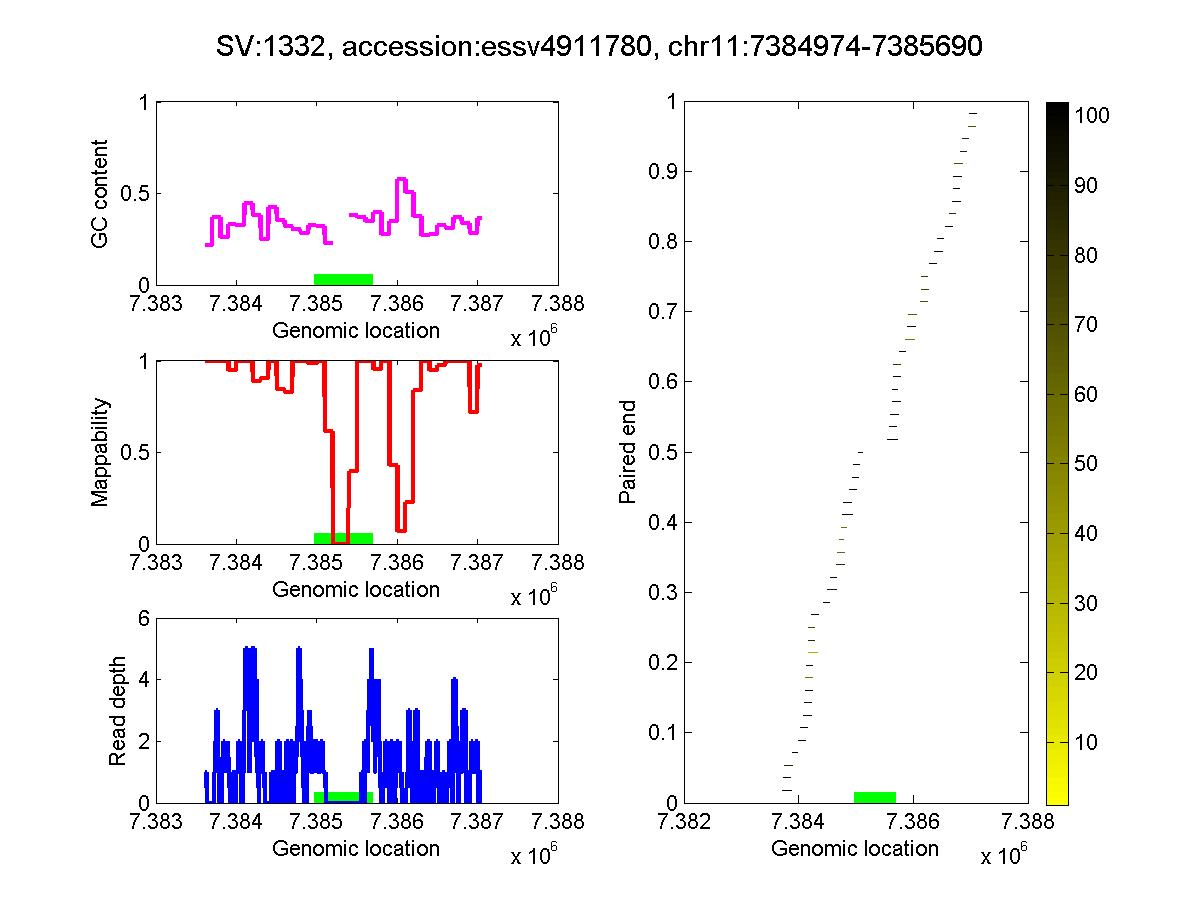

Supplement: Supplementary Materials — Supplementary data are available with this article at http://gr.xjtu.edu.cn/c/document_library/get_file?p_l_id=2403541&folderId=2539941&name=DLFE-115097.zip. Table S1 lists the complete information of suspicious variants and false positives, and the FIG directory contains the validation figures of each false positive. [file 8420547.f1.zip › 8420547.f1/FIG/SV1332.jpg]
